# Somatic mutations in the DNA repairome in prostate cancers in African Americans and Caucasians

**DOI:** 10.1038/s41388-020-1280-x

**Published:** 2020-04-16

**Authors:** Santosh Yadav, Muralidharan Anbalagan, Melody Baddoo, Vinodh K. Chellamuthu, Sudurika Mukhopadhyay, Carol Woods, Wei Jiang, Krzysztof Moroz, Erik K Flemington, Nick Makridakis

**Affiliations:** 1 Louisiana Cancer Research Center, New Orleans, LA USA; 2 Tulane Center for Aging, Department of Medicine, Tulane University, New Orleans, LA USA; 3 Department of Structural and Cellular Biology, Tulane University, New Orleans, LA USA; 4 Department of Pathology, Tulane University, New Orleans, LA USA; 5 Department of Mathematics, Dixie State University, St. George, UT USA; 6 1Globe Health Institute, Boston, MA USA

**Keywords:** Cancer genomics, Cancer genetics

## Abstract

Most hereditary tumors show aberrations in DNA repair genes or their regulators. In contrast, only a minority of sporadic tumors show alterations in these genes. As a result, genomic instability is currently considered an enhancer of tumorigenesis rather than an obligatory event in this process. However, tumor heterogeneity presents a significant technical challenge for most cancer genomics studies performed at less than 100× mean resolution depth. To address the importance of genomic instability in prostate carcinogenesis and tumor progression, we performed ultrahigh depth exome sequencing of 124 DNA damage repair/response (repairome) genes in 63 tumors and matched normal tissue samples in African Americans and Caucasians. The average sequence depth was 712-fold for DNA isolated from normal tissue and 368-fold for FFPE tumors. We identified 671 somatic mutations in tumors from African Americans and 762 somatic mutations in tumors in Caucasians. The most frequently mutated DNA repairome genes were *EXO1, ATR, POLQ, NEIL3, ERCC6, BRCA2, BRCA1, XPC, JAG1, RPA1, POLE, ATM*, and *LIG1* in African American men, and *POLQ, NEIL3, POLB, BRCA2, EXO1, ERCC6, ATR, RBBP8, BRCA1, ATM, JAG1, XPC*, and *POLE* in Caucasians. We found that 89% of tumors had at least one mutation in nucleotide excision repair pathway genes in African Americans, whereas >40% of tumors had mutations in base excision repair pathway genes in Caucasians. We further identified a marginal increase in mutation rate in tumors in African Americans with increasing age. Tumors in Caucasians did not show a correlation with age, but a progressive increase in the mutation rate was observed at higher Gleason scores. Our data reveal significant differences in the molecular signatures in the DNA repairome in prostate cancer between African Americans and Caucasians. These data also have substantial implications regarding the well-known health disparities in prostate cancer, such as the higher mortality in African Americans than Caucasians.

## Introduction

Prostate cancer (PCa) is the most common type of cancer in men in USA (164,690 estimated new cases and 29,430 mortalities in 2018, according to the Surveillance, Epidemiology, and End Results database, National Cancer Institute). African American men have the highest incidence of PCa of any other racial group in the United States [1, 2] and the highest rates of aggressive disease and mortality [3, 4]. According to GLOBOCAN 2018, globally, African American men have the highest incidence rates and more aggressive types of PCa than Caucasian men. In contrast, DeSantis et al. (2019) have recently reported that overall cancer death rates declined faster in African Americans than Caucasians among both males (2.6% vs 1.6% per year) and females (1.5 vs 1.3% per year), a finding primarily driven by more significant declines related to lung cancer, colorectal cancer, and PCa (American Cancer Society) [5]. The extent of genetic instability varies among tumors of distinct racial groups, owing to genetic factors, lifestyle-related factors, and occupation [6, 7]. Genetic DNA repair alterations may thus play a crucial role in explaining the racial differences in PCa. The inherent genetic/genomic instability of prostate tumors has been studied in numerous investigations [8–10]. However, why some tumors are more unstable than others, or what exactly causes this instability, remains unclear.

DNA is highly vulnerable to chemical modifications, which can cause several types of DNA lesions, such as double-strand DNA breaks, base loss, or base modification. These lesions can occur endogenously or because of exposure to environmental toxins [11, 12]. Cells have thus evolved a series of mechanisms tailored to maintaining genomic integrity [13, 14]. We have previously provided evidence that three DNA repair polymerases, pol β, η, and κ, are often somatically mutated in PCa, and some of these mutations affect enzyme activity, catalytic efficiency, or the fidelity of DNA synthesis [15–17]. The incidence of germline mutations in DNA-repair genes among men is higher in those with metastatic PCa than localized PCa [18].

Most experts agree that genomic instability plays a role during cancer evolution, but its exact role is debated [19]. Two types of genomic instability are evident in most cancers: large-scale alterations (e.g., aneuploidy or translocations) and small-scale alterations (e.g., single-nucleotide substitutions or microsatellite instability). The extent of somatic alterations has been examined with both PCR- and non-PCR-based methods: most tumors display low rates of genomic instability [19–21], although cancers arising from a mutator phenotype can contain as many as 142,000 alterations per tumor cell [22].

Regardless of the method used, quantifying the somatic instability of heterogeneous tumor genomes is challenging [23, 24]. To address the importance of genomic instability in DNA repair processes for prostate carcinogenesis, we deep sequenced the exons of 124 genes involved in DNA damage repair/response in 63 PCa tumors and matched healthy tissue (peripheral blood lymphocytes or adjacent normal tissue), at very high resolution. We selected the DNA repair/damage response pathway because mutations targeting this pathway are more likely to cause a mutator phenotype than mutations in other pathways. We report that somatic alteration of DNA repair/response (repairome) genes is nearly obligatory in prostate tumors. However, somatic mutations differ among racial groups.

## Results

### Clinical characteristics of the samples

We sequenced DNA extracted from 63 prostate tumors and matched control tissue from the same patient; 61.9% (*n* = 39) of the patients were Caucasian, and 38% (*n* = 24) were African American. We included patients diagnosed between the ages of 46 and 89 years (mean 60 ± 6.2 years) with Gleason scores between 6 and 9, and PSA scores from 2 to 26 (Table 1).

**Table 1:** Demographic and clinical characteristics of the prostate cancer samples of African Americans and Caucasians.

ID	Age	PSA	Gleason score	Mutations
AA-1	54	6	7	28
AA-2	63	5.1	7	57
AA-3	52	NR	7	49
AA-4	57	4.6	7	28
AA-5	55	3	7	68
AA-6	57	4.1	6	61
AA-7	54	9.7	6	18
AA-8	68	6.9	6	42
AA-9	66	5.8	8	62
AA-10	51	NR	NR	49
AA-11	62	4.6	7	7
AA-12	46	NR	8	35
AA-13	58	5.3	7	4
AA-14	70	9.53	7	19
AA-15	60	16.8	8	32
AA-16	64	31	7	*20*
AA-17	59	26	9	11
AA-18	54	9.7	6	39
AA-19	72	13.4	7	41
W-20	65	12.5	7	21
W-21	64	9.5	9	9
W-22	61	4	7	11
W-23	74	7.5	6	24
W-24	57	4.51	7	3
W-25	71	2.9	7	3
W-26	61	NR	7	83
W-27	65	12.5	7	31
W-28	63	NR	7	23
W-29	47	4.65	7	34
W-30	61	7	8	17
W-31	65	12.9	7	10
W-32	52	2	7	53
W-33	62	8.4	6	37
W-34	64	12.4	7	15
W-35	68	18	6	4
W-36	54	9.7	6	18
W-37	64	9.5	7	9
W-38	61	4	7	11
W-39	74	7.5	6	24
W-40	57	4.51	7	3
W-41	71	2.9	7	3
W-42	61	3.2	7	83
W-43	65	12.5	7	31
W-44	63	NR	7	23
W-45	47	4.65	7	34
W-46	61	12.2	8	17
W-47	65	12.9	7	10
W-48	52	2	7	53
W-49	62	8.4	6	37
W-50	64	12.4	7	15
W-51	68	18	6	4
W-52	54	9.7	6	18

To examine somatic mutations in DNA damage response and repair genes in PCas, we performed systematic somatic mutation analysis of DNA repair/damage response genes using SureSelect custom target enrichment, after tissue microdissection and DNA extraction. Illumina paired-end libraries were prepared from the captured target regions and sequenced on HiSeq 2000 or HiSeq 2500 Sequencing System (Illumina) with 99 bp reads. Somatic variants were called with VarScan. Six FFPE samples from African Americans and five samples from Caucasians did not pass the quality control and were excluded from the analysis. We thus analyzed somatic mutations in 19 tumors in African Americans and 33 tumors in Caucasians, in addition to matched normal tissue.

**Fig. 1:**
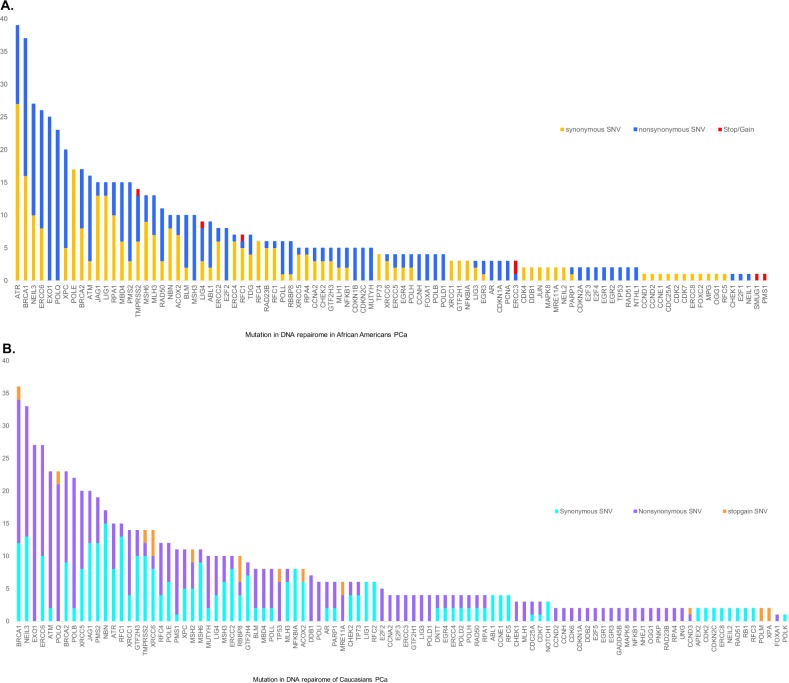
Number of somatic mutations in DNA repairome genes in African Americans and Caucasians PCa. **a** All somatic mutations (SNVs, non-SNVs, and stop codons) identified in PCa samples from African Americans. **b** All somatic mutations (SNVs, non-SNVs, and stop codons) identified in PCa samples from Caucasians. Validation status was determined by comparing tumor/normal read counts for each allele with VarScan. For validation, we had more than 50 reads with base quality ≥15 (Phred score) for both normal and tumor samples. The somatic *p* value significance threshold was set as <0.05 and was calculated by VarScan with Fisher’s exact test.

We identified 671 somatic point mutations in tumors in African Americans; 344 were predicted to generate missense mutations, 321 were predicted to generate nonsense mutations, 5 were predicted to generate stop-gain mutations, and 1 was predicted to generate stop loss codon read-through (Fig. 1a). We identified 762 somatic point mutations in the Caucasian cohort; 425 were predicted to generate missense mutations, 306 were predicted to generate nonsense mutations, 27 were predicted to generate stop codon read-through, and 4 were predicted to generate splicing (Fig. 1b).

**Fig. 2:**
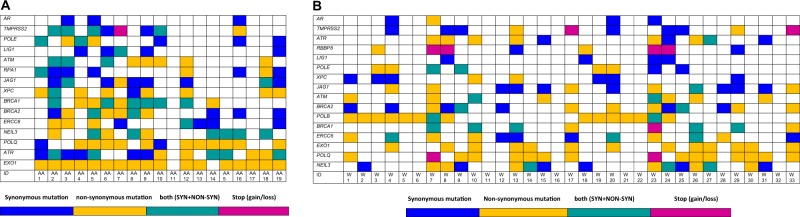
Landscape of the top most mutated DNA repairome genes in PCa. **a** Somatic mutations identified in the DNA repairome in African Americans PCa (*n* = 19). **b** Somatic mutations identified in the DNA repairome in Caucasians PCa (*n* = 33). Samples are ordered in columns, and mutated genes are displayed in rows. Color indicates the type of mutation: non synonymous (yellow), synonymous (blue), both (sea green), and stop-gain/loss (magenta).

The most frequently mutated DNA repairome genes in African Americans tumors were *EXO1* (89%), *ATR* (73%), *POLQ* (68% of PCa), *NEIL3* (47%), *ERCC6* (42%), *BRCA2* (52.6%), *BRCA1* (52%), *XPC* (47%), *JAG1* (47%), *RPA1* (42%), *ATM* (42%), *POLE* (36%), and *LIG1* (26%) (Fig. 2a). We also identified mutations in the non-DNA repair genes *AR* (15.7% of PCa) and *TMPRSS2* (42% of PCa), which were included as controls. We then focused on whether a mutator phenotype was potentially present. In this analysis, *ATM* and *ATR* were found to be associated with higher mutational burden (i.e., 50 mutations/tumor; Supplementary data 2) in all samples except one.

In Caucasians, the most frequently mutated DNA repairome genes were *POLQ* (48.4%), *NEIL3* (45.4%), *POLB* (45.4%), *BRCA2* (42.4%), *EXO1* (39%), *ERCC6* (36%), *ATR* (30%), *RBBP8* (24%) *BRCA1*, *ATM*, *JAG1*, *XPC*, and *POLE* (24%) (Fig. 2b). The non-DNA repair genes *AR* and *TMPRSS2* were found to be mutated in 12% and 30% of PCa samples, respectively. Regarding the mutator phenotype, we identified *BRCA2* and *NEIL3* to be associated with higher mutational burden (i.e., 20 mutations/tumor) in tumors in Caucasians (Supplementary data 2).

### Clinical implications

**Fig. 3:**
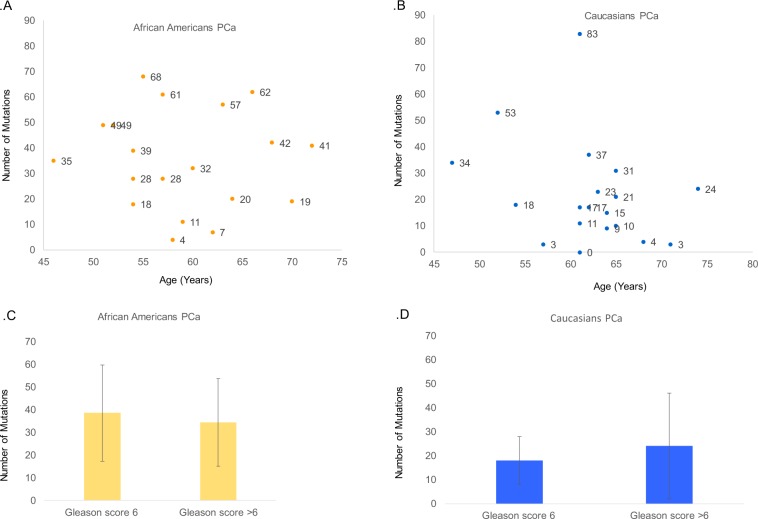
Analysis of Somatic mutations in the DNA repairome in African Americans (*n* = 19) and Caucasians (*n* = 33) PCa with age and Gleason score. **a** Mutation rate in different age groups in African American PCa. **b** Mutation rate in different age groups in Caucasian PCa. **c** Comparison of number of mutations in African American PCa with Gleason score 6 and Gleason score > 6. **d** Comparison of number of mutations in Caucasian PCa with Gleason score 6 and Gleason score > 6.

Next, we sought to identify whether age might influence the mutational rate in these two racial groups. In PCa tumors in African Americans, we found a marginal increase in the mutation rate for patients diagnosed at ages younger than 65 years (average 33 mutations) vs 65 years or older (average 41 mutations). We did not find a significant (*p* = 0.41) association between mutations in DNA-repair genes and age in both groups (Fig. 3a, b).

A slight increase in the mutation rate (18 vs 24 mutations, *p* = 0.55) was observed with higher Gleason scores only in Caucasians (Fig. 3d). Because of the limitation of samples with a Gleason score 6 in both races, we were unable to draw conclusions regarding low vs high Gleason scores. Gleason score 7 samples were further evaluated on the basis of 4 + 3 vs 3 + 4, but we did not find a significant difference with regards to clinical parameters (data not shown). We also did not find an association between PSA and the mutation rate in either racial group (data not shown).

### Recurrent somatic mutations in DNA repairome genes

Next, we focused on identifying recurrent mutations in top mutated DNA repair/response (DDR) genes. In PCa tumors in African Americans, *ERCC6* in the nuclear excision repair pathway (NER) showed recurrent mutation of M1097V (methionine-1097 to valine) (Table 2). The *XPC* gene in the NER pathway of DNA repair was not only frequently mutated but also contained two recurrent mutations (Table 2). In the base excision repair (BER) pathway, we identified three recurrent mutations in *NEIL3* (Table 2). The mismatch repair (MMR) pathway gene *EXO1* had three recurrent mutations: p.H354R, p.V458M, and p.E589K (Table 2). Among these, p.E589K (glutamic acid-586 to lysine) was the most prevalent missense mutation identified in PCa in African Americans. *ATR* was also a top mutated gene in tumors in African Americans, with two recurrent mutations (Table 2). *TMPRSS2*, a non-DNA repair gene, was also frequently mutated in tumors in African Americans and contained a recurrent missense mutation (valine-160 to methionine; Table 2).

**Table 2:** Distribution of recurrent somatic substitutions in prostate tumors in African American men (*n* = 19).

Variant	DNA Repair Pathways	Incidence	Frequency (%)
*ERCC6*	NER		
p.M1097V	–	4	21
p.G399*	–	5	26
p.L45L	–	4	21
*XPC*	–		
p.Q939K	–	7	36
p.A499V	–	4	21
*NEIL3*	BER		
p.P117R	–	3	15
p.R381R	–	4	21
p.Q471H	–	3	15
*EXO1*	MMR		
p.E589K	–	8	42
p.H354R	–	4	21
p.V458M	–	6	31
*ATR*	–		
p.M211T	–	4	21
p.G592G	–	4	21
*ATM*	–		
p.D1853N	–	4	21
*TMPRSS2*			
p.V160M		4	21
p.T75*		4	21
*AR*			
p.E213*		3	15%

Missense substitutions are indicated by the normal amino acid and the codon number followed by the mutant amino acid, in one letter code, and for protein p, frequencies are approximate.

The most recurrent missense mutations in PCa tumors in Caucasians were detected in the *POLB* gene in the BER pathway. The somatic mutation p.E216K (glutamic acid-216 to lysine) was present in 33% of the tumors (8/24; Table 3). In the *EXO1* gene in the MMR pathway, we identified two recurrent mutations (Table 3), including p.H354R (histidine-354 to arginine), which was also found in tumors in African Americans. *ERCC6* in the NER pathway also had the recurrent mutation p.M1097V (methionine-1097 to valine) in PCa tumors in Caucasians (Table 3) and was also found in those in African Americans.

**Table 3:** Distribution of recurrent somatic substitutions in prostate tumors in Caucasian men (*n* = 33).

Variant	DNA Repair Pathways	Incidence	Frequency (%)
*POLB*	BER		
p.E216K	–	8	24
*EXO1*	MMR		
p.H354R	–	3	1
p.E670G	–	6	24
*ERCC6*	NER		
p.M1097V	–	3	1
*RFC1*	NER		
p.P847P	–	3	1
*BRCA2*	FA		
p.N372H	–	4	1.2
*AR*			
p.E213*		2	6

Missense substitutions are indicated by the normal amino acid and the codon number followed by the mutant amino acid, in one letter code, and for protein p, frequencies are approximate.

### Somatic mutations in functional domains of DNA repairome genes

**Fig. 4:**
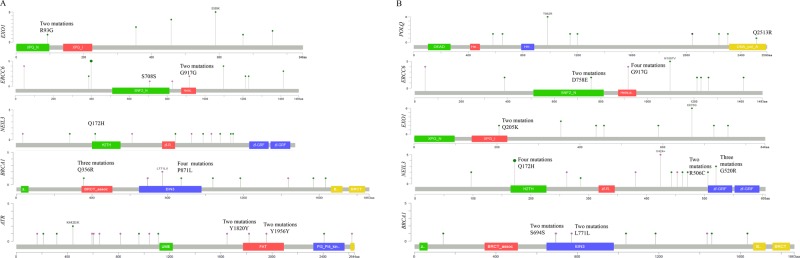
Representation of the protein-coding sequences and major functional domains in DNA repairome genes in PCa samples. **a** Location of mutations and protein domains encoded by the top mutated genes *ATR, BRCA1, NEIL3*, *ERCC6*, and *EXO1* in African American PCa samples. **b** Location of mutations and protein domains encoded by the op mutated genes *BRCA1, NEIL3, EXO1*, *ERCC6*, and *POLQ* in Caucasian PCa samples, determined using cBioPortal/mutation-mapper. Protein domains are distinguished by color. On the graph of each gene, the y axis represents the number of mutations identified and the x axis reflects the number of amino acid residues. Somatic mutations are shown in circles; missense mutations are shown in green-circle, silent mutations are shown in purple-circle, and truncating mutations are shown in black-circle. *Helic helicase*, *H2TH helix*-*2turn-helix domain, ZF-GRF* *zinc finger GRF domain, BRCT* *BRCA1*.

Our next aim was to analyze the distribution of these mutations at specific protein domains. Protein domains are particular sequences that have formed over evolution through duplication, recombination, or both. Domains often encode a structural entity associated with specific cellular functions. We systematically analyzed somatic mutations in the top five highly mutated DNA repair genes in both groups (tumors in African Americans and Caucasians) in terms of conserved protein domains. Here, we used mutation mapper, a publicly available visualization tool by cBioPortal (www.cbioportal.org) for cancer genomics data. We filtered out the top five mutated genes, *ATR, BRCA1, NEIL3, ERCC6*, and *EXO1*, in PCa tumors in African Americans and *NEIL3*, *BRCA1*, *EXO1*, *ERCC6*, and *POLQ* in PCa tumors in Caucasians. Our aim was to identify domains with mutations in all possible domains in the above-listed genes. In African Americans, in the *ATR* gene, the highest mutation burden was found in the FAT domain, where five somatic mutations were identified (Fig. 4a). We also identified one missense mutation in PI3K(s). In the *BRCA1* gene, we identified three missense mutations in the BRCT domain. In the helix-2turn-helix domain (H2TH) of *NEIL3*, a DNA-binding domain (DNA glycosylase/AP lyase enzymes), we found two missense mutations. For the *ERCC6* gene, we identified one silent mutation in the SNF2 domain and two silent mutations in the helicase conserved domain. For *EXO1*, we found two missense mutations in the XPG_N domain (Fig. 4a). In tumors in Caucasians, in *BRCA1*, we did not find mutations in the BRCT domain (in contrast to PCa tumors in African Americans). We identified four missense mutations in the H2TH domain and five missense mutations in the ZF-GRF domain of *NEIL3*. In *EXO1*, we identified two missense mutations in the XPG_I domain. In *ERCC6*, we found two missense mutations in the SNF2_N domain and four silent mutations in the helicase domain. We identified three missense mutations in the DNA_polA domain of *POLQ* (Fig. 4b).

### Mutations in the MMR, BER, and NER pathways

**Fig. 5:**
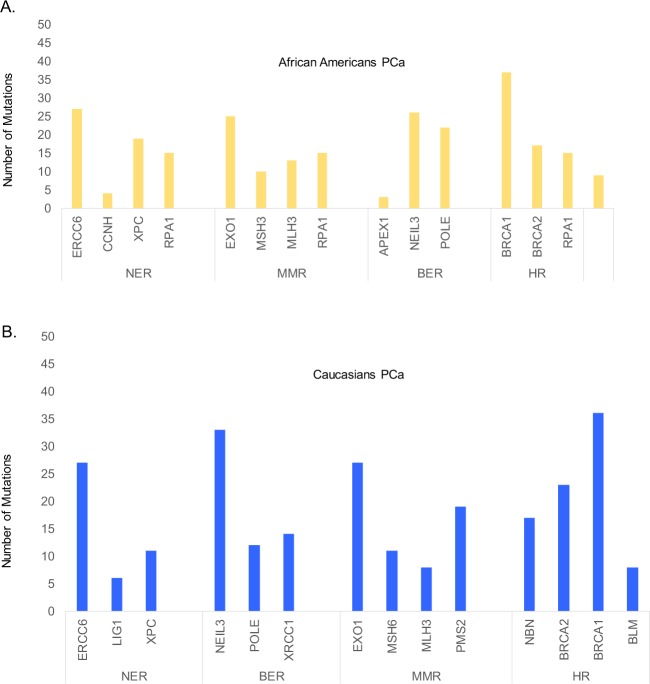
Frequency of mutations in the DNA nucleotide excision repair (NER), base excision repair (BER), homologus recombination (HR), and mismatch repair (MMR) pathways in PCa samples. **a** most frequently mutated genes across NER, MMR, BER, and HR pathways in African Americans (*n* = 19) and **b** most frequently mutated genes across NER, BER, MMR, and HR pathways in Caucasians (*n* = 33).

We also examined the distribution of mutations in genes in the NER, MMR, BER, and homologous recombination (HR) pathways in prostate tumors from African Americans and Caucasians. In African Americans, the most commonly mutated gene in the NER pathway was *ERCC6*, followed by *EXO1* in the MMR pathway, *NEIL3* in the BER pathway, and *BRCA1* and *BRCA2* in the HR pathway (Fig. 5a). In tumors in Caucasians, mutation frequencies within NER, MMR, and BER genes were approximately equal. Among tumors from Caucasians, the most mutations were found in the *NEIL3* gene in the BER pathway and *BRCA1* in the HR pathway. *ERCC6* in the NER pathway and *EXO1* in the MMR pathway were the second most mutated genes (Fig. 5b).

### Mutation spectra across the DNA repairome genes in PCa

**Fig. 6:**
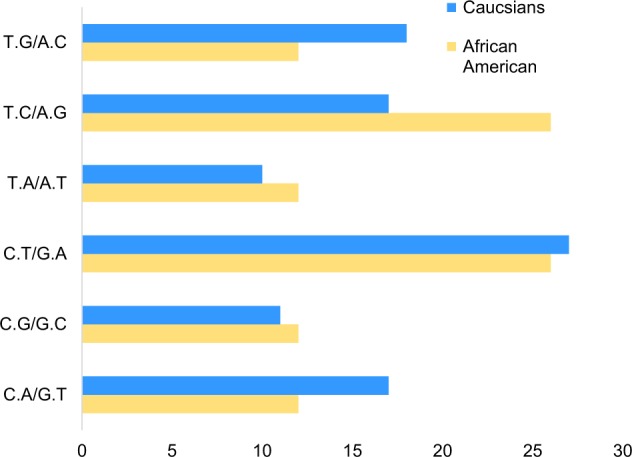
Somatic mutation spectra in DNA repairome genes: in PCa samples from African Americans (*n* = 19) and Caucasians (*n* = 33). Bars show the mutational context (%) of C.A>G.T, C.G>G.C, C.T>G.A., T.A>A.T, T.C>A.G, and T.G>A.C.

To further characterize the genetic instability, we sought to examine the T- to C-transitions in our data. We discovered markedly higher T- to C-transition frequencies (27%) in prostate tumors in African Americans than Caucasians (17.84%) (Fig. 6).

### Predicted effects of missense mutations

In our last objective, we attempted to identify substitutions affecting protein function through prediction methods by using sorting intolerant from tolerant (SIFT) [25] and Protein Variation Effect Analyzer (PROVEAN) [26]. SIFT predicts whether the amino acid substitution alters the protein function. Chromosome, start position, references, and variants were submitted to SIFT as input. The intolerance range of SIFT is ≤0.05, thus indicating that a variant is damaging/deleterious to the protein function, and a score > 0.05 predicts the tolerance range. In African Americans, out of 671 mutations, 278 were predicted to be deleterious by PROVEAN, and 217 were predicted to be damaging by SIFT (Supplementary data 3). In Caucasians, out of 762 mutations, 231 missense mutations were predicted to be deleterious by PROVEAN, and 207 missense mutations were predicted to be damaging by SIFT (Supplementary data 4). We did not analyze prediction in the context of protein stability that causes deleteriousness of nonsynonymous mutations; however, further studies in the area could be performed to elucidate this aspect.

## Discussion

The DDR genes (DNA “repairome”) protects genome stability. To address the importance of genomic instability in human prostate carcinogenesis, we deep sequenced the exons of 124 genes involved in the DNA damage repair/response in 52 PCas and matched normal tissue samples (peripheral blood lymphocytes), at very high resolution (312–768 average read depth). We found 1433 somatic mutations in these tumors (24–38 mutations per patient, depending on race and Gleason score). We further found that all prostate tumors had somatic mutations in DDR genes, regardless of the Gleason score. Somatic mutations included indels, stop-gain, LOH, and missense substitutions. Approximately 30–40% of these somatic mutations were predicted to be damaging in different types of software analysis and thus were unlikely to be “passenger” mutations. Control non-DNA repair genes were included in the analysis: *AR* was the 59th highest gene hit in African Americans, and *TMPRSS2* was the 14th highest gene hit in Caucasians. Thus, many DNA repair genes are somatically mutated more often than well characterized targets in prostate tumors. We also found that somatic mutations in *BRCA2*, *NEIL3*, *ATM*, and *ATR* were associated with higher mutational burden in prostate tumors. The higher rate of somatic mutations in the DNA repairome in this study compared with previous publications [8] may be the result of: (a) the much higher read depth that we used and (b) the exclusion of samples with less than 50% tumor tissue (upon biopsy) in our analysis (other studies often use a 10–20% tumor cutoff [24]. We conclude that a mutation “hit” in a DNA repair gene appears to be an obligatory event in PCa, a finding consistent with the mutator phenotype hypothesis.

Our data also show that, although DNA repair genes are often “hit” in PCa, only some of these mutations are prevalent (clonal), and in only some tumors (Supplementary data 1), during primary prostate tumor development. Furthermore, the DNA repairome somatic mutation frequency does not directly correlate with the higher Gleason score (>7). These data are somewhat but not fully consistent with the mutator phenotype hypothesis for prostate tumor progression [27]. An alternative hypothesis consistent with these data is that mutator mutations may become clonal very late in tumor evolution, after successive “bottlenecks,” such as chemo/immunotherapy, changes in the tumor microenvironment, or metastasis. Until that “final clonality” point, intratumor genetic heterogeneity may actually regulate tumor caused mortality, by keeping “aggressive” tumor cells (also sometimes called “cancer stem cells”) [28] from dominating. In this model, tumors may resemble genetic Darwinian evolution landscapes in microscale. The presence of various tumor genotypes may reduce the chances of organismal mortality until successive bottlenecks result in the selection of a highly lethal genotype.

Another major goal of this study was to examine the issue of health disparities in PCa: African Americans have the highest incidence of PCa in the world. We found that part of this difference between African Americans and Caucasians may be due to the high heterogeneity in the somatic mutations of the DNA repairome: although the top somatically mutated genes were similar in both racial groups, the exact order and frequency of specific mutations (biomarkers) differed (Fig. 4). For example, the most common somatic mutation in Caucasians, p.E216K in *POLB*, found in 43% of patients, was found in only three African Americans. p.E589K of *EXO1* and p.Q939K of *XPC* were identified in 42 and 36% of African American tumors only. Moreover, African Americans had more somatic mutations in DNA repair genes per tumor than Caucasians. This finding, together with the established higher incidence and mortality observed in African Americans, suggests that somatic mutations of the DNA repairome may be important in prostate tumor progression.

We identified mutations in all major DNA repair pathways in tumors in both African Americans and Caucasians. *NEIL3* and *BRCA1* were the top hits in Caucasians, and *ATR* was the top hit in African Americans. Some patients showed only LOH, whereas others showed only missense mutations. However, most patients had both LOH and missense mutations, often in different genes. The overall picture is very complex and heterogeneous. This finding is consistent with the analysis of whole genome studies in prostate tumors [29]. However, our study has certain limitations. Because very few samples had a Gleason score 6 or lower, or one higher than 8, we could not reach conclusions in the analysis of low vs high Gleason scores. Furthermore, very few patients were <40 years or >70 years old at diagnosis, and the incidence of DNA-repair gene mutations may differ between these age groups and the rest of the population. Lastly, we did not have information on the smoking habits of these patients, which might have affected the DNA repair process or mutations. In addition, ancestry of the samples was not determined. These limitations can be addressed is future studies.

A relatively recent method of targeting tumors is synthetic lethality. This approach relies on the sensitivity of tumors with, e.g., prevalent loss-of-function *BRCA1*/*BRCA2* mutations to *PARP1* or *POLQ* inhibitory drugs [30]. Indeed, owing to the inhibition of both the HR and NHEJ pathways, these tumor cells cannot repair double strand breaks. Recently, this approach has been found to be efficacious even for tumors displaying LOH at *BRCA1*/*BRCA2*, but not complete loss of function (presumably because the second hit remains unknown in these tumors) or mutations in genes such as *ATM*, *ATR*, *PALB2*, and *FANCA* [31, 32]. The synthetic lethality approach has also been extended to the use of other chemotherapeutic drugs like platinum [33]. We found that 20 (39%) patients with PCa had prevalent (i.e., >50% mutant) missense or frameshift somatic mutations at both (a) *BRCA1* or *BRCA2*, and (b) *POLQ*, *ATM*, *ATR*, or *PARP1*. Of these 20 patients, 15 had LOH at *BRCA1* or *BRCA2* (Supplementary data 1). Furthermore, at least two of these prevalent missense mutations in each patient (including at least one in each of the HR and NHEJ pathways) has been reported to affect protein function, survival, outcome, or response to therapy (data not shown). We conclude that these somatic mutations may be good biomarkers for selecting patients to be treated with a synthetic lethality approach. The fact that 20–25% of metastatic prostate tumors have defects in the DNA repairome, mostly in the HR pathway [33], suggests that synthetic lethality may be an attractive approach for the treatment of advanced and/ or metastatic PCa.

We identified several recurrent somatic mutations in the DNA repairome in prostate tumors. These mutations may serve as important biomarkers of tumor progression. The most common mutation in Caucasians was p.E216K in *POLB*, which was found in 14 tumors (43%), whereas in African Americans, p.E589K in *EXO1* was found in 8 tumors (42%). Both mutations have been reported in previous studies. The p.E216K mutation in *POLB* does not alter polymerase activity in vitro [15], but it has cellular effects in vivo (manuscript in preparation). The p.E589K mutation in *EXO1* has been reported to affect the response to cisplatin treatment in patients with head and neck tumors [34], and thus may also be functional. Furthermore, all tumors with a Gleason score >7 contained either the p.E216K *POLB* mutation or a prevalent missense mutation in *EXO1* (p.E589K, p.V458M, or p.H354R). One of these tumors also contained a prevalent missense mutation in *XPC*, p.Q939K, which has been reported to affect the response to cisplatin in patients with osteosarcoma [35]. The p.H354R mutation in *EXO1* has been shown to affect survival in pancreatic cancer [36]. Thus, all these somatic mutations may be important biomarkers of prostate tumor progression.

As the cost of genomic technologies (such as GWAS and NGS) drops and studies demonstrating the importance of genetic screening for disease outcome amount, genomic profiling of tumors will become part of standard cancer care. Traditional thinking dictates that germline alterations are more important for cancer prognosis, since they predate tumors, while somatic alterations are more important for therapy, since they are more likely to contribute to therapeutic resistance, metastasis, and mortality. However, more recent reports suggest that with regards to the DDR pathway, both germline and somatic mutations are of therapeutic importance. For example, 12% of metastatic PCa patients have germline alterations in HR DDR genes, with *BRCA1*, *BRCA2*, and *ATM*, the most commonly affected genes [37]. More specifically, germline *BRCA1*/*BRCA2* alterations have been associated with a higher Gleason score, metastases at diagnosis, rates of overall survival, and metastasis-free survival in PCa [37] as well as response to treatment [32]. Furthermore, the importance of germline variants in the DNA repairome is not confined to the prostate, but it has been shown in other tumors, such as colorectal and breast/ovarian [38]. Thus, future translational studies for the discovery of therapeutic biomarkers in the DNA repairome should interrogate both the germline and somatic variants.

In conclusion, through deep sequencing of highly pure tumor biopsies, we found that the DNA repairome was somatically mutated in all prostate tumors tested. Highly mutated genes included *EXO1*, *ERCC6*, *POLQ*, *NEIL3*, *BRCA1*, *BRCA2*, *ATM*, and *ATR*. We further observed that the well-known difference in PCa incidence and mortality between African Americans and Caucasians may have a genetic basis, specifically involving the DNA repairome. Indeed, tumors in African Americans, compared with Caucasians, had distinct mutations in the DNA repairome, a higher rate of somatic mutations overall, and major disparities in genes such as *XPC*, *ATR*, and *MBD4*. If validated, these data can be used to select important biomarkers of PCa progression, mortality and racial disparities, and to guide therapeutic options.

## Methods and materials

### Clinical data

PCa samples were obtained from Tulane University Hospital in New Orleans, LA, under ethical approval granted by Tulane University’s local research ethics committee. Prostate samples were obtained from patients who underwent radical prostatectomy for PCa. Each sample was microscopically verified for the presence of tumor by a pathologist and those samples containing more than 50% of tumor were selected for our study. Correlative clinical data were collected and entered into databases. DNA was extracted from 63 PCa tumors and 63 matched normal tissue samples (peripheral blood lymphocytes), and only samples found to be composed of >50% tumor cells, were included the study. Targeted exome sequencing was performed for all 63 tumor samples and 63 matched normal tissue samples; 11 samples were excluded because of low read coverage or failure to pass quality control. Specimens were grouped based on self-identified race.

### Microdissection

Specimens were formalin fixed, embedded in paraffin, sectioned, and transferred on microscopic slides where they were deparaffinized and stained with hematoxylin and eosin. Selected populations of carcinoma cells were microdissected and tumor DNA was then extracted from the microdissected cells using established methods [39].

### Genomic DNA (gDNA) extraction

DNA from normal tissue and peripheral blood lymphocytes was extracted with a DNeasy blood and tissue kit (Qiagen Inc, Valencia, CA), as described by the manufacturer. DNA from FFPE samples was extracted with the Qiagen/heating method. Then 18 0 μL ATL buffer was added to the tube, and samples were subjected to high-heat treatment at 90 °C for 20 min to melt the paraffin. After 20 min, the samples were incubated at RT for 3 min and quickly centrifuged. Then 20 μL of proteinase K was added, and samples were briefly vortexed and incubated at 56 °C for 16 h. The tubes were quickly centrifuged, and 200 μL of buffer AL and 200 μL of ethanol were added. The mixture was added to a DNeasy Mini spin column and centrifuged for 1 min at 8000 rpm. The following steps were performed according to the manufacturer’s protocol.

### SureSelect custom target enrichment library preparation

We constructed exon baits for 124 DNA damage repair/response genes (Supplementary data 1). The length of each oligonucleotide of the customized bait was 120 bp, with 5× tiling. The average number of baits per target was 630.07, and the total number of baits was 36,544. We obtained RNA probes for the baits from Agilent SureSelect with a target size of 0.429230 Mb (SureSelect ELID 0308271, Agilent, Santa Clara, CA). The gDNA (50–500 ng) was diluted with 1× low Tris-EDTA buffer (10 ng/μL) and sheared with a Bioruptor (Diagenode). To obtain a target size of 200–350 bp, seven 30 s on/off cycles were applied. Agilent’s SureSelect XT Target Enrichment protocol version 1.5 was followed for library preparation with the following modifications. In brief, sheared DNA samples were purified with Agencourt AMPure XP magnetic Beads. DNA ends were repaired with an End it DNA repair kit (Epicentre Madison, WI), and adenine bases were added to the ends of repaired fragments (Ligation kit, NEB Ipswich, MA). Samples were purified with Agencourt AMPure XP magnetic beads, and indexing-specific paired-end adapters were ligated. The ligated library was then amplified with the Herculase II Fusion DNA Polymerase system and the following program: 2 min at 98 °C, six cycles of 30 s at 98 °C, 30 s at 65 °C, and 1 min at 72 °C, and 10 min at 72 °C. The library was hybridized with no modifications, as described by the manufacturer (SureSelect Agilent, Santa Clara, CA). Subsequently, post selection was performed with Streptavidin T1, and final extension was used to add the index. For indexing, the Herculase II Fusion DNA Polymerase 2 system was used with the following program: 2 min at 98 °C, 12 cycles of 30 s at 98 °C, 30 s at 57 °C, and 1 min at 72 °C, and 10 min at 72 °C. The indexed library was purified and analyzed with a 2100 Bioanalyzer (Agilent, Santa Clara, CA).

### Massively parallel exome sequencing and alignment

Samples were sequenced at the John P. Hussman Institute for Human Genomics, University of Miami Miller School of Medicine (Miami, FL) and UW Biotechnology Center (Madison, WI). Flow cells were prepared, and sequencing clusters were generated according to Illumina library protocols. Sequencing was performed with 99 base paired-end sequencing on Hiseq 2000 or Hiseq 2500 genome analyzers, in accordance with the Illumina Genome Analyzer operating manual. The average sequence coverage was 712-fold for normal samples and 368-fold for tumor samples (Supplementary data 2).

### Downstream analysis of exome sequencing

Downstream analysis of samples was performed at the Tulane Cancer Center at the Crusaders Next Generation Sequence Analysis Core. Before downstream analysis, quality control checks on raw sequence data were run with FastQC. To generate the prerequisite input BAM file for SAMtools mpileup function, we aligned paired-end reads to the reference human genome (GRCh37) with STAR Aligner. The BAM files were then sorted and indexed with a reference sequence.

### Germline variant calling and somatic mutation detection by VarScan2

Variant calling was performed with the SAMtools mpileup function with the command samtools mpileup -f [reference sequence] [parameters] [BAM file(s)] >myData.mpileup. In this study, we present data for tumor samples and matched (normal) controls to distinguish acquired/somatic mutations (<0.01% of variants) from inherited germline variation (>99.99% of variants). As described by the developer [40], the VarScan somatic command accepts mpileup input from a normal and tumor sample (in that order); at every position meeting the minimum coverage requirement, it calls both samples independently to identify possible variants such as germline, LOH, or somatic variants. We also specified the threshold –somatic-p-value (Fisher’s exact test) of the read counts supporting reference and variant alleles in normal samples to determine the statistical significance between normal and tumor samples. The following two commands were used, as described by the developer [40]:

To run SAMtools mpileup on the BAM files for normal and tumor samples:

samtools mpileup –B –q 1 –f reference.fasta normal.bam tumor.bam >normal-tumor.mpileup

To detect somatic mutations, with VarScan run on the mpileup output file:

java –jar VarScan.jar somatic normal-tumor.mpileup output.basename –min-coverage 10 –min-var-freq 0.08 –somatic-p-value 0.05,

where (normal-tumor.mpileup) is the mpileup output file, and (output.basename) is the basename for the VarScan output files.

### Functional annotation of genetic variants by ANNOVAR

We used ANNOVAR (http://www.openbioinformatics.org/annovar/) to annotate single-nucleotide variants and insertions/deletions, to examine their functional consequences on genes, or to identify variants reported in dbSNP [41].

### Prediction of variant effects

We applied SIFT (http://sift-dna.org) and PROVEAN (http://provean.jcvi.org) to predict whether a given amino acid substitution affects protein function.

### Statistical test

The validation status was determined by comparing tumor/normal read counts for each allele with VarScan. For validation, we had more than 50 reads with base quality ≥15 (Phred score) for both normal and tumor samples. The somatic *p* value significance threshold was set as <0.05 and was calculated by VarScan with Fisher’s exact test. Mutation rates in DNA-repair gene mutations and age, race, or Gleason score were evaluated with Fisher’s exact test.

## Supplementary information

supplementary legends

Supplementary data 1

Supplementary data 2

Supplementary data 3

Supplementary data 4
