# Supplementary material for: Somatic mutations in the DNA repairome in prostate cancers in African Americans and Caucasians
Source: Oncogene. 2020 Apr 16;39(21):4299–311. doi: 10.1038/s41388-020-1280-x (PMC7239769; doi:10.1038/s41388-020-1280-x)
Supplement: Supplementary file 1 — supplementary legends [file 41388_2020_1280_MOESM1_ESM.docx]

**Supplementary legends**

**Supplementary data 1.** List of DNA repairome genes used in this study.

**Supplementary data 2.** Mutations reported in targeted exome sequencing in prostate tumors in African Americans and Caucasians.

**Supplementary data 3.** Predicted effects of mutations in DNA repairome genes in prostate tumors in African Americans.

**Supplementary data 4.** Predicted effects of mutations in DNA repairome genes in prostate tumors in Caucasians.
