# Supplementary material for: Somatic mutations in the DNA repairome in prostate cancers in African Americans and Caucasians
Source: Oncogene. 2020 Apr 16;39(21):4299–311. doi: 10.1038/s41388-020-1280-x (PMC7239769; doi:10.1038/s41388-020-1280-x)
Supplement: Supplementary file 2 — Supplementary data 1 [file 41388_2020_1280_MOESM2_ESM.docx]

**Supplementary data 1.**

|  |  |  |  |  |  |  |
| --- | --- | --- | --- | --- | --- | --- |
| TP73 |  |  |  |  |  |  |
| E2F2 |  |  |  |  |  |  |
| MUTYH |  |  |  |  |  |  |
| CDKN2C |  |  |  |  |  |  |
| JUN |  |  |  |  |  |  |
| GADD45A |  |  |  |  |  |  |
| PARP1 |  |  |  |  |  |  |
| EXO1 |  |  |  |  |  |  |
| MAPK8 |  |  |  |  |  |  |
| ERCC6 |  |  |  |  |  |  |
| CDC2 |  |  |  |  |  |  |
| EGR2 |  |  |  |  |  |  |
| DNTT |  |  |  |  |  |  |
| POLL |  |  |  |  |  |  |
| GTF2H1 |  |  |  |  |  |  |
| DDB2 |  |  |  |  |  |  |
| DDB1 |  |  |  |  |  |  |
| FEN1 |  |  |  |  |  |  |
| POLD4 |  |  |  |  |  |  |
| CCND1 |  |  |  |  |  |  |
| MRE11A |  |  |  |  |  |  |
| ATM |  |  |  |  |  |  |
| CHEK1 |  |  |  |  |  |  |
| CCND2 |  |  |  |  |  |  |
| CDKN1B |  |  |  |  |  |  |
| SMUG1 |  |  |  |  |  |  |
| CDK2 |  |  |  |  |  |  |
| CDK4 |  |  |  |  |  |  |
| MDM2 |  |  |  |  |  |  |
| TDG |  |  |  |  |  |  |
| UNG |  |  |  |  |  |  |
| RFC5 |  |  |  |  |  |  |
| GTF2H3 |  |  |  |  |  |  |
| POLE |  |  |  |  |  |  |
| BRCA2 |  |  |  |  |  |  |
| RFC3 |  |  |  |  |  |  |
| RB1 |  |  |  |  |  |  |
| ERCC5 |  |  |  |  |  |  |
| LIG4 |  |  |  |  |  |  |
| CUL4A |  |  |  |  |  |  |
| TFDP1 |  |  |  |  |  |  |
| APEX1 |  |  |  |  |  |  |
| NFKBIA |  |  |  |  |  |  |
| FOXA1 |  |  |  |  |  |  |
| MNAT1 |  |  |  |  |  |  |
| MLH3 |  |  |  |  |  |  |
| POLK |  |  |  |  |  |  |
| RAD51 |  |  |  |  |  |  |
| NEIL1 |  |  |  |  |  |  |
| BLM |  |  |  |  |  |  |
| MPG |  |  |  |  |  |  |
| NTHL1 |  |  |  |  |  |  |
| ERCC4 |  |  |  |  |  |  |
| E2F4 |  |  |  |  |  |  |
| RPA1 |  |  |  |  |  |  |
| TP53 |  |  |  |  |  |  |
| LIG3 |  |  |  |  |  |  |
| BRCA1 |  |  |  |  |  |  |
| RBBP8 |  |  |  |  |  |  |
| POLI |  |  |  |  |  |  |
| GADD45B |  |  |  |  |  |  |
| CDKN2D |  |  |  |  |  |  |
| RAD23A |  |  |  |  |  |  |
| CCNE1 |  |  |  |  |  |  |
| XRCC1 |  |  |  |  |  |  |
| ERCC2 |  |  |  |  |  |  |
| LIG1 |  |  |  |  |  |  |
| PNKP |  |  |  |  |  |  |
| POLD1 |  |  |  |  |  |  |
| MSH2 |  |  |  |  |  |  |
| MSH6 |  |  |  |  |  |  |
| EGR4 |  |  |  |  |  |  |
| ERCC3 |  |  |  |  |  |  |
| PMS1 |  |  |  |  |  |  |
| XRCC5 |  |  |  |  |  |  |
| NHEJ1 |  |  |  |  |  |  |
| PCNA |  |  |  |  |  |  |
| JAG1 |  |  |  |  |  |  |
| E2F1 |  |  |  |  |  |  |
| TMPRSS2 |  |  |  |  |  |  |
| CHEK2 |  |  |  |  |  |  |
| RBX1 |  |  |  |  |  |  |
| XRCC6 |  |  |  |  |  |  |
| OGG1 |  |  |  |  |  |  |
| XPC |  |  |  |  |  |  |
| MLH1 |  |  |  |  |  |  |
| CDC25A |  |  |  |  |  |  |
| ACOX2 |  |  |  |  |  |  |
| POLQ |  |  |  |  |  |  |
| MBD4 |  |  |  |  |  |  |
| ATR |  |  |  |  |  |  |
| RFC4 |  |  |  |  |  |  |
| RFC1 |  |  |  |  |  |  |
| NFKB1 |  |  |  |  |  |  |
| CCNA2 |  |  |  |  |  |  |
| NEIL3 |  |  |  |  |  |  |
| ERCC8 |  |  |  |  |  |  |
| CDK7 |  |  |  |  |  |  |
| MSH3 |  |  |  |  |  |  |
| CCNH |  |  |  |  |  |  |
| RAD50 |  |  |  |  |  |  |
| EGR1 |  |  |  |  |  |  |
| E2F3 |  |  |  |  |  |  |
| CDKN1A |  |  |  |  |  |  |
| CCND3 |  |  |  |  |  |  |
| POLH |  |  |  |  |  |  |
| GTF2H4 |  |  |  |  |  |  |
| PMS2 |  |  |  |  |  |  |
| POLM |  |  |  |  |  |  |
| POLD2 |  |  |  |  |  |  |
| RFC2 |  |  |  |  |  |  |
| CDK6 |  |  |  |  |  |  |
| SHFM1 |  |  |  |  |  |  |
| NEIL2 |  |  |  |  |  |  |
| EGR3 |  |  |  |  |  |  |
| POLB |  |  |  |  |  |  |
| E2F5 |  |  |  |  |  |  |
| NBN |  |  |  |  |  |  |
| CDKN2A |  |  |  |  |  |  |
| CDKN2B |  |  |  |  |  |  |
| GADD45G |  |  |  |  |  |  |
| XPA |  |  |  |  |  |  |
| RAD23B |  |  |  |  |  |  |
| ABL1 |  |  |  |  |  |  |
| Notch1 |  |  |  |  |  |  |
| APEX2 |  |  |  |  |  |  |
| AR |  |  |  |  |  |  |
| RPA4 |  |  |  |  |  |  |
