# Supplementary material for: Somatic mutations in the DNA repairome in prostate cancers in African Americans and Caucasians
Source: Oncogene. 2020 Apr 16;39(21):4299–311. doi: 10.1038/s41388-020-1280-x (PMC7239769; doi:10.1038/s41388-020-1280-x)
Supplement: Supplementary file 4 — Supplementary data 3 [file 41388_2020_1280_MOESM4_ESM.docx]

**Supplementary data 3.**

| INPUT | CODON_CHANGE | POS | RESIDUE_REF | RESIDUE_ALT | SCORE | PREDICTION (cutoff=-2.5) | dbSNP_ID |
| --- | --- | --- | --- | --- | --- | --- | --- |
| 3,142217537,A,G | TTT A[T/C]G ACT | 48 | M | T | -6 | Deleterious | rs2227932 |
| 14,75513828,G,A | ATG C[C/T]T AGT | 844 | P | L | -2.89 | Deleterious | rs175080 |
| 14,75513828,G,A | ATG C[C/T]T AGT | 844 | P | L | -2.95 | Deleterious | rs175080 |
| 17,41246481,T,C | AAG C[A/G]G AAA | 356 | Q | R | -3.33 | Deleterious | rs1799950 |
| 17,41246481,T,C | AAG C[A/G]G AAA | 60 | Q | R | -3.18 | Deleterious | rs1799950 |
| 17,41246481,T,C | AAG C[A/G]G AAA | 356 | Q | R | -3.33 | Deleterious | rs1799950 |
| 17,41246481,T,C | AAG C[A/G]G AAA | 356 | Q | R | -3.3 | Deleterious | rs1799950 |
| 17,41246481,T,C | AAG C[A/G]G AAA | 356 | Q | R | -3.34 | Deleterious | rs1799950 |
| 17,41246481,T,C | AAG C[A/G]G AAA | 309 | Q | R | -3.3 | Deleterious | rs1799950 |
| 17,41246481,T,C | AAG C[A/G]G AAA | 356 | Q | R | -3.34 | Deleterious | rs1799950 |
| 17,41246481,T,C | AAG C[A/G]G AAA | 60 | Q | R | -3.47 | Deleterious | rs1799950 |
| 17,41246481,T,C | AAG C[A/G]G AAA | 356 | Q | R | -3.46 | Deleterious | rs1799950 |
| 17,41246481,T,C | AAG C[A/G]G AAA | 356 | Q | R | -3.37 | Deleterious | rs1799950 |
| 17,41246481,T,C | AAG C[A/G]G AAA | 330 | Q | R | -3.37 | Deleterious | rs1799950 |
| 17,41246481,T,C | AAG C[A/G]G AAA | 222 | Q | R | -3.5 | Deleterious | rs1799950 |
| 17,41244435,T,C | AAA G[A/G]A GCC | 1038 | E | G | -5.59 | Deleterious | rs16941 |
| 17,41244435,T,C | AAA G[A/G]A GCC | 742 | E | G | -5.36 | Deleterious | rs16941 |
| 17,41244435,T,C | AAA G[A/G]A GCC | 1038 | E | G | -5.59 | Deleterious | rs16941 |
| 17,41244435,T,C | AAA G[A/G]A GCC | 1038 | E | G | -5.69 | Deleterious | rs16941 |
| 17,41244435,T,C | AAA G[A/G]A GCC | 1038 | E | G | -5.78 | Deleterious | rs16941 |
| 17,41244435,T,C | AAA G[A/G]A GCC | 991 | E | G | -5.65 | Deleterious | rs16941 |
| 17,41244435,T,C | AAA G[A/G]A GCC | 1038 | E | G | -5.78 | Deleterious | rs16941 |
| 1,242015709,A,G | AGA [A/G]GA GAA | 93 | R | G | -6.14 | Deleterious | rs4149865 |
| 1,242015709,A,G | AGA [A/G]GA GAA | 93 | R | G | -6.14 | Deleterious | rs4149865 |
| 1,242015709,A,G | AGA [A/G]GA GAA | 93 | R | G | -6.37 | Deleterious | rs4149865 |
| 1,242015709,A,G | AGA [A/G]GA GAA | 93 | R | G | -6.14 | Deleterious | rs4149865 |
| 1,242048674,C,T | AAA C[C/T]T CTA | 757 | P | L | -4.15 | Deleterious | rs9350 |
| 1,242048674,C,T | AAA C[C/T]T CTA | 757 | P | L | -4.15 | Deleterious | rs9350 |
| 1,242048674,C,T | AAA C[C/T]T CTA | 122 | P | L | -7.83 | Deleterious | rs9350 |
| 1,242048674,C,T | AAA C[C/T]T CTA | 757 | P | L | -4.05 | Deleterious | rs9350 |
| 1,23836455,C,T | GAC [G/A]AC TAC | 411 | D | N | -3.37 | Deleterious | |
| 1,23836455,C,T | GAC [G/A]AC TAC | 411 | D | N | -3.37 | Deleterious | |
| 1,45800156,C,T | AGC C[G/A]T GGG | 27 | R | H | -5 | Deleterious | rs3219484 |
| 1,242015709,A,G | AGA [A/G]GA GAA | 93 | R | G | -6.14 | Deleterious | rs4149865 |
| 1,242015709,A,G | AGA [A/G]GA GAA | 93 | R | G | -6.14 | Deleterious | rs4149865 |
| 1,242015709,A,G | AGA [A/G]GA GAA | 93 | R | G | -6.37 | Deleterious | rs4149865 |
| 1,242015709,A,G | AGA [A/G]GA GAA | 93 | R | G | -6.14 | Deleterious | rs4149865 |
| 1,242048674,C,T | AAA C[C/T]T CTA | 757 | P | L | -4.15 | Deleterious | rs9350 |
| 1,242048674,C,T | AAA C[C/T]T CTA | 757 | P | L | -4.15 | Deleterious | rs9350 |
| 1,242048674,C,T | AAA C[C/T]T CTA | 122 | P | L | -7.83 | Deleterious | rs9350 |
| 1,242048674,C,T | AAA C[C/T]T CTA | 757 | P | L | -4.05 | Deleterious | rs9350 |
| 1,242048674,C,T | AAA C[C/T]T CTA | 757 | P | L | -4.15 | Deleterious | rs9350 |
| 1,242048674,C,T | AAA C[C/T]T CTA | 757 | P | L | -4.15 | Deleterious | rs9350 |
| 1,242048674,C,T | AAA C[C/T]T CTA | 122 | P | L | -7.83 | Deleterious | rs9350 |
| 1,242048674,C,T | AAA C[C/T]T CTA | 757 | P | L | -4.05 | Deleterious | rs9350 |
| 10,103340056,G,A | GGC [C/T]GG TCC | 438 | R | W | -4 | Deleterious | rs3730477 |
| 10,103340056,G,A | GGC [C/T]GG TCC | 161 | R | W | -4.6 | Deleterious | rs3730477 |
| 10,103340056,G,A | GGC [C/T]GG TCC | 438 | R | W | -4.1 | Deleterious | rs3730477 |
| 10,103340056,G,A | GGC [C/T]GG TCC | 163 | R | W | -4.6 | Deleterious | rs3730477 |
| 10,103340056,G,A | GGC [C/T]GG TCC | 438 | R | W | -4 | Deleterious | rs3730477 |
| 10,103340056,G,A | GGC [C/T]GG TCC | 111 | R | W | -4.84 | Deleterious | rs3730477 |
| 10,103340056,G,A | GGC [C/T]GG TCC | 438 | R | W | -4 | Deleterious | rs3730477 |
| 10,103340056,G,A | GGC [C/T]GG TCC | 350 | R | W | -4.44 | Deleterious | rs3730477 |
| 10,103340056,G,A | GGC [C/T]GG TCC | 438 | R | W | -4.1 | Deleterious | rs3730477 |
| 10,103340056,G,A | GGC [C/T]GG TCC | 175 | R | W | -4.6 | Deleterious | rs3730477 |
| 10,103340056,G,A | GGC [C/T]GG TCC | 346 | R | W | -4.34 | Deleterious | rs3730477 |
| 10,103340056,G,A | GGC [C/T]GG TCC | 161 | R | W | -4.75 | Deleterious | rs3730477 |
| 10,64573772,G,A | TAT C[C/T]G TCC | 209 | P | L | -3.82 | Deleterious | |
| 10,64573772,G,A | TAT C[C/T]G TCC | 159 | P | L | -3.65 | Deleterious | |
| 10,64573772,G,A | TAT C[C/T]G TCC | 209 | P | L | -3.82 | Deleterious | |
| 10,64573772,G,A | TAT C[C/T]G TCC | 222 | P | L | -3.35 | Deleterious | |
| 10,103340056,G,A | GGC [C/T]GG TCC | 438 | R | W | -4 | Deleterious | rs3730477 |
| 10,103340056,G,A | GGC [C/T]GG TCC | 161 | R | W | -4.6 | Deleterious | rs3730477 |
| 10,103340056,G,A | GGC [C/T]GG TCC | 438 | R | W | -4.1 | Deleterious | rs3730477 |
| 10,103340056,G,A | GGC [C/T]GG TCC | 163 | R | W | -4.6 | Deleterious | rs3730477 |
| 10,103340056,G,A | GGC [C/T]GG TCC | 438 | R | W | -4 | Deleterious | rs3730477 |
| 10,103340056,G,A | GGC [C/T]GG TCC | 111 | R | W | -4.84 | Deleterious | rs3730477 |
| 10,103340056,G,A | GGC [C/T]GG TCC | 438 | R | W | -4 | Deleterious | rs3730477 |
| 10,103340056,G,A | GGC [C/T]GG TCC | 350 | R | W | -4.44 | Deleterious | rs3730477 |
| 10,103340056,G,A | GGC [C/T]GG TCC | 438 | R | W | -4.1 | Deleterious | rs3730477 |
| 10,103340056,G,A | GGC [C/T]GG TCC | 175 | R | W | -4.6 | Deleterious | rs3730477 |
| 10,103340056,G,A | GGC [C/T]GG TCC | 346 | R | W | -4.34 | Deleterious | rs3730477 |
| 10,103340056,G,A | GGC [C/T]GG TCC | 161 | R | W | -4.75 | Deleterious | rs3730477 |
| 10,103340056,G,A | GGC [C/T]GG TCC | 438 | R | W | -4 | Deleterious | rs3730477 |
| 10,103340056,G,A | GGC [C/T]GG TCC | 161 | R | W | -4.6 | Deleterious | rs3730477 |
| 10,103340056,G,A | GGC [C/T]GG TCC | 438 | R | W | -4.1 | Deleterious | rs3730477 |
| 10,103340056,G,A | GGC [C/T]GG TCC | 163 | R | W | -4.6 | Deleterious | rs3730477 |
| 10,103340056,G,A | GGC [C/T]GG TCC | 438 | R | W | -4 | Deleterious | rs3730477 |
| 10,103340056,G,A | GGC [C/T]GG TCC | 111 | R | W | -4.84 | Deleterious | rs3730477 |
| 10,103340056,G,A | GGC [C/T]GG TCC | 438 | R | W | -4 | Deleterious | rs3730477 |
| 10,103340056,G,A | GGC [C/T]GG TCC | 350 | R | W | -4.44 | Deleterious | rs3730477 |
| 10,103340056,G,A | GGC [C/T]GG TCC | 438 | R | W | -4.1 | Deleterious | rs3730477 |
| 10,103340056,G,A | GGC [C/T]GG TCC | 175 | R | W | -4.6 | Deleterious | rs3730477 |
| 10,103340056,G,A | GGC [C/T]GG TCC | 346 | R | W | -4.34 | Deleterious | rs3730477 |
| 10,103340056,G,A | GGC [C/T]GG TCC | 161 | R | W | -4.75 | Deleterious | rs3730477 |
| 10,64573772,G,A | TAT C[C/T]G TCC | 209 | P | L | -3.82 | Deleterious | |
| 10,64573772,G,A | TAT C[C/T]G TCC | 159 | P | L | -3.65 | Deleterious | |
| 10,64573772,G,A | TAT C[C/T]G TCC | 209 | P | L | -3.82 | Deleterious | |
| 10,64573772,G,A | TAT C[C/T]G TCC | 222 | P | L | -3.35 | Deleterious | |
| 10,50678369,T,C | TGC [A/G]GA GAC | 1213 | R | G | -3.21 | Deleterious | rs2228527 |
| 10,50678369,T,C | TGC [A/G]GA GAC | 590 | R | G | -3.31 | Deleterious | rs2228527 |
| 10,50678369,T,C | TGC [A/G]GA GAC | 583 | R | G | -3.21 | Deleterious | rs2228527 |
| 10,50678369,T,C | TGC [A/G]GA GAC | 1213 | R | G | -3.21 | Deleterious | rs2228527 |
| 10,50678369,T,C | TGC [A/G]GA GAC | 590 | R | G | -3.31 | Deleterious | rs2228527 |
| 10,50678369,T,C | TGC [A/G]GA GAC | 583 | R | G | -3.21 | Deleterious | rs2228527 |
| 11,108175462,G,A | CAA [G/A]AT ACA | 24 | D | N | -3.18 | Deleterious | rs1801516 |
| 11,108175462,G,A | CAA [G/A]AT ACA | 24 | D | N | -3.18 | Deleterious | rs1801516 |
| 11,108175462,G,A | CAA [G/A]AT ACA | 24 | D | N | -3.18 | Deleterious | rs1801516 |
| 11,108173677,T,C | TGG A[T/C]A AAG | 1806 | I | T | -3.99 | Deleterious | |
| 11,108173677,T,C | TGG A[T/C]A AAG | 1806 | I | T | -3.99 | Deleterious | |
| 11,108175462,G,A | CAA [G/A]AT ACA | 24 | D | N | -3.18 | Deleterious | rs1801516 |
| 11,108173677,T,C | TGG A[T/C]A AAG | 1806 | I | T | -3.99 | Deleterious | |
| 11,108173677,T,C | TGG A[T/C]A AAG | 1806 | I | T | -3.99 | Deleterious | |
| 12,104376693,G,A | CCC [G/A]GC AGC | 195 | G | S | -5.5 | Deleterious | rs4135113 |
| 12,104376693,G,A | CCC [G/A]GC AGC | 199 | G | S | -5.5 | Deleterious | rs4135113 |
| 12,104376693,G,A | CCC [G/A]GC AGC | 192 | G | S | -5.3 | Deleterious | rs4135113 |
| 12,104376693,G,A | CCC [G/A]GC AGC | 103 | G | S | -5.5 | Deleterious | rs4135113 |
| 12,104376693,G,A | CCC [G/A]GC AGC | 56 | G | S | -5.6 | Deleterious | rs4135113 |
| 12,133220526,T,C | TAC A[A/G]T CTC | 1396 | N | S | -3.34 | Deleterious | rs5744934 |
| 12,133220526,T,C | TAC A[A/G]T CTC | 1407 | N | S | -3.34 | Deleterious | rs5744934 |
| 12,133220526,T,C | TAC A[A/G]T CTC | 1369 | N | S | -3.34 | Deleterious | rs5744934 |
| 12,133237569,C,T | GAC [G/A]TG CTG | 796 | V | M | -2.52 | Deleterious | rs147692158 |
| 12,104376931,G,A | GAA G[G/A]A GGA | 207 | G | E | -7.6 | Deleterious | |
| 12,104376931,G,A | GAA G[G/A]A GGA | 211 | G | E | -7.6 | Deleterious | |
| 12,104376931,G,A | GAA G[G/A]A GGA | 204 | G | E | -7.33 | Deleterious | |
| 12,104376931,G,A | GAA G[G/A]A GGA | 68 | G | E | -7.73 | Deleterious | |
| 13,108863591,G,A | CAA A[C/T]T GTT | 9 | T | I | -2.7 | Deleterious | rs1805388 |
| 13,108863591,G,A | CAA A[C/T]T GTT | 9 | T | I | -2.7 | Deleterious | rs1805388 |
| 13,108863591,G,A | CAA A[C/T]T GTT | 9 | T | I | -2.7 | Deleterious | rs1805388 |
| 13,108863591,G,A | CAA A[C/T]T GTT | 9 | T | I | -2.7 | Deleterious | rs1805388 |
| 13,108863591,G,A | CAA A[C/T]T GTT | 9 | T | I | -2.7 | Deleterious | rs1805388 |
| 13,108863591,G,A | CAA A[C/T]T GTT | 9 | T | I | -2.7 | Deleterious | rs1805388 |
| 13,103520519,G,A | ACC [G/A]AA GGA | 864 | E | K | -2.57 | Deleterious | |
| 13,103520519,G,A | ACC [G/A]AA GGA | 97 | E | K | -3.11 | Deleterious | |
| 13,103520519,G,A | ACC [G/A]AA GGA | 696 | E | K | -2.57 | Deleterious | |
| 13,103520565,A,G | CTC A[A/G]T GAA | 879 | N | S | -2.62 | Deleterious | rs4150342 |
| 13,103520565,A,G | CTC A[A/G]T GAA | 711 | N | S | -2.52 | Deleterious | rs4150342 |
| 13,103520565,A,G | CTC A[A/G]T GAA | 1304 | N | S | -2.76 | Deleterious | rs4150342 |
| 13,108863591,G,A | CAA A[C/T]T GTT | 9 | T | I | -2.7 | Deleterious | rs1805388 |
| 13,108863591,G,A | CAA A[C/T]T GTT | 9 | T | I | -2.7 | Deleterious | rs1805388 |
| 13,108863591,G,A | CAA A[C/T]T GTT | 9 | T | I | -2.7 | Deleterious | rs1805388 |
| 14,20924167,G,C | GAT CA[G/C] AAA | 34 | Q | H | -4.63 | Deleterious | rs1048945 |
| 14,75513828,G,A | ATG C[C/T]T AGT | 844 | P | L | -2.89 | Deleterious | rs175080 |
| 14,75513828,G,A | ATG C[C/T]T AGT | 844 | P | L | -2.95 | Deleterious | rs175080 |
| 14,75513828,G,A | ATG C[C/T]T AGT | 844 | P | L | -2.66 | Deleterious | rs175080 |
| 14,75513828,G,A | ATG C[C/T]T AGT | 844 | P | L | -2.95 | Deleterious | rs175080 |
| 14,75513828,G,A | ATG C[C/T]T AGT | 844 | P | L | -2.89 | Deleterious | rs175080 |
| 14,75513828,G,A | ATG C[C/T]T AGT | 844 | P | L | -2.95 | Deleterious | rs175080 |
| 14,75513828,G,A | ATG C[C/T]T AGT | 844 | P | L | -2.66 | Deleterious | rs175080 |
| 14,75513828,G,A | ATG C[C/T]T AGT | 844 | P | L | -2.95 | Deleterious | rs175080 |
| 14,75513828,G,A | ATG C[C/T]T AGT | 844 | P | L | -2.89 | Deleterious | rs175080 |
| 14,75513828,G,A | ATG C[C/T]T AGT | 844 | P | L | -2.95 | Deleterious | rs175080 |
| 14,75513828,G,A | ATG C[C/T]T AGT | 844 | P | L | -2.66 | Deleterious | rs175080 |
| 14,75513828,G,A | ATG C[C/T]T AGT | 844 | P | L | -2.95 | Deleterious | rs175080 |
| 14,75513828,G,A | ATG C[C/T]T AGT | 844 | P | L | -2.89 | Deleterious | rs175080 |
| 14,75513828,G,A | ATG C[C/T]T AGT | 844 | P | L | -2.95 | Deleterious | rs175080 |
| 14,75513828,G,A | ATG C[C/T]T AGT | 844 | P | L | -2.66 | Deleterious | rs175080 |
| 14,75513828,G,A | ATG C[C/T]T AGT | 844 | P | L | -2.95 | Deleterious | rs175080 |
| 15,41001295,C,T | CAT A[C/T]G CTA | 139 | T | M | -4.88 | Deleterious | rs148345609 |
| 15,41001295,C,T | CAT A[C/T]G CTA | 140 | T | M | -4.66 | Deleterious | rs148345609 |
| 15,41001295,C,T | CAT A[C/T]G CTA | 139 | T | M | -4.58 | Deleterious | rs148345609 |
| 15,41001295,C,T | CAT A[C/T]G CTA | 139 | T | M | -4.94 | Deleterious | rs148345609 |
| 15,41001295,C,T | CAT A[C/T]G CTA | 139 | T | M | -4.83 | Deleterious | rs148345609 |
| 15,41001295,C,T | CAT A[C/T]G CTA | 140 | T | M | -4.66 | Deleterious | rs148345609 |
| 15,41001295,C,T | CAT A[C/T]G CTA | 139 | T | M | -4.88 | Deleterious | rs148345609 |
| 15,41001295,C,T | CAT A[C/T]G CTA | 140 | T | M | -4.66 | Deleterious | rs148345609 |
| 15,41001295,C,T | CAT A[C/T]G CTA | 139 | T | M | -4.58 | Deleterious | rs148345609 |
| 15,41001295,C,T | CAT A[C/T]G CTA | 139 | T | M | -4.94 | Deleterious | rs148345609 |
| 15,41001295,C,T | CAT A[C/T]G CTA | 139 | T | M | -4.83 | Deleterious | rs148345609 |
| 15,41001295,C,T | CAT A[C/T]G CTA | 140 | T | M | -4.66 | Deleterious | rs148345609 |
| 16,14029033,G,A | GAC C[G/A]A ACA | 415 | R | Q | -2.82 | Deleterious | rs1800067 |
| 16,14029033,G,A | GAC C[G/A]A ACA | 404 | R | Q | -2.82 | Deleterious | rs1800067 |
| 16,2090156,C,T | GCC [G/A]CC CTG | 265 | A | T | -2.66 | Deleterious | rs148474733 |
| 16,2090156,C,T | GCC [G/A]CC CTG | 189 | A | T | -2.59 | Deleterious | rs148474733 |
| 16,2090156,C,T | GCC [G/A]CC CTG | 147 | A | T | -2.59 | Deleterious | rs148474733 |
| 16,2090156,C,T | GCC [G/A]CC CTG | 265 | A | T | -2.66 | Deleterious | rs148474733 |
| 16,2090156,C,T | GCC [G/A]CC CTG | 189 | A | T | -2.59 | Deleterious | rs148474733 |
| 16,2090156,C,T | GCC [G/A]CC CTG | 147 | A | T | -2.59 | Deleterious | rs148474733 |
| 17,41246481,T,C | AAG C[A/G]G AAA | 356 | Q | R | -3.33 | Deleterious | rs1799950 |
| 17,41246481,T,C | AAG C[A/G]G AAA | 60 | Q | R | -3.18 | Deleterious | rs1799950 |
| 17,41246481,T,C | AAG C[A/G]G AAA | 356 | Q | R | -3.33 | Deleterious | rs1799950 |
| 17,41246481,T,C | AAG C[A/G]G AAA | 356 | Q | R | -3.3 | Deleterious | rs1799950 |
| 17,41246481,T,C | AAG C[A/G]G AAA | 356 | Q | R | -3.34 | Deleterious | rs1799950 |
| 17,41246481,T,C | AAG C[A/G]G AAA | 309 | Q | R | -3.3 | Deleterious | rs1799950 |
| 17,41246481,T,C | AAG C[A/G]G AAA | 356 | Q | R | -3.34 | Deleterious | rs1799950 |
| 17,41246481,T,C | AAG C[A/G]G AAA | 60 | Q | R | -3.47 | Deleterious | rs1799950 |
| 17,41246481,T,C | AAG C[A/G]G AAA | 356 | Q | R | -3.46 | Deleterious | rs1799950 |
| 17,41246481,T,C | AAG C[A/G]G AAA | 356 | Q | R | -3.37 | Deleterious | rs1799950 |
| 17,41246481,T,C | AAG C[A/G]G AAA | 330 | Q | R | -3.37 | Deleterious | rs1799950 |
| 17,41246481,T,C | AAG C[A/G]G AAA | 222 | Q | R | -3.5 | Deleterious | rs1799950 |
| 17,41244435,T,C | AAA G[A/G]A GCC | 1038 | E | G | -5.59 | Deleterious | rs16941 |
| 17,41244435,T,C | AAA G[A/G]A GCC | 742 | E | G | -5.36 | Deleterious | rs16941 |
| 17,41244435,T,C | AAA G[A/G]A GCC | 1038 | E | G | -5.59 | Deleterious | rs16941 |
| 17,41244435,T,C | AAA G[A/G]A GCC | 1038 | E | G | -5.69 | Deleterious | rs16941 |
| 17,41244435,T,C | AAA G[A/G]A GCC | 1038 | E | G | -5.78 | Deleterious | rs16941 |
| 17,41244435,T,C | AAA G[A/G]A GCC | 991 | E | G | -5.65 | Deleterious | rs16941 |
| 17,41244435,T,C | AAA G[A/G]A GCC | 1038 | E | G | -5.78 | Deleterious | rs16941 |
| 17,41246481,T,C | AAG C[A/G]G AAA | 356 | Q | R | -3.33 | Deleterious | rs1799950 |
| 17,41246481,T,C | AAG C[A/G]G AAA | 60 | Q | R | -3.18 | Deleterious | rs1799950 |
| 17,41246481,T,C | AAG C[A/G]G AAA | 356 | Q | R | -3.33 | Deleterious | rs1799950 |
| 17,41246481,T,C | AAG C[A/G]G AAA | 356 | Q | R | -3.3 | Deleterious | rs1799950 |
| 17,41246481,T,C | AAG C[A/G]G AAA | 356 | Q | R | -3.34 | Deleterious | rs1799950 |
| 17,41246481,T,C | AAG C[A/G]G AAA | 309 | Q | R | -3.3 | Deleterious | rs1799950 |
| 17,41246481,T,C | AAG C[A/G]G AAA | 356 | Q | R | -3.34 | Deleterious | rs1799950 |
| 17,41246481,T,C | AAG C[A/G]G AAA | 60 | Q | R | -3.47 | Deleterious | rs1799950 |
| 17,41246481,T,C | AAG C[A/G]G AAA | 356 | Q | R | -3.46 | Deleterious | rs1799950 |
| 17,41246481,T,C | AAG C[A/G]G AAA | 356 | Q | R | -3.37 | Deleterious | rs1799950 |
| 17,41246481,T,C | AAG C[A/G]G AAA | 330 | Q | R | -3.37 | Deleterious | rs1799950 |
| 17,41246481,T,C | AAG C[A/G]G AAA | 222 | Q | R | -3.5 | Deleterious | rs1799950 |
| 17,41244435,T,C | AAA G[A/G]A GCC | 1038 | E | G | -5.59 | Deleterious | rs16941 |
| 17,41244435,T,C | AAA G[A/G]A GCC | 742 | E | G | -5.36 | Deleterious | rs16941 |
| 17,41244435,T,C | AAA G[A/G]A GCC | 1038 | E | G | -5.59 | Deleterious | rs16941 |
| 17,41244435,T,C | AAA G[A/G]A GCC | 1038 | E | G | -5.69 | Deleterious | rs16941 |
| 17,41244435,T,C | AAA G[A/G]A GCC | 1038 | E | G | -5.78 | Deleterious | rs16941 |
| 17,41244435,T,C | AAA G[A/G]A GCC | 991 | E | G | -5.65 | Deleterious | rs16941 |
| 17,41244435,T,C | AAA G[A/G]A GCC | 1038 | E | G | -5.78 | Deleterious | rs16941 |
| 19,48626550,C,A | TCC C[G/T]G CGC | 677 | R | L | -3.36 | Deleterious | rs3731008 |
| 19,48626550,C,A | TCC C[G/T]G CGC | 708 | R | L | -3.42 | Deleterious | rs3731008 |
| 19,48626550,C,A | TCC C[G/T]G CGC | 609 | R | L | -3.36 | Deleterious | rs3731008 |
| 19,48626550,C,A | TCC C[G/T]G CGC | 646 | R | L | -3.36 | Deleterious | rs3731008 |
| 19,50912844,C,T | CTG G[C/T]G CTG | 693 | A | V | -3.96 | Deleterious | |
| 19,50912844,C,T | CTG G[C/T]G CTG | 692 | A | V | -3.96 | Deleterious | |
| 19,48624555,C,T | GGC [G/A]TG GGT | 753 | V | M | -2.72 | Deleterious | rs146309259 |
| 19,48624555,C,T | GGC [G/A]TG GGT | 784 | V | M | -2.62 | Deleterious | rs146309259 |
| 19,48624555,C,T | GGC [G/A]TG GGT | 685 | V | M | -2.72 | Deleterious | rs146309259 |
| 19,48624555,C,T | GGC [G/A]TG GGT | 722 | V | M | -2.72 | Deleterious | rs146309259 |
| 2,48010488,G,A | CCC G[G/A]G GCC | 39 | G | E | -6.49 | Deleterious | rs1042821 |
| 2,48010488,G,A | CCC G[G/A]G GCC | 39 | G | E | -6.49 | Deleterious | rs1042821 |
| 2,48010488,G,A | CCC G[G/A]G GCC | 39 | G | E | -6.49 | Deleterious | rs1042821 |
| 2,128016978,G,A | TTT T[C/T]G ACA | 704 | S | L | -3.26 | Deleterious | rs4150521 |
| 2,128016978,G,A | TTT T[C/T]G ACA | 640 | S | L | -3.16 | Deleterious | rs4150521 |
| 20,10622501,G,C | ATA C[C/G]A GAT | 871 | P | R | -5.74 | Deleterious | rs35761929 |
| 20,10622501,G,C | ATA C[C/G]A GAT | 712 | P | R | -5.44 | Deleterious | rs35761929 |
| 20,10621566,G,A | ATA [C/T]GG GAT | 1022 | R | W | -3.69 | Deleterious | |
| 20,10621566,G,A | ATA [C/T]GG GAT | 863 | R | W | -3.46 | Deleterious | |
| 20,5098172,C,T | CTT [G/A]GA AAT | 176 | G | R | -7.81 | Deleterious | |
| 20,5098172,C,T | CTT [G/A]GA AAT | 176 | G | R | -7.81 | Deleterious | |
| 20,5098181,C,T | AGT [G/A]GA GAA | 173 | G | R | -7.81 | Deleterious | |
| 20,5098181,C,T | AGT [G/A]GA GAA | 173 | G | R | -7.81 | Deleterious | |
| 20,5098172,C,T | CTT [G/A]GA AAT | 176 | G | R | -7.81 | Deleterious | |
| 20,5098172,C,T | CTT [G/A]GA AAT | 176 | G | R | -7.81 | Deleterious | |
| 20,5098181,C,T | AGT [G/A]GA GAA | 173 | G | R | -7.81 | Deleterious | |
| 20,5098181,C,T | AGT [G/A]GA GAA | 173 | G | R | -7.81 | Deleterious | |
| 20,10622501,G,C | ATA C[C/G]A GAT | 871 | P | R | -5.74 | Deleterious | rs35761929 |
| 20,10622501,G,C | ATA C[C/G]A GAT | 712 | P | R | -5.44 | Deleterious | rs35761929 |
| 20,10622501,G,C | ATA C[C/G]A GAT | 871 | P | R | -5.74 | Deleterious | rs35761929 |
| 20,10622501,G,C | ATA C[C/G]A GAT | 712 | P | R | -5.44 | Deleterious | rs35761929 |
| 3,121208390,T,A | ACT [A/T]AT GAT | 1130 | N | Y | -2.98 | Deleterious | rs77744524 |
| 3,121208390,T,A | ACT [A/T]AT GAT | 1266 | N | Y | -2.98 | Deleterious | rs77744524 |
| 3,121208390,T,A | ACT [A/T]AT GAT | 753 | N | Y | -2.78 | Deleterious | rs77744524 |
| 3,121207094,C,A | ATG [G/T]AT TCT | 1562 | D | Y | -3.51 | Deleterious | rs3218643 |
| 3,121207094,C,A | ATG [G/T]AT TCT | 1698 | D | Y | -3.51 | Deleterious | rs3218643 |
| 3,121207094,C,A | ATG [G/T]AT TCT | 1185 | D | Y | -3.05 | Deleterious | rs3218643 |
| 3,121256014,G,A | TCT [C/T]AC CGA | 225 | H | Y | -3.37 | Deleterious | rs73857907 |
| 3,121256014,G,A | TCT [C/T]AC CGA | 360 | H | Y | -3.37 | Deleterious | rs73857907 |
| 3,142217537,A,G | TTT A[T/C]G ACT | 48 | M | T | -6 | Deleterious | rs2227932 |
| 3,142178199,G,A | CTT [C/T]GC CAG | 2407 | R | C | -5.22 | Deleterious | |
| 3,142178199,G,A | CTT [C/T]GC CAG | 2343 | R | C | -5.22 | Deleterious | |
| 3,142178199,G,A | CTT [C/T]GC CAG | 254 | R | C | -5.67 | Deleterious | |
| 3,129152090,C,A | TCA G[G/T]C AAA | 471 | G | V | -7.04 | Deleterious | |
| 3,129152090,C,A | TCA G[G/T]C AAA | 153 | G | V | -7.15 | Deleterious | |
| 3,129152090,C,A | TCA G[G/T]C AAA | 465 | G | V | -7.04 | Deleterious | |
| 3,129152090,C,A | TCA G[G/T]C AAA | 471 | G | V | -7.04 | Deleterious | |
| 3,129152090,C,A | TCA G[G/T]C AAA | 471 | G | V | -7.04 | Deleterious | |
| 3,142217537,A,G | TTT A[T/C]G ACT | 48 | M | T | -6 | Deleterious | rs2227932 |
| 3,142178199,G,A | CTT [C/T]GC CAG | 2407 | R | C | -5.22 | Deleterious | |
| 3,142178199,G,A | CTT [C/T]GC CAG | 2343 | R | C | -5.22 | Deleterious | |
| 3,142178199,G,A | CTT [C/T]GC CAG | 254 | R | C | -5.67 | Deleterious | |
| 3,14187589,G,A | AGC T[C/T]T CAA | 892 | S | F | -2.62 | Deleterious | |
| 3,14187589,G,A | AGC T[C/T]T CAA | 855 | S | F | -2.62 | Deleterious | |
| 3,121228960,G,A | GGA G[C/T]G ATT | 581 | A | V | -3.1 | Deleterious | rs487848 |
| 3,121228960,G,A | GGA G[C/T]G ATT | 717 | A | V | -3.1 | Deleterious | rs487848 |
| 3,121228960,G,A | GGA G[C/T]G ATT | 204 | A | V | -2.8 | Deleterious | rs487848 |
| 3,121256014,G,A | TCT [C/T]AC CGA | 225 | H | Y | -3.37 | Deleterious | rs73857907 |
| 3,121256014,G,A | TCT [C/T]AC CGA | 360 | H | Y | -3.37 | Deleterious | rs73857907 |
| 4,178256913,C,G | TCT C[C/G]T GTT | 117 | P | R | -5.64 | Deleterious | rs7689099 |
| 4,178256913,C,G | TCT C[C/G]T GTT | 117 | P | R | -5.64 | Deleterious | rs7689099 |
| 4,178256913,C,G | TCT C[C/G]T GTT | 117 | P | R | -5.64 | Deleterious | rs7689099 |
| 5,86695274,A,G | GAA G[T/C]T GCT | 270 | V | A | -2.76 | Deleterious | rs2230641 |
| 5,86695274,A,G | GAA G[T/C]T GCT | 196 | V | A | -2.82 | Deleterious | rs2230641 |
| 5,86695274,A,G | GAA G[T/C]T GCT | 196 | V | A | -2.92 | Deleterious | rs2230641 |
| 7,92300822,C,T | CCC [G/A]AA GTC | 189 | E | K | -3.9 | Deleterious | |
| 7,92300822,C,T | CCC [G/A]AA GTC | 189 | E | K | -3.9 | Deleterious | |
| 9,133760808,C,A | ATC T[C/A]T AGG | 1044 | S | Y | -2.55 | Deleterious | |
| 9,133760808,C,A | ATC T[C/A]T AGG | 1063 | S | Y | -2.55 | Deleterious | |
| 11,108173749,T,C | N[T/C]G TGT | 1 | X | X | NA | NA | rs145812395 |
| 12,54577718,G,A | CCC [C/T]AG GCT | 3 | Q | * | NA | NA | rs2233919 |
| 12,54577718,G,A | CCC [C/T]AG GCT | 3 | Q | * | NA | NA | rs2233919 |
| 12,54577718,G,A | CCC [C/T]AG GCT | 3 | Q | * | NA | NA | rs2233919 |
| 12,54577718,G,A | CCC [C/T]AG GCT | 3 | Q | * | NA | NA | rs2233919 |
| 12,54577718,G,A | CCC [C/T]AG GCT | 3 | Q | * | NA | NA | rs2233919 |
| 12,54577718,G,A | CCC [C/T]AG GCT | 3 | Q | * | NA | NA | rs2233919 |
| 12,54577718,G,A | CCC [C/T]AG GCT | 3 | Q | * | NA | NA | rs2233919 |
| 12,54577718,G,A | CCC [C/T]AG GCT | 3 | Q | * | NA | NA | rs2233919 |
| 12,54577718,G,A | CCC [C/T]AG GCT | 3 | Q | * | NA | NA | rs2233919 |
| 12,54577718,G,A | CCC [C/T]AG GCT | 3 | Q | * | NA | NA | rs2233919 |
| 12,54577718,G,A | CCC [C/T]AG GCT | 3 | Q | * | NA | NA | rs2233919 |
| 12,54577718,G,A | CCC [C/T]AG GCT | 3 | Q | * | NA | NA | rs2233919 |
| 12,54577718,G,A | CCC [C/T]AG GCT | 3 | Q | * | NA | NA | rs2233919 |
| 12,54577718,G,A | CCC [C/T]AG GCT | 3 | Q | * | NA | NA | rs2233919 |
| 12,54577718,G,A | CCC [C/T]AG GCT | 3 | Q | * | NA | NA | rs2233919 |
| 14,20924167,G,C | NA[G/C] AAA | 1 | X | X | NA | NA | rs1048945 |
| 2,128044351,C,A | GCC [G/T]AG CGA | 424 | E | * | NA | NA |  |
| 2,128044351,C,A | GCC [G/T]AG CGA | 360 | E | * | NA | NA |  |
| 2,190708804,C,T | TCT [C/T]AG ATT | 57 | Q | * | NA | NA | rs121434628 |
| 2,190708804,C,T | TCT [C/T]AG ATT | 172 | Q | * | NA | NA | rs121434628 |
| 2,190708804,C,T | TCT [C/T]AG ATT | 57 | Q | * | NA | NA | rs121434628 |
| 2,190708804,C,T | TCT [C/T]AG ATT | 233 | Q | * | NA | NA | rs121434628 |
| 2,190708804,C,T | TCT [C/T]AG ATT | 57 | Q | * | NA | NA | rs121434628 |
| 2,190708804,C,T | TCT [C/T]AG ATT | 233 | Q | * | NA | NA | rs121434628 |
| 2,128044351,C,A | GCC [G/T]AG CGA | 424 | E | * | NA | NA |  |
| 2,128044351,C,A | GCC [G/T]AG CGA | 360 | E | * | NA | NA |  |
| 21,42866487,C,A | ATT [G/T]GA CCT | 12 | G | * | NA | NA |  |
| 21,42866487,C,A | ATT [G/T]GA CCT | 49 | G | * | NA | NA |  |
| 21,42866487,C,A | ATT [G/T]GA CCT | 12 | G | * | NA | NA |  |
| 21,42866487,C,A | ATT [G/T]GA CCT | 12 | G | * | NA | NA |  |
| 21,42866487,C,A | ATT [G/T]GA CCT | 12 | G | * | NA | NA |  |
| 21,42866487,C,A | ATT [G/T]GA CCT | 12 | G | * | NA | NA |  |
| 3,129152089,G,A | CGG [C/T]AA AAT | 36 | Q | * | NA | NA | rs140696 |
| 3,129152089,G,A | CGG [C/T]AA AAT | 36 | Q | * | NA | NA | rs140696 |
| 3,129152089,G,A | CGG [C/T]AA AAT | 36 | Q | * | NA | NA | rs140696 |
| 3,129152089,G,A | CGG [C/T]AA AAT | 36 | Q | * | NA | NA | rs140696 |
| 3,129152089,G,A | CGG [C/T]AA AAT | 36 | Q | * | NA | NA | rs140696 |
| 3,129152089,G,A | CGG [C/T]AA AAT | 36 | Q | * | NA | NA | rs140696 |
| 1,23847464,C,A | GAC CA[G/T] CTC | 226 | Q | H | -1.61 | Neutral | rs2075995 |
| 1,242042301,G,A | TTT [G/A]AG AGC | 589 | E | K | -0.18 | Neutral | rs1047840 |
| 1,242042301,G,A | TTT [G/A]AG AGC | 589 | E | K | -0.18 | Neutral | rs1047840 |
| 1,242042301,G,A | TTT [G/A]AG AGC | 589 | E | K | -0.12 | Neutral | rs1047840 |
| 3,58512237,A,G | TCC TG[T/C] ACC | 434 | C | C | 0 | Neutral | rs1127745 |
| 3,58512237,A,G | TCC TG[T/C] ACC | 420 | C | C | 0 | Neutral | rs1127745 |
| 3,142226860,G,C | CGC TC[C/G] AAA | 1648 | S | S | 0 | Neutral |  |
| 3,142226860,G,C | CGC TC[C/G] AAA | 1584 | S | S | 0 | Neutral |  |
| 3,142168331,C,T | ATA CA[G/A] GAA | 2625 | Q | Q | 0 | Neutral | rs1802904 |
| 3,142168331,C,T | ATA CA[G/A] GAA | 2561 | Q | Q | 0 | Neutral | rs1802904 |
| 3,37053568,A,G | TCC [A/G]TC TTT | 219 | I | V | -0.46 | Neutral | rs1799977 |
| 3,37053568,A,G | TCC [A/G]TC TTT | 83 | I | V | -0.36 | Neutral | rs1799977 |
| 3,37053568,A,G | TCC [A/G]TC TTT | 13 | I | V | -0.3 | Neutral | rs1799977 |
| 3,37053568,A,G | TCC [A/G]TC TTT | 121 | I | V | -0.43 | Neutral | rs1799977 |
| 3,37053568,A,G | TCC [A/G]TC TTT | 185 | I | V | -0.4 | Neutral | rs1799977 |
| 3,37053568,A,G | TCC [A/G]TC TTT | 211 | I | V | -0.39 | Neutral | rs1799977 |
| 3,37053568,A,G | TCC [A/G]TC TTT | 185 | I | V | -0.46 | Neutral | rs1799977 |
| 3,121207143,G,T | ACC CA[C/A] CAG | 1545 | H | Q | 0.66 | Neutral | rs3218652 |
| 3,121207143,G,T | ACC CA[C/A] CAG | 1681 | H | Q | 0.66 | Neutral | rs3218652 |
| 3,121207143,G,T | ACC CA[C/A] CAG | 1168 | H | Q | 0.6 | Neutral | rs3218652 |
| 3,121151784,G,A | GTG G[C/T]A GAA | 2547 | A | V | -2.42 | Neutral | rs2306211 |
| 3,121151784,G,A | GTG G[C/T]A GAA | 2683 | A | V | -2.42 | Neutral | rs2306211 |
| 3,121151784,G,A | GTG G[C/T]A GAA | 2170 | A | V | -2.42 | Neutral | rs2306211 |
| 3,14187449,G,T | GAG [C/A]AG CTG | 939 | Q | K | 1.67 | Neutral | rs2228001 |
| 3,14187449,G,T | GAG [C/A]AG CTG | 902 | Q | K | 1.67 | Neutral | rs2228001 |
| 3,14199887,G,A | CCA G[C/T]G GCA | 499 | A | V | -0.96 | Neutral | rs2228000 |
| 3,14199887,G,A | CCA G[C/T]G GCA | 462 | A | V | -0.96 | Neutral | rs2228000 |
| 3,58512285,T,C | TAC TC[A/G] AAG | 418 | S | S | 0 | Neutral | rs13097249 |
| 3,58512285,T,C | TAC TC[A/G] AAG | 404 | S | S | 0 | Neutral | rs13097249 |
| 3,142217537,A,G | TTT TA[T/C] GAC | 1820 | Y | Y | 0 | Neutral | rs2227932 |
| 3,142217537,A,G | TTT TA[T/C] GAC | 1756 | Y | Y | 0 | Neutral | rs2227932 |
| 4,122743793,G,T | GCA CC[C/A] CTT | 74 | P | P | 0 | Neutral | rs3217760 |
| 5,68531253,C,T | AAG AA[C/T] ACC | 33 | N | N | 0 | Neutral | rs2972388 |
| 5,68531253,C,T | AAG AA[C/T] ACC | 33 | N | N | 0 | Neutral | rs2972388 |
| 5,68531253,C,T | AAG AA[C/T] ACC | 33 | N | N | 0 | Neutral | rs2972388 |
| 5,68531253,C,T | AAG AA[C/T] ACC | 33 | N | N | 0 | Neutral | rs2972388 |
| 5,68531253,C,T | AAG AA[C/T] ACC | 33 | N | N | 0 | Neutral | rs2972388 |
| 5,68531253,C,T | AAG AA[C/T] ACC | 33 | N | N | 0 | Neutral | rs2972388 |
| 5,68531253,C,T | AAG AA[C/T] ACC | 33 | N | N | 0 | Neutral | rs2972388 |
| 5,68531253,C,T | AAG AA[C/T] ACC | 33 | N | N | 0 | Neutral | rs2972388 |
| 5,131977963,T,C | GAA TA[T/C] GTG | 1282 | Y | Y | 0 | Neutral | rs1804670 |
| 5,131977963,T,C | GAA TA[T/C] GTG | 1143 | Y | Y | 0 | Neutral | rs1804670 |
| 5,68531253,C,T | AAG AA[C/T] ACC | 33 | N | N | 0 | Neutral | rs2972388 |
| 5,68531253,C,T | AAG AA[C/T] ACC | 33 | N | N | 0 | Neutral | rs2972388 |
| 5,68531253,C,T | AAG AA[C/T] ACC | 33 | N | N | 0 | Neutral | rs2972388 |
| 5,68531253,C,T | AAG AA[C/T] ACC | 33 | N | N | 0 | Neutral | rs2972388 |
| 5,68531253,C,T | AAG AA[C/T] ACC | 33 | N | N | 0 | Neutral | rs2972388 |
| 5,68531253,C,T | AAG AA[C/T] ACC | 33 | N | N | 0 | Neutral | rs2972388 |
| 5,68531253,C,T | AAG AA[C/T] ACC | 33 | N | N | 0 | Neutral | rs2972388 |
| 5,68531253,C,T | AAG AA[C/T] ACC | 33 | N | N | 0 | Neutral | rs2972388 |
| 6,30877760,A,C | CAC AC[A/C] CAG | 98 | T | T | 0 | Neutral | rs114336365 |
| 6,30877760,A,C | CAC AC[A/C] CAG | 98 | T | T | 0 | Neutral | rs114336365 |
| 6,30877760,A,C | CAC AC[A/C] CAG | 98 | T | T | 0 | Neutral | rs114336365 |
| 6,30877760,A,C | CAC AC[A/C] CAG | 42 | T | T | 0 | Neutral | rs114336365 |
| 6,36651971,C,A | CTG AG[C/A] CGC | 31 | S | R | 0.79 | Neutral | rs1801270 |
| 6,36651971,C,A | CTG AG[C/A] CGC | 31 | S | R | 0.79 | Neutral | rs1801270 |
| 6,36651971,C,A | CTG AG[C/A] CGC | 31 | S | R | 0.79 | Neutral | rs1801270 |
| 6,36651971,C,A | CTG AG[C/A] CGC | 65 | S | R | 0.82 | Neutral | rs1801270 |
| 6,30877760,A,C | CAC AC[A/C] CAG | 98 | T | T | 0 | Neutral | rs114336365 |
| 6,30877760,A,C | CAC AC[A/C] CAG | 98 | T | T | 0 | Neutral | rs114336365 |
| 6,30877760,A,C | CAC AC[A/C] CAG | 98 | T | T | 0 | Neutral | rs114336365 |
| 6,30877760,A,C | CAC AC[A/C] CAG | 42 | T | T | 0 | Neutral | rs114336365 |
| 8,90990479,C,G | GTT [G/C]AG TCC | 185 | E | Q | 0.53 | Neutral | rs1805794 |
| 8,90990479,C,G | GTT [G/C]AG TCC | 103 | E | Q | 0.56 | Neutral | rs1805794 |
| 8,90990479,C,G | GTT [G/C]AG TCC | 103 | E | Q | 0.69 | Neutral | rs1805794 |
| 8,90990479,C,G | GTT [G/C]AG TCC | 185 | E | Q | 0.63 | Neutral | rs1805794 |
| 8,90995019,C,T | ATT CT[G/A] ATT | 34 | L | L | 0 | Neutral | rs1063045 |
| 8,90995019,C,T | ATT CT[G/A] ATT | 34 | L | L | 0 | Neutral | rs1063045 |
| 8,90995019,C,T | ATT CT[G/A] ATT | 34 | L | L | 0 | Neutral | rs1063045 |
| 8,90995019,C,T | ATT CT[G/A] ATT | 34 | L | L | 0 | Neutral | rs1063045 |
| 8,90995019,C,T | ATT CT[G/A] ATT | 34 | L | L | 0 | Neutral | rs1063045 |
| 8,90967711,A,G | CAA GA[T/C] GCA | 399 | D | D | 0 | Neutral | rs709816 |
| 8,90967711,A,G | CAA GA[T/C] GCA | 317 | D | D | 0 | Neutral | rs709816 |
| 8,90967711,A,G | CAA GA[T/C] GCA | 399 | D | D | 0 | Neutral | rs709816 |
| 9,133756050,C,T | GAG AG[C/T] GAT | 559 | S | S | 0 | Neutral | rs35381228 |
| 9,133756050,C,T | GAG AG[C/T] GAT | 578 | S | S | 0 | Neutral | rs35381228 |
| 9,133756050,C,T | GAG AG[C/T] GAT | 374 | S | S | 0 | Neutral | rs35381228 |
| 10,50732280,C,T | GAT G[G/A]T ACT | 399 | G | D | 1.23 | Neutral | rs2228528 |
| 10,50732280,C,T | GAT G[G/A]T ACT | 399 | G | D | 0.57 | Neutral | rs2228528 |
| 10,50732280,C,T | GAT G[G/A]T ACT | 399 | G | D | 0.57 | Neutral | rs2228528 |
| 10,50678317,C,G | AGG C[G/C]T TAC | 1230 | R | P | -2.29 | Neutral | rs4253211 |
| 10,50678317,C,G | AGG C[G/C]T TAC | 607 | R | P | -2.42 | Neutral | rs4253211 |
| 10,50678317,C,G | AGG C[G/C]T TAC | 600 | R | P | -2.42 | Neutral | rs4253211 |
| 11,108114840,T,C | CAG TG[T/C] GCG | 219 | C | C | 0 | Neutral | rs2235003 |
| 11,108114840,T,C | CAG TG[T/C] GCG | 219 | C | C | 0 | Neutral | rs2235003 |
| 11,108114840,T,C | CAG TG[T/C] GCG | 219 | C | C | 0 | Neutral | rs2235003 |
| 11,108106443,T,A | ATG GA[T/A] ACA | 126 | D | E | -0.28 | Neutral | rs2234997 |
| 11,108106443,T,A | ATG GA[T/A] ACA | 126 | D | E | -0.28 | Neutral | rs2234997 |
| 11,108106443,T,A | ATG GA[T/A] ACA | 126 | D | E | -0.17 | Neutral | rs2234997 |
| 11,18369240,C,T | GTC [C/T]TG GCA | 315 | L | L | 0 | Neutral | rs145372677 |
| 11,18369240,C,T | GTC [C/T]TG GCA | 315 | L | L | 0 | Neutral | rs145372677 |
| 11,18369240,C,T | GTC [C/T]TG GCA | 3 | L | L | 0 | Neutral | rs145372677 |
| 11,18369240,C,T | GTC [C/T]TG GCA | 199 | L | L | 0 | Neutral | rs145372677 |
| 11,18369240,C,T | GTC [C/T]TG GCA | 111 | L | L | 0 | Neutral | rs145372677 |
| 12,12871099,T,G | GAT G[T/G]C AGC | 109 | V | G | -1.35 | Neutral | rs2066827 |
| 12,12871099,T,G | GAT G[T/G]C AGC | 109 | V | G | -2.06 | Neutral | rs2066827 |
| 12,12871099,T,G | GAT G[T/G]C AGC | 58 | V | G | -1.44 | Neutral | rs2066827 |
| 12,133219831,T,C | AGG GC[A/G] TCC | 1510 | A | A | 0 | Neutral | rs5744944 |
| 12,133219831,T,C | AGG GC[A/G] TCC | 1521 | A | A | 0 | Neutral | rs5744944 |
| 12,133219831,T,C | AGG GC[A/G] TCC | 1483 | A | A | 0 | Neutral | rs5744944 |
| 12,124144395,A,G | CCC CC[A/G] GTT | 205 | P | P | 0 | Neutral | rs1051793 |
| 12,124144395,A,G | CCC CC[A/G] GTT | 132 | P | P | 0 | Neutral | rs1051793 |
| 12,124144395,A,G | CCC [A/G]GT TCA | 198 | S | G | -0.87 | Neutral | rs1051793 |
| 12,124144395,A,G | CCC CC[A/G] GTT | 196 | P | P | 0 | Neutral | rs1051793 |
| 12,124144395,A,G | CCC CC[A/G] GTT | 203 | P | P | 0 | Neutral | rs1051793 |
| 12,124144395,A,G | CCC CC[A/G] GTT | 246 | P | P | 0 | Neutral | rs1051793 |
| 13,32929232,A,G | AAA TC[A/G] CAT | 2414 | S | S | 0 | Neutral | rs1799955 |
| 13,32929232,A,G | AAA TC[A/G] CAT | 2414 | S | S | 0 | Neutral | rs1799955 |
| 13,32911888,A,G | AGA AA[A/G] CCA | 1132 | K | K | 0 | Neutral | rs1801406 |
| 13,32911888,A,G | AGA AA[A/G] CCA | 1132 | K | K | 0 | Neutral | rs1801406 |
| 14,38061742,C,T | GTA [G/A]CC GGC | 83 | A | T | -0.23 | Neutral | rs7144658 |
| 14,38061742,C,T | GTA [G/A]CC GGC | 50 | A | T | -0.3 | Neutral | rs7144658 |
| 14,75483812,T,C | CAG CA[A/G] TCC | 1421 | Q | Q | 0 | Neutral | rs13712 |
| 14,75483812,T,C | CAG CA[A/G] TCC | 1445 | Q | Q | 0 | Neutral | rs13712 |
| 14,75483812,T,C | CAG CA[A/G] TCC | 383 | Q | Q | 0 | Neutral | rs13712 |
| 14,75483812,T,C | CAG CA[A/G] TCC | 116 | Q | Q | 0 | Neutral | rs13712 |
| 14,75483812,T,C | CAG CA[A/G] TCC | 469 | Q | Q | 0 | Neutral | rs13712 |
| 14,75483812,T,C | CAG CA[A/G] TCC | 1267 | Q | Q | 0 | Neutral | rs13712 |
| 14,75483812,T,C | CAG CA[A/G] TCC | 1445 | Q | Q | 0 | Neutral | rs13712 |
| 15,91354521,G,A | CCC [G/A]TA TCT | 1321 | V | I | -0.43 | Neutral | rs7167216 |
| 15,91354521,G,A | CCC [G/A]TA TCT | 508 | V | I | -0.45 | Neutral | rs7167216 |
| 15,91354521,G,A | CCC [G/A]TA TCT | 951 | V | I | -0.43 | Neutral | rs7167216 |
| 15,91354521,G,A | CCC [G/A]TA TCT | 1190 | V | I | -0.2 | Neutral | rs7167216 |
| 16,14031695,A,G | AAG GA[A/G] GCT | 628 | E | E | 0 | Neutral | rs2020958 |
| 16,14031695,A,G | AAG GA[A/G] GCT | 616 | E | E | 0 | Neutral | rs2020958 |
| 17,1782957,C,T | ACA TC[C/T] GGG | 352 | S | S | 0 | Neutral | rs2230930 |
| 17,1733399,A,G | GGC CA[A/G] CTG | 4 | Q | Q | 0 | Neutral | rs5030749 |
| 17,1782952,A,G | GAC [A/G]CA TCC | 351 | T | A | -0.41 | Neutral | rs5030755 |
| 17,41245466,G,A | GAC AG[C/T] GAT | 694 | S | S | 0 | Neutral | rs1799949 |
| 17,41245466,G,A | GAC AG[C/T] GAT | 398 | S | S | 0 | Neutral | rs1799949 |
| 17,41245466,G,A | GAC AG[C/T] GAT | 694 | S | S | 0 | Neutral | rs1799949 |
| 17,41245466,G,A | GAC AG[C/T] GAT | 694 | S | S | 0 | Neutral | rs1799949 |
| 17,41245466,G,A | GAC AG[C/T] GAT | 694 | S | S | 0 | Neutral | rs1799949 |
| 17,41245466,G,A | GAC AG[C/T] GAT | 647 | S | S | 0 | Neutral | rs1799949 |
| 17,41245466,G,A | GAC AG[C/T] GAT | 694 | S | S | 0 | Neutral | rs1799949 |
| 17,41234470,A,G | GAC TC[T/C] TCT | 1436 | S | S | 0 | Neutral | rs1060915 |
| 17,41234470,A,G | GAC TC[T/C] TCT | 1140 | S | S | 0 | Neutral | rs1060915 |
| 17,41234470,A,G | GAC TC[T/C] TCT | 294 | S | S | 0 | Neutral | rs1060915 |
| 17,41234470,A,G | GAC TC[T/C] TCT | 1436 | S | S | 0 | Neutral | rs1060915 |
| 17,41234470,A,G | GAC TC[T/C] TCT | 253 | S | S | 0 | Neutral | rs1060915 |
| 17,41234470,A,G | GAC TC[T/C] TCT | 1436 | S | S | 0 | Neutral | rs1060915 |
| 17,41234470,A,G | GAC TC[T/C] TCT | 286 | S | S | 0 | Neutral | rs1060915 |
| 17,41234470,A,G | GAC TC[T/C] TCT | 1436 | S | S | 0 | Neutral | rs1060915 |
| 17,41234470,A,G | GAC TC[T/C] TCT | 333 | S | S | 0 | Neutral | rs1060915 |
| 17,41234470,A,G | GAC TC[T/C] TCT | 201 | S | S | 0 | Neutral | rs1060915 |
| 17,41234470,A,G | GAC TC[T/C] TCT | 208 | S | S | 0 | Neutral | rs1060915 |
| 17,41234470,A,G | GAC TC[T/C] TCT | 1389 | S | S | 0 | Neutral | rs1060915 |
| 17,41234470,A,G | GAC TC[T/C] TCT | 286 | S | S | 0 | Neutral | rs1060915 |
| 17,41234470,A,G | GAC TC[T/C] TCT | 1436 | S | S | 0 | Neutral | rs1060915 |
| 17,41234470,A,G | GAC TC[T/C] TCT | 207 | S | S | 0 | Neutral | rs1060915 |
| 17,41234470,A,G | GAC TC[T/C] TCT | 332 | S | S | 0 | Neutral | rs1060915 |
| 17,41234470,A,G | GAC TC[T/C] TCT | 333 | S | S | 0 | Neutral | rs1060915 |
| 17,41223094,T,C | CAG [A/G]GT CCA | 1317 | S | G | -0.44 | Neutral | rs1799966 |
| 17,41223094,T,C | CAG [A/G]GT CCA | 471 | S | G | -2.09 | Neutral | rs1799966 |
| 17,41223094,T,C | CAG [A/G]GT CCA | 430 | S | G | -1.95 | Neutral | rs1799966 |
| 17,41223094,T,C | CAG [A/G]GT CCA | 1613 | S | G | -0.51 | Neutral | rs1799966 |
| 17,41223094,T,C | CAG [A/G]GT CCA | 462 | S | G | -1.99 | Neutral | rs1799966 |
| 17,41223094,T,C | CAG [A/G]GT CCA | 1634 | S | G | -0.51 | Neutral | rs1799966 |
| 17,41223094,T,C | CAG [A/G]GT CCA | 509 | S | G | -1.86 | Neutral | rs1799966 |
| 17,41223094,T,C | CAG [A/G]GT CCA | 1566 | S | G | -0.5 | Neutral | rs1799966 |
| 17,41223094,T,C | CAG [A/G]GT CCA | 463 | S | G | -1.83 | Neutral | rs1799966 |
| 17,41223094,T,C | CAG [A/G]GT CCA | 1635 | S | G | -0.51 | Neutral | rs1799966 |
| 17,41223094,T,C | CAG [A/G]GT CCA | 384 | S | G | -1.76 | Neutral | rs1799966 |
| 17,41223094,T,C | CAG [A/G]GT CCA | 509 | S | G | -1.45 | Neutral | rs1799966 |
| 17,41223094,T,C | CAG [A/G]GT CCA | 509 | S | G | -1.99 | Neutral | rs1799966 |
| 17,41244000,T,C | CAG A[A/G]A GGA | 1183 | K | R | 0.49 | Neutral | rs16942 |
| 17,41244000,T,C | CAG A[A/G]A GGA | 887 | K | R | 0.29 | Neutral | rs16942 |
| 17,41244000,T,C | CAG A[A/G]A GGA | 1183 | K | R | 0.49 | Neutral | rs16942 |
| 17,41244000,T,C | CAG A[A/G]A GGA | 1183 | K | R | 0.4 | Neutral | rs16942 |
| 17,41244000,T,C | CAG A[A/G]A GGA | 1183 | K | R | 0.42 | Neutral | rs16942 |
| 17,41244000,T,C | CAG A[A/G]A GGA | 1136 | K | R | 0.38 | Neutral | rs16942 |
| 17,41244000,T,C | CAG A[A/G]A GGA | 1183 | K | R | 0.42 | Neutral | rs16942 |
| 17,41245237,A,G | TCA [T/C]TG GTA | 771 | L | L | 0 | Neutral | rs16940 |
| 17,41245237,A,G | TCA [T/C]TG GTA | 475 | L | L | 0 | Neutral | rs16940 |
| 17,41245237,A,G | TCA [T/C]TG GTA | 771 | L | L | 0 | Neutral | rs16940 |
| 17,41245237,A,G | TCA [T/C]TG GTA | 771 | L | L | 0 | Neutral | rs16940 |
| 17,41245237,A,G | TCA [T/C]TG GTA | 771 | L | L | 0 | Neutral | rs16940 |
| 17,41245237,A,G | TCA [T/C]TG GTA | 724 | L | L | 0 | Neutral | rs16940 |
| 17,41245237,A,G | TCA [T/C]TG GTA | 771 | L | L | 0 | Neutral | rs16940 |
| 19,48654553,G,T | GTG GC[C/A] ACA | 170 | A | A | 0 | Neutral | rs20580 |
| 19,48654553,G,T | GTG GC[C/A] ACA | 201 | A | A | 0 | Neutral | rs20580 |
| 19,48654553,G,T | GTG GC[C/A] ACA | 139 | A | A | 0 | Neutral | rs20580 |
| 19,48654553,G,T | GTG GC[C/A] ACA | 138 | A | A | 0 | Neutral | rs20580 |
| 1,242035438,G,A | GAA [G/A]TG TTT | 458 | V | M | 0.48 | Neutral | rs4149965 |
| 1,242035438,G,A | GAA [G/A]TG TTT | 458 | V | M | 0.48 | Neutral | rs4149965 |
| 1,242035438,G,A | GAA [G/A]TG TTT | 458 | V | M | 0.48 | Neutral | rs4149965 |
| 1,226578278,C,A | CCA CA[G/T] CTA | 150 | Q | H | -1.18 | Neutral | rs142376976 |
| 1,226578278,C,A | CCA CA[G/T] CTA | 150 | Q | H | -1.79 | Neutral | rs142376976 |
| 1,242030151,A,G | AGT C[A/G]T AGT | 354 | H | R | -0.92 | Neutral | rs735943 |
| 1,242030151,A,G | AGT C[A/G]T AGT | 354 | H | R | -0.92 | Neutral | rs735943 |
| 1,242030151,A,G | AGT C[A/G]T AGT | 354 | H | R | -0.92 | Neutral | rs735943 |
| 1,242035438,G,A | GAA [G/A]TG TTT | 458 | V | M | 0.48 | Neutral | rs4149965 |
| 1,242035438,G,A | GAA [G/A]TG TTT | 458 | V | M | 0.48 | Neutral | rs4149965 |
| 1,242035438,G,A | GAA [G/A]TG TTT | 458 | V | M | 0.48 | Neutral | rs4149965 |
| 1,51439777,C,T | GAA GG[C/T] CAC | 114 | G | G | 0 | Neutral | rs1043141 |
| 1,51439777,C,T | GAA GG[C/T] CAC | 114 | G | G | 0 | Neutral | rs1043141 |
| 1,51439777,C,T | GAA GG[C/T] CAC | 114 | G | G | 0 | Neutral | rs1043141 |
| 1,23847464,C,A | GAC CA[G/T] CTC | 226 | Q | H | -1.61 | Neutral | rs2075995 |
| 1,242042301,G,A | TTT [G/A]AG AGC | 589 | E | K | -0.18 | Neutral | rs1047840 |
| 1,242042301,G,A | TTT [G/A]AG AGC | 589 | E | K | -0.18 | Neutral | rs1047840 |
| 1,242042301,G,A | TTT [G/A]AG AGC | 589 | E | K | -0.12 | Neutral | rs1047840 |
| 1,3638674,C,T | GGC AC[C/T] GCC | 173 | T | T | 0 | Neutral | rs1801174 |
| 1,3638674,C,T | GGC AC[C/T] GCC | 173 | T | T | 0 | Neutral | rs1801174 |
| 1,3638674,C,T | GGC AC[C/T] GCC | 173 | T | T | 0 | Neutral | rs1801174 |
| 1,3638674,C,T | GGC AC[C/T] GCC | 124 | T | T | 0 | Neutral | rs1801174 |
| 1,3638674,C,T | GGC AC[C/T] GCC | 124 | T | T | 0 | Neutral | rs1801174 |
| 1,3638674,C,T | GGC AC[C/T] GCC | 124 | T | T | 0 | Neutral | rs1801174 |
| 1,3638674,C,T | GGC AC[C/T] GCC | 102 | T | T | 0 | Neutral | rs1801174 |
| 1,3638674,C,T | GGC AC[C/T] GCC | 173 | T | T | 0 | Neutral | rs1801174 |
| 1,23847464,C,A | GAC CA[G/T] CTC | 226 | Q | H | -1.61 | Neutral | rs2075995 |
| 1,242042301,G,A | TTT [G/A]AG AGC | 589 | E | K | -0.18 | Neutral | rs1047840 |
| 1,242042301,G,A | TTT [G/A]AG AGC | 589 | E | K | -0.18 | Neutral | rs1047840 |
| 1,242042301,G,A | TTT [G/A]AG AGC | 589 | E | K | -0.12 | Neutral | rs1047840 |
| 1,59247993,C,T | TCC CA[G/A] GAG | 250 | Q | Q | 0 | Neutral | rs11688 |
| 1,45797505,C,G | GGA CA[G/C] TGC | 311 | Q | H | -1.03 | Neutral | rs3219489 |
| 1,45797505,C,G | GGA CA[G/C] TGC | 310 | Q | H | -1.03 | Neutral | rs3219489 |
| 1,45797505,C,G | GGA CA[G/C] TGC | 335 | Q | H | -1.03 | Neutral | rs3219489 |
| 1,45797505,C,G | GGA CA[G/C] TGC | 321 | Q | H | -1.03 | Neutral | rs3219489 |
| 1,45797505,C,G | GGA CA[G/C] TGC | 310 | Q | H | -1.03 | Neutral | rs3219489 |
| 1,45797505,C,G | GGA CA[G/C] TGC | 325 | Q | H | -1.03 | Neutral | rs3219489 |
| 1,45797505,C,G | GGA CA[G/C] TGC | 324 | Q | H | -1.03 | Neutral | rs3219489 |
| 1,45797505,C,G | GGA CA[G/C] TGC | 310 | Q | H | -1.03 | Neutral | rs3219489 |
| 1,45797505,C,G | GGA CA[G/C] TGC | 338 | Q | H | -1.03 | Neutral | rs3219489 |
| 1,45797505,C,G | GGA CA[G/C] TGC | 321 | Q | H | -1.03 | Neutral | rs3219489 |
| 1,45797505,C,G | GGA CA[G/C] TGC | 182 | Q | H | -1.03 | Neutral | rs3219489 |
| 1,45797505,C,G | GGA CA[G/C] TGC | 182 | Q | H | -1.03 | Neutral | rs3219489 |
| 1,45797505,C,G | GAC A[G/C]T GCC | 116 | S | T | 0.02 | Neutral | rs3219489 |
| 1,3649562,G,A | TGG GC[G/A] GAC | 514 | A | A | 0 | Neutral | rs9662633 |
| 1,3649562,G,A | TGG GC[G/A] GAC | 529 | A | A | 0 | Neutral | rs9662633 |
| 1,3649562,G,A | TGG GC[G/A] GAC | 561 | A | A | 0 | Neutral | rs9662633 |
| 1,3649562,G,A | TGG GC[G/A] GAC | 539 | A | A | 0 | Neutral | rs9662633 |
| 1,3649562,G,A | TGG GC[G/A] GAC | 610 | A | A | 0 | Neutral | rs9662633 |
| 1,242030151,A,G | AGT C[A/G]T AGT | 354 | H | R | -0.92 | Neutral | rs735943 |
| 1,242030151,A,G | AGT C[A/G]T AGT | 354 | H | R | -0.92 | Neutral | rs735943 |
| 1,242030151,A,G | AGT C[A/G]T AGT | 354 | H | R | -0.92 | Neutral | rs735943 |
| 1,242042545,A,G | GAA G[A/G]G GCA | 670 | E | G | -1.55 | Neutral | rs1776148 |
| 1,242042545,A,G | GAA G[A/G]G GCA | 670 | E | G | -1.55 | Neutral | rs1776148 |
| 1,242042545,A,G | GAA G[A/G]G GCA | 69 | E | G | -0.79 | Neutral | rs1776148 |
| 1,242042545,A,G | GAA G[A/G]G GCA | 670 | E | G | -1.51 | Neutral | rs1776148 |
| 1,45797505,C,G | GGA CA[G/C] TGC | 311 | Q | H | -1.03 | Neutral | rs3219489 |
| 1,45797505,C,G | GGA CA[G/C] TGC | 310 | Q | H | -1.03 | Neutral | rs3219489 |
| 1,45797505,C,G | GGA CA[G/C] TGC | 335 | Q | H | -1.03 | Neutral | rs3219489 |
| 1,45797505,C,G | GGA CA[G/C] TGC | 321 | Q | H | -1.03 | Neutral | rs3219489 |
| 1,45797505,C,G | GGA CA[G/C] TGC | 310 | Q | H | -1.03 | Neutral | rs3219489 |
| 1,45797505,C,G | GGA CA[G/C] TGC | 325 | Q | H | -1.03 | Neutral | rs3219489 |
| 1,45797505,C,G | GGA CA[G/C] TGC | 324 | Q | H | -1.03 | Neutral | rs3219489 |
| 1,45797505,C,G | GGA CA[G/C] TGC | 310 | Q | H | -1.03 | Neutral | rs3219489 |
| 1,45797505,C,G | GGA CA[G/C] TGC | 338 | Q | H | -1.03 | Neutral | rs3219489 |
| 1,45797505,C,G | GGA CA[G/C] TGC | 321 | Q | H | -1.03 | Neutral | rs3219489 |
| 1,45797505,C,G | GGA CA[G/C] TGC | 182 | Q | H | -1.03 | Neutral | rs3219489 |
| 1,45797505,C,G | GGA CA[G/C] TGC | 182 | Q | H | -1.03 | Neutral | rs3219489 |
| 1,45797505,C,G | GAC A[G/C]T GCC | 116 | S | T | 0.02 | Neutral | rs3219489 |
| 1,23847464,C,A | GAC CA[G/T] CTC | 226 | Q | H | -1.61 | Neutral | rs2075995 |
| 1,242042301,G,A | TTT [G/A]AG AGC | 589 | E | K | -0.18 | Neutral | rs1047840 |
| 1,242042301,G,A | TTT [G/A]AG AGC | 589 | E | K | -0.18 | Neutral | rs1047840 |
| 1,242042301,G,A | TTT [G/A]AG AGC | 589 | E | K | -0.12 | Neutral | rs1047840 |
| 1,59247993,C,T | TCC CA[G/A] GAG | 250 | Q | Q | 0 | Neutral | rs11688 |
| 1,45797505,C,G | GGA CA[G/C] TGC | 311 | Q | H | -1.03 | Neutral | rs3219489 |
| 1,45797505,C,G | GGA CA[G/C] TGC | 310 | Q | H | -1.03 | Neutral | rs3219489 |
| 1,45797505,C,G | GGA CA[G/C] TGC | 335 | Q | H | -1.03 | Neutral | rs3219489 |
| 1,45797505,C,G | GGA CA[G/C] TGC | 321 | Q | H | -1.03 | Neutral | rs3219489 |
| 1,45797505,C,G | GGA CA[G/C] TGC | 310 | Q | H | -1.03 | Neutral | rs3219489 |
| 1,45797505,C,G | GGA CA[G/C] TGC | 325 | Q | H | -1.03 | Neutral | rs3219489 |
| 1,45797505,C,G | GGA CA[G/C] TGC | 324 | Q | H | -1.03 | Neutral | rs3219489 |
| 1,45797505,C,G | GGA CA[G/C] TGC | 310 | Q | H | -1.03 | Neutral | rs3219489 |
| 1,45797505,C,G | GGA CA[G/C] TGC | 338 | Q | H | -1.03 | Neutral | rs3219489 |
| 1,45797505,C,G | GGA CA[G/C] TGC | 321 | Q | H | -1.03 | Neutral | rs3219489 |
| 1,45797505,C,G | GGA CA[G/C] TGC | 182 | Q | H | -1.03 | Neutral | rs3219489 |
| 1,45797505,C,G | GGA CA[G/C] TGC | 182 | Q | H | -1.03 | Neutral | rs3219489 |
| 1,45797505,C,G | GAC A[G/C]T GCC | 116 | S | T | 0.02 | Neutral | rs3219489 |
| 1,3649562,G,A | TGG GC[G/A] GAC | 514 | A | A | 0 | Neutral | rs9662633 |
| 1,3649562,G,A | TGG GC[G/A] GAC | 529 | A | A | 0 | Neutral | rs9662633 |
| 1,3649562,G,A | TGG GC[G/A] GAC | 561 | A | A | 0 | Neutral | rs9662633 |
| 1,3649562,G,A | TGG GC[G/A] GAC | 539 | A | A | 0 | Neutral | rs9662633 |
| 1,3649562,G,A | TGG GC[G/A] GAC | 610 | A | A | 0 | Neutral | rs9662633 |
| 1,242030151,A,G | AGT C[A/G]T AGT | 354 | H | R | -0.92 | Neutral | rs735943 |
| 1,242030151,A,G | AGT C[A/G]T AGT | 354 | H | R | -0.92 | Neutral | rs735943 |
| 1,242030151,A,G | AGT C[A/G]T AGT | 354 | H | R | -0.92 | Neutral | rs735943 |
| 1,242030151,A,G | AGT C[A/G]T AGT | 354 | H | R | -0.92 | Neutral | rs735943 |
| 1,242030151,A,G | AGT C[A/G]T AGT | 354 | H | R | -0.92 | Neutral | rs735943 |
| 1,242030151,A,G | AGT C[A/G]T AGT | 354 | H | R | -0.92 | Neutral | rs735943 |
| 1,242042301,G,A | TTT [G/A]AG AGC | 589 | E | K | -0.18 | Neutral | rs1047840 |
| 1,242042301,G,A | TTT [G/A]AG AGC | 589 | E | K | -0.18 | Neutral | rs1047840 |
| 1,242042301,G,A | TTT [G/A]AG AGC | 589 | E | K | -0.12 | Neutral | rs1047840 |
| 1,3638674,C,T | GGC AC[C/T] GCC | 173 | T | T | 0 | Neutral | rs1801174 |
| 1,3638674,C,T | GGC AC[C/T] GCC | 173 | T | T | 0 | Neutral | rs1801174 |
| 1,3638674,C,T | GGC AC[C/T] GCC | 173 | T | T | 0 | Neutral | rs1801174 |
| 1,3638674,C,T | GGC AC[C/T] GCC | 124 | T | T | 0 | Neutral | rs1801174 |
| 1,3638674,C,T | GGC AC[C/T] GCC | 124 | T | T | 0 | Neutral | rs1801174 |
| 1,3638674,C,T | GGC AC[C/T] GCC | 124 | T | T | 0 | Neutral | rs1801174 |
| 1,3638674,C,T | GGC AC[C/T] GCC | 102 | T | T | 0 | Neutral | rs1801174 |
| 1,3638674,C,T | GGC AC[C/T] GCC | 173 | T | T | 0 | Neutral | rs1801174 |
| 1,51439777,C,T | GAA GG[C/T] CAC | 114 | G | G | 0 | Neutral | rs1043141 |
| 1,51439777,C,T | GAA GG[C/T] CAC | 114 | G | G | 0 | Neutral | rs1043141 |
| 1,51439777,C,T | GAA GG[C/T] CAC | 114 | G | G | 0 | Neutral | rs1043141 |
| 1,242035438,G,A | GAA [G/A]TG TTT | 458 | V | M | 0.48 | Neutral | rs4149965 |
| 1,242035438,G,A | GAA [G/A]TG TTT | 458 | V | M | 0.48 | Neutral | rs4149965 |
| 1,242035438,G,A | GAA [G/A]TG TTT | 458 | V | M | 0.48 | Neutral | rs4149965 |
| 1,51439777,C,T | GAA GG[C/T] CAC | 114 | G | G | 0 | Neutral | rs1043141 |
| 1,51439777,C,T | GAA GG[C/T] CAC | 114 | G | G | 0 | Neutral | rs1043141 |
| 1,51439777,C,T | GAA GG[C/T] CAC | 114 | G | G | 0 | Neutral | rs1043141 |
| 1,23847464,C,A | GAC CA[G/T] CTC | 226 | Q | H | -1.61 | Neutral | rs2075995 |
| 1,242042301,G,A | TTT [G/A]AG AGC | 589 | E | K | -0.18 | Neutral | rs1047840 |
| 1,242042301,G,A | TTT [G/A]AG AGC | 589 | E | K | -0.18 | Neutral | rs1047840 |
| 1,242042301,G,A | TTT [G/A]AG AGC | 589 | E | K | -0.12 | Neutral | rs1047840 |
| 1,45800156,C,T | GCC [G/A]TG GGA | 8 | V | M | -0.56 | Neutral | rs3219484 |
| 1,45800156,C,T | GCC [G/A]TG GGA | 8 | V | M | -0.65 | Neutral | rs3219484 |
| 1,45800156,C,T | GCC [G/A]TG GGA | 22 | V | M | -0.6 | Neutral | rs3219484 |
| 1,45800156,C,T | GCC [G/A]TG GGA | 8 | V | M | -0.59 | Neutral | rs3219484 |
| 1,45800156,C,T | GCC [G/A]TG GGA | 8 | V | M | -0.65 | Neutral | rs3219484 |
| 1,45800156,C,T | GCC [G/A]TG GGA | 22 | V | M | -0.58 | Neutral | rs3219484 |
| 1,45800156,C,T | GCC [G/A]TG GGA | 22 | V | M | -0.64 | Neutral | rs3219484 |
| 1,45800156,C,T | GCC [G/A]TG GGA | 8 | V | M | -0.53 | Neutral | rs3219484 |
| 1,45800156,C,T | GCC [G/A]TG GGA | 8 | V | M | -0.65 | Neutral | rs3219484 |
| 1,45800156,C,T | GCC [G/A]TG GGA | 22 | V | M | -0.57 | Neutral | rs3219484 |
| 1,45800156,C,T | GCC [G/A]TG GGA | 8 | V | M | -0.59 | Neutral | rs3219484 |
| 1,45800156,C,T | GCC [G/A]TG GGA | 8 | V | M | -1.06 | Neutral | rs3219484 |
| 1,45800156,C,T | GCC [G/A]TG GGA | 22 | V | M | -1.38 | Neutral | rs3219484 |
| 1,45800156,C,T | GCC [G/A]TG GGA | 8 | V | M | -0.45 | Neutral | rs3219484 |
| 1,45800156,C,T | GCC [G/A]TG GGA | 22 | V | M | -0.26 | Neutral | rs3219484 |
| 1,45800156,C,T | GCC [G/A]TG GGA | 8 | V | M | -0.62 | Neutral | rs3219484 |
| 1,45800156,C,T | GCC [G/A]TG GGA | 8 | V | M | -1.08 | Neutral | rs3219484 |
| 1,45800156,C,T | GCC [G/A]TG GGA | 8 | V | M | -0.86 | Neutral | rs3219484 |
| 1,45800156,C,T | GCC [G/A]TG GGA | 10 | V | M | -1.38 | Neutral | rs3219484 |
| 1,45800156,C,T | GCC [G/A]TG GGA | 14 | V | M | -0.86 | Neutral | rs3219484 |
| 1,45800156,C,T | GCC [G/A]TG GGA | 22 | V | M | -1.4 | Neutral | rs3219484 |
| 1,45800156,C,T | GCC [G/A]TG GGA | 8 | V | M | -0.45 | Neutral | rs3219484 |
| 1,45800156,C,T | GCC [G/A]TG GGA | 22 | V | M | -1.4 | Neutral | rs3219484 |
| 1,242042301,G,A | TTT [G/A]AG AGC | 589 | E | K | -0.18 | Neutral | rs1047840 |
| 1,242042301,G,A | TTT [G/A]AG AGC | 589 | E | K | -0.18 | Neutral | rs1047840 |
| 1,242042301,G,A | TTT [G/A]AG AGC | 589 | E | K | -0.12 | Neutral | rs1047840 |
| 1,242042301,G,A | TTT [G/A]AG AGC | 589 | E | K | -0.18 | Neutral | rs1047840 |
| 1,242042301,G,A | TTT [G/A]AG AGC | 589 | E | K | -0.18 | Neutral | rs1047840 |
| 1,242042301,G,A | TTT [G/A]AG AGC | 589 | E | K | -0.12 | Neutral | rs1047840 |
| 1,226570840,T,C | AAA AA[A/G] CAG | 352 | K | K | 0 | Neutral | rs1805415 |
| 1,242035438,G,A | GAA [G/A]TG TTT | 458 | V | M | 0.48 | Neutral | rs4149965 |
| 1,242035438,G,A | GAA [G/A]TG TTT | 458 | V | M | 0.48 | Neutral | rs4149965 |
| 1,242035438,G,A | GAA [G/A]TG TTT | 458 | V | M | 0.48 | Neutral | rs4149965 |
| 1,51439777,C,T | GAA GG[C/T] CAC | 114 | G | G | 0 | Neutral | rs1043141 |
| 1,51439777,C,T | GAA GG[C/T] CAC | 114 | G | G | 0 | Neutral | rs1043141 |
| 1,51439777,C,T | GAA GG[C/T] CAC | 114 | G | G | 0 | Neutral | rs1043141 |
| 1,242035438,G,A | GAA [G/A]TG TTT | 458 | V | M | 0.48 | Neutral | rs4149965 |
| 1,242035438,G,A | GAA [G/A]TG TTT | 458 | V | M | 0.48 | Neutral | rs4149965 |
| 1,242035438,G,A | GAA [G/A]TG TTT | 458 | V | M | 0.48 | Neutral | rs4149965 |
| 1,45797505,C,G | GGA CA[G/C] TGC | 311 | Q | H | -1.03 | Neutral | rs3219489 |
| 1,45797505,C,G | GGA CA[G/C] TGC | 310 | Q | H | -1.03 | Neutral | rs3219489 |
| 1,45797505,C,G | GGA CA[G/C] TGC | 335 | Q | H | -1.03 | Neutral | rs3219489 |
| 1,45797505,C,G | GGA CA[G/C] TGC | 321 | Q | H | -1.03 | Neutral | rs3219489 |
| 1,45797505,C,G | GGA CA[G/C] TGC | 310 | Q | H | -1.03 | Neutral | rs3219489 |
| 1,45797505,C,G | GGA CA[G/C] TGC | 325 | Q | H | -1.03 | Neutral | rs3219489 |
| 1,45797505,C,G | GGA CA[G/C] TGC | 324 | Q | H | -1.03 | Neutral | rs3219489 |
| 1,45797505,C,G | GGA CA[G/C] TGC | 310 | Q | H | -1.03 | Neutral | rs3219489 |
| 1,45797505,C,G | GGA CA[G/C] TGC | 338 | Q | H | -1.03 | Neutral | rs3219489 |
| 1,45797505,C,G | GGA CA[G/C] TGC | 321 | Q | H | -1.03 | Neutral | rs3219489 |
| 1,45797505,C,G | GGA CA[G/C] TGC | 182 | Q | H | -1.03 | Neutral | rs3219489 |
| 1,45797505,C,G | GGA CA[G/C] TGC | 182 | Q | H | -1.03 | Neutral | rs3219489 |
| 1,45797505,C,G | GAC A[G/C]T GCC | 116 | S | T | 0.02 | Neutral | rs3219489 |
| 1,242042545,A,G | GAA G[A/G]G GCA | 670 | E | G | -1.55 | Neutral | rs1776148 |
| 1,242042545,A,G | GAA G[A/G]G GCA | 670 | E | G | -1.55 | Neutral | rs1776148 |
| 1,242042545,A,G | GAA G[A/G]G GCA | 69 | E | G | -0.79 | Neutral | rs1776148 |
| 1,242042545,A,G | GAA G[A/G]G GCA | 670 | E | G | -1.51 | Neutral | rs1776148 |
| 10,103340144,A,G | AAC TC[T/C] GGG | 408 | S | S | 0 | Neutral | rs3730476 |
| 10,103340144,A,G | AAC TC[T/C] GGG | 131 | S | S | 0 | Neutral | rs3730476 |
| 10,103340144,A,G | AAC TC[T/C] GGG | 408 | S | S | 0 | Neutral | rs3730476 |
| 10,103340144,A,G | AAC TC[T/C] GGG | 133 | S | S | 0 | Neutral | rs3730476 |
| 10,103340144,A,G | AAC TC[T/C] GGG | 408 | S | S | 0 | Neutral | rs3730476 |
| 10,103340144,A,G | AAC TC[T/C] GGG | 81 | S | S | 0 | Neutral | rs3730476 |
| 10,103340144,A,G | AAC TC[T/C] GGG | 408 | S | S | 0 | Neutral | rs3730476 |
| 10,103340144,A,G | AAC TC[T/C] GGG | 320 | S | S | 0 | Neutral | rs3730476 |
| 10,103340144,A,G | AAC TC[T/C] GGG | 408 | S | S | 0 | Neutral | rs3730476 |
| 10,103340144,A,G | AAC TC[T/C] GGG | 145 | S | S | 0 | Neutral | rs3730476 |
| 10,103340144,A,G | AAC TC[T/C] GGG | 316 | S | S | 0 | Neutral | rs3730476 |
| 10,103340144,A,G | AAC TC[T/C] GGG | 131 | S | S | 0 | Neutral | rs3730476 |
| 10,50678317,C,G | AGG C[G/C]T TAC | 1230 | R | P | -2.29 | Neutral | rs4253211 |
| 10,50678317,C,G | AGG C[G/C]T TAC | 607 | R | P | -2.42 | Neutral | rs4253211 |
| 10,50678317,C,G | AGG C[G/C]T TAC | 600 | R | P | -2.42 | Neutral | rs4253211 |
| 10,50732280,C,T | GAT G[G/A]T ACT | 399 | G | D | 1.23 | Neutral | rs2228528 |
| 10,50732280,C,T | GAT G[G/A]T ACT | 399 | G | D | 0.57 | Neutral | rs2228528 |
| 10,50732280,C,T | GAT G[G/A]T ACT | 399 | G | D | 0.57 | Neutral | rs2228528 |
| 10,50732280,C,T | GAT G[G/A]T ACT | 399 | G | D | 1.23 | Neutral | rs2228528 |
| 10,50732280,C,T | GAT G[G/A]T ACT | 399 | G | D | 0.57 | Neutral | rs2228528 |
| 10,50732280,C,T | GAT G[G/A]T ACT | 399 | G | D | 0.57 | Neutral | rs2228528 |
| 10,49643060,C,G | CTG GG[C/G] TGC | 424 | G | G | 0 | Neutral |  |
| 10,49643060,C,G | CTG GG[C/G] TGC | 424 | G | G | 0 | Neutral |  |
| 10,49643060,C,G | CTG GG[C/G] TGC | 424 | G | G | 0 | Neutral |  |
| 10,50681033,G,A | GTG GG[C/T] GGC | 917 | G | G | 0 | Neutral | rs2229760 |
| 10,50681033,G,A | GTG GG[C/T] GGC | 294 | G | G | 0 | Neutral | rs2229760 |
| 10,50681033,G,A | GTG GG[C/T] GGC | 287 | G | G | 0 | Neutral | rs2229760 |
| 10,50678717,T,C | CAC [A/G]TG AGT | 1097 | M | V | 0.32 | Neutral | rs2228526 |
| 10,50678717,T,C | CAC [A/G]TG AGT | 474 | M | V | 0.2 | Neutral | rs2228526 |
| 10,50678717,T,C | CAC [A/G]TG AGT | 467 | M | V | 0.17 | Neutral | rs2228526 |
| 10,50740876,G,C | TAC CT[C/G] TCC | 45 | L | L | 0 | Neutral | rs2228524 |
| 10,50740876,G,C | TAC CT[C/G] TCC | 45 | L | L | 0 | Neutral | rs2228524 |
| 10,50740876,G,C | TAC CT[C/G] TCC | 45 | L | L | 0 | Neutral | rs2228524 |
| 10,50740876,G,C | TAC CT[C/G] TCC | 45 | L | L | 0 | Neutral | rs2228524 |
| 10,50732280,C,T | GAT G[G/A]T ACT | 399 | G | D | 1.23 | Neutral | rs2228528 |
| 10,50732280,C,T | GAT G[G/A]T ACT | 399 | G | D | 0.57 | Neutral | rs2228528 |
| 10,50732280,C,T | GAT G[G/A]T ACT | 399 | G | D | 0.57 | Neutral | rs2228528 |
| 10,50667105,T,C | CTG C[A/G]G GAA | 1413 | Q | R | -1.09 | Neutral | rs2228529 |
| 10,50667105,T,C | CTG C[A/G]G GAA | 790 | Q | R | -1.15 | Neutral | rs2228529 |
| 10,50667105,T,C | CTG C[A/G]G GAA | 783 | Q | R | -1.09 | Neutral | rs2228529 |
| 10,49643060,C,G | CTG GG[C/G] TGC | 424 | G | G | 0 | Neutral |  |
| 10,49643060,C,G | CTG GG[C/G] TGC | 424 | G | G | 0 | Neutral |  |
| 10,49643060,C,G | CTG GG[C/G] TGC | 424 | G | G | 0 | Neutral |  |
| 10,50678717,T,C | CAC [A/G]TG AGT | 1097 | M | V | 0.32 | Neutral | rs2228526 |
| 10,50678717,T,C | CAC [A/G]TG AGT | 474 | M | V | 0.2 | Neutral | rs2228526 |
| 10,50678717,T,C | CAC [A/G]TG AGT | 467 | M | V | 0.17 | Neutral | rs2228526 |
| 10,50667105,T,C | CTG C[A/G]G GAA | 1413 | Q | R | -1.09 | Neutral | rs2228529 |
| 10,50667105,T,C | CTG C[A/G]G GAA | 790 | Q | R | -1.15 | Neutral | rs2228529 |
| 10,50667105,T,C | CTG C[A/G]G GAA | 783 | Q | R | -1.09 | Neutral | rs2228529 |
| 10,50732318,G,C | GAT GA[C/G] GAG | 386 | D | E | 0.8 | Neutral | rs141391984 |
| 10,50732318,G,C | GAT GA[C/G] GAG | 386 | D | E | 0.49 | Neutral | rs141391984 |
| 10,50732318,G,C | GAT GA[C/G] GAG | 386 | D | E | 0.49 | Neutral | rs141391984 |
| 10,50678717,T,C | CAC [A/G]TG AGT | 1097 | M | V | 0.32 | Neutral | rs2228526 |
| 10,50678717,T,C | CAC [A/G]TG AGT | 474 | M | V | 0.2 | Neutral | rs2228526 |
| 10,50678717,T,C | CAC [A/G]TG AGT | 467 | M | V | 0.17 | Neutral | rs2228526 |
| 10,50740876,G,C | TAC CT[C/G] TCC | 45 | L | L | 0 | Neutral | rs2228524 |
| 10,50740876,G,C | TAC CT[C/G] TCC | 45 | L | L | 0 | Neutral | rs2228524 |
| 10,50740876,G,C | TAC CT[C/G] TCC | 45 | L | L | 0 | Neutral | rs2228524 |
| 10,50740876,G,C | TAC CT[C/G] TCC | 45 | L | L | 0 | Neutral | rs2228524 |
| 10,50682192,G,A | GAA [C/T]TA GAA | 827 | L | L | 0 | Neutral | rs115875661 |
| 10,50682192,G,A | GAA [C/T]TA GAA | 204 | L | L | 0 | Neutral | rs115875661 |
| 10,50682192,G,A | GAA [C/T]TA GAA | 197 | L | L | 0 | Neutral | rs115875661 |
| 10,50690778,G,A | TTC TC[C/T] GTC | 708 | S | S | 0 | Neutral | rs114832108 |
| 10,50690778,G,A | TTC TC[C/T] GTC | 117 | S | S | 0 | Neutral | rs114832108 |
| 10,50690778,G,A | TTC TC[C/T] GTC | 78 | S | S | 0 | Neutral | rs114832108 |
| 10,50732280,C,T | GAT G[G/A]T ACT | 399 | G | D | 1.23 | Neutral | rs2228528 |
| 10,50732280,C,T | GAT G[G/A]T ACT | 399 | G | D | 0.57 | Neutral | rs2228528 |
| 10,50732280,C,T | GAT G[G/A]T ACT | 399 | G | D | 0.57 | Neutral | rs2228528 |
| 10,50678717,T,C | CAC [A/G]TG AGT | 1097 | M | V | 0.32 | Neutral | rs2228526 |
| 10,50678717,T,C | CAC [A/G]TG AGT | 474 | M | V | 0.2 | Neutral | rs2228526 |
| 10,50678717,T,C | CAC [A/G]TG AGT | 467 | M | V | 0.17 | Neutral | rs2228526 |
| 10,50732318,G,C | GAT GA[C/G] GAG | 386 | D | E | 0.8 | Neutral | rs141391984 |
| 10,50732318,G,C | GAT GA[C/G] GAG | 386 | D | E | 0.49 | Neutral | rs141391984 |
| 10,50732318,G,C | GAT GA[C/G] GAG | 386 | D | E | 0.49 | Neutral | rs141391984 |
| 10,50667105,T,C | CTG C[A/G]G GAA | 1413 | Q | R | -1.09 | Neutral | rs2228529 |
| 10,50667105,T,C | CTG C[A/G]G GAA | 790 | Q | R | -1.15 | Neutral | rs2228529 |
| 10,50667105,T,C | CTG C[A/G]G GAA | 783 | Q | R | -1.09 | Neutral | rs2228529 |
| 10,50740876,G,C | TAC CT[C/G] TCC | 45 | L | L | 0 | Neutral | rs2228524 |
| 10,50740876,G,C | TAC CT[C/G] TCC | 45 | L | L | 0 | Neutral | rs2228524 |
| 10,50740876,G,C | TAC CT[C/G] TCC | 45 | L | L | 0 | Neutral | rs2228524 |
| 10,50740876,G,C | TAC CT[C/G] TCC | 45 | L | L | 0 | Neutral | rs2228524 |
| 10,50681033,G,A | GTG GG[C/T] GGC | 917 | G | G | 0 | Neutral | rs2229760 |
| 10,50681033,G,A | GTG GG[C/T] GGC | 294 | G | G | 0 | Neutral | rs2229760 |
| 10,50681033,G,A | GTG GG[C/T] GGC | 287 | G | G | 0 | Neutral | rs2229760 |
| 10,50740876,G,C | TAC CT[C/G] TCC | 45 | L | L | 0 | Neutral | rs2228524 |
| 10,50740876,G,C | TAC CT[C/G] TCC | 45 | L | L | 0 | Neutral | rs2228524 |
| 10,50740876,G,C | TAC CT[C/G] TCC | 45 | L | L | 0 | Neutral | rs2228524 |
| 10,50740876,G,C | TAC CT[C/G] TCC | 45 | L | L | 0 | Neutral | rs2228524 |
| 11,108123551,C,T | TTT [C/T]CT CAT | 604 | P | S | -2.17 | Neutral | rs2227922 |
| 11,108123551,C,T | TTT [C/T]CT CAT | 604 | P | S | -2.17 | Neutral | rs2227922 |
| 11,108123551,C,T | TTT [C/T]CT CAT | 604 | P | S | -2.47 | Neutral | rs2227922 |
| 11,108138037,C,T | GAT G[C/T]A AAC | 869 | A | V | -1.09 | Neutral |  |
| 11,108138037,C,T | GAT G[C/T]A AAC | 869 | A | V | -1.09 | Neutral |  |
| 11,108138037,C,T | GAT G[C/T]A AAC | 129 | A | V | -2.35 | Neutral |  |
| 11,108138037,C,T | GAT G[C/T]A AAC | 869 | A | V | -1.17 | Neutral |  |
| 11,108158375,T,C | TTA [T/C]TG ATG | 1348 | L | L | 0 | Neutral | rs56355831 |
| 11,108158375,T,C | TTA [T/C]TG ATG | 1348 | L | L | 0 | Neutral | rs56355831 |
| 11,108158375,T,C | TTA [T/C]TG ATG | 18 | L | L | 0 | Neutral | rs56355831 |
| 11,108158375,T,C | TTA [T/C]TG ATG | 1348 | L | L | 0 | Neutral | rs56355831 |
| 11,108173749,T,C | CCA A[T/C]G TGT | 1830 | M | T | -2.46 | Neutral | rs145812395 |
| 11,108173749,T,C | CCA A[T/C]G TGT | 1830 | M | T | -2.46 | Neutral | rs145812395 |
| 11,108121446,A,G | ACC CA[A/G] TTA | 418 | Q | Q | 0 | Neutral | rs4987943 |
| 11,108121446,A,G | ACC CA[A/G] TTA | 418 | Q | Q | 0 | Neutral | rs4987943 |
| 11,108121446,A,G | ACC CA[A/G] TTA | 418 | Q | Q | 0 | Neutral | rs4987943 |
| 11,108106443,T,A | ATG GA[T/A] ACA | 126 | D | E | -0.28 | Neutral | rs2234997 |
| 11,108106443,T,A | ATG GA[T/A] ACA | 126 | D | E | -0.28 | Neutral | rs2234997 |
| 11,108106443,T,A | ATG GA[T/A] ACA | 126 | D | E | -0.17 | Neutral | rs2234997 |
| 11,18369240,C,T | GTC [C/T]TG GCA | 315 | L | L | 0 | Neutral | rs145372677 |
| 11,18369240,C,T | GTC [C/T]TG GCA | 315 | L | L | 0 | Neutral | rs145372677 |
| 11,18369240,C,T | GTC [C/T]TG GCA | 3 | L | L | 0 | Neutral | rs145372677 |
| 11,18369240,C,T | GTC [C/T]TG GCA | 199 | L | L | 0 | Neutral | rs145372677 |
| 11,18369240,C,T | GTC [C/T]TG GCA | 111 | L | L | 0 | Neutral | rs145372677 |
| 11,108175462,G,A | CAA [G/A]AT ACA | 1853 | D | N | -1.31 | Neutral | rs1801516 |
| 11,108175462,G,A | CAA [G/A]AT ACA | 1853 | D | N | -1.31 | Neutral | rs1801516 |
| 11,61067686,G,A | TAT GA[C/T] GAT | 1115 | D | D | 0 | Neutral | rs138674997 |
| 11,61067686,G,A | TAT GA[C/T] GAT | 426 | D | D | 0 | Neutral | rs138674997 |
| 11,61067686,G,A | TAT GA[C/T] GAT | 102 | D | D | 0 | Neutral | rs138674997 |
| 11,61067686,G,A | TAT GA[C/T] GAT | 162 | D | D | 0 | Neutral | rs138674997 |
| 11,94209469,C,A | TTT GT[G/T] ATT | 215 | V | V | 0 | Neutral |  |
| 11,94209469,C,A | TTT GT[G/T] ATT | 215 | V | V | 0 | Neutral |  |
| 11,94209469,C,A | TTT GT[G/T] ATT | 215 | V | V | 0 | Neutral |  |
| 11,94209469,C,A | TTT GT[G/T] ATT | 218 | V | V | 0 | Neutral |  |
| 11,94209469,C,A | TTT GT[G/T] ATT | 215 | V | V | 0 | Neutral |  |
| 11,108106443,T,A | ATG GA[T/A] ACA | 126 | D | E | -0.28 | Neutral | rs2234997 |
| 11,108106443,T,A | ATG GA[T/A] ACA | 126 | D | E | -0.28 | Neutral | rs2234997 |
| 11,108106443,T,A | ATG GA[T/A] ACA | 126 | D | E | -0.17 | Neutral | rs2234997 |
| 11,18369240,C,T | GTC [C/T]TG GCA | 315 | L | L | 0 | Neutral | rs145372677 |
| 11,18369240,C,T | GTC [C/T]TG GCA | 315 | L | L | 0 | Neutral | rs145372677 |
| 11,18369240,C,T | GTC [C/T]TG GCA | 3 | L | L | 0 | Neutral | rs145372677 |
| 11,18369240,C,T | GTC [C/T]TG GCA | 199 | L | L | 0 | Neutral | rs145372677 |
| 11,18369240,C,T | GTC [C/T]TG GCA | 111 | L | L | 0 | Neutral | rs145372677 |
| 11,108175462,G,A | CAA [G/A]AT ACA | 1853 | D | N | -1.31 | Neutral | rs1801516 |
| 11,108175462,G,A | CAA [G/A]AT ACA | 1853 | D | N | -1.31 | Neutral | rs1801516 |
| 11,94209469,C,A | TTT GT[G/T] ATT | 215 | V | V | 0 | Neutral |  |
| 11,94209469,C,A | TTT GT[G/T] ATT | 215 | V | V | 0 | Neutral |  |
| 11,94209469,C,A | TTT GT[G/T] ATT | 215 | V | V | 0 | Neutral |  |
| 11,94209469,C,A | TTT GT[G/T] ATT | 218 | V | V | 0 | Neutral |  |
| 11,94209469,C,A | TTT GT[G/T] ATT | 215 | V | V | 0 | Neutral |  |
| 11,108175462,G,A | CAA [G/A]AT ACA | 1853 | D | N | -1.31 | Neutral | rs1801516 |
| 11,108175462,G,A | CAA [G/A]AT ACA | 1853 | D | N | -1.31 | Neutral | rs1801516 |
| 11,125525195,A,G | AAG [A/G]TT TGG | 427 | I | V | 0.4 | Neutral | rs506504 |
| 11,125525195,A,G | AAG [A/G]TT TGG | 471 | I | V | 0.38 | Neutral | rs506504 |
| 11,125525195,A,G | AAG [A/G]TT TGG | 487 | I | V | 0.42 | Neutral | rs506504 |
| 11,125525195,A,G | AAG [A/G]TT TGG | 471 | I | V | 0.38 | Neutral | rs506504 |
| 11,125525195,A,G | AAG [A/G]TT TGG | 471 | I | V | 0.38 | Neutral | rs506504 |
| 11,125525195,A,G | AAG [A/G]TT TGG | 471 | I | V | 0.38 | Neutral | rs506504 |
| 11,125525195,A,G | AAG [A/G]TT TGG | 437 | I | V | 0.35 | Neutral | rs506504 |
| 11,108175462,G,A | CAA [G/A]AT ACA | 1853 | D | N | -1.31 | Neutral | rs1801516 |
| 11,108175462,G,A | CAA [G/A]AT ACA | 1853 | D | N | -1.31 | Neutral | rs1801516 |
| 11,61099072,G,A | CGG CC[C/T] GTC | 51 | P | P | 0 | Neutral | rs2230356 |
| 11,61099072,G,A | CGG CC[C/T] GTC | 51 | P | P | 0 | Neutral | rs2230356 |
| 11,61099072,G,A | CGG CC[C/T] GTC | 51 | P | P | 0 | Neutral | rs2230356 |
| 11,61099072,G,A | CGG CC[C/T] GTC | 51 | P | P | 0 | Neutral | rs2230356 |
| 11,61099072,G,A | CGG CC[C/T] GTC | 51 | P | P | 0 | Neutral | rs2230356 |
| 11,61099072,G,A | CGG CC[C/T] GTC | 51 | P | P | 0 | Neutral | rs2230356 |
| 11,69462910,G,A | GAC CC[G/A] GAC | 241 | P | P | 0 | Neutral | rs9344 |
| 11,69462910,G,A | GAC CC[G/A] GAC | 107 | P | P | 0 | Neutral | rs9344 |
| 12,104376693,G,A | CGC CC[G/A] GCA | 22 | P | P | 0 | Neutral | rs4135113 |
| 12,12871099,T,G | GAT G[T/G]C AGC | 109 | V | G | -1.35 | Neutral | rs2066827 |
| 12,12871099,T,G | GAT G[T/G]C AGC | 109 | V | G | -2.06 | Neutral | rs2066827 |
| 12,12871099,T,G | GAT G[T/G]C AGC | 58 | V | G | -1.44 | Neutral | rs2066827 |
| 12,104380734,G,A | AGC [G/A]TG GAG | 363 | V | M | -0.59 | Neutral | rs2888805 |
| 12,104380734,G,A | AGC [G/A]TG GAG | 367 | V | M | -0.59 | Neutral | rs2888805 |
| 12,104380734,G,A | AGC [G/A]TG GAG | 163 | V | M | -0.81 | Neutral | rs2888805 |
| 12,104380734,G,A | AGC [G/A]TG GAG | 224 | V | M | -0.71 | Neutral | rs2888805 |
| 12,104373829,C,A | TTG AC[C/A] TTC | 125 | T | T | 0 | Neutral | rs75200561 |
| 12,104373829,C,A | TTG AC[C/A] TTC | 129 | T | T | 0 | Neutral | rs75200561 |
| 12,104373829,C,A | TTG AC[C/A] TTC | 104 | T | T | 0 | Neutral | rs75200561 |
| 12,104373829,C,A | TTG AC[C/A] TTC | 129 | T | T | 0 | Neutral | rs75200561 |
| 12,104373829,C,A | TTG AC[C/A] TTC | 33 | T | T | 0 | Neutral | rs75200561 |
| 12,133219831,T,C | AGG GC[A/G] TCC | 1510 | A | A | 0 | Neutral | rs5744944 |
| 12,133219831,T,C | AGG GC[A/G] TCC | 1521 | A | A | 0 | Neutral | rs5744944 |
| 12,133219831,T,C | AGG GC[A/G] TCC | 1483 | A | A | 0 | Neutral | rs5744944 |
| 12,133208979,T,C | CTC TC[A/G] GAG | 2084 | S | S | 0 | Neutral | rs5745022 |
| 12,133208979,T,C | CTC TC[A/G] GAG | 2095 | S | S | 0 | Neutral | rs5745022 |
| 12,133208979,T,C | CTC TC[A/G] GAG | 294 | S | S | 0 | Neutral | rs5745022 |
| 12,133208979,T,C | CTC TC[A/G] GAG | 2057 | S | S | 0 | Neutral | rs5745022 |
| 12,133236000,C,T | TCT AC[G/A] TCC | 1052 | T | T | 0 | Neutral | rs5744857 |
| 12,133236000,C,T | TCT AC[G/A] TCC | 987 | T | T | 0 | Neutral | rs5744857 |
| 12,133236000,C,T | TCT AC[G/A] TCC | 1063 | T | T | 0 | Neutral | rs5744857 |
| 12,133236000,C,T | TCT AC[G/A] TCC | 29 | T | T | 0 | Neutral | rs5744857 |
| 12,133236000,C,T | TCT AC[G/A] TCC | 832 | T | T | 0 | Neutral | rs5744857 |
| 12,133236000,C,T | TCT AC[G/A] TCC | 1025 | T | T | 0 | Neutral | rs5744857 |
| 12,12871099,T,G | GAT G[T/G]C AGC | 109 | V | G | -1.35 | Neutral | rs2066827 |
| 12,12871099,T,G | GAT G[T/G]C AGC | 109 | V | G | -2.06 | Neutral | rs2066827 |
| 12,12871099,T,G | GAT G[T/G]C AGC | 58 | V | G | -1.44 | Neutral | rs2066827 |
| 12,133237569,C,T | GAC [G/A]TG CTG | 1016 | V | M | -2.43 | Neutral | rs147692158 |
| 12,133237569,C,T | GAC [G/A]TG CTG | 951 | V | M | -2.48 | Neutral | rs147692158 |
| 12,133237569,C,T | GAC [G/A]TG CTG | 1027 | V | M | -2.43 | Neutral | rs147692158 |
| 12,133237569,C,T | GAC [G/A]TG CTG | 989 | V | M | -2.43 | Neutral | rs147692158 |
| 12,133219831,T,C | AGG GC[A/G] TCC | 1510 | A | A | 0 | Neutral | rs5744944 |
| 12,133219831,T,C | AGG GC[A/G] TCC | 1521 | A | A | 0 | Neutral | rs5744944 |
| 12,133219831,T,C | AGG GC[A/G] TCC | 1483 | A | A | 0 | Neutral | rs5744944 |
| 12,56360876,G,A | GGA GA[G/A] GTG | 28 | E | E | 0 | Neutral | rs2069398 |
| 12,56360876,G,A | GGA GA[G/A] GTG | 28 | E | E | 0 | Neutral | rs2069398 |
| 12,56360876,G,A | GGA GA[G/A] GTG | 28 | E | E | 0 | Neutral | rs2069398 |
| 12,56360876,G,A | GGA GA[G/A] GTG | 28 | E | E | 0 | Neutral | rs2069398 |
| 12,56360876,G,A | GGA GA[G/A] GTG | 28 | E | E | 0 | Neutral | rs2069398 |
| 12,56360876,G,A | GGA GA[G/A] GTG | 28 | E | E | 0 | Neutral | rs2069398 |
| 12,58143088,C,T | GGG CT[G/A] CCT | 232 | L | L | 0 | Neutral | rs2227953 |
| 12,58143088,C,T | GGG CT[G/A] CCT | 112 | L | L | 0 | Neutral | rs2227953 |
| 12,58143088,C,T | GGG CT[G/A] CCT | 158 | L | L | 0 | Neutral | rs2227953 |
| 12,58143088,C,T | GGG CT[G/A] CCT | 158 | L | L | 0 | Neutral | rs2227953 |
| 12,58144781,T,C | GTG AC[A/G] AGT | 149 | T | T | 0 | Neutral | rs2069501 |
| 12,58144781,T,C | GTG AC[A/G] AGT | 29 | T | T | 0 | Neutral | rs2069501 |
| 12,58144781,T,C | GTG AC[A/G] AGT | 75 | T | T | 0 | Neutral | rs2069501 |
| 12,58144781,T,C | GTG AC[A/G] AGT | 75 | T | T | 0 | Neutral | rs2069501 |
| 12,58144781,T,C | GTG AC[A/G] AGT | 149 | T | T | 0 | Neutral | rs2069501 |
| 12,58144781,T,C | GTG AC[A/G] AGT | 149 | T | T | 0 | Neutral | rs2069501 |
| 12,58144781,T,C | GTG AC[A/G] AGT | 149 | T | T | 0 | Neutral | rs2069501 |
| 12,58144781,T,C | GTG AC[A/G] AGT | 75 | T | T | 0 | Neutral | rs2069501 |
| 12,133201379,G,C | CAG AT[C/G] GGA | 225 | I | M | -0.51 | Neutral |  |
| 12,133201379,G,C | CAG AT[C/G] GGA | 2255 | I | M | -0.26 | Neutral |  |
| 12,133201379,G,C | CAG AT[C/G] GGA | 2266 | I | M | -0.26 | Neutral |  |
| 12,133201379,G,C | CAG AT[C/G] GGA | 465 | I | M | -0.56 | Neutral |  |
| 12,133201379,G,C | CAG AT[C/G] GGA | 2228 | I | M | -0.26 | Neutral |  |
| 12,133236000,C,T | TCT AC[G/A] TCC | 1052 | T | T | 0 | Neutral | rs5744857 |
| 12,133236000,C,T | TCT AC[G/A] TCC | 987 | T | T | 0 | Neutral | rs5744857 |
| 12,133236000,C,T | TCT AC[G/A] TCC | 1063 | T | T | 0 | Neutral | rs5744857 |
| 12,133236000,C,T | TCT AC[G/A] TCC | 29 | T | T | 0 | Neutral | rs5744857 |
| 12,133236000,C,T | TCT AC[G/A] TCC | 832 | T | T | 0 | Neutral | rs5744857 |
| 12,133236000,C,T | TCT AC[G/A] TCC | 1025 | T | T | 0 | Neutral | rs5744857 |
| 12,133208979,T,C | CTC TC[A/G] GAG | 2084 | S | S | 0 | Neutral | rs5745022 |
| 12,133208979,T,C | CTC TC[A/G] GAG | 2095 | S | S | 0 | Neutral | rs5745022 |
| 12,133208979,T,C | CTC TC[A/G] GAG | 294 | S | S | 0 | Neutral | rs5745022 |
| 12,133208979,T,C | CTC TC[A/G] GAG | 2057 | S | S | 0 | Neutral | rs5745022 |
| 12,133219831,T,C | AGG GC[A/G] TCC | 1510 | A | A | 0 | Neutral | rs5744944 |
| 12,133219831,T,C | AGG GC[A/G] TCC | 1521 | A | A | 0 | Neutral | rs5744944 |
| 12,133219831,T,C | AGG GC[A/G] TCC | 1483 | A | A | 0 | Neutral | rs5744944 |
| 12,12871099,T,G | GAT G[T/G]C AGC | 109 | V | G | -1.35 | Neutral | rs2066827 |
| 12,12871099,T,G | GAT G[T/G]C AGC | 109 | V | G | -2.06 | Neutral | rs2066827 |
| 12,12871099,T,G | GAT G[T/G]C AGC | 58 | V | G | -1.44 | Neutral | rs2066827 |
| 12,104373829,C,A | TTG AC[C/A] TTC | 125 | T | T | 0 | Neutral | rs75200561 |
| 12,104373829,C,A | TTG AC[C/A] TTC | 129 | T | T | 0 | Neutral | rs75200561 |
| 12,104373829,C,A | TTG AC[C/A] TTC | 104 | T | T | 0 | Neutral | rs75200561 |
| 12,104373829,C,A | TTG AC[C/A] TTC | 129 | T | T | 0 | Neutral | rs75200561 |
| 12,104373829,C,A | TTG AC[C/A] TTC | 33 | T | T | 0 | Neutral | rs75200561 |
| 12,124132621,A,T | CTT TT[A/T] ACC | 63 | L | F | 2.91 | Neutral |  |
| 12,124132621,A,T | CTT TT[A/T] ACC | 8 | L | F | 2.88 | Neutral |  |
| 12,124132621,A,T | CTT TT[A/T] ACC | 63 | L | F | 2.88 | Neutral |  |
| 12,124132621,A,T | CTT TT[A/T] ACC | 54 | L | F | 2.91 | Neutral |  |
| 12,124132621,A,T | CTT TT[A/T] ACC | 104 | L | F | 2.71 | Neutral |  |
| 12,124132621,A,T | CTT TT[A/T] ACC | 63 | L | F | 4.11 | Neutral |  |
| 12,124132621,A,T | CTT TT[A/T] ACC | 104 | L | F | 2.81 | Neutral |  |
| 12,124132621,A,T | CTT TT[A/T] ACC | 119 | L | F | 2.81 | Neutral |  |
| 12,124132621,A,T | CTT TT[A/T] ACC | 94 | L | F | 2.78 | Neutral |  |
| 12,133237618,C,A | ACG CT[G/T] GAA | 999 | L | L | 0 | Neutral |  |
| 12,133237618,C,A | ACG CT[G/T] GAA | 934 | L | L | 0 | Neutral |  |
| 12,133237618,C,A | ACG CT[G/T] GAA | 1010 | L | L | 0 | Neutral |  |
| 12,133237618,C,A | ACG CT[G/T] GAA | 779 | L | L | 0 | Neutral |  |
| 12,133237618,C,A | ACG CT[G/T] GAA | 972 | L | L | 0 | Neutral |  |
| 12,133219831,T,C | AGG GC[A/G] TCC | 1510 | A | A | 0 | Neutral | rs5744944 |
| 12,133219831,T,C | AGG GC[A/G] TCC | 1521 | A | A | 0 | Neutral | rs5744944 |
| 12,133219831,T,C | AGG GC[A/G] TCC | 1483 | A | A | 0 | Neutral | rs5744944 |
| 12,133208979,T,C | CTC TC[A/G] GAG | 2084 | S | S | 0 | Neutral | rs5745022 |
| 12,133208979,T,C | CTC TC[A/G] GAG | 2095 | S | S | 0 | Neutral | rs5745022 |
| 12,133208979,T,C | CTC TC[A/G] GAG | 294 | S | S | 0 | Neutral | rs5745022 |
| 12,133208979,T,C | CTC TC[A/G] GAG | 2057 | S | S | 0 | Neutral | rs5745022 |
| 12,12871099,T,G | GAT G[T/G]C AGC | 109 | V | G | -1.35 | Neutral | rs2066827 |
| 12,12871099,T,G | GAT G[T/G]C AGC | 109 | V | G | -2.06 | Neutral | rs2066827 |
| 12,12871099,T,G | GAT G[T/G]C AGC | 58 | V | G | -1.44 | Neutral | rs2066827 |
| 12,104373829,C,A | TTG AC[C/A] TTC | 125 | T | T | 0 | Neutral | rs75200561 |
| 12,104373829,C,A | TTG AC[C/A] TTC | 129 | T | T | 0 | Neutral | rs75200561 |
| 12,104373829,C,A | TTG AC[C/A] TTC | 104 | T | T | 0 | Neutral | rs75200561 |
| 12,104373829,C,A | TTG AC[C/A] TTC | 129 | T | T | 0 | Neutral | rs75200561 |
| 12,104373829,C,A | TTG AC[C/A] TTC | 33 | T | T | 0 | Neutral | rs75200561 |
| 12,12871860,C,T | GGC [C/T]TC AGA | 193 | L | F | -1.32 | Neutral | rs73281150 |
| 12,12871860,C,T | GGC [C/T]TC AGA | 99 | L | F | -0.31 | Neutral | rs73281150 |
| 12,12871860,C,T | GGC [C/T]TC AGA | 142 | L | F | -1.05 | Neutral | rs73281150 |
| 12,124132621,A,T | CTT TT[A/T] ACC | 63 | L | F | 2.91 | Neutral |  |
| 12,124132621,A,T | CTT TT[A/T] ACC | 8 | L | F | 2.88 | Neutral |  |
| 12,124132621,A,T | CTT TT[A/T] ACC | 63 | L | F | 2.88 | Neutral |  |
| 12,124132621,A,T | CTT TT[A/T] ACC | 54 | L | F | 2.91 | Neutral |  |
| 12,124132621,A,T | CTT TT[A/T] ACC | 104 | L | F | 2.71 | Neutral |  |
| 12,124132621,A,T | CTT TT[A/T] ACC | 63 | L | F | 4.11 | Neutral |  |
| 12,124132621,A,T | CTT TT[A/T] ACC | 104 | L | F | 2.81 | Neutral |  |
| 12,124132621,A,T | CTT TT[A/T] ACC | 119 | L | F | 2.81 | Neutral |  |
| 12,124132621,A,T | CTT TT[A/T] ACC | 94 | L | F | 2.78 | Neutral |  |
| 12,133212582,G,A | TCT [C/T]TG ACA | 1903 | L | L | 0 | Neutral | rs5744990 |
| 12,133212582,G,A | TCT [C/T]TG ACA | 1914 | L | L | 0 | Neutral | rs5744990 |
| 12,133212582,G,A | TCT [C/T]TG ACA | 113 | L | L | 0 | Neutral | rs5744990 |
| 12,133212582,G,A | TCT [C/T]TG ACA | 1876 | L | L | 0 | Neutral | rs5744990 |
| 12,133237618,C,A | ACG CT[G/T] GAA | 999 | L | L | 0 | Neutral |  |
| 12,133237618,C,A | ACG CT[G/T] GAA | 934 | L | L | 0 | Neutral |  |
| 12,133237618,C,A | ACG CT[G/T] GAA | 1010 | L | L | 0 | Neutral |  |
| 12,133237618,C,A | ACG CT[G/T] GAA | 779 | L | L | 0 | Neutral |  |
| 12,133237618,C,A | ACG CT[G/T] GAA | 972 | L | L | 0 | Neutral |  |
| 12,124144395,A,G | CCC CC[A/G] GTT | 205 | P | P | 0 | Neutral | rs1051793 |
| 12,124144395,A,G | CCC CC[A/G] GTT | 132 | P | P | 0 | Neutral | rs1051793 |
| 12,124144395,A,G | CCC [A/G]GT TCA | 198 | S | G | -0.87 | Neutral | rs1051793 |
| 12,124144395,A,G | CCC CC[A/G] GTT | 196 | P | P | 0 | Neutral | rs1051793 |
| 12,124144395,A,G | CCC CC[A/G] GTT | 203 | P | P | 0 | Neutral | rs1051793 |
| 12,124144395,A,G | CCC CC[A/G] GTT | 246 | P | P | 0 | Neutral | rs1051793 |
| 12,104376608,G,A | AGT GA[G/A] GTC | 166 | E | E | 0 | Neutral | rs61937629 |
| 12,104376608,G,A | AGT GA[G/A] GTC | 170 | E | E | 0 | Neutral | rs61937629 |
| 12,104376608,G,A | AGT GA[G/A] GTC | 163 | E | E | 0 | Neutral | rs61937629 |
| 12,104376608,G,A | AGT GA[G/A] GTC | 74 | E | E | 0 | Neutral | rs61937629 |
| 12,104376608,G,A | AGT GA[G/A] GTC | 27 | E | E | 0 | Neutral | rs61937629 |
| 12,118465820,T,C | CAC [T/C]TG TTT | 201 | L | L | 0 | Neutral | rs5745873 |
| 12,118465820,T,C | CAC [T/C]TG TTT | 265 | L | L | 0 | Neutral | rs5745873 |
| 12,118465820,T,C | CAC [T/C]TG TTT | 286 | L | L | 0 | Neutral | rs5745873 |
| 12,133236000,C,T | TCT AC[G/A] TCC | 1052 | T | T | 0 | Neutral | rs5744857 |
| 12,133236000,C,T | TCT AC[G/A] TCC | 987 | T | T | 0 | Neutral | rs5744857 |
| 12,133236000,C,T | TCT AC[G/A] TCC | 1063 | T | T | 0 | Neutral | rs5744857 |
| 12,133236000,C,T | TCT AC[G/A] TCC | 29 | T | T | 0 | Neutral | rs5744857 |
| 12,133236000,C,T | TCT AC[G/A] TCC | 832 | T | T | 0 | Neutral | rs5744857 |
| 12,133236000,C,T | TCT AC[G/A] TCC | 1025 | T | T | 0 | Neutral | rs5744857 |
| 12,4388084,C,G | GCC AC[C/G] GAC | 190 | T | T | 0 | Neutral | rs3217805 |
| 12,4388084,C,G | GCC AC[C/G] GCT | 106 | T | T | 0 | Neutral | rs3217805 |
| 12,104376608,G,A | AGT GA[G/A] GTC | 166 | E | E | 0 | Neutral | rs61937629 |
| 12,104376608,G,A | AGT GA[G/A] GTC | 170 | E | E | 0 | Neutral | rs61937629 |
| 12,104376608,G,A | AGT GA[G/A] GTC | 163 | E | E | 0 | Neutral | rs61937629 |
| 12,104376608,G,A | AGT GA[G/A] GTC | 74 | E | E | 0 | Neutral | rs61937629 |
| 12,104376608,G,A | AGT GA[G/A] GTC | 27 | E | E | 0 | Neutral | rs61937629 |
| 12,124144395,A,G | CCC CC[A/G] GTT | 205 | P | P | 0 | Neutral | rs1051793 |
| 12,124144395,A,G | CCC CC[A/G] GTT | 132 | P | P | 0 | Neutral | rs1051793 |
| 12,124144395,A,G | CCC [A/G]GT TCA | 198 | S | G | -0.87 | Neutral | rs1051793 |
| 12,124144395,A,G | CCC CC[A/G] GTT | 196 | P | P | 0 | Neutral | rs1051793 |
| 12,124144395,A,G | CCC CC[A/G] GTT | 203 | P | P | 0 | Neutral | rs1051793 |
| 12,124144395,A,G | CCC CC[A/G] GTT | 246 | P | P | 0 | Neutral | rs1051793 |
| 12,133208979,T,C | CTC TC[A/G] GAG | 2084 | S | S | 0 | Neutral | rs5745022 |
| 12,133208979,T,C | CTC TC[A/G] GAG | 2095 | S | S | 0 | Neutral | rs5745022 |
| 12,133208979,T,C | CTC TC[A/G] GAG | 294 | S | S | 0 | Neutral | rs5745022 |
| 12,133208979,T,C | CTC TC[A/G] GAG | 2057 | S | S | 0 | Neutral | rs5745022 |
| 12,133253995,G,A | AAT G[C/T]T TTT | 252 | A | V | -0.36 | Neutral | rs5744751 |
| 12,133253995,G,A | AAT G[C/T]T TTT | 187 | A | V | -0.51 | Neutral | rs5744751 |
| 12,133253995,G,A | AAT G[C/T]T TTT | 263 | A | V | -0.36 | Neutral | rs5744751 |
| 12,133253995,G,A | AAT G[C/T]T TTT | 32 | A | V | -0.51 | Neutral | rs5744751 |
| 12,133253995,G,A | AAT G[C/T]T TTT | 252 | A | V | -1.01 | Neutral | rs5744751 |
| 12,133253995,G,A | AAT G[C/T]T TTT | 225 | A | V | -0.36 | Neutral | rs5744751 |
| 12,133212582,G,A | TCT [C/T]TG ACA | 1903 | L | L | 0 | Neutral | rs5744990 |
| 12,133212582,G,A | TCT [C/T]TG ACA | 1914 | L | L | 0 | Neutral | rs5744990 |
| 12,133212582,G,A | TCT [C/T]TG ACA | 113 | L | L | 0 | Neutral | rs5744990 |
| 12,133212582,G,A | TCT [C/T]TG ACA | 1876 | L | L | 0 | Neutral | rs5744990 |
| 12,12871099,T,G | GAT G[T/G]C AGC | 109 | V | G | -1.35 | Neutral | rs2066827 |
| 12,12871099,T,G | GAT G[T/G]C AGC | 109 | V | G | -2.06 | Neutral | rs2066827 |
| 12,12871099,T,G | GAT G[T/G]C AGC | 58 | V | G | -1.44 | Neutral | rs2066827 |
| 12,133242016,C,T | CTC TC[G/A] GCG | 780 | S | S | 0 | Neutral | rs5744822 |
| 12,133242016,C,T | CTC TC[G/A] GCG | 715 | S | S | 0 | Neutral | rs5744822 |
| 12,133242016,C,T | CTC TC[G/A] GCG | 791 | S | S | 0 | Neutral | rs5744822 |
| 12,133242016,C,T | CTC TC[G/A] GCG | 560 | S | S | 0 | Neutral | rs5744822 |
| 12,133242016,C,T | CTC TC[G/A] GCG | 753 | S | S | 0 | Neutral | rs5744822 |
| 13,32906729,A,C | GCA [A/C]AT CAG | 372 | N | H | -0.6 | Neutral | rs144848 |
| 13,32906729,A,C | GCA [A/C]AT CAG | 370 | N | H | -1.55 | Neutral | rs144848 |
| 13,32906729,A,C | GCA [A/C]AT CAG | 372 | N | H | -0.6 | Neutral | rs144848 |
| 13,108861913,A,G | AGT GA[T/C] ATG | 568 | D | D | 0 | Neutral | rs1805386 |
| 13,108861913,A,G | AGT GA[T/C] ATG | 568 | D | D | 0 | Neutral | rs1805386 |
| 13,108861913,A,G | AGT GA[T/C] ATG | 568 | D | D | 0 | Neutral | rs1805386 |
| 13,32912299,T,C | TCT GT[T/C] GTT | 1269 | V | V | 0 | Neutral | rs543304 |
| 13,32912299,T,C | TCT GT[T/C] GTT | 1269 | V | V | 0 | Neutral | rs543304 |
| 13,32972884,A,G | ACA [A/G]TT ACA | 3412 | I | V | -0.09 | Neutral | rs1801426 |
| 13,32972884,A,G | ACA [A/G]TT ACA | 3412 | I | V | -0.09 | Neutral | rs1801426 |
| 13,32972884,A,G | ACA [A/G]TT ACA | 3412 | I | V | -0.09 | Neutral | rs1801426 |
| 13,32972884,A,G | ACA [A/G]TT ACA | 3412 | I | V | -0.09 | Neutral | rs1801426 |
| 13,32953529,A,T | CAG [A/T]TC CAG | 2944 | I | F | -1.12 | Neutral | rs4987047 |
| 13,32953529,A,T | CAG [A/T]TC CAG | 2944 | I | F | -1.12 | Neutral | rs4987047 |
| 13,32906729,A,C | GCA [A/C]AT CAG | 372 | N | H | -0.6 | Neutral | rs144848 |
| 13,32906729,A,C | GCA [A/C]AT CAG | 370 | N | H | -1.55 | Neutral | rs144848 |
| 13,32906729,A,C | GCA [A/C]AT CAG | 372 | N | H | -0.6 | Neutral | rs144848 |
| 13,32929232,A,G | AAA TC[A/G] CAT | 2414 | S | S | 0 | Neutral | rs1799955 |
| 13,32929232,A,G | AAA TC[A/G] CAT | 2414 | S | S | 0 | Neutral | rs1799955 |
| 13,108860884,A,T | TTG AT[T/A] | 911 | I | I | 0 | Neutral |  |
| 13,108860884,A,T | TTG AT[T/A] | 911 | I | I | 0 | Neutral |  |
| 13,108860884,A,T | TTG AT[T/A] | 911 | I | I | 0 | Neutral |  |
| 13,32972884,A,G | ACA [A/G]TT ACA | 3412 | I | V | -0.09 | Neutral | rs1801426 |
| 13,32972884,A,G | ACA [A/G]TT ACA | 3412 | I | V | -0.09 | Neutral | rs1801426 |
| 13,32953529,A,T | CAG [A/T]TC CAG | 2944 | I | F | -1.12 | Neutral | rs4987047 |
| 13,32953529,A,T | CAG [A/T]TC CAG | 2944 | I | F | -1.12 | Neutral | rs4987047 |
| 13,32906729,A,C | GCA [A/C]AT CAG | 372 | N | H | -0.6 | Neutral | rs144848 |
| 13,32906729,A,C | GCA [A/C]AT CAG | 370 | N | H | -1.55 | Neutral | rs144848 |
| 13,32906729,A,C | GCA [A/C]AT CAG | 372 | N | H | -0.6 | Neutral | rs144848 |
| 13,108862810,G,A | TTC TA[C/T] ATA | 269 | Y | Y | 0 | Neutral | rs2232638 |
| 13,108862810,G,A | TTC TA[C/T] ATA | 269 | Y | Y | 0 | Neutral | rs2232638 |
| 13,108862810,G,A | TTC TA[C/T] ATA | 269 | Y | Y | 0 | Neutral | rs2232638 |
| 13,108861092,G,T | AAA G[C/A]C TTG | 842 | A | D | -1.38 | Neutral | rs72660870 |
| 13,108861092,G,T | AAA G[C/A]C TTG | 842 | A | D | -1.38 | Neutral | rs72660870 |
| 13,108861092,G,T | AAA G[C/A]C TTG | 842 | A | D | -1.38 | Neutral | rs72660870 |
| 13,108861048,C,T | TTA [G/A]CT GAG | 857 | A | T | 0.88 | Neutral | rs2232642 |
| 13,108861048,C,T | TTA [G/A]CT GAG | 857 | A | T | 0.88 | Neutral | rs2232642 |
| 13,108861048,C,T | TTA [G/A]CT GAG | 857 | A | T | 0.88 | Neutral | rs2232642 |
| 13,32912679,A,G | GCT C[A/G]A GAA | 1396 | Q | R | -1.99 | Neutral | rs55969723 |
| 13,32912679,A,G | GCT C[A/G]A GAA | 1396 | Q | R | -1.99 | Neutral | rs55969723 |
| 13,32944667,A,C | GAT GT[A/C] ATT | 2820 | V | V | 0 | Neutral | rs9590940 |
| 13,32944667,A,C | GAT GT[A/C] ATT | 2820 | V | V | 0 | Neutral | rs9590940 |
| 13,32912299,T,C | TCT GT[T/C] GTT | 1269 | V | V | 0 | Neutral | rs543304 |
| 13,32912299,T,C | TCT GT[T/C] GTT | 1269 | V | V | 0 | Neutral | rs543304 |
| 13,103504517,T,C | CGC CA[T/C] GGG | 46 | H | H | 0 | Neutral | rs1047768 |
| 13,103504517,T,C | CGC CA[T/C] GGG | 471 | H | H | 0 | Neutral | rs1047768 |
| 13,103504517,T,C | CGC CA[T/C] GGG | 46 | H | H | 0 | Neutral | rs1047768 |
| 13,103520519,G,A | ACC [G/A]AA GGA | 1289 | E | K | -2.2 | Neutral |  |
| 13,103514459,C,T | TTT GA[C/T] GTG | 320 | D | D | 0 | Neutral | rs4150314 |
| 13,103514459,C,T | TTT GA[C/T] GTG | 152 | D | D | 0 | Neutral | rs4150314 |
| 13,103514459,C,T | TTT GA[C/T] GTG | 745 | D | D | 0 | Neutral | rs4150314 |
| 13,103520565,A,G | CTC A[A/G]T GAA | 112 | N | S | -2.33 | Neutral | rs4150342 |
| 13,32911888,A,G | AGA AA[A/G] CCA | 1132 | K | K | 0 | Neutral | rs1801406 |
| 13,32911888,A,G | AGA AA[A/G] CCA | 1132 | K | K | 0 | Neutral | rs1801406 |
| 13,32929232,A,G | AAA TC[A/G] CAT | 2414 | S | S | 0 | Neutral | rs1799955 |
| 13,32929232,A,G | AAA TC[A/G] CAT | 2414 | S | S | 0 | Neutral | rs1799955 |
| 14,75515915,C,G | ACA GT[G/C] TAT | 148 | V | V | 0 | Neutral | rs11556091 |
| 14,75515915,C,G | ACA GT[G/C] TAT | 148 | V | V | 0 | Neutral | rs11556091 |
| 14,75515915,C,G | ACA GT[G/C] TAT | 148 | V | V | 0 | Neutral | rs11556091 |
| 14,75515915,C,G | ACA GT[G/C] TAT | 148 | V | V | 0 | Neutral | rs11556091 |
| 14,75515915,C,G | ACA GT[G/C] TAT | 148 | V | V | 0 | Neutral | rs11556091 |
| 14,20924167,G,C | GAT CA[G/C] AAA | 51 | Q | H | -0.55 | Neutral | rs1048945 |
| 14,20924167,G,C | GAT CA[G/C] AAA | 51 | Q | H | -0.55 | Neutral | rs1048945 |
| 14,20924167,G,C | GAT CA[G/C] AAA | 34 | Q | H | -0.58 | Neutral | rs1048945 |
| 14,20924167,G,C | GAT CA[G/C] AAA | 51 | Q | H | -0.86 | Neutral | rs1048945 |
| 14,20924167,G,C | GAT CA[G/C] AAA | 51 | Q | H | -0.66 | Neutral | rs1048945 |
| 14,20924167,G,C | GAT CA[G/C] AAA | 51 | Q | H | -0.55 | Neutral | rs1048945 |
| 14,20924167,G,C | GAT CA[G/C] AAA | 51 | Q | H | -0.95 | Neutral | rs1048945 |
| 14,20924167,G,C | GAT CA[G/C] AAA | 51 | Q | H | -0.98 | Neutral | rs1048945 |
| 14,20924167,G,C | GAT CA[G/C] AAA | 34 | Q | H | -0.73 | Neutral | rs1048945 |
| 14,20924167,G,C | GAT CA[G/C] AAA | 51 | Q | H | -0.58 | Neutral | rs1048945 |
| 14,38060646,C,T | AGG A[G/A]C CCC | 448 | S | N | -0.7 | Neutral | rs33984772 |
| 14,38060646,C,T | AGG A[G/A]C CCC | 415 | S | N | -0.7 | Neutral | rs33984772 |
| 14,35872926,G,A | CTG GC[C/T] TTC | 102 | A | A | 0 | Neutral | rs1050851 |
| 14,35872926,G,A | CTG GC[C/T] TTC | 12 | A | A | 0 | Neutral | rs1050851 |
| 14,35872926,G,A | CTG GC[C/T] TTC | 102 | A | A | 0 | Neutral | rs1050851 |
| 14,38061742,C,T | GTA [G/A]CC GGC | 83 | A | T | -0.23 | Neutral | rs7144658 |
| 14,38061742,C,T | GTA [G/A]CC GGC | 50 | A | T | -0.3 | Neutral | rs7144658 |
| 14,75483812,T,C | CAG CA[A/G] TCC | 1421 | Q | Q | 0 | Neutral | rs13712 |
| 14,75483812,T,C | CAG CA[A/G] TCC | 1445 | Q | Q | 0 | Neutral | rs13712 |
| 14,75483812,T,C | CAG CA[A/G] TCC | 383 | Q | Q | 0 | Neutral | rs13712 |
| 14,75483812,T,C | CAG CA[A/G] TCC | 116 | Q | Q | 0 | Neutral | rs13712 |
| 14,75483812,T,C | CAG CA[A/G] TCC | 469 | Q | Q | 0 | Neutral | rs13712 |
| 14,75483812,T,C | CAG CA[A/G] TCC | 1267 | Q | Q | 0 | Neutral | rs13712 |
| 14,75483812,T,C | CAG CA[A/G] TCC | 1445 | Q | Q | 0 | Neutral | rs13712 |
| 14,20925154,T,G | GGC GA[T/G] GAG | 148 | D | E | -0.2 | Neutral | rs1130409 |
| 14,20925154,T,G | GGC GA[T/G] GAG | 148 | D | E | -0.2 | Neutral | rs1130409 |
| 14,20925154,T,G | GGC GA[T/G] GAG | 131 | D | E | -0.25 | Neutral | rs1130409 |
| 14,20925154,T,G | GGC GA[T/G] GAG | 148 | D | E | -0.12 | Neutral | rs1130409 |
| 14,20925154,T,G | GGC GA[T/G] GAG | 148 | D | E | -0.03 | Neutral | rs1130409 |
| 14,20925154,T,G | GGC GA[T/G] GAG | 148 | D | E | -0.2 | Neutral | rs1130409 |
| 14,20925154,T,G | GGC GA[T/G] GAG | 131 | D | E | -0.36 | Neutral | rs1130409 |
| 14,75513969,C,T | AAC C[G/A]C TTA | 797 | R | H | 1.47 | Neutral | rs28756991 |
| 14,75513969,C,T | AAC C[G/A]C TTA | 797 | R | H | 1.51 | Neutral | rs28756991 |
| 14,75513969,C,T | AAC C[G/A]C TTA | 797 | R | H | 1.6 | Neutral | rs28756991 |
| 14,75513969,C,T | AAC C[G/A]C TTA | 797 | R | H | 1.51 | Neutral | rs28756991 |
| 14,75515915,C,G | ACA GT[G/C] TAT | 148 | V | V | 0 | Neutral | rs11556091 |
| 14,75515915,C,G | ACA GT[G/C] TAT | 148 | V | V | 0 | Neutral | rs11556091 |
| 14,75515915,C,G | ACA GT[G/C] TAT | 148 | V | V | 0 | Neutral | rs11556091 |
| 14,75515915,C,G | ACA GT[G/C] TAT | 148 | V | V | 0 | Neutral | rs11556091 |
| 14,75515915,C,G | ACA GT[G/C] TAT | 148 | V | V | 0 | Neutral | rs11556091 |
| 14,35872926,G,A | CTG GC[C/T] TTC | 102 | A | A | 0 | Neutral | rs1050851 |
| 14,35872926,G,A | CTG GC[C/T] TTC | 12 | A | A | 0 | Neutral | rs1050851 |
| 14,35872926,G,A | CTG GC[C/T] TTC | 102 | A | A | 0 | Neutral | rs1050851 |
| 14,75515915,C,G | ACA GT[G/C] TAT | 148 | V | V | 0 | Neutral | rs11556091 |
| 14,75515915,C,G | ACA GT[G/C] TAT | 148 | V | V | 0 | Neutral | rs11556091 |
| 14,75515915,C,G | ACA GT[G/C] TAT | 148 | V | V | 0 | Neutral | rs11556091 |
| 14,75515915,C,G | ACA GT[G/C] TAT | 148 | V | V | 0 | Neutral | rs11556091 |
| 14,75515915,C,G | ACA GT[G/C] TAT | 148 | V | V | 0 | Neutral | rs11556091 |
| 14,35872926,G,A | CTG GC[C/T] TTC | 102 | A | A | 0 | Neutral | rs1050851 |
| 14,35872926,G,A | CTG GC[C/T] TTC | 12 | A | A | 0 | Neutral | rs1050851 |
| 14,35872926,G,A | CTG GC[C/T] TTC | 102 | A | A | 0 | Neutral | rs1050851 |
| 14,75515915,C,G | ACA GT[G/C] TAT | 148 | V | V | 0 | Neutral | rs11556091 |
| 14,75515915,C,G | ACA GT[G/C] TAT | 148 | V | V | 0 | Neutral | rs11556091 |
| 14,75515915,C,G | ACA GT[G/C] TAT | 148 | V | V | 0 | Neutral | rs11556091 |
| 14,75515915,C,G | ACA GT[G/C] TAT | 148 | V | V | 0 | Neutral | rs11556091 |
| 14,75515915,C,G | ACA GT[G/C] TAT | 148 | V | V | 0 | Neutral | rs11556091 |
| 14,75483812,T,C | CAG CA[A/G] TCC | 1421 | Q | Q | 0 | Neutral | rs13712 |
| 14,75483812,T,C | CAG CA[A/G] TCC | 1445 | Q | Q | 0 | Neutral | rs13712 |
| 14,75483812,T,C | CAG CA[A/G] TCC | 383 | Q | Q | 0 | Neutral | rs13712 |
| 14,75483812,T,C | CAG CA[A/G] TCC | 116 | Q | Q | 0 | Neutral | rs13712 |
| 14,75483812,T,C | CAG CA[A/G] TCC | 469 | Q | Q | 0 | Neutral | rs13712 |
| 14,75483812,T,C | CAG CA[A/G] TCC | 1267 | Q | Q | 0 | Neutral | rs13712 |
| 14,75483812,T,C | CAG CA[A/G] TCC | 1445 | Q | Q | 0 | Neutral | rs13712 |
| 14,20925154,T,G | GGC GA[T/G] GAG | 148 | D | E | -0.2 | Neutral | rs1130409 |
| 14,20925154,T,G | GGC GA[T/G] GAG | 148 | D | E | -0.2 | Neutral | rs1130409 |
| 14,20925154,T,G | GGC GA[T/G] GAG | 131 | D | E | -0.25 | Neutral | rs1130409 |
| 14,20925154,T,G | GGC GA[T/G] GAG | 148 | D | E | -0.12 | Neutral | rs1130409 |
| 14,20925154,T,G | GGC GA[T/G] GAG | 148 | D | E | -0.03 | Neutral | rs1130409 |
| 14,20925154,T,G | GGC GA[T/G] GAG | 148 | D | E | -0.2 | Neutral | rs1130409 |
| 14,20925154,T,G | GGC GA[T/G] GAG | 131 | D | E | -0.36 | Neutral | rs1130409 |
| 14,38061742,C,T | GTA [G/A]CC GGC | 83 | A | T | -0.23 | Neutral | rs7144658 |
| 14,38061742,C,T | GTA [G/A]CC GGC | 50 | A | T | -0.3 | Neutral | rs7144658 |
| 15,91354521,G,A | CCC [G/A]TA TCT | 1321 | V | I | -0.43 | Neutral | rs7167216 |
| 15,91354521,G,A | CCC [G/A]TA TCT | 508 | V | I | -0.45 | Neutral | rs7167216 |
| 15,91354521,G,A | CCC [G/A]TA TCT | 951 | V | I | -0.43 | Neutral | rs7167216 |
| 15,91354521,G,A | CCC [G/A]TA TCT | 1190 | V | I | -0.2 | Neutral | rs7167216 |
| 15,41001295,C,T | ATA [C/T]GC | 100 | R | C | 1.65 | Neutral | rs148345609 |
| 15,91292697,G,A | AAT [G/A]TT ACC | 67 | V | I | -0.32 | Neutral |  |
| 15,91292697,G,A | AAT [G/A]TT ACC | 67 | V | I | -0.52 | Neutral |  |
| 15,91292697,G,A | AAT [G/A]TT ACC | 67 | V | I | -0.32 | Neutral |  |
| 15,91354521,G,A | CCC [G/A]TA TCT | 1321 | V | I | -0.43 | Neutral | rs7167216 |
| 15,91354521,G,A | CCC [G/A]TA TCT | 508 | V | I | -0.45 | Neutral | rs7167216 |
| 15,91354521,G,A | CCC [G/A]TA TCT | 951 | V | I | -0.43 | Neutral | rs7167216 |
| 15,91354521,G,A | CCC [G/A]TA TCT | 1190 | V | I | -0.2 | Neutral | rs7167216 |
| 15,41001295,C,T | ATA [C/T]GC | 100 | R | C | 1.65 | Neutral | rs148345609 |
| 15,91292697,G,A | AAT [G/A]TT ACC | 67 | V | I | -0.32 | Neutral |  |
| 15,91292697,G,A | AAT [G/A]TT ACC | 67 | V | I | -0.52 | Neutral |  |
| 15,91292697,G,A | AAT [G/A]TT ACC | 67 | V | I | -0.32 | Neutral |  |
| 15,75647311,G,A | CAC T[G/A]C AGA | 370 | C | Y | -0.77 | Neutral |  |
| 15,75647311,G,A | CAC T[G/A]C AGA | 370 | C | Y | -0.77 | Neutral |  |
| 15,75647311,G,A | CAC T[G/A]C AGA | 370 | C | Y | -0.77 | Neutral |  |
| 15,91312789,C,A | CTG A[C/A]T CAG | 843 | T | N | 1.5 | Neutral |  |
| 15,91312789,C,A | CTG A[C/A]T CAG | 30 | T | N | 1.15 | Neutral |  |
| 15,91312789,C,A | CTG A[C/A]T CAG | 496 | T | N | 1.33 | Neutral |  |
| 15,91312789,C,A | CTG A[C/A]T CAG | 843 | T | N | 1.16 | Neutral |  |
| 15,91292917,A,G | TTA G[A/G]A TTT | 140 | E | G | -1.79 | Neutral | rs35886055 |
| 15,91292917,A,G | TTA G[A/G]A TTT | 140 | E | G | -2.06 | Neutral | rs35886055 |
| 15,91292917,A,G | TTA G[A/G]A TTT | 140 | E | G | -1.83 | Neutral | rs35886055 |
| 15,91346923,C,A | AAA GC[C/A] CAA | 1177 | A | A | 0 | Neutral | rs2227934 |
| 15,91346923,C,A | AAA GC[C/A] CAA | 364 | A | A | 0 | Neutral | rs2227934 |
| 15,91346923,C,A | AAA GC[C/A] CAA | 807 | A | A | 0 | Neutral | rs2227934 |
| 15,91354505,C,T | GAG CT[C/T] GAC | 1315 | L | L | 0 | Neutral | rs1063147 |
| 15,91354505,C,T | GAG CT[C/T] GAC | 502 | L | L | 0 | Neutral | rs1063147 |
| 15,91354505,C,T | GAG CT[C/T] GAC | 945 | L | L | 0 | Neutral | rs1063147 |
| 15,91354505,C,T | GAG CT[C/T] GAC | 1184 | L | L | 0 | Neutral | rs1063147 |
| 16,129654,C,T | GAG TT[C/T] TTC | 90 | F | F | 0 | Neutral | rs710081 |
| 16,129654,C,T | GAG TT[C/T] TTC | 85 | F | F | 0 | Neutral | rs710081 |
| 16,129654,C,T | GAG TT[C/T] TTC | 73 | F | F | 0 | Neutral | rs710081 |
| 16,129654,C,T | GAG TT[C/T] TTC | 73 | F | F | 0 | Neutral | rs710081 |
| 16,67227385,G,A | TAC [G/A]TC ACT | 140 | V | I | -0.48 | Neutral |  |
| 16,67227385,G,A | CCT AC[G/A] TCA | 102 | T | T | 0 | Neutral |  |
| 16,14041958,T,C | GAT TC[T/C] GAA | 835 | S | S | 0 | Neutral | rs1799801 |
| 16,14041958,T,C | GAT TC[T/C] GAA | 823 | S | S | 0 | Neutral | rs1799801 |
| 16,14031695,A,G | AAG GA[A/G] GCT | 628 | E | E | 0 | Neutral | rs2020958 |
| 16,14031695,A,G | AAG GA[A/G] GCT | 616 | E | E | 0 | Neutral | rs2020958 |
| 16,67227385,G,A | TAC [G/A]TC ACT | 140 | V | I | -0.48 | Neutral |  |
| 16,67227385,G,A | CCT AC[G/A] TCA | 102 | T | T | 0 | Neutral |  |
| 16,14041958,T,C | GAT TC[T/C] GAA | 835 | S | S | 0 | Neutral | rs1799801 |
| 16,14041958,T,C | GAT TC[T/C] GAA | 823 | S | S | 0 | Neutral | rs1799801 |
| 16,14031695,A,G | AAG GA[A/G] GCT | 628 | E | E | 0 | Neutral | rs2020958 |
| 16,14031695,A,G | AAG GA[A/G] GCT | 616 | E | E | 0 | Neutral | rs2020958 |
| 16,14041958,T,C | GAT TC[T/C] GAA | 835 | S | S | 0 | Neutral | rs1799801 |
| 16,14041958,T,C | GAT TC[T/C] GAA | 823 | S | S | 0 | Neutral | rs1799801 |
| 16,86601367,G,A | GGC AA[G/A] GGC | 142 | K | K | 0 | Neutral | rs41312258 |
| 17,1782957,C,T | ACA TC[C/T] GGG | 352 | S | S | 0 | Neutral | rs2230930 |
| 17,1733399,A,G | GGC CA[A/G] CTG | 4 | Q | Q | 0 | Neutral | rs5030749 |
| 17,1795180,T,C | GAG TC[T/C] GCT | 535 | S | S | 0 | Neutral | rs2230931 |
| 17,1782957,C,T | ACA TC[C/T] GGG | 352 | S | S | 0 | Neutral | rs2230930 |
| 17,1733399,A,G | GGC CA[A/G] CTG | 4 | Q | Q | 0 | Neutral | rs5030749 |
| 17,1782952,A,G | GAC [A/G]CA TCC | 351 | T | A | -0.41 | Neutral | rs5030755 |
| 17,1782957,C,T | ACA TC[C/T] GGG | 352 | S | S | 0 | Neutral | rs2230930 |
| 17,1795180,T,C | GAG TC[T/C] GCT | 535 | S | S | 0 | Neutral | rs2230931 |
| 17,41244936,G,A | GCT C[C/T]G TTT | 871 | P | L | 5.71 | Neutral | rs799917 |
| 17,41244936,G,A | GCT C[C/T]G TTT | 575 | P | L | 5.67 | Neutral | rs799917 |
| 17,41244936,G,A | GCT C[C/T]G TTT | 871 | P | L | 5.71 | Neutral | rs799917 |
| 17,41244936,G,A | GCT C[C/T]G TTT | 871 | P | L | 5.74 | Neutral | rs799917 |
| 17,41244936,G,A | GCT C[C/T]G TTT | 871 | P | L | 5.84 | Neutral | rs799917 |
| 17,41244936,G,A | GCT C[C/T]G TTT | 824 | P | L | 5.7 | Neutral | rs799917 |
| 17,41244936,G,A | GCT C[C/T]G TTT | 871 | P | L | 5.84 | Neutral | rs799917 |
| 17,7579472,G,C | CCC C[C/G]C GTG | 72 | P | R | -0.23 | Neutral | rs1042522 |
| 17,7579472,G,C | CCC C[C/G]C GTG | 72 | P | R | -0.19 | Neutral | rs1042522 |
| 17,7579472,G,C | CCC C[C/G]C GTG | 72 | P | R | -0.23 | Neutral | rs1042522 |
| 17,7579472,G,C | CCC C[C/G]C GTG | 72 | P | R | -0.19 | Neutral | rs1042522 |
| 17,7579472,G,C | CCC C[C/G]C GTG | 72 | P | R | -0.23 | Neutral | rs1042522 |
| 17,7579472,G,C | CCC C[C/G]C GTG | 72 | P | R | -0.19 | Neutral | rs1042522 |
| 17,7579472,G,C | CCC C[C/G]C GTG | 72 | P | R | -0.29 | Neutral | rs1042522 |
| 17,7579472,G,C | CCC C[C/G]C GTG | 72 | P | R | -0.73 | Neutral | rs1042522 |
| 17,7579472,G,C | CCC C[C/G]C GTG | 72 | P | R | -0.24 | Neutral | rs1042522 |
| 17,41245466,G,A | GAC AG[C/T] GAT | 694 | S | S | 0 | Neutral | rs1799949 |
| 17,41245466,G,A | GAC AG[C/T] GAT | 398 | S | S | 0 | Neutral | rs1799949 |
| 17,41245466,G,A | GAC AG[C/T] GAT | 694 | S | S | 0 | Neutral | rs1799949 |
| 17,41245466,G,A | GAC AG[C/T] GAT | 694 | S | S | 0 | Neutral | rs1799949 |
| 17,41245466,G,A | GAC AG[C/T] GAT | 694 | S | S | 0 | Neutral | rs1799949 |
| 17,41245466,G,A | GAC AG[C/T] GAT | 647 | S | S | 0 | Neutral | rs1799949 |
| 17,41245466,G,A | GAC AG[C/T] GAT | 694 | S | S | 0 | Neutral | rs1799949 |
| 17,41245237,A,G | TCA [T/C]TG GTA | 771 | L | L | 0 | Neutral | rs16940 |
| 17,41245237,A,G | TCA [T/C]TG GTA | 475 | L | L | 0 | Neutral | rs16940 |
| 17,41245237,A,G | TCA [T/C]TG GTA | 771 | L | L | 0 | Neutral | rs16940 |
| 17,41245237,A,G | TCA [T/C]TG GTA | 771 | L | L | 0 | Neutral | rs16940 |
| 17,41245237,A,G | TCA [T/C]TG GTA | 771 | L | L | 0 | Neutral | rs16940 |
| 17,41245237,A,G | TCA [T/C]TG GTA | 724 | L | L | 0 | Neutral | rs16940 |
| 17,41245237,A,G | TCA [T/C]TG GTA | 771 | L | L | 0 | Neutral | rs16940 |
| 17,41244000,T,C | CAG A[A/G]A GGA | 1183 | K | R | 0.49 | Neutral | rs16942 |
| 17,41244000,T,C | CAG A[A/G]A GGA | 887 | K | R | 0.29 | Neutral | rs16942 |
| 17,41244000,T,C | CAG A[A/G]A GGA | 1183 | K | R | 0.49 | Neutral | rs16942 |
| 17,41244000,T,C | CAG A[A/G]A GGA | 1183 | K | R | 0.4 | Neutral | rs16942 |
| 17,41244000,T,C | CAG A[A/G]A GGA | 1183 | K | R | 0.42 | Neutral | rs16942 |
| 17,41244000,T,C | CAG A[A/G]A GGA | 1136 | K | R | 0.38 | Neutral | rs16942 |
| 17,41244000,T,C | CAG A[A/G]A GGA | 1183 | K | R | 0.42 | Neutral | rs16942 |
| 17,41226380,G,A | TTG A[C/T]G GAA | 1252 | T | M | 0.49 | Neutral |  |
| 17,41226380,G,A | TTG A[C/T]G GAA | 406 | T | M | 0.66 | Neutral |  |
| 17,41226380,G,A | TTG A[C/T]G GAA | 365 | T | M | 0.82 | Neutral |  |
| 17,41226380,G,A | TTG A[C/T]G GAA | 1548 | T | M | 0.39 | Neutral |  |
| 17,41226380,G,A | TTG A[C/T]G GAA | 397 | T | M | 0.63 | Neutral |  |
| 17,41226380,G,A | TTG A[C/T]G GAA | 1569 | T | M | 0.36 | Neutral |  |
| 17,41226380,G,A | TTG A[C/T]G GAA | 444 | T | M | 0.4 | Neutral |  |
| 17,41226380,G,A | TTG A[C/T]G GAA | 1501 | T | M | 0.4 | Neutral |  |
| 17,41226380,G,A | TTG A[C/T]G GAA | 398 | T | M | 0.74 | Neutral |  |
| 17,41226380,G,A | TTG A[C/T]G GAA | 1570 | T | M | 0.36 | Neutral |  |
| 17,41226380,G,A | TTG A[C/T]G GAA | 319 | T | M | 0.84 | Neutral |  |
| 17,41226380,G,A | TTG A[C/T]G GAA | 444 | T | M | 0.13 | Neutral |  |
| 17,41226380,G,A | TTG A[C/T]G GAA | 444 | T | M | 0.47 | Neutral |  |
| 17,41244936,G,A | GCT C[C/T]G TTT | 871 | P | L | 5.71 | Neutral | rs799917 |
| 17,41244936,G,A | GCT C[C/T]G TTT | 575 | P | L | 5.67 | Neutral | rs799917 |
| 17,41244936,G,A | GCT C[C/T]G TTT | 871 | P | L | 5.71 | Neutral | rs799917 |
| 17,41244936,G,A | GCT C[C/T]G TTT | 871 | P | L | 5.74 | Neutral | rs799917 |
| 17,41244936,G,A | GCT C[C/T]G TTT | 871 | P | L | 5.84 | Neutral | rs799917 |
| 17,41244936,G,A | GCT C[C/T]G TTT | 824 | P | L | 5.7 | Neutral | rs799917 |
| 17,41244936,G,A | GCT C[C/T]G TTT | 871 | P | L | 5.84 | Neutral | rs799917 |
| 17,41234470,A,G | GAC TC[T/C] TCT | 1436 | S | S | 0 | Neutral | rs1060915 |
| 17,41234470,A,G | GAC TC[T/C] TCT | 1140 | S | S | 0 | Neutral | rs1060915 |
| 17,41234470,A,G | GAC TC[T/C] TCT | 294 | S | S | 0 | Neutral | rs1060915 |
| 17,41234470,A,G | GAC TC[T/C] TCT | 1436 | S | S | 0 | Neutral | rs1060915 |
| 17,41234470,A,G | GAC TC[T/C] TCT | 253 | S | S | 0 | Neutral | rs1060915 |
| 17,41234470,A,G | GAC TC[T/C] TCT | 1436 | S | S | 0 | Neutral | rs1060915 |
| 17,41234470,A,G | GAC TC[T/C] TCT | 286 | S | S | 0 | Neutral | rs1060915 |
| 17,41234470,A,G | GAC TC[T/C] TCT | 1436 | S | S | 0 | Neutral | rs1060915 |
| 17,41234470,A,G | GAC TC[T/C] TCT | 333 | S | S | 0 | Neutral | rs1060915 |
| 17,41234470,A,G | GAC TC[T/C] TCT | 201 | S | S | 0 | Neutral | rs1060915 |
| 17,41234470,A,G | GAC TC[T/C] TCT | 208 | S | S | 0 | Neutral | rs1060915 |
| 17,41234470,A,G | GAC TC[T/C] TCT | 1389 | S | S | 0 | Neutral | rs1060915 |
| 17,41234470,A,G | GAC TC[T/C] TCT | 286 | S | S | 0 | Neutral | rs1060915 |
| 17,41234470,A,G | GAC TC[T/C] TCT | 1436 | S | S | 0 | Neutral | rs1060915 |
| 17,41234470,A,G | GAC TC[T/C] TCT | 207 | S | S | 0 | Neutral | rs1060915 |
| 17,41234470,A,G | GAC TC[T/C] TCT | 332 | S | S | 0 | Neutral | rs1060915 |
| 17,41234470,A,G | GAC TC[T/C] TCT | 333 | S | S | 0 | Neutral | rs1060915 |
| 17,41244000,T,C | CAG A[A/G]A GGA | 1183 | K | R | 0.49 | Neutral | rs16942 |
| 17,41244000,T,C | CAG A[A/G]A GGA | 887 | K | R | 0.29 | Neutral | rs16942 |
| 17,41244000,T,C | CAG A[A/G]A GGA | 1183 | K | R | 0.49 | Neutral | rs16942 |
| 17,41244000,T,C | CAG A[A/G]A GGA | 1183 | K | R | 0.4 | Neutral | rs16942 |
| 17,41244000,T,C | CAG A[A/G]A GGA | 1183 | K | R | 0.42 | Neutral | rs16942 |
| 17,41244000,T,C | CAG A[A/G]A GGA | 1136 | K | R | 0.38 | Neutral | rs16942 |
| 17,41244000,T,C | CAG A[A/G]A GGA | 1183 | K | R | 0.42 | Neutral | rs16942 |
| 17,33329049,G,A | TCC C[G/A]C AAG | 867 | R | H | -0.71 | Neutral | rs3136025 |
| 17,33329049,G,A | TCC C[G/A]C AAG | 867 | R | H | -0.65 | Neutral | rs3136025 |
| 17,41245466,G,A | GAC AG[C/T] GAT | 694 | S | S | 0 | Neutral | rs1799949 |
| 17,41245466,G,A | GAC AG[C/T] GAT | 398 | S | S | 0 | Neutral | rs1799949 |
| 17,41245466,G,A | GAC AG[C/T] GAT | 694 | S | S | 0 | Neutral | rs1799949 |
| 17,41245466,G,A | GAC AG[C/T] GAT | 694 | S | S | 0 | Neutral | rs1799949 |
| 17,41245466,G,A | GAC AG[C/T] GAT | 694 | S | S | 0 | Neutral | rs1799949 |
| 17,41245466,G,A | GAC AG[C/T] GAT | 647 | S | S | 0 | Neutral | rs1799949 |
| 17,41245466,G,A | GAC AG[C/T] GAT | 694 | S | S | 0 | Neutral | rs1799949 |
| 17,41245237,A,G | TCA [T/C]TG GTA | 771 | L | L | 0 | Neutral | rs16940 |
| 17,41245237,A,G | TCA [T/C]TG GTA | 475 | L | L | 0 | Neutral | rs16940 |
| 17,41245237,A,G | TCA [T/C]TG GTA | 771 | L | L | 0 | Neutral | rs16940 |
| 17,41245237,A,G | TCA [T/C]TG GTA | 771 | L | L | 0 | Neutral | rs16940 |
| 17,41245237,A,G | TCA [T/C]TG GTA | 771 | L | L | 0 | Neutral | rs16940 |
| 17,41245237,A,G | TCA [T/C]TG GTA | 724 | L | L | 0 | Neutral | rs16940 |
| 17,41245237,A,G | TCA [T/C]TG GTA | 771 | L | L | 0 | Neutral | rs16940 |
| 17,33316549,C,T | TCA AG[C/T] AAA | 252 | S | S | 0 | Neutral |  |
| 17,33316549,C,T | TCA AG[C/T] AAA | 252 | S | S | 0 | Neutral |  |
| 17,41245466,G,A | GAC AG[C/T] GAT | 694 | S | S | 0 | Neutral | rs1799949 |
| 17,41245466,G,A | GAC AG[C/T] GAT | 398 | S | S | 0 | Neutral | rs1799949 |
| 17,41245466,G,A | GAC AG[C/T] GAT | 694 | S | S | 0 | Neutral | rs1799949 |
| 17,41245466,G,A | GAC AG[C/T] GAT | 694 | S | S | 0 | Neutral | rs1799949 |
| 17,41245466,G,A | GAC AG[C/T] GAT | 694 | S | S | 0 | Neutral | rs1799949 |
| 17,41245466,G,A | GAC AG[C/T] GAT | 647 | S | S | 0 | Neutral | rs1799949 |
| 17,41245466,G,A | GAC AG[C/T] GAT | 694 | S | S | 0 | Neutral | rs1799949 |
| 17,41245237,A,G | TCA [T/C]TG GTA | 771 | L | L | 0 | Neutral | rs16940 |
| 17,41245237,A,G | TCA [T/C]TG GTA | 475 | L | L | 0 | Neutral | rs16940 |
| 17,41245237,A,G | TCA [T/C]TG GTA | 771 | L | L | 0 | Neutral | rs16940 |
| 17,41245237,A,G | TCA [T/C]TG GTA | 771 | L | L | 0 | Neutral | rs16940 |
| 17,41245237,A,G | TCA [T/C]TG GTA | 771 | L | L | 0 | Neutral | rs16940 |
| 17,41245237,A,G | TCA [T/C]TG GTA | 724 | L | L | 0 | Neutral | rs16940 |
| 17,41245237,A,G | TCA [T/C]TG GTA | 771 | L | L | 0 | Neutral | rs16940 |
| 17,41244000,T,C | CAG A[A/G]A GGA | 1183 | K | R | 0.49 | Neutral | rs16942 |
| 17,41244000,T,C | CAG A[A/G]A GGA | 887 | K | R | 0.29 | Neutral | rs16942 |
| 17,41244000,T,C | CAG A[A/G]A GGA | 1183 | K | R | 0.49 | Neutral | rs16942 |
| 17,41244000,T,C | CAG A[A/G]A GGA | 1183 | K | R | 0.4 | Neutral | rs16942 |
| 17,41244000,T,C | CAG A[A/G]A GGA | 1183 | K | R | 0.42 | Neutral | rs16942 |
| 17,41244000,T,C | CAG A[A/G]A GGA | 1136 | K | R | 0.38 | Neutral | rs16942 |
| 17,41244000,T,C | CAG A[A/G]A GGA | 1183 | K | R | 0.42 | Neutral | rs16942 |
| 17,41226380,G,A | TTG A[C/T]G GAA | 1252 | T | M | 0.49 | Neutral |  |
| 17,41226380,G,A | TTG A[C/T]G GAA | 406 | T | M | 0.66 | Neutral |  |
| 17,41226380,G,A | TTG A[C/T]G GAA | 365 | T | M | 0.82 | Neutral |  |
| 17,41226380,G,A | TTG A[C/T]G GAA | 1548 | T | M | 0.39 | Neutral |  |
| 17,41226380,G,A | TTG A[C/T]G GAA | 397 | T | M | 0.63 | Neutral |  |
| 17,41226380,G,A | TTG A[C/T]G GAA | 1569 | T | M | 0.36 | Neutral |  |
| 17,41226380,G,A | TTG A[C/T]G GAA | 444 | T | M | 0.4 | Neutral |  |
| 17,41226380,G,A | TTG A[C/T]G GAA | 1501 | T | M | 0.4 | Neutral |  |
| 17,41226380,G,A | TTG A[C/T]G GAA | 398 | T | M | 0.74 | Neutral |  |
| 17,41226380,G,A | TTG A[C/T]G GAA | 1570 | T | M | 0.36 | Neutral |  |
| 17,41226380,G,A | TTG A[C/T]G GAA | 319 | T | M | 0.84 | Neutral |  |
| 17,41226380,G,A | TTG A[C/T]G GAA | 444 | T | M | 0.13 | Neutral |  |
| 17,41226380,G,A | TTG A[C/T]G GAA | 444 | T | M | 0.47 | Neutral |  |
| 17,41244936,G,A | GCT C[C/T]G TTT | 871 | P | L | 5.71 | Neutral | rs799917 |
| 17,41244936,G,A | GCT C[C/T]G TTT | 575 | P | L | 5.67 | Neutral | rs799917 |
| 17,41244936,G,A | GCT C[C/T]G TTT | 871 | P | L | 5.71 | Neutral | rs799917 |
| 17,41244936,G,A | GCT C[C/T]G TTT | 871 | P | L | 5.74 | Neutral | rs799917 |
| 17,41244936,G,A | GCT C[C/T]G TTT | 871 | P | L | 5.84 | Neutral | rs799917 |
| 17,41244936,G,A | GCT C[C/T]G TTT | 824 | P | L | 5.7 | Neutral | rs799917 |
| 17,41244936,G,A | GCT C[C/T]G TTT | 871 | P | L | 5.84 | Neutral | rs799917 |
| 17,41234470,A,G | GAC TC[T/C] TCT | 1436 | S | S | 0 | Neutral | rs1060915 |
| 17,41234470,A,G | GAC TC[T/C] TCT | 1140 | S | S | 0 | Neutral | rs1060915 |
| 17,41234470,A,G | GAC TC[T/C] TCT | 294 | S | S | 0 | Neutral | rs1060915 |
| 17,41234470,A,G | GAC TC[T/C] TCT | 1436 | S | S | 0 | Neutral | rs1060915 |
| 17,41234470,A,G | GAC TC[T/C] TCT | 253 | S | S | 0 | Neutral | rs1060915 |
| 17,41234470,A,G | GAC TC[T/C] TCT | 1436 | S | S | 0 | Neutral | rs1060915 |
| 17,41234470,A,G | GAC TC[T/C] TCT | 286 | S | S | 0 | Neutral | rs1060915 |
| 17,41234470,A,G | GAC TC[T/C] TCT | 1436 | S | S | 0 | Neutral | rs1060915 |
| 17,41234470,A,G | GAC TC[T/C] TCT | 333 | S | S | 0 | Neutral | rs1060915 |
| 17,41234470,A,G | GAC TC[T/C] TCT | 201 | S | S | 0 | Neutral | rs1060915 |
| 17,41234470,A,G | GAC TC[T/C] TCT | 208 | S | S | 0 | Neutral | rs1060915 |
| 17,41234470,A,G | GAC TC[T/C] TCT | 1389 | S | S | 0 | Neutral | rs1060915 |
| 17,41234470,A,G | GAC TC[T/C] TCT | 286 | S | S | 0 | Neutral | rs1060915 |
| 17,41234470,A,G | GAC TC[T/C] TCT | 1436 | S | S | 0 | Neutral | rs1060915 |
| 17,41234470,A,G | GAC TC[T/C] TCT | 207 | S | S | 0 | Neutral | rs1060915 |
| 17,41234470,A,G | GAC TC[T/C] TCT | 332 | S | S | 0 | Neutral | rs1060915 |
| 17,41234470,A,G | GAC TC[T/C] TCT | 333 | S | S | 0 | Neutral | rs1060915 |
| 17,41245466,G,A | GAC AG[C/T] GAT | 694 | S | S | 0 | Neutral | rs1799949 |
| 17,41245466,G,A | GAC AG[C/T] GAT | 398 | S | S | 0 | Neutral | rs1799949 |
| 17,41245466,G,A | GAC AG[C/T] GAT | 694 | S | S | 0 | Neutral | rs1799949 |
| 17,41245466,G,A | GAC AG[C/T] GAT | 694 | S | S | 0 | Neutral | rs1799949 |
| 17,41245466,G,A | GAC AG[C/T] GAT | 694 | S | S | 0 | Neutral | rs1799949 |
| 17,41245466,G,A | GAC AG[C/T] GAT | 647 | S | S | 0 | Neutral | rs1799949 |
| 17,41245466,G,A | GAC AG[C/T] GAT | 694 | S | S | 0 | Neutral | rs1799949 |
| 17,41245237,A,G | TCA [T/C]TG GTA | 771 | L | L | 0 | Neutral | rs16940 |
| 17,41245237,A,G | TCA [T/C]TG GTA | 475 | L | L | 0 | Neutral | rs16940 |
| 17,41245237,A,G | TCA [T/C]TG GTA | 771 | L | L | 0 | Neutral | rs16940 |
| 17,41245237,A,G | TCA [T/C]TG GTA | 771 | L | L | 0 | Neutral | rs16940 |
| 17,41245237,A,G | TCA [T/C]TG GTA | 771 | L | L | 0 | Neutral | rs16940 |
| 17,41245237,A,G | TCA [T/C]TG GTA | 724 | L | L | 0 | Neutral | rs16940 |
| 17,41245237,A,G | TCA [T/C]TG GTA | 771 | L | L | 0 | Neutral | rs16940 |
| 17,33316549,C,T | TCA AG[C/T] AAA | 252 | S | S | 0 | Neutral |  |
| 17,33316549,C,T | TCA AG[C/T] AAA | 252 | S | S | 0 | Neutral |  |
| 17,1782952,A,G | GAC [A/G]CA TCC | 351 | T | A | -0.41 | Neutral | rs5030755 |
| 17,41234470,A,G | GAC TC[T/C] TCT | 1436 | S | S | 0 | Neutral | rs1060915 |
| 17,41234470,A,G | GAC TC[T/C] TCT | 1140 | S | S | 0 | Neutral | rs1060915 |
| 17,41234470,A,G | GAC TC[T/C] TCT | 294 | S | S | 0 | Neutral | rs1060915 |
| 17,41234470,A,G | GAC TC[T/C] TCT | 1436 | S | S | 0 | Neutral | rs1060915 |
| 17,41234470,A,G | GAC TC[T/C] TCT | 253 | S | S | 0 | Neutral | rs1060915 |
| 17,41234470,A,G | GAC TC[T/C] TCT | 1436 | S | S | 0 | Neutral | rs1060915 |
| 17,41234470,A,G | GAC TC[T/C] TCT | 286 | S | S | 0 | Neutral | rs1060915 |
| 17,41234470,A,G | GAC TC[T/C] TCT | 1436 | S | S | 0 | Neutral | rs1060915 |
| 17,41234470,A,G | GAC TC[T/C] TCT | 333 | S | S | 0 | Neutral | rs1060915 |
| 17,41234470,A,G | GAC TC[T/C] TCT | 201 | S | S | 0 | Neutral | rs1060915 |
| 17,41234470,A,G | GAC TC[T/C] TCT | 208 | S | S | 0 | Neutral | rs1060915 |
| 17,41234470,A,G | GAC TC[T/C] TCT | 1389 | S | S | 0 | Neutral | rs1060915 |
| 17,41234470,A,G | GAC TC[T/C] TCT | 286 | S | S | 0 | Neutral | rs1060915 |
| 17,41234470,A,G | GAC TC[T/C] TCT | 1436 | S | S | 0 | Neutral | rs1060915 |
| 17,41234470,A,G | GAC TC[T/C] TCT | 207 | S | S | 0 | Neutral | rs1060915 |
| 17,41234470,A,G | GAC TC[T/C] TCT | 332 | S | S | 0 | Neutral | rs1060915 |
| 17,41234470,A,G | GAC TC[T/C] TCT | 333 | S | S | 0 | Neutral | rs1060915 |
| 17,41245237,A,G | TCA [T/C]TG GTA | 771 | L | L | 0 | Neutral | rs16940 |
| 17,41245237,A,G | TCA [T/C]TG GTA | 475 | L | L | 0 | Neutral | rs16940 |
| 17,41245237,A,G | TCA [T/C]TG GTA | 771 | L | L | 0 | Neutral | rs16940 |
| 17,41245237,A,G | TCA [T/C]TG GTA | 771 | L | L | 0 | Neutral | rs16940 |
| 17,41245237,A,G | TCA [T/C]TG GTA | 771 | L | L | 0 | Neutral | rs16940 |
| 17,41245237,A,G | TCA [T/C]TG GTA | 724 | L | L | 0 | Neutral | rs16940 |
| 17,41245237,A,G | TCA [T/C]TG GTA | 771 | L | L | 0 | Neutral | rs16940 |
| 17,41245466,G,A | GAC AG[C/T] GAT | 694 | S | S | 0 | Neutral | rs1799949 |
| 17,41245466,G,A | GAC AG[C/T] GAT | 398 | S | S | 0 | Neutral | rs1799949 |
| 17,41245466,G,A | GAC AG[C/T] GAT | 694 | S | S | 0 | Neutral | rs1799949 |
| 17,41245466,G,A | GAC AG[C/T] GAT | 694 | S | S | 0 | Neutral | rs1799949 |
| 17,41245466,G,A | GAC AG[C/T] GAT | 694 | S | S | 0 | Neutral | rs1799949 |
| 17,41245466,G,A | GAC AG[C/T] GAT | 647 | S | S | 0 | Neutral | rs1799949 |
| 17,41245466,G,A | GAC AG[C/T] GAT | 694 | S | S | 0 | Neutral | rs1799949 |
| 17,41223094,T,C | CAG [A/G]GT CCA | 1317 | S | G | -0.44 | Neutral | rs1799966 |
| 17,41223094,T,C | CAG [A/G]GT CCA | 471 | S | G | -2.09 | Neutral | rs1799966 |
| 17,41223094,T,C | CAG [A/G]GT CCA | 430 | S | G | -1.95 | Neutral | rs1799966 |
| 17,41223094,T,C | CAG [A/G]GT CCA | 1613 | S | G | -0.51 | Neutral | rs1799966 |
| 17,41223094,T,C | CAG [A/G]GT CCA | 462 | S | G | -1.99 | Neutral | rs1799966 |
| 17,41223094,T,C | CAG [A/G]GT CCA | 1634 | S | G | -0.51 | Neutral | rs1799966 |
| 17,41223094,T,C | CAG [A/G]GT CCA | 509 | S | G | -1.86 | Neutral | rs1799966 |
| 17,41223094,T,C | CAG [A/G]GT CCA | 1566 | S | G | -0.5 | Neutral | rs1799966 |
| 17,41223094,T,C | CAG [A/G]GT CCA | 463 | S | G | -1.83 | Neutral | rs1799966 |
| 17,41223094,T,C | CAG [A/G]GT CCA | 1635 | S | G | -0.51 | Neutral | rs1799966 |
| 17,41223094,T,C | CAG [A/G]GT CCA | 384 | S | G | -1.76 | Neutral | rs1799966 |
| 17,41223094,T,C | CAG [A/G]GT CCA | 509 | S | G | -1.45 | Neutral | rs1799966 |
| 17,41223094,T,C | CAG [A/G]GT CCA | 509 | S | G | -1.99 | Neutral | rs1799966 |
| 17,41244000,T,C | CAG A[A/G]A GGA | 1183 | K | R | 0.49 | Neutral | rs16942 |
| 17,41244000,T,C | CAG A[A/G]A GGA | 887 | K | R | 0.29 | Neutral | rs16942 |
| 17,41244000,T,C | CAG A[A/G]A GGA | 1183 | K | R | 0.49 | Neutral | rs16942 |
| 17,41244000,T,C | CAG A[A/G]A GGA | 1183 | K | R | 0.4 | Neutral | rs16942 |
| 17,41244000,T,C | CAG A[A/G]A GGA | 1183 | K | R | 0.42 | Neutral | rs16942 |
| 17,41244000,T,C | CAG A[A/G]A GGA | 1136 | K | R | 0.38 | Neutral | rs16942 |
| 17,41244000,T,C | CAG A[A/G]A GGA | 1183 | K | R | 0.42 | Neutral | rs16942 |
| 17,41245471,C,T | CAT [G/A]AC AGC | 693 | D | N | 0.03 | Neutral | rs4986850 |
| 17,41245471,C,T | CAT [G/A]AC AGC | 397 | D | N | 0.04 | Neutral | rs4986850 |
| 17,41245471,C,T | CAT [G/A]AC AGC | 693 | D | N | 0.03 | Neutral | rs4986850 |
| 17,41245471,C,T | CAT [G/A]AC AGC | 693 | D | N | 0.03 | Neutral | rs4986850 |
| 17,41245471,C,T | CAT [G/A]AC AGC | 693 | D | N | 0.02 | Neutral | rs4986850 |
| 17,41245471,C,T | CAT [G/A]AC AGC | 646 | D | N | 0.03 | Neutral | rs4986850 |
| 17,41245471,C,T | CAT [G/A]AC AGC | 693 | D | N | 0.02 | Neutral | rs4986850 |
| 17,7579472,G,C | CCC C[C/G]C GTG | 72 | P | R | -0.23 | Neutral | rs1042522 |
| 17,7579472,G,C | CCC C[C/G]C GTG | 72 | P | R | -0.19 | Neutral | rs1042522 |
| 17,7579472,G,C | CCC C[C/G]C GTG | 72 | P | R | -0.23 | Neutral | rs1042522 |
| 17,7579472,G,C | CCC C[C/G]C GTG | 72 | P | R | -0.19 | Neutral | rs1042522 |
| 17,7579472,G,C | CCC C[C/G]C GTG | 72 | P | R | -0.23 | Neutral | rs1042522 |
| 17,7579472,G,C | CCC C[C/G]C GTG | 72 | P | R | -0.19 | Neutral | rs1042522 |
| 17,7579472,G,C | CCC C[C/G]C GTG | 72 | P | R | -0.29 | Neutral | rs1042522 |
| 17,7579472,G,C | CCC C[C/G]C GTG | 72 | P | R | -0.73 | Neutral | rs1042522 |
| 17,7579472,G,C | CCC C[C/G]C GTG | 72 | P | R | -0.24 | Neutral | rs1042522 |
| 17,41244936,G,A | GCT C[C/T]G TTT | 871 | P | L | 5.71 | Neutral | rs799917 |
| 17,41244936,G,A | GCT C[C/T]G TTT | 575 | P | L | 5.67 | Neutral | rs799917 |
| 17,41244936,G,A | GCT C[C/T]G TTT | 871 | P | L | 5.71 | Neutral | rs799917 |
| 17,41244936,G,A | GCT C[C/T]G TTT | 871 | P | L | 5.74 | Neutral | rs799917 |
| 17,41244936,G,A | GCT C[C/T]G TTT | 871 | P | L | 5.84 | Neutral | rs799917 |
| 17,41244936,G,A | GCT C[C/T]G TTT | 824 | P | L | 5.7 | Neutral | rs799917 |
| 17,41244936,G,A | GCT C[C/T]G TTT | 871 | P | L | 5.84 | Neutral | rs799917 |
| 17,1782957,C,T | ACA TC[C/T] GGG | 352 | S | S | 0 | Neutral | rs2230930 |
| 17,41245471,C,T | CAT [G/A]AC AGC | 693 | D | N | 0.03 | Neutral | rs4986850 |
| 17,41245471,C,T | CAT [G/A]AC AGC | 397 | D | N | 0.04 | Neutral | rs4986850 |
| 17,41245471,C,T | CAT [G/A]AC AGC | 693 | D | N | 0.03 | Neutral | rs4986850 |
| 17,41245471,C,T | CAT [G/A]AC AGC | 693 | D | N | 0.03 | Neutral | rs4986850 |
| 17,41245471,C,T | CAT [G/A]AC AGC | 693 | D | N | 0.02 | Neutral | rs4986850 |
| 17,41245471,C,T | CAT [G/A]AC AGC | 646 | D | N | 0.03 | Neutral | rs4986850 |
| 17,41245471,C,T | CAT [G/A]AC AGC | 693 | D | N | 0.02 | Neutral | rs4986850 |
| 17,1782952,A,G | GAC [A/G]CA TCC | 351 | T | A | -0.41 | Neutral | rs5030755 |
| 17,1782952,A,G | GAC [A/G]CA TCC | 351 | T | A | -0.41 | Neutral | rs5030755 |
| 18,20569210,C,G | ACC [C/G]CT GAT | 246 | P | A | -1.12 | Neutral | rs34372414 |
| 18,20569210,C,G | ACC [C/G]CT GAT | 246 | P | A | -1.32 | Neutral | rs34372414 |
| 18,20569210,C,G | ACC [C/G]CT GAT | 246 | P | A | -1.12 | Neutral | rs34372414 |
| 18,20569210,C,G | ACC [C/G]CT GAT | 246 | P | A | -1.12 | Neutral | rs34372414 |
| 18,20569210,C,G | ACC [C/G]CT GAT | 246 | P | A | -1.12 | Neutral | rs34372414 |
| 18,20529656,C,T | GAA AC[C/T] ATT | 76 | T | T | 0 | Neutral | rs139706715 |
| 18,20529656,C,T | GAA AC[C/T] ATT | 76 | T | T | 0 | Neutral | rs139706715 |
| 18,20529656,C,T | GAA AC[C/T] ATT | 76 | T | T | 0 | Neutral | rs139706715 |
| 18,20529656,C,T | GAA AC[C/T] ATT | 76 | T | T | 0 | Neutral | rs139706715 |
| 18,20529656,C,T | GAA AC[C/T] ATT | 76 | T | T | 0 | Neutral | rs139706715 |
| 18,20569210,C,G | ACC [C/G]CT GAT | 246 | P | A | -1.12 | Neutral | rs34372414 |
| 18,20569210,C,G | ACC [C/G]CT GAT | 246 | P | A | -1.32 | Neutral | rs34372414 |
| 18,20569210,C,G | ACC [C/G]CT GAT | 246 | P | A | -1.12 | Neutral | rs34372414 |
| 18,20569210,C,G | ACC [C/G]CT GAT | 246 | P | A | -1.12 | Neutral | rs34372414 |
| 18,20569210,C,G | ACC [C/G]CT GAT | 246 | P | A | -1.12 | Neutral | rs34372414 |
| 18,51820805,G,A | AGA [G/A]CA GGA | 731 | A | T | -0.4 | Neutral | rs8305 |
| 18,51820805,G,A | AGA [G/A]CA GGA | 652 | A | T | -0.42 | Neutral | rs8305 |
| 18,20606115,A,C | ATT A[A/C]G GAA | 869 | K | T | -2.35 | Neutral |  |
| 18,20606115,A,C | ATT A[A/C]G GAA | 874 | K | T | -2.35 | Neutral |  |
| 18,20606115,A,C | TTA [A/C]GG AAG | 837 | R | R | 0 | Neutral |  |
| 18,20606115,A,C | ATT A[A/C]G GAA | 869 | K | T | -2.35 | Neutral |  |
| 18,20606115,A,C | TTA [A/C]GG AAG | 837 | R | R | 0 | Neutral |  |
| 18,20606116,G,A | ATT AA[G/A] GAA | 869 | K | K | 0 | Neutral |  |
| 18,20606116,G,A | ATT AA[G/A] GAA | 874 | K | K | 0 | Neutral |  |
| 18,20606116,G,A | TTA A[G/A]G AAG | 837 | R | K | 0.45 | Neutral |  |
| 18,20606116,G,A | ATT AA[G/A] GAA | 869 | K | K | 0 | Neutral |  |
| 18,20606116,G,A | TTA A[G/A]G AAG | 837 | R | K | 0.5 | Neutral |  |
| 18,20569210,C,G | ACC [C/G]CT GAT | 246 | P | A | -1.12 | Neutral | rs34372414 |
| 18,20569210,C,G | ACC [C/G]CT GAT | 246 | P | A | -1.32 | Neutral | rs34372414 |
| 18,20569210,C,G | ACC [C/G]CT GAT | 246 | P | A | -1.12 | Neutral | rs34372414 |
| 18,20569210,C,G | ACC [C/G]CT GAT | 246 | P | A | -1.12 | Neutral | rs34372414 |
| 18,20569210,C,G | ACC [C/G]CT GAT | 246 | P | A | -1.12 | Neutral | rs34372414 |
| 18,51820805,G,A | AGA [G/A]CA GGA | 731 | A | T | -0.4 | Neutral | rs8305 |
| 18,51820805,G,A | AGA [G/A]CA GGA | 652 | A | T | -0.42 | Neutral | rs8305 |
| 19,48654556,C,T | GAA GT[G/A] GCC | 169 | V | V | 0 | Neutral | rs77648519 |
| 19,48654556,C,T | GAA GT[G/A] GCC | 200 | V | V | 0 | Neutral | rs77648519 |
| 19,48654556,C,T | GAA GT[G/A] GCC | 138 | V | V | 0 | Neutral | rs77648519 |
| 19,48654556,C,T | GAA GT[G/A] GCC | 137 | V | V | 0 | Neutral | rs77648519 |
| 19,48665555,G,A | GAG G[C/T]A TCC | 24 | A | V | 0 | Neutral | rs3730855 |
| 19,48665555,G,A | GAG G[C/T]A TCC | 56 | A | V | -0.14 | Neutral | rs3730855 |
| 19,48665555,G,A | GAG G[C/T]A TCC | 24 | A | V | -0.38 | Neutral | rs3730855 |
| 19,48665555,G,A | GAG G[C/T]A TCC | 24 | A | V | -0.54 | Neutral | rs3730855 |
| 19,44047550,T,C | CCG CA[A/G] GCC | 632 | Q | Q | 0 | Neutral | rs3547 |
| 19,44047550,T,C | CCG CA[A/G] GCC | 646 | Q | Q | 0 | Neutral | rs3547 |
| 19,44047550,T,C | CCG CA[A/G] GCC | 601 | Q | Q | 0 | Neutral | rs3547 |
| 19,45868309,T,G | TGC CG[A/C] TTC | 132 | R | R | 0 | Neutral | rs238406 |
| 19,45868309,T,G | TGC CG[A/C] TTC | 106 | R | R | 0 | Neutral | rs238406 |
| 19,45868309,T,G | TGC CG[A/C] TTC | 132 | R | R | 0 | Neutral | rs238406 |
| 19,45868309,T,G | TGC CG[A/C] TTC | 156 | R | R | 0 | Neutral | rs238406 |
| 19,45868309,T,G | TGC CG[A/C] TTC | 132 | R | R | 0 | Neutral | rs238406 |
| 19,30314666,C,T | AGC AG[C/T] GGG | 405 | S | S | 0 | Neutral | rs7257694 |
| 19,30314666,C,T | AGC AG[C/T] GGG | 362 | S | S | 0 | Neutral | rs7257694 |
| 19,30314666,C,T | AGC AG[C/T] GGG | 390 | S | S | 0 | Neutral | rs7257694 |
| 19,48654553,G,T | GTG GC[C/A] ACA | 170 | A | A | 0 | Neutral | rs20580 |
| 19,48654553,G,T | GTG GC[C/A] ACA | 201 | A | A | 0 | Neutral | rs20580 |
| 19,48654553,G,T | GTG GC[C/A] ACA | 139 | A | A | 0 | Neutral | rs20580 |
| 19,48654553,G,T | GTG GC[C/A] ACA | 138 | A | A | 0 | Neutral | rs20580 |
| 19,48654553,G,T | GTG GC[C/A] ACA | 170 | A | A | 0 | Neutral | rs20580 |
| 19,48654553,G,T | GTG GC[C/A] ACA | 201 | A | A | 0 | Neutral | rs20580 |
| 19,48654553,G,T | GTG GC[C/A] ACA | 139 | A | A | 0 | Neutral | rs20580 |
| 19,48654553,G,T | GTG GC[C/A] ACA | 138 | A | A | 0 | Neutral | rs20580 |
| 19,48654556,C,T | GAA GT[G/A] GCC | 169 | V | V | 0 | Neutral | rs77648519 |
| 19,48654556,C,T | GAA GT[G/A] GCC | 200 | V | V | 0 | Neutral | rs77648519 |
| 19,48654556,C,T | GAA GT[G/A] GCC | 138 | V | V | 0 | Neutral | rs77648519 |
| 19,48654556,C,T | GAA GT[G/A] GCC | 137 | V | V | 0 | Neutral | rs77648519 |
| 19,48654553,G,T | GTG GC[C/A] ACA | 170 | A | A | 0 | Neutral | rs20580 |
| 19,48654553,G,T | GTG GC[C/A] ACA | 201 | A | A | 0 | Neutral | rs20580 |
| 19,48654553,G,T | GTG GC[C/A] ACA | 139 | A | A | 0 | Neutral | rs20580 |
| 19,48654553,G,T | GTG GC[C/A] ACA | 138 | A | A | 0 | Neutral | rs20580 |
| 19,50905074,G,A | TCC C[G/A]C GGC | 120 | R | H | -0.44 | Neutral | rs1726801 |
| 19,50905074,G,A | TCC C[G/A]C GGC | 119 | R | H | -0.37 | Neutral | rs1726801 |
| 19,48621036,C,G | AAG GC[G/C] CTG | 814 | A | A | 0 | Neutral | rs13436 |
| 19,48621036,C,G | AAG GC[G/C] CTG | 746 | A | A | 0 | Neutral | rs13436 |
| 19,48621036,C,G | AAG GC[G/C] CTG | 783 | A | A | 0 | Neutral | rs13436 |
| 19,45868309,T,G | TGC CG[A/C] TTC | 132 | R | R | 0 | Neutral | rs238406 |
| 19,45868309,T,G | TGC CG[A/C] TTC | 106 | R | R | 0 | Neutral | rs238406 |
| 19,45868309,T,G | TGC CG[A/C] TTC | 132 | R | R | 0 | Neutral | rs238406 |
| 19,45868309,T,G | TGC CG[A/C] TTC | 156 | R | R | 0 | Neutral | rs238406 |
| 19,45868309,T,G | TGC CG[A/C] TTC | 132 | R | R | 0 | Neutral | rs238406 |
| 19,50905310,G,A | ATC A[G/A]C CGG | 174 | S | N | -0.92 | Neutral | rs1726803 |
| 19,50905310,G,A | ATC A[G/A]C CGG | 173 | S | N | -1.03 | Neutral | rs1726803 |
| 19,48622427,A,G | AGT GA[T/C] GAG | 802 | D | D | 0 | Neutral | rs20581 |
| 19,48622427,A,G | AGT GA[T/C] GAG | 734 | D | D | 0 | Neutral | rs20581 |
| 19,48622427,A,G | AGT GA[T/C] GAG | 771 | D | D | 0 | Neutral | rs20581 |
| 19,45855524,G,A | GTG GA[C/T] GAG | 661 | D | D | 0 | Neutral | rs1052555 |
| 19,45855524,G,A | GTG GA[C/T] GAG | 687 | D | D | 0 | Neutral | rs1052555 |
| 19,45855524,G,A | GTG GA[C/T] GAG | 633 | D | D | 0 | Neutral | rs1052555 |
| 19,45855524,G,A | GTG GA[C/T] GAG | 711 | D | D | 0 | Neutral | rs1052555 |
| 19,48654556,C,T | GAA GT[G/A] GCC | 169 | V | V | 0 | Neutral | rs77648519 |
| 19,48654556,C,T | GAA GT[G/A] GCC | 200 | V | V | 0 | Neutral | rs77648519 |
| 19,48654556,C,T | GAA GT[G/A] GCC | 138 | V | V | 0 | Neutral | rs77648519 |
| 19,48654556,C,T | GAA GT[G/A] GCC | 137 | V | V | 0 | Neutral | rs77648519 |
| 19,48654553,G,T | GTG GC[C/A] ACA | 170 | A | A | 0 | Neutral | rs20580 |
| 19,48654553,G,T | GTG GC[C/A] ACA | 201 | A | A | 0 | Neutral | rs20580 |
| 19,48654553,G,T | GTG GC[C/A] ACA | 139 | A | A | 0 | Neutral | rs20580 |
| 19,48654553,G,T | GTG GC[C/A] ACA | 138 | A | A | 0 | Neutral | rs20580 |
| 19,50905074,G,A | TCC C[G/A]C GGC | 120 | R | H | -0.44 | Neutral | rs1726801 |
| 19,50905074,G,A | TCC C[G/A]C GGC | 119 | R | H | -0.37 | Neutral | rs1726801 |
| 19,48621036,C,G | AAG GC[G/C] CTG | 814 | A | A | 0 | Neutral | rs13436 |
| 19,48621036,C,G | AAG GC[G/C] CTG | 746 | A | A | 0 | Neutral | rs13436 |
| 19,48621036,C,G | AAG GC[G/C] CTG | 783 | A | A | 0 | Neutral | rs13436 |
| 19,45868309,T,G | TGC CG[A/C] TTC | 132 | R | R | 0 | Neutral | rs238406 |
| 19,45868309,T,G | TGC CG[A/C] TTC | 106 | R | R | 0 | Neutral | rs238406 |
| 19,45868309,T,G | TGC CG[A/C] TTC | 132 | R | R | 0 | Neutral | rs238406 |
| 19,45868309,T,G | TGC CG[A/C] TTC | 156 | R | R | 0 | Neutral | rs238406 |
| 19,45868309,T,G | TGC CG[A/C] TTC | 132 | R | R | 0 | Neutral | rs238406 |
| 19,44057227,T,C | GAC CC[A/G] GCA | 206 | P | P | 0 | Neutral | rs915927 |
| 19,44057227,T,C | GAC CC[A/G] GCA | 220 | P | P | 0 | Neutral | rs915927 |
| 19,44057227,T,C | GAC CC[A/G] GCA | 175 | P | P | 0 | Neutral | rs915927 |
| 19,44057227,T,C | GAC CC[A/G] GCA | 206 | P | P | 0 | Neutral | rs915927 |
| 19,45854919,T,G | CTG [A/C]AG AGG | 701 | K | Q | -0.81 | Neutral | rs13181 |
| 19,45854919,T,G | CTG [A/C]AG AGG | 727 | K | Q | -0.83 | Neutral | rs13181 |
| 19,45854919,T,G | CTG [A/C]AG AGG | 673 | K | Q | -0.62 | Neutral | rs13181 |
| 19,45854919,T,G | CTG [A/C]AG AGG | 751 | K | Q | -0.94 | Neutral | rs13181 |
| 19,45854919,T,G | CTG [A/C]AG AGG | 701 | K | Q | -0.81 | Neutral | rs13181 |
| 19,45854919,T,G | CTG [A/C]AG AGG | 727 | K | Q | -0.83 | Neutral | rs13181 |
| 19,45854919,T,G | CTG [A/C]AG AGG | 673 | K | Q | -0.62 | Neutral | rs13181 |
| 19,45854919,T,G | CTG [A/C]AG AGG | 751 | K | Q | -0.94 | Neutral | rs13181 |
| 19,45855524,G,A | GTG GA[C/T] GAG | 661 | D | D | 0 | Neutral | rs1052555 |
| 19,45855524,G,A | GTG GA[C/T] GAG | 687 | D | D | 0 | Neutral | rs1052555 |
| 19,45855524,G,A | GTG GA[C/T] GAG | 633 | D | D | 0 | Neutral | rs1052555 |
| 19,45855524,G,A | GTG GA[C/T] GAG | 711 | D | D | 0 | Neutral | rs1052555 |
| 19,45855524,G,A | GTG GA[C/T] GAG | 661 | D | D | 0 | Neutral | rs1052555 |
| 19,45855524,G,A | GTG GA[C/T] GAG | 687 | D | D | 0 | Neutral | rs1052555 |
| 19,45855524,G,A | GTG GA[C/T] GAG | 633 | D | D | 0 | Neutral | rs1052555 |
| 19,45855524,G,A | GTG GA[C/T] GAG | 711 | D | D | 0 | Neutral | rs1052555 |
| 19,48654553,G,T | GTG GC[C/A] ACA | 170 | A | A | 0 | Neutral | rs20580 |
| 19,48654553,G,T | GTG GC[C/A] ACA | 201 | A | A | 0 | Neutral | rs20580 |
| 19,48654553,G,T | GTG GC[C/A] ACA | 139 | A | A | 0 | Neutral | rs20580 |
| 19,48654553,G,T | GTG GC[C/A] ACA | 138 | A | A | 0 | Neutral | rs20580 |
| 19,44047550,T,C | CCG CA[A/G] GCC | 632 | Q | Q | 0 | Neutral | rs3547 |
| 19,44047550,T,C | CCG CA[A/G] GCC | 646 | Q | Q | 0 | Neutral | rs3547 |
| 19,44047550,T,C | CCG CA[A/G] GCC | 601 | Q | Q | 0 | Neutral | rs3547 |
| 2,48010488,G,A | CCC G[G/A]G GCC | 39 | G | E | -0.28 | Neutral | rs1042821 |
| 2,48010488,G,A | CCC G[G/A]G GCC | 39 | G | E | 0.17 | Neutral | rs1042821 |
| 2,48010488,G,A | CCC G[G/A]G GCC | 39 | G | E | 0.31 | Neutral | rs1042821 |
| 2,48010488,G,A | CCC CG[G/A] GGC | 38 | R | R | 0 | Neutral | rs1042821 |
| 2,48010488,G,A | CCC G[G/A]G GCC | 39 | G | E | 0.13 | Neutral | rs1042821 |
| 2,73519366,G,A | CAG G[C/T]C GCC | 227 | A | V | -0.63 | Neutral | rs138743995 |
| 2,73519366,G,A | CAG G[C/T]C GCC | 330 | A | V | -0.5 | Neutral | rs138743995 |
| 2,73518735,A,G | AAG CC[T/C] TTT | 437 | P | P | 0 | Neutral | rs35362064 |
| 2,73518735,A,G | AAG CC[T/C] TTT | 540 | P | P | 0 | Neutral | rs35362064 |
| 2,48018081,A,G | TCA CC[A/G] GGA | 92 | P | P | 0 | Neutral | rs1800932 |
| 2,48018081,A,G | TCA CC[A/G] GGA | 92 | P | P | 0 | Neutral | rs1800932 |
| 2,48018081,A,G | TCA CC[A/G] GGA | 92 | P | P | 0 | Neutral | rs1800932 |
| 2,48018081,A,G | TCA CC[A/G] GGA | 90 | P | P | 0 | Neutral | rs1800932 |
| 2,48023115,T,C | GCA GA[T/C] GAA | 180 | D | D | 0 | Neutral | rs1800935 |
| 2,48023115,T,C | GCA GA[T/C] GAA | 81 | D | D | 0 | Neutral | rs1800935 |
| 2,48023115,T,C | GCA GA[T/C] GAA | 81 | D | D | 0 | Neutral | rs1800935 |
| 2,48023115,T,C | GCA GA[T/C] GAA | 81 | D | D | 0 | Neutral | rs1800935 |
| 2,48023115,T,C | GCA GA[T/C] GAA | 180 | D | D | 0 | Neutral | rs1800935 |
| 2,48023115,T,C | GCA GA[T/C] GAA | 178 | D | D | 0 | Neutral | rs1800935 |
| 2,48010488,G,A | CCC G[G/A]G GCC | 39 | G | E | -0.28 | Neutral | rs1042821 |
| 2,48010488,G,A | CCC G[G/A]G GCC | 39 | G | E | 0.17 | Neutral | rs1042821 |
| 2,48010488,G,A | CCC G[G/A]G GCC | 39 | G | E | 0.31 | Neutral | rs1042821 |
| 2,48010488,G,A | CCC CG[G/A] GGC | 38 | R | R | 0 | Neutral | rs1042821 |
| 2,48010488,G,A | CCC G[G/A]G GCC | 39 | G | E | 0.13 | Neutral | rs1042821 |
| 2,48010488,G,A | CCC G[G/A]G GCC | 39 | G | E | -0.28 | Neutral | rs1042821 |
| 2,48010488,G,A | CCC G[G/A]G GCC | 39 | G | E | 0.17 | Neutral | rs1042821 |
| 2,48010488,G,A | CCC G[G/A]G GCC | 39 | G | E | 0.31 | Neutral | rs1042821 |
| 2,48010488,G,A | CCC CG[G/A] GGC | 38 | R | R | 0 | Neutral | rs1042821 |
| 2,48010488,G,A | CCC G[G/A]G GCC | 39 | G | E | 0.13 | Neutral | rs1042821 |
| 2,48033742,G,A | CAT A[G/A]A AAA | 1318 | R | K | 0.14 | Neutral |  |
| 2,48033742,G,A | CAT A[G/A]A AAA | 1016 | R | K | 0.14 | Neutral |  |
| 2,48033742,G,A | CAT A[G/A]A AAA | 282 | R | K | -0.07 | Neutral |  |
| 2,48033742,G,A | CAT A[G/A]A AAA | 1188 | R | K | 0.14 | Neutral |  |
| 2,216990753,C,T | AAA T[C/T]G ATT | 266 | S | L | -1.58 | Neutral |  |
| 2,216990753,C,T | AAA T[C/T]G ATT | 266 | S | L | -1.58 | Neutral |  |
| 2,217012901,A,G | ACA AC[A/G] AAA | 524 | T | T | 0 | Neutral | rs207906 |
| 2,217012901,A,G | ACA AC[A/G] AAA | 524 | T | T | 0 | Neutral | rs207906 |
| 2,48018081,A,G | TCA CC[A/G] GGA | 92 | P | P | 0 | Neutral | rs1800932 |
| 2,48018081,A,G | TCA CC[A/G] GGA | 92 | P | P | 0 | Neutral | rs1800932 |
| 2,48018081,A,G | TCA CC[A/G] GGA | 92 | P | P | 0 | Neutral | rs1800932 |
| 2,48018081,A,G | TCA CC[A/G] GGA | 90 | P | P | 0 | Neutral | rs1800932 |
| 2,48027375,T,C | ACA AA[T/C] GGT | 751 | N | N | 0 | Neutral | rs2020913 |
| 2,48027375,T,C | ACA AA[T/C] GGT | 449 | N | N | 0 | Neutral | rs2020913 |
| 2,48027375,T,C | ACA AA[T/C] GGT | 749 | N | N | 0 | Neutral | rs2020913 |
| 2,48027375,T,C | ACA AA[T/C] GGT | 621 | N | N | 0 | Neutral | rs2020913 |
| 2,73519366,G,A | CAG G[C/T]C GCC | 227 | A | V | -0.63 | Neutral | rs138743995 |
| 2,73519366,G,A | CAG G[C/T]C GCC | 330 | A | V | -0.5 | Neutral | rs138743995 |
| 2,73518735,A,G | AAG CC[T/C] TTT | 437 | P | P | 0 | Neutral | rs35362064 |
| 2,73518735,A,G | AAG CC[T/C] TTT | 540 | P | P | 0 | Neutral | rs35362064 |
| 2,48018081,A,G | TCA CC[A/G] GGA | 92 | P | P | 0 | Neutral | rs1800932 |
| 2,48018081,A,G | TCA CC[A/G] GGA | 92 | P | P | 0 | Neutral | rs1800932 |
| 2,48018081,A,G | TCA CC[A/G] GGA | 92 | P | P | 0 | Neutral | rs1800932 |
| 2,48018081,A,G | TCA CC[A/G] GGA | 90 | P | P | 0 | Neutral | rs1800932 |
| 2,48023115,T,C | GCA GA[T/C] GAA | 180 | D | D | 0 | Neutral | rs1800935 |
| 2,48023115,T,C | GCA GA[T/C] GAA | 81 | D | D | 0 | Neutral | rs1800935 |
| 2,48023115,T,C | GCA GA[T/C] GAA | 81 | D | D | 0 | Neutral | rs1800935 |
| 2,48023115,T,C | GCA GA[T/C] GAA | 81 | D | D | 0 | Neutral | rs1800935 |
| 2,48023115,T,C | GCA GA[T/C] GAA | 180 | D | D | 0 | Neutral | rs1800935 |
| 2,48023115,T,C | GCA GA[T/C] GAA | 178 | D | D | 0 | Neutral | rs1800935 |
| 2,217012901,A,G | ACA AC[A/G] AAA | 524 | T | T | 0 | Neutral | rs207906 |
| 2,217012901,A,G | ACA AC[A/G] AAA | 524 | T | T | 0 | Neutral | rs207906 |
| 2,217012901,A,G | ACA AC[A/G] AAA | 524 | T | T | 0 | Neutral | rs207906 |
| 2,217012901,A,G | ACA AC[A/G] AAA | 524 | T | T | 0 | Neutral | rs207906 |
| 2,217012901,A,G | ACA AC[A/G] AAA | 524 | T | T | 0 | Neutral | rs207906 |
| 2,217012901,A,G | ACA AC[A/G] AAA | 524 | T | T | 0 | Neutral | rs207906 |
| 2,48018081,A,G | TCA CC[A/G] GGA | 92 | P | P | 0 | Neutral | rs1800932 |
| 2,48018081,A,G | TCA CC[A/G] GGA | 92 | P | P | 0 | Neutral | rs1800932 |
| 2,48018081,A,G | TCA CC[A/G] GGA | 92 | P | P | 0 | Neutral | rs1800932 |
| 2,48018081,A,G | TCA CC[A/G] GGA | 90 | P | P | 0 | Neutral | rs1800932 |
| 2,48023115,T,C | GCA GA[T/C] GAA | 180 | D | D | 0 | Neutral | rs1800935 |
| 2,48023115,T,C | GCA GA[T/C] GAA | 81 | D | D | 0 | Neutral | rs1800935 |
| 2,48023115,T,C | GCA GA[T/C] GAA | 81 | D | D | 0 | Neutral | rs1800935 |
| 2,48023115,T,C | GCA GA[T/C] GAA | 81 | D | D | 0 | Neutral | rs1800935 |
| 2,48023115,T,C | GCA GA[T/C] GAA | 180 | D | D | 0 | Neutral | rs1800935 |
| 2,48023115,T,C | GCA GA[T/C] GAA | 178 | D | D | 0 | Neutral | rs1800935 |
| 2,48018081,A,G | TCA CC[A/G] GGA | 92 | P | P | 0 | Neutral | rs1800932 |
| 2,48018081,A,G | TCA CC[A/G] GGA | 92 | P | P | 0 | Neutral | rs1800932 |
| 2,48018081,A,G | TCA CC[A/G] GGA | 92 | P | P | 0 | Neutral | rs1800932 |
| 2,48018081,A,G | TCA CC[A/G] GGA | 90 | P | P | 0 | Neutral | rs1800932 |
| 20,10639222,G,A | GGC TG[C/T] AAT | 196 | C | C | 0 | Neutral | rs1801138 |
| 20,10639222,G,A | GGC TG[C/T] AAT | 37 | C | C | 0 | Neutral | rs1801138 |
| 20,10622258,G,A | CTG GA[C/T] GAC | 922 | D | D | 0 | Neutral | rs2229895 |
| 20,10622258,G,A | CTG GA[C/T] GAC | 763 | D | D | 0 | Neutral | rs2229895 |
| 20,10653469,C,T | GCC GG[G/A] GGG | 89 | G | G | 0 | Neutral | rs1051415 |
| 20,10637057,T,C | CTC CC[A/G] GGT | 248 | P | P | 0 | Neutral | rs10485741 |
| 20,10637057,T,C | CTC CC[A/G] GGT | 89 | P | P | 0 | Neutral | rs10485741 |
| 20,10625804,T,G | GGA AC[A/C] ACC | 738 | T | T | 0 | Neutral | rs1801140 |
| 20,10625804,T,G | GGA AC[A/C] ACC | 579 | T | T | 0 | Neutral | rs1801140 |
| 20,10639222,G,A | GGC TG[C/T] AAT | 196 | C | C | 0 | Neutral | rs1801138 |
| 20,10639222,G,A | GGC TG[C/T] AAT | 37 | C | C | 0 | Neutral | rs1801138 |
| 20,10620386,A,G | GAT TA[T/C] GAG | 1139 | Y | Y | 0 | Neutral | rs1051419 |
| 20,10620386,A,G | GAT TA[T/C] GAG | 980 | Y | Y | 0 | Neutral | rs1051419 |
| 20,10639222,G,A | GGC TG[C/T] AAT | 196 | C | C | 0 | Neutral | rs1801138 |
| 20,10639222,G,A | GGC TG[C/T] AAT | 37 | C | C | 0 | Neutral | rs1801138 |
| 20,10620386,A,G | GAT TA[T/C] GAG | 1139 | Y | Y | 0 | Neutral | rs1051419 |
| 20,10620386,A,G | GAT TA[T/C] GAG | 980 | Y | Y | 0 | Neutral | rs1051419 |
| 20,10620386,A,G | GAT TA[T/C] GAG | 1139 | Y | Y | 0 | Neutral | rs1051419 |
| 20,10620386,A,G | GAT TA[T/C] GAG | 980 | Y | Y | 0 | Neutral | rs1051419 |
| 20,10625804,T,G | GGA AC[A/C] ACC | 738 | T | T | 0 | Neutral | rs1801140 |
| 20,10625804,T,G | GGA AC[A/C] ACC | 579 | T | T | 0 | Neutral | rs1801140 |
| 20,10625804,T,G | GGA AC[A/C] ACC | 738 | T | T | 0 | Neutral | rs1801140 |
| 20,10625804,T,G | GGA AC[A/C] ACC | 579 | T | T | 0 | Neutral | rs1801140 |
| 20,32264675,C,T | TCC [G/A]GC CTC | 393 | G | S | 0.49 | Neutral | rs3213176 |
| 20,10633237,G,A | CAG TA[C/T] GGC | 255 | Y | Y | 0 | Neutral | rs1131695 |
| 20,10633237,G,A | CAG TA[C/T] GGC | 96 | Y | Y | 0 | Neutral | rs1131695 |
| 21,42879909,C,A | GGA G[G/T]T GAA | 8 | G | V | 0.22 | Neutral | rs75603675 |
| 21,42866296,T,C | GGG AC[A/G] GTG | 75 | T | T | 0 | Neutral | rs3787950 |
| 21,42866296,T,C | GGG AC[A/G] GTG | 112 | T | T | 0 | Neutral | rs3787950 |
| 21,42866296,T,C | GGG AC[A/G] GTG | 75 | T | T | 0 | Neutral | rs3787950 |
| 21,42866296,T,C | GGG AC[A/G] GTG | 75 | T | T | 0 | Neutral | rs3787950 |
| 21,42866296,T,C | GGG AC[A/G] GTG | 75 | T | T | 0 | Neutral | rs3787950 |
| 21,42866296,T,C | GGG AC[A/G] GTG | 75 | T | T | 0 | Neutral | rs3787950 |
| 21,42852497,C,T | CAG [G/A]TG TAC | 160 | V | M | -1.89 | Neutral | rs12329760 |
| 21,42852497,C,T | CAG [G/A]TG TAC | 197 | V | M | -1.89 | Neutral | rs12329760 |
| 21,42852497,C,T | CAG [G/A]TG TAC | 160 | V | M | -1.89 | Neutral | rs12329760 |
| 21,42852497,C,T | CAG [G/A]TG TAC | 160 | V | M | -1.89 | Neutral | rs12329760 |
| 21,42852497,C,T | CAG [G/A]TG TAC | 120 | V | M | -1.78 | Neutral | rs12329760 |
| 21,42840394,C,T | TAT [G/A]TC TAT | 415 | V | I | -0.27 | Neutral | rs148125094 |
| 21,42840394,C,T | TAT [G/A]TC TAT | 452 | V | I | -0.27 | Neutral | rs148125094 |
| 21,42840394,C,T | TAT [G/A]TC TAT | 415 | V | I | -0.27 | Neutral | rs148125094 |
| 21,42840394,C,T | TAT [G/A]TC TAT | 415 | V | I | -0.27 | Neutral | rs148125094 |
| 21,42845374,G,A | GGC GG[C/T] GAG | 259 | G | G | 0 | Neutral | rs2298659 |
| 21,42845374,G,A | GGC GG[C/T] GAG | 296 | G | G | 0 | Neutral | rs2298659 |
| 21,42845374,G,A | GGC GG[C/T] GAG | 259 | G | G | 0 | Neutral | rs2298659 |
| 21,42845374,G,A | GGC GG[C/T] GAG | 259 | G | G | 0 | Neutral | rs2298659 |
| 21,42845374,G,A | GGC GG[C/T] | 219 | G | G | 0 | Neutral | rs2298659 |
| 21,42852497,C,T | CAG [G/A]TG TAC | 160 | V | M | -1.89 | Neutral | rs12329760 |
| 21,42852497,C,T | CAG [G/A]TG TAC | 197 | V | M | -1.89 | Neutral | rs12329760 |
| 21,42852497,C,T | CAG [G/A]TG TAC | 160 | V | M | -1.89 | Neutral | rs12329760 |
| 21,42852497,C,T | CAG [G/A]TG TAC | 160 | V | M | -1.89 | Neutral | rs12329760 |
| 21,42852497,C,T | CAG [G/A]TG TAC | 120 | V | M | -1.78 | Neutral | rs12329760 |
| 21,42866296,T,C | GGG AC[A/G] GTG | 75 | T | T | 0 | Neutral | rs3787950 |
| 21,42866296,T,C | GGG AC[A/G] GTG | 112 | T | T | 0 | Neutral | rs3787950 |
| 21,42866296,T,C | GGG AC[A/G] GTG | 75 | T | T | 0 | Neutral | rs3787950 |
| 21,42866296,T,C | GGG AC[A/G] GTG | 75 | T | T | 0 | Neutral | rs3787950 |
| 21,42866296,T,C | GGG AC[A/G] GTG | 75 | T | T | 0 | Neutral | rs3787950 |
| 21,42866296,T,C | GGG AC[A/G] GTG | 75 | T | T | 0 | Neutral | rs3787950 |
| 21,42866296,T,C | GGG AC[A/G] GTG | 75 | T | T | 0 | Neutral | rs3787950 |
| 21,42866296,T,C | GGG AC[A/G] GTG | 112 | T | T | 0 | Neutral | rs3787950 |
| 21,42866296,T,C | GGG AC[A/G] GTG | 75 | T | T | 0 | Neutral | rs3787950 |
| 21,42866296,T,C | GGG AC[A/G] GTG | 75 | T | T | 0 | Neutral | rs3787950 |
| 21,42866296,T,C | GGG AC[A/G] GTG | 75 | T | T | 0 | Neutral | rs3787950 |
| 21,42866296,T,C | GGG AC[A/G] GTG | 75 | T | T | 0 | Neutral | rs3787950 |
| 21,42866296,T,C | GGG AC[A/G] GTG | 75 | T | T | 0 | Neutral | rs3787950 |
| 21,42866296,T,C | GGG AC[A/G] GTG | 112 | T | T | 0 | Neutral | rs3787950 |
| 21,42866296,T,C | GGG AC[A/G] GTG | 75 | T | T | 0 | Neutral | rs3787950 |
| 21,42866296,T,C | GGG AC[A/G] GTG | 75 | T | T | 0 | Neutral | rs3787950 |
| 21,42866296,T,C | GGG AC[A/G] GTG | 75 | T | T | 0 | Neutral | rs3787950 |
| 21,42866296,T,C | GGG AC[A/G] GTG | 75 | T | T | 0 | Neutral | rs3787950 |
| 21,42842581,C,T | GAG [G/A]AG AAA | 389 | E | K | -2.39 | Neutral |  |
| 21,42842581,C,T | GAG [G/A]AG AAA | 426 | E | K | -2.39 | Neutral |  |
| 21,42842581,C,T | GAG [G/A]AG AAA | 389 | E | K | -2.39 | Neutral |  |
| 21,42842581,C,T | GAG [G/A]AG AAA | 389 | E | K | -2.39 | Neutral |  |
| 21,42852497,C,T | CAG [G/A]TG TAC | 160 | V | M | -1.89 | Neutral | rs12329760 |
| 21,42852497,C,T | CAG [G/A]TG TAC | 197 | V | M | -1.89 | Neutral | rs12329760 |
| 21,42852497,C,T | CAG [G/A]TG TAC | 160 | V | M | -1.89 | Neutral | rs12329760 |
| 21,42852497,C,T | CAG [G/A]TG TAC | 160 | V | M | -1.89 | Neutral | rs12329760 |
| 21,42852497,C,T | CAG [G/A]TG TAC | 120 | V | M | -1.78 | Neutral | rs12329760 |
| 21,42866398,C,T | CAT CC[G/A] GCT | 41 | P | P | 0 | Neutral | rs143203874 |
| 21,42866398,C,T | CAT CC[G/A] GCT | 78 | P | P | 0 | Neutral | rs143203874 |
| 21,42866398,C,T | CAT CC[G/A] GCT | 41 | P | P | 0 | Neutral | rs143203874 |
| 21,42866398,C,T | CAT CC[G/A] GCT | 41 | P | P | 0 | Neutral | rs143203874 |
| 21,42866398,C,T | CAT CC[G/A] GCT | 41 | P | P | 0 | Neutral | rs143203874 |
| 21,42866398,C,T | CAT CC[G/A] GCT | 41 | P | P | 0 | Neutral | rs143203874 |
| 21,42852497,C,T | CAG [G/A]TG TAC | 160 | V | M | -1.89 | Neutral | rs12329760 |
| 21,42852497,C,T | CAG [G/A]TG TAC | 197 | V | M | -1.89 | Neutral | rs12329760 |
| 21,42852497,C,T | CAG [G/A]TG TAC | 160 | V | M | -1.89 | Neutral | rs12329760 |
| 21,42852497,C,T | CAG [G/A]TG TAC | 160 | V | M | -1.89 | Neutral | rs12329760 |
| 21,42852497,C,T | CAG [G/A]TG TAC | 120 | V | M | -1.78 | Neutral | rs12329760 |
| 22,29130458,T,C | CAA GA[A/G] CCT | 84 | E | E | 0 | Neutral | rs1805129 |
| 22,29130458,T,C | CAA GA[A/G] CCT | 84 | E | E | 0 | Neutral | rs1805129 |
| 22,29130458,T,C | CAA GA[A/G] CCT | 84 | E | E | 0 | Neutral | rs1805129 |
| 22,29130458,T,C | CAA GA[A/G] CCT | 84 | E | E | 0 | Neutral | rs1805129 |
| 22,29130458,T,C | CAA GA[A/G] CCT | 84 | E | E | 0 | Neutral | rs1805129 |
| 22,29130458,T,C | CAA GA[A/G] CCT | 84 | E | E | 0 | Neutral | rs1805129 |
| 22,29130458,T,C | CAA GA[A/G] CCT | 94 | E | E | 0 | Neutral | rs1805129 |
| 22,29130458,T,C | CAA GA[A/G] CCT | 84 | E | E | 0 | Neutral | rs1805129 |
| 22,29130458,T,C | CAA GA[A/G] CCT | 84 | E | E | 0 | Neutral | rs1805129 |
| 22,29130458,T,C | CAA GA[A/G] CCT | 84 | E | E | 0 | Neutral | rs1805129 |
| 22,29130458,T,C | CAA GA[A/G] CCT | 84 | E | E | 0 | Neutral | rs1805129 |
| 22,29130458,T,C | CAA GA[A/G] CCT | 84 | E | E | 0 | Neutral | rs1805129 |
| 22,29130458,T,C | CAA GA[A/G] CCT | 84 | E | E | 0 | Neutral | rs1805129 |
| 22,29130458,T,C | CAA GA[A/G] CCT | 84 | E | E | 0 | Neutral | rs1805129 |
| 22,29130458,T,C | CAA GA[A/G] CCT | 84 | E | E | 0 | Neutral | rs1805129 |
| 22,29130458,T,C | CAA GA[A/G] CCT | 84 | E | E | 0 | Neutral | rs1805129 |
| 22,29130458,T,C | CAA GA[A/G] CCT | 84 | E | E | 0 | Neutral | rs1805129 |
| 22,29130458,T,C | CAA GA[A/G] CCT | 84 | E | E | 0 | Neutral | rs1805129 |
| 22,42059768,G,T | AGT GG[G/T] CTG | 593 | G | G | 0 | Neutral | rs132788 |
| 22,42059768,G,T | AGT GG[G/T] CTG | 593 | G | G | 0 | Neutral | rs132788 |
| 22,42059768,G,T | AGT GG[G/T] CTG | 543 | G | G | 0 | Neutral | rs132788 |
| 22,42059768,G,T | AGT GG[G/T] CTG | 593 | G | G | 0 | Neutral | rs132788 |
| 22,42059768,G,T | AGT GG[G/T] CTG | 552 | G | G | 0 | Neutral | rs132788 |
| 22,42059768,G,T | AGT GG[G/T] CTG | 460 | G | G | 0 | Neutral | rs132788 |
| 22,29085168,C,G | CTT CT[G/C] TCT | 470 | L | L | 0 | Neutral |  |
| 22,29085168,C,G | CTT CT[G/C] TCT | 499 | L | L | 0 | Neutral |  |
| 22,29085168,C,G | CTT CT[G/C] TCT | 182 | L | L | 0 | Neutral |  |
| 22,29085168,C,G | CTT CT[G/C] TCT | 119 | L | L | 0 | Neutral |  |
| 22,29085168,C,G | CTT CT[G/C] TCT | 408 | L | L | 0 | Neutral |  |
| 22,29085168,C,G | CTT CT[G/C] TCT | 542 | L | L | 0 | Neutral |  |
| 22,29085168,C,G | CTT CT[G/C] TCT | 470 | L | L | 0 | Neutral |  |
| 22,29085168,C,G | CTT CT[G/C] TCT | 408 | L | L | 0 | Neutral |  |
| 22,29085168,C,G | CTT CT[G/C] TCT | 499 | L | L | 0 | Neutral |  |
| 22,29085168,C,G | CTT CT[G/C] TCT | 499 | L | L | 0 | Neutral |  |
| 22,29085168,C,G | CTT CT[G/C] TCT | 100 | L | L | 0 | Neutral |  |
| 22,29085168,C,G | CTT CT[G/C] TCT | 232 | L | L | 0 | Neutral |  |
| 22,29085168,C,G | CTT CT[G/C] TCT | 278 | L | L | 0 | Neutral |  |
| 22,29090064,C,A | AAG [G/T]CA CGT | 444 | A | S | 0.38 | Neutral |  |
| 22,29090064,C,A | AAG [G/T]CA CGT | 473 | A | S | 0.49 | Neutral |  |
| 22,29090064,C,A | AAG [G/T]CA CGT | 156 | A | S | 0.34 | Neutral |  |
| 22,29090064,C,A | AAG [G/T]CA CGT | 382 | A | S | 0.29 | Neutral |  |
| 22,29090064,C,A | AAG [G/T]CA CGT | 516 | A | S | 0.36 | Neutral |  |
| 22,29090064,C,A | AAG [G/T]CA CGT | 444 | A | S | 0.38 | Neutral |  |
| 22,29090064,C,A | AAG [G/T]CA CGT | 382 | A | S | 0.29 | Neutral |  |
| 22,29090064,C,A | AAG [G/T]CA CGT | 473 | A | S | 0.49 | Neutral |  |
| 22,29090064,C,A | AAG [G/T]CA CGT | 473 | A | S | 0.49 | Neutral |  |
| 22,29090064,C,A | AAG [G/T]CA CGT | 206 | A | S | 0.31 | Neutral |  |
| 22,29090064,C,A | AAG [G/T]CA CGT | 252 | A | S | 0.33 | Neutral |  |
| 22,29085168,C,G | CTT CT[G/C] TCT | 470 | L | L | 0 | Neutral |  |
| 22,29085168,C,G | CTT CT[G/C] TCT | 499 | L | L | 0 | Neutral |  |
| 22,29085168,C,G | CTT CT[G/C] TCT | 182 | L | L | 0 | Neutral |  |
| 22,29085168,C,G | CTT CT[G/C] TCT | 119 | L | L | 0 | Neutral |  |
| 22,29085168,C,G | CTT CT[G/C] TCT | 408 | L | L | 0 | Neutral |  |
| 22,29085168,C,G | CTT CT[G/C] TCT | 542 | L | L | 0 | Neutral |  |
| 22,29085168,C,G | CTT CT[G/C] TCT | 470 | L | L | 0 | Neutral |  |
| 22,29085168,C,G | CTT CT[G/C] TCT | 408 | L | L | 0 | Neutral |  |
| 22,29085168,C,G | CTT CT[G/C] TCT | 499 | L | L | 0 | Neutral |  |
| 22,29085168,C,G | CTT CT[G/C] TCT | 499 | L | L | 0 | Neutral |  |
| 22,29085168,C,G | CTT CT[G/C] TCT | 100 | L | L | 0 | Neutral |  |
| 22,29085168,C,G | CTT CT[G/C] TCT | 232 | L | L | 0 | Neutral |  |
| 22,29085168,C,G | CTT CT[G/C] TCT | 278 | L | L | 0 | Neutral |  |
| 22,29090064,C,A | AAG [G/T]CA CGT | 444 | A | S | 0.38 | Neutral |  |
| 22,29090064,C,A | AAG [G/T]CA CGT | 473 | A | S | 0.49 | Neutral |  |
| 22,29090064,C,A | AAG [G/T]CA CGT | 156 | A | S | 0.34 | Neutral |  |
| 22,29090064,C,A | AAG [G/T]CA CGT | 382 | A | S | 0.29 | Neutral |  |
| 22,29090064,C,A | AAG [G/T]CA CGT | 516 | A | S | 0.36 | Neutral |  |
| 22,29090064,C,A | AAG [G/T]CA CGT | 444 | A | S | 0.38 | Neutral |  |
| 22,29090064,C,A | AAG [G/T]CA CGT | 382 | A | S | 0.29 | Neutral |  |
| 22,29090064,C,A | AAG [G/T]CA CGT | 473 | A | S | 0.49 | Neutral |  |
| 22,29090064,C,A | AAG [G/T]CA CGT | 473 | A | S | 0.49 | Neutral |  |
| 22,29090064,C,A | AAG [G/T]CA CGT | 206 | A | S | 0.31 | Neutral |  |
| 22,29090064,C,A | AAG [G/T]CA CGT | 252 | A | S | 0.33 | Neutral |  |
| 22,42052998,G,A | GGC AA[G/A] ATG | 461 | K | K | 0 | Neutral | rs147467039 |
| 22,42052998,G,A | GGC AA[G/A] ATG | 461 | K | K | 0 | Neutral | rs147467039 |
| 22,42052998,G,A | GGC AA[G/A] ATG | 411 | K | K | 0 | Neutral | rs147467039 |
| 22,42052998,G,A | GGC AA[G/A] ATG | 461 | K | K | 0 | Neutral | rs147467039 |
| 22,42052998,G,A | GGC AA[G/A] ATG | 461 | K | K | 0 | Neutral | rs147467039 |
| 22,42052998,G,A | GGC AA[G/A] ATG | 420 | K | K | 0 | Neutral | rs147467039 |
| 22,42052998,G,A | GGC AA[G/A] ATG | 328 | K | K | 0 | Neutral | rs147467039 |
| 22,42052998,G,A | GGC AA[G/A] ATG | 461 | K | K | 0 | Neutral | rs147467039 |
| 22,42052998,G,A | GGC AA[G/A] ATG | 461 | K | K | 0 | Neutral | rs147467039 |
| 22,42052998,G,A | GGC AA[G/A] ATG | 411 | K | K | 0 | Neutral | rs147467039 |
| 22,42052998,G,A | GGC AA[G/A] ATG | 461 | K | K | 0 | Neutral | rs147467039 |
| 22,42052998,G,A | GGC AA[G/A] ATG | 461 | K | K | 0 | Neutral | rs147467039 |
| 22,42052998,G,A | GGC AA[G/A] ATG | 420 | K | K | 0 | Neutral | rs147467039 |
| 22,42052998,G,A | GGC AA[G/A] ATG | 328 | K | K | 0 | Neutral | rs147467039 |
| 22,42046893,T,C | GTG A[T/C]T GGG | 376 | I | T | 2.19 | Neutral |  |
| 22,42046893,T,C | GTG A[T/C]T GGG | 376 | I | T | 2.19 | Neutral |  |
| 22,42046893,T,C | GTG A[T/C]T GGG | 326 | I | T | 2.32 | Neutral |  |
| 22,42046893,T,C | GTG A[T/C]T GGG | 376 | I | T | 2.36 | Neutral |  |
| 22,42046893,T,C | GTG A[T/C]T GGG | 376 | I | T | 2.19 | Neutral |  |
| 22,42046893,T,C | GTG A[T/C]T GGG | 335 | I | T | 1.99 | Neutral |  |
| 22,42046893,T,C | GTG A[T/C]T GGG | 243 | I | T | 1.86 | Neutral |  |
| 3,129155670,C,T | GAT [G/A]CT GAA | 273 | A | T | -0.9 | Neutral | rs10342 |
| 3,129155670,C,T | GAT [G/A]CT GAA | 273 | A | T | -0.9 | Neutral | rs10342 |
| 3,129155670,C,T | GAT [G/A]CT GAA | 273 | A | T | -0.93 | Neutral | rs10342 |
| 3,129155670,C,T | GAT [G/A]CT GAA | 273 | A | T | -0.8 | Neutral | rs10342 |
| 3,129152089,G,A | TCA GG[C/T] AAA | 471 | G | G | 0 | Neutral | rs140696 |
| 3,129152089,G,A | TCA GG[C/T] AAA | 153 | G | G | 0 | Neutral | rs140696 |
| 3,129152089,G,A | TCA GG[C/T] AAA | 465 | G | G | 0 | Neutral | rs140696 |
| 3,129152089,G,A | TCA GG[C/T] AAA | 471 | G | G | 0 | Neutral | rs140696 |
| 3,129152089,G,A | TCA GG[C/T] AAA | 471 | G | G | 0 | Neutral | rs140696 |
| 3,9798763,C,T | CCG AC[C/T] TGC | 328 | T | T | 0 | Neutral | rs9824261 |
| 3,9798763,C,T | CCG AC[C/T] TGC | 115 | T | T | 0 | Neutral | rs9824261 |
| 3,9798763,C,T | GAC [C/T]TG CGC | 323 | L | L | 0 | Neutral | rs9824261 |
| 3,9798763,C,T | CCG AC[C/T] TGC | 95 | T | T | 0 | Neutral | rs9824261 |
| 3,14200382,G,T | AAA C[C/A]T TCC | 334 | P | H | -1.23 | Neutral |  |
| 3,14200382,G,T | AAA C[C/A]T TCC | 297 | P | H | -1.46 | Neutral |  |
| 3,14197840,G,A | GTC TA[C/T] TCC | 676 | Y | Y | 0 | Neutral | rs2228004 |
| 3,14197840,G,A | GTC TA[C/T] TCC | 639 | Y | Y | 0 | Neutral | rs2228004 |
| 3,142281755,A,G | AGA AA[T/C] GTG | 163 | N | N | 0 | Neutral |  |
| 3,142281755,A,G | AGA AA[T/C] GTG | 163 | N | N | 0 | Neutral |  |
| 3,142277536,A,G | GAT GA[T/C] GGC | 605 | D | D | 0 | Neutral | rs2227929 |
| 3,142277536,A,G | GAT GA[T/C] GGC | 541 | D | D | 0 | Neutral | rs2227929 |
| 3,142277536,A,G | GAT GA[T/C] GGC | 222 | D | D | 0 | Neutral | rs2227929 |
| 3,142281612,A,G | TTA A[T/C]G GTT | 211 | M | T | -0.2 | Neutral | rs2227928 |
| 3,142281612,A,G | TTA A[T/C]G GTT | 211 | M | T | 0.02 | Neutral | rs2227928 |
| 3,142277575,A,T | TGT GG[T/A] ATG | 592 | G | G | 0 | Neutral | rs2227930 |
| 3,142277575,A,T | TGT GG[T/A] ATG | 528 | G | G | 0 | Neutral | rs2227930 |
| 3,142277575,A,T | TGT GG[T/A] ATG | 209 | G | G | 0 | Neutral | rs2227930 |
| 3,58512237,A,G | TCC TG[T/C] ACC | 434 | C | C | 0 | Neutral | rs1127745 |
| 3,58512237,A,G | TCC TG[T/C] ACC | 420 | C | C | 0 | Neutral | rs1127745 |
| 3,142226860,G,C | CGC TC[C/G] AAA | 1648 | S | S | 0 | Neutral |  |
| 3,142226860,G,C | CGC TC[C/G] AAA | 1584 | S | S | 0 | Neutral |  |
| 3,142168331,C,T | ATA CA[G/A] GAA | 2625 | Q | Q | 0 | Neutral | rs1802904 |
| 3,142168331,C,T | ATA CA[G/A] GAA | 2561 | Q | Q | 0 | Neutral | rs1802904 |
| 3,37053568,A,G | TCC [A/G]TC TTT | 219 | I | V | -0.46 | Neutral | rs1799977 |
| 3,37053568,A,G | TCC [A/G]TC TTT | 83 | I | V | -0.36 | Neutral | rs1799977 |
| 3,37053568,A,G | TCC [A/G]TC TTT | 13 | I | V | -0.3 | Neutral | rs1799977 |
| 3,37053568,A,G | TCC [A/G]TC TTT | 121 | I | V | -0.43 | Neutral | rs1799977 |
| 3,37053568,A,G | TCC [A/G]TC TTT | 185 | I | V | -0.4 | Neutral | rs1799977 |
| 3,37053568,A,G | TCC [A/G]TC TTT | 211 | I | V | -0.39 | Neutral | rs1799977 |
| 3,37053568,A,G | TCC [A/G]TC TTT | 185 | I | V | -0.46 | Neutral | rs1799977 |
| 3,121207143,G,T | ACC CA[C/A] CAG | 1545 | H | Q | 0.66 | Neutral | rs3218652 |
| 3,121207143,G,T | ACC CA[C/A] CAG | 1681 | H | Q | 0.66 | Neutral | rs3218652 |
| 3,121207143,G,T | ACC CA[C/A] CAG | 1168 | H | Q | 0.6 | Neutral | rs3218652 |
| 3,121151784,G,A | GTG G[C/T]A GAA | 2547 | A | V | -2.42 | Neutral | rs2306211 |
| 3,121151784,G,A | GTG G[C/T]A GAA | 2683 | A | V | -2.42 | Neutral | rs2306211 |
| 3,121151784,G,A | GTG G[C/T]A GAA | 2170 | A | V | -2.42 | Neutral | rs2306211 |
| 3,14187449,G,T | GAG [C/A]AG CTG | 939 | Q | K | 1.67 | Neutral | rs2228001 |
| 3,14187449,G,T | GAG [C/A]AG CTG | 902 | Q | K | 1.67 | Neutral | rs2228001 |
| 3,14199887,G,A | CCA G[C/T]G GCA | 499 | A | V | -0.96 | Neutral | rs2228000 |
| 3,14199887,G,A | CCA G[C/T]G GCA | 462 | A | V | -0.96 | Neutral | rs2228000 |
| 3,58512375,T,C | CAC GC[A/G] CTG | 388 | A | A | 0 | Neutral | rs1127743 |
| 3,58512375,T,C | CAC GC[A/G] CTG | 374 | A | A | 0 | Neutral | rs1127743 |
| 3,142268372,C,T | CAT TT[G/A] GTC | 1040 | L | L | 0 | Neutral | rs28910272 |
| 3,142268372,C,T | CAT TT[G/A] GTC | 976 | L | L | 0 | Neutral | rs28910272 |
| 3,142272757,T,C | ATG GA[A/G] GAT | 814 | E | E | 0 | Neutral | rs55895932 |
| 3,142272757,T,C | ATG GA[A/G] GAT | 750 | E | E | 0 | Neutral | rs55895932 |
| 3,142275353,C,T | CTT GA[G/A] TGG | 650 | E | E | 0 | Neutral | rs28910270 |
| 3,142275353,C,T | CTT GA[G/A] TGG | 586 | E | E | 0 | Neutral | rs28910270 |
| 3,142275353,C,T | CTT GA[G/A] TGG | 267 | E | E | 0 | Neutral | rs28910270 |
| 3,142269075,C,T | GAC [G/A]TG CGA | 959 | V | M | 0.11 | Neutral | rs28910271 |
| 3,142269075,C,T | GAC [G/A]TG CGA | 895 | V | M | 0.11 | Neutral | rs28910271 |
| 3,142280108,T,C | TCT AA[A/G] AGA | 442 | K | K | 0 | Neutral | rs28897765 |
| 3,142280108,T,C | TCT AA[A/G] AGA | 442 | K | K | 0 | Neutral | rs28897765 |
| 3,142280108,T,C | TCT AA[A/G] AGA | 123 | K | K | 0 | Neutral | rs28897765 |
| 3,142281298,C,T | CCT [G/A]TC TAT | 316 | V | I | 0.08 | Neutral | rs28897764 |
| 3,142281298,C,T | CCT [G/A]TC TAT | 316 | V | I | -0.06 | Neutral | rs28897764 |
| 3,142215233,G,A | CTG TA[C/T] GTG | 1956 | Y | Y | 0 | Neutral | rs112018640 |
| 3,142215233,G,A | CTG TA[C/T] GTG | 1892 | Y | Y | 0 | Neutral | rs112018640 |
| 3,129155670,C,T | GAT [G/A]CT GAA | 273 | A | T | -0.9 | Neutral | rs10342 |
| 3,129155670,C,T | GAT [G/A]CT GAA | 273 | A | T | -0.9 | Neutral | rs10342 |
| 3,129155670,C,T | GAT [G/A]CT GAA | 273 | A | T | -0.93 | Neutral | rs10342 |
| 3,129155670,C,T | GAT [G/A]CT GAA | 273 | A | T | -0.8 | Neutral | rs10342 |
| 3,121207143,G,T | ACC CA[C/A] CAG | 1545 | H | Q | 0.66 | Neutral | rs3218652 |
| 3,121207143,G,T | ACC CA[C/A] CAG | 1681 | H | Q | 0.66 | Neutral | rs3218652 |
| 3,121207143,G,T | ACC CA[C/A] CAG | 1168 | H | Q | 0.6 | Neutral | rs3218652 |
| 3,142277575,A,T | TGT GG[T/A] ATG | 592 | G | G | 0 | Neutral | rs2227930 |
| 3,142277575,A,T | TGT GG[T/A] ATG | 528 | G | G | 0 | Neutral | rs2227930 |
| 3,142277575,A,T | TGT GG[T/A] ATG | 209 | G | G | 0 | Neutral | rs2227930 |
| 3,142281612,A,G | TTA A[T/C]G GTT | 211 | M | T | -0.2 | Neutral | rs2227928 |
| 3,142281612,A,G | TTA A[T/C]G GTT | 211 | M | T | 0.02 | Neutral | rs2227928 |
| 3,129152089,G,A | TCA GG[C/T] AAA | 471 | G | G | 0 | Neutral | rs140696 |
| 3,129152089,G,A | TCA GG[C/T] AAA | 153 | G | G | 0 | Neutral | rs140696 |
| 3,129152089,G,A | TCA GG[C/T] AAA | 465 | G | G | 0 | Neutral | rs140696 |
| 3,129152089,G,A | TCA GG[C/T] AAA | 471 | G | G | 0 | Neutral | rs140696 |
| 3,129152089,G,A | TCA GG[C/T] AAA | 471 | G | G | 0 | Neutral | rs140696 |
| 3,121208833,G,C | CAG A[C/G]A TGT | 982 | T | R | -0.34 | Neutral | rs3218649 |
| 3,121208833,G,C | CAG A[C/G]A TGT | 1118 | T | R | -0.34 | Neutral | rs3218649 |
| 3,121208833,G,C | CAG A[C/G]A TGT | 605 | T | R | -0.28 | Neutral | rs3218649 |
| 3,142217537,A,G | TTT TA[T/C] GAC | 1820 | Y | Y | 0 | Neutral | rs2227932 |
| 3,142217537,A,G | TTT TA[T/C] GAC | 1756 | Y | Y | 0 | Neutral | rs2227932 |
| 3,37038191,C,T | GGC AC[C/T] GGG | 66 | T | T | 0 | Neutral | rs61751642 |
| 3,37038191,C,T | GGC AC[C/T] GGG | 32 | T | T | 0 | Neutral | rs61751642 |
| 3,37038191,C,T | GGC AC[C/T] GGG | 58 | T | T | 0 | Neutral | rs61751642 |
| 3,37038191,C,T | GGC AC[C/T] GGG | 32 | T | T | 0 | Neutral | rs61751642 |
| 3,186509517,G,A | ATT GC[C/T] GGG | 266 | A | A | 0 | Neutral | rs187868 |
| 3,186509517,G,A | ATT GC[C/T] GGG | 266 | A | A | 0 | Neutral | rs187868 |
| 3,186509517,G,A | ATT GC[C/T] GGG | 266 | A | A | 0 | Neutral | rs187868 |
| 3,186509517,G,A | ATT GC[C/T] GGG | 41 | A | A | 0 | Neutral | rs187868 |
| 3,14200382,G,T | AAA C[C/A]T TCC | 334 | P | H | -1.23 | Neutral |  |
| 3,14200382,G,T | AAA C[C/A]T TCC | 297 | P | H | -1.46 | Neutral |  |
| 3,121154974,T,C | GAC C[A/G]A ACA | 2513 | Q | R | 0.77 | Neutral | rs1381057 |
| 3,121154974,T,C | GAC C[A/G]A ACA | 2649 | Q | R | 0.77 | Neutral | rs1381057 |
| 3,121154974,T,C | GAC C[A/G]A ACA | 2136 | Q | R | 0.84 | Neutral | rs1381057 |
| 3,14187449,G,T | GAG [C/A]AG CTG | 939 | Q | K | 1.67 | Neutral | rs2228001 |
| 3,14187449,G,T | GAG [C/A]AG CTG | 902 | Q | K | 1.67 | Neutral | rs2228001 |
| 3,129155670,C,T | GAT [G/A]CT GAA | 273 | A | T | -0.9 | Neutral | rs10342 |
| 3,129155670,C,T | GAT [G/A]CT GAA | 273 | A | T | -0.9 | Neutral | rs10342 |
| 3,129155670,C,T | GAT [G/A]CT GAA | 273 | A | T | -0.93 | Neutral | rs10342 |
| 3,129155670,C,T | GAT [G/A]CT GAA | 273 | A | T | -0.8 | Neutral | rs10342 |
| 3,121208338,G,A | GTG A[C/T]T TGT | 1147 | T | I | -1.69 | Neutral |  |
| 3,121208338,G,A | GTG A[C/T]T TGT | 1283 | T | I | -1.69 | Neutral |  |
| 3,121208338,G,A | GTG A[C/T]T TGT | 770 | T | I | -1.69 | Neutral |  |
| 3,186509517,G,A | ATT GC[C/T] GGG | 266 | A | A | 0 | Neutral | rs187868 |
| 3,186509517,G,A | ATT GC[C/T] GGG | 266 | A | A | 0 | Neutral | rs187868 |
| 3,186509517,G,A | ATT GC[C/T] GGG | 266 | A | A | 0 | Neutral | rs187868 |
| 3,186509517,G,A | ATT GC[C/T] GGG | 41 | A | A | 0 | Neutral | rs187868 |
| 3,129152090,C,A | CTC CG[G/T] CAA | 35 | R | R | 0 | Neutral |  |
| 3,58512285,T,C | TAC TC[A/G] AAG | 418 | S | S | 0 | Neutral | rs13097249 |
| 3,58512285,T,C | TAC TC[A/G] AAG | 404 | S | S | 0 | Neutral | rs13097249 |
| 3,129152089,G,A | TCA GG[C/T] AAA | 471 | G | G | 0 | Neutral | rs140696 |
| 3,129152089,G,A | TCA GG[C/T] AAA | 153 | G | G | 0 | Neutral | rs140696 |
| 3,129152089,G,A | TCA GG[C/T] AAA | 465 | G | G | 0 | Neutral | rs140696 |
| 3,129152089,G,A | TCA GG[C/T] AAA | 471 | G | G | 0 | Neutral | rs140696 |
| 3,129152089,G,A | TCA GG[C/T] AAA | 471 | G | G | 0 | Neutral | rs140696 |
| 3,14193889,C,T | TCC AG[G/A] GAC | 687 | R | R | 0 | Neutral | rs2227998 |
| 3,14193889,C,T | TCC AG[G/A] GAC | 650 | R | R | 0 | Neutral | rs2227998 |
| 3,14193889,C,T | CAG [G/A]GA CAC | 130 | G | R | 1.4 | Neutral | rs2227998 |
| 3,142217537,A,G | TTT TA[T/C] GAC | 1820 | Y | Y | 0 | Neutral | rs2227932 |
| 3,142217537,A,G | TTT TA[T/C] GAC | 1756 | Y | Y | 0 | Neutral | rs2227932 |
| 3,37038191,C,T | GGC AC[C/T] GGG | 66 | T | T | 0 | Neutral | rs61751642 |
| 3,37038191,C,T | GGC AC[C/T] GGG | 32 | T | T | 0 | Neutral | rs61751642 |
| 3,37038191,C,T | GGC AC[C/T] GGG | 58 | T | T | 0 | Neutral | rs61751642 |
| 3,37038191,C,T | GGC AC[C/T] GGG | 32 | T | T | 0 | Neutral | rs61751642 |
| 3,186509517,G,A | ATT GC[C/T] GGG | 266 | A | A | 0 | Neutral | rs187868 |
| 3,186509517,G,A | ATT GC[C/T] GGG | 266 | A | A | 0 | Neutral | rs187868 |
| 3,186509517,G,A | ATT GC[C/T] GGG | 266 | A | A | 0 | Neutral | rs187868 |
| 3,186509517,G,A | ATT GC[C/T] GGG | 41 | A | A | 0 | Neutral | rs187868 |
| 3,14200382,G,T | AAA C[C/A]T TCC | 334 | P | H | -1.23 | Neutral |  |
| 3,14200382,G,T | AAA C[C/A]T TCC | 297 | P | H | -1.46 | Neutral |  |
| 3,121154974,T,C | GAC C[A/G]A ACA | 2513 | Q | R | 0.77 | Neutral | rs1381057 |
| 3,121154974,T,C | GAC C[A/G]A ACA | 2649 | Q | R | 0.77 | Neutral | rs1381057 |
| 3,121154974,T,C | GAC C[A/G]A ACA | 2136 | Q | R | 0.84 | Neutral | rs1381057 |
| 3,14187449,G,T | GAG [C/A]AG CTG | 939 | Q | K | 1.67 | Neutral | rs2228001 |
| 3,14187449,G,T | GAG [C/A]AG CTG | 902 | Q | K | 1.67 | Neutral | rs2228001 |
| 3,58512285,T,C | TAC TC[A/G] AAG | 418 | S | S | 0 | Neutral | rs13097249 |
| 3,58512285,T,C | TAC TC[A/G] AAG | 404 | S | S | 0 | Neutral | rs13097249 |
| 3,129155463,A,G | GAC [T/C]CA GAA | 342 | S | P | -0.88 | Neutral | rs2307289 |
| 3,129155463,A,G | GAC [T/C]CA GAA | 342 | S | P | -1.03 | Neutral | rs2307289 |
| 3,129155463,A,G | GAC [T/C]CA GAA | 342 | S | P | -0.62 | Neutral | rs2307289 |
| 3,129155463,A,G | GAC [T/C]CA GAA | 342 | S | P | -0.82 | Neutral | rs2307289 |
| 3,121208176,T,C | TCT C[A/G]T GAA | 1201 | H | R | -0.47 | Neutral | rs3218651 |
| 3,121208176,T,C | TCT C[A/G]T GAA | 1337 | H | R | -0.47 | Neutral | rs3218651 |
| 3,121208176,T,C | TCT C[A/G]T GAA | 824 | H | R | -0.44 | Neutral | rs3218651 |
| 3,14193889,C,T | TCC AG[G/A] GAC | 687 | R | R | 0 | Neutral | rs2227998 |
| 3,14193889,C,T | TCC AG[G/A] GAC | 650 | R | R | 0 | Neutral | rs2227998 |
| 3,14193889,C,T | CAG [G/A]GA CAC | 130 | G | R | 1.4 | Neutral | rs2227998 |
| 3,14187594,G,A | GGG AC[C/T] AGC | 890 | T | T | 0 | Neutral |  |
| 3,14187594,G,A | GGG AC[C/T] AGC | 853 | T | T | 0 | Neutral |  |
| 3,142266595,G,T | GGC C[C/A]G AGA | 1110 | P | Q | -1.98 | Neutral |  |
| 3,142266595,G,T | GGC C[C/A]G AGA | 1046 | P | Q | -1.98 | Neutral |  |
| 3,186509517,G,A | ATT GC[C/T] GGG | 266 | A | A | 0 | Neutral | rs187868 |
| 3,186509517,G,A | ATT GC[C/T] GGG | 266 | A | A | 0 | Neutral | rs187868 |
| 3,186509517,G,A | ATT GC[C/T] GGG | 266 | A | A | 0 | Neutral | rs187868 |
| 3,186509517,G,A | ATT GC[C/T] GGG | 41 | A | A | 0 | Neutral | rs187868 |
| 3,14187449,G,T | GAG [C/A]AG CTG | 939 | Q | K | 1.67 | Neutral | rs2228001 |
| 3,14187449,G,T | GAG [C/A]AG CTG | 902 | Q | K | 1.67 | Neutral | rs2228001 |
| 3,14199887,G,A | CCA G[C/T]G GCA | 499 | A | V | -0.96 | Neutral | rs2228000 |
| 3,14199887,G,A | CCA G[C/T]G GCA | 462 | A | V | -0.96 | Neutral | rs2228000 |
| 3,121208176,T,C | TCT C[A/G]T GAA | 1201 | H | R | -0.47 | Neutral | rs3218651 |
| 3,121208176,T,C | TCT C[A/G]T GAA | 1337 | H | R | -0.47 | Neutral | rs3218651 |
| 3,121208176,T,C | TCT C[A/G]T GAA | 824 | H | R | -0.44 | Neutral | rs3218651 |
| 3,121207258,A,G | GAT A[T/C]G CAA | 1507 | M | T | 0.5 | Neutral | rs35877350 |
| 3,121207258,A,G | GAT A[T/C]G CAA | 1643 | M | T | 0.5 | Neutral | rs35877350 |
| 3,121207258,A,G | GAT A[T/C]G CAA | 1130 | M | T | 0.26 | Neutral | rs35877350 |
| 3,121186422,G,A | GCT G[C/T]A GAC | 2304 | A | V | -1.55 | Neutral | rs532411 |
| 3,121186422,G,A | GCT G[C/T]A GAC | 2440 | A | V | -1.55 | Neutral | rs532411 |
| 3,121186422,G,A | GCT G[C/T]A GAC | 1927 | A | V | -1.61 | Neutral | rs532411 |
| 3,142277575,A,T | TGT GG[T/A] ATG | 592 | G | G | 0 | Neutral | rs2227930 |
| 3,142277575,A,T | TGT GG[T/A] ATG | 528 | G | G | 0 | Neutral | rs2227930 |
| 3,142277575,A,T | TGT GG[T/A] ATG | 209 | G | G | 0 | Neutral | rs2227930 |
| 3,142281612,A,G | TTA A[T/C]G GTT | 211 | M | T | -0.2 | Neutral | rs2227928 |
| 3,142281612,A,G | TTA A[T/C]G GTT | 211 | M | T | 0.02 | Neutral | rs2227928 |
| 3,142215233,G,A | CTG TA[C/T] GTG | 1956 | Y | Y | 0 | Neutral | rs112018640 |
| 3,142215233,G,A | CTG TA[C/T] GTG | 1892 | Y | Y | 0 | Neutral | rs112018640 |
| 3,142275353,C,T | CTT GA[G/A] TGG | 650 | E | E | 0 | Neutral | rs28910270 |
| 3,142275353,C,T | CTT GA[G/A] TGG | 586 | E | E | 0 | Neutral | rs28910270 |
| 3,142275353,C,T | CTT GA[G/A] TGG | 267 | E | E | 0 | Neutral | rs28910270 |
| 3,142268372,C,T | CAT TT[G/A] GTC | 1040 | L | L | 0 | Neutral | rs28910272 |
| 3,142268372,C,T | CAT TT[G/A] GTC | 976 | L | L | 0 | Neutral | rs28910272 |
| 3,142272757,T,C | ATG GA[A/G] GAT | 814 | E | E | 0 | Neutral | rs55895932 |
| 3,142272757,T,C | ATG GA[A/G] GAT | 750 | E | E | 0 | Neutral | rs55895932 |
| 3,142269075,C,T | GAC [G/A]TG CGA | 959 | V | M | 0.11 | Neutral | rs28910271 |
| 3,142269075,C,T | GAC [G/A]TG CGA | 895 | V | M | 0.11 | Neutral | rs28910271 |
| 3,142281298,C,T | CCT [G/A]TC TAT | 316 | V | I | 0.08 | Neutral | rs28897764 |
| 3,142281298,C,T | CCT [G/A]TC TAT | 316 | V | I | -0.06 | Neutral | rs28897764 |
| 3,142280108,T,C | TCT AA[A/G] AGA | 442 | K | K | 0 | Neutral | rs28897765 |
| 3,142280108,T,C | TCT AA[A/G] AGA | 442 | K | K | 0 | Neutral | rs28897765 |
| 3,142280108,T,C | TCT AA[A/G] AGA | 123 | K | K | 0 | Neutral | rs28897765 |
| 3,129155670,C,T | GAT [G/A]CT GAA | 273 | A | T | -0.9 | Neutral | rs10342 |
| 3,129155670,C,T | GAT [G/A]CT GAA | 273 | A | T | -0.9 | Neutral | rs10342 |
| 3,129155670,C,T | GAT [G/A]CT GAA | 273 | A | T | -0.93 | Neutral | rs10342 |
| 3,129155670,C,T | GAT [G/A]CT GAA | 273 | A | T | -0.8 | Neutral | rs10342 |
| 3,121207143,G,T | ACC CA[C/A] CAG | 1545 | H | Q | 0.66 | Neutral | rs3218652 |
| 3,121207143,G,T | ACC CA[C/A] CAG | 1681 | H | Q | 0.66 | Neutral | rs3218652 |
| 3,121207143,G,T | ACC CA[C/A] CAG | 1168 | H | Q | 0.6 | Neutral | rs3218652 |
| 3,186509517,G,A | ATT GC[C/T] GGG | 266 | A | A | 0 | Neutral | rs187868 |
| 3,186509517,G,A | ATT GC[C/T] GGG | 266 | A | A | 0 | Neutral | rs187868 |
| 3,186509517,G,A | ATT GC[C/T] GGG | 266 | A | A | 0 | Neutral | rs187868 |
| 3,186509517,G,A | ATT GC[C/T] GGG | 41 | A | A | 0 | Neutral | rs187868 |
| 3,129152089,G,A | TCA GG[C/T] AAA | 471 | G | G | 0 | Neutral | rs140696 |
| 3,129152089,G,A | TCA GG[C/T] AAA | 153 | G | G | 0 | Neutral | rs140696 |
| 3,129152089,G,A | TCA GG[C/T] AAA | 465 | G | G | 0 | Neutral | rs140696 |
| 3,129152089,G,A | TCA GG[C/T] AAA | 471 | G | G | 0 | Neutral | rs140696 |
| 3,129152089,G,A | TCA GG[C/T] AAA | 471 | G | G | 0 | Neutral | rs140696 |
| 3,129155670,C,T | GAT [G/A]CT GAA | 273 | A | T | -0.9 | Neutral | rs10342 |
| 3,129155670,C,T | GAT [G/A]CT GAA | 273 | A | T | -0.9 | Neutral | rs10342 |
| 3,129155670,C,T | GAT [G/A]CT GAA | 273 | A | T | -0.93 | Neutral | rs10342 |
| 3,129155670,C,T | GAT [G/A]CT GAA | 273 | A | T | -0.8 | Neutral | rs10342 |
| 3,121208833,G,C | CAG A[C/G]A TGT | 982 | T | R | -0.34 | Neutral | rs3218649 |
| 3,121208833,G,C | CAG A[C/G]A TGT | 1118 | T | R | -0.34 | Neutral | rs3218649 |
| 3,121208833,G,C | CAG A[C/G]A TGT | 605 | T | R | -0.28 | Neutral | rs3218649 |
| 3,142281353,C,G | TCA AA[G/C] CTG | 297 | K | N | -0.62 | Neutral | rs2229033 |
| 3,142281353,C,G | TCA AA[G/C] CTG | 297 | K | N | -0.73 | Neutral | rs2229033 |
| 3,129155670,C,T | GAT [G/A]CT GAA | 273 | A | T | -0.9 | Neutral | rs10342 |
| 3,129155670,C,T | GAT [G/A]CT GAA | 273 | A | T | -0.9 | Neutral | rs10342 |
| 3,129155670,C,T | GAT [G/A]CT GAA | 273 | A | T | -0.93 | Neutral | rs10342 |
| 3,129155670,C,T | GAT [G/A]CT GAA | 273 | A | T | -0.8 | Neutral | rs10342 |
| 3,129152089,G,A | TCA GG[C/T] AAA | 471 | G | G | 0 | Neutral | rs140696 |
| 3,129152089,G,A | TCA GG[C/T] AAA | 153 | G | G | 0 | Neutral | rs140696 |
| 3,129152089,G,A | TCA GG[C/T] AAA | 465 | G | G | 0 | Neutral | rs140696 |
| 3,129152089,G,A | TCA GG[C/T] AAA | 471 | G | G | 0 | Neutral | rs140696 |
| 3,129152089,G,A | TCA GG[C/T] AAA | 471 | G | G | 0 | Neutral | rs140696 |
| 3,121208176,T,C | TCT C[A/G]T GAA | 1201 | H | R | -0.47 | Neutral | rs3218651 |
| 3,121208176,T,C | TCT C[A/G]T GAA | 1337 | H | R | -0.47 | Neutral | rs3218651 |
| 3,121208176,T,C | TCT C[A/G]T GAA | 824 | H | R | -0.44 | Neutral | rs3218651 |
| 3,14199887,G,A | CCA G[C/T]G GCA | 499 | A | V | -0.96 | Neutral | rs2228000 |
| 3,14199887,G,A | CCA G[C/T]G GCA | 462 | A | V | -0.96 | Neutral | rs2228000 |
| 3,142222284,A,G | CAT TA[T/C] CAT | 1736 | Y | Y | 0 | Neutral | rs2227931 |
| 3,142222284,A,G | CAT TA[T/C] CAT | 1672 | Y | Y | 0 | Neutral | rs2227931 |
| 3,186509517,G,A | ATT GC[C/T] GGG | 266 | A | A | 0 | Neutral | rs187868 |
| 3,186509517,G,A | ATT GC[C/T] GGG | 266 | A | A | 0 | Neutral | rs187868 |
| 3,186509517,G,A | ATT GC[C/T] GGG | 266 | A | A | 0 | Neutral | rs187868 |
| 3,186509517,G,A | ATT GC[C/T] GGG | 41 | A | A | 0 | Neutral | rs187868 |
| 3,14187449,G,T | GAG [C/A]AG CTG | 939 | Q | K | 1.67 | Neutral | rs2228001 |
| 3,14187449,G,T | GAG [C/A]AG CTG | 902 | Q | K | 1.67 | Neutral | rs2228001 |
| 3,129155670,C,T | GAT [G/A]CT GAA | 273 | A | T | -0.9 | Neutral | rs10342 |
| 3,129155670,C,T | GAT [G/A]CT GAA | 273 | A | T | -0.9 | Neutral | rs10342 |
| 3,129155670,C,T | GAT [G/A]CT GAA | 273 | A | T | -0.93 | Neutral | rs10342 |
| 3,129155670,C,T | GAT [G/A]CT GAA | 273 | A | T | -0.8 | Neutral | rs10342 |
| 3,58512237,A,G | TCC TG[T/C] ACC | 434 | C | C | 0 | Neutral | rs1127745 |
| 3,58512237,A,G | TCC TG[T/C] ACC | 420 | C | C | 0 | Neutral | rs1127745 |
| 3,37053568,A,G | TCC [A/G]TC TTT | 219 | I | V | -0.46 | Neutral | rs1799977 |
| 3,37053568,A,G | TCC [A/G]TC TTT | 83 | I | V | -0.36 | Neutral | rs1799977 |
| 3,37053568,A,G | TCC [A/G]TC TTT | 13 | I | V | -0.3 | Neutral | rs1799977 |
| 3,37053568,A,G | TCC [A/G]TC TTT | 121 | I | V | -0.43 | Neutral | rs1799977 |
| 3,37053568,A,G | TCC [A/G]TC TTT | 185 | I | V | -0.4 | Neutral | rs1799977 |
| 3,37053568,A,G | TCC [A/G]TC TTT | 211 | I | V | -0.39 | Neutral | rs1799977 |
| 3,37053568,A,G | TCC [A/G]TC TTT | 185 | I | V | -0.46 | Neutral | rs1799977 |
| 3,58512285,T,C | TAC TC[A/G] AAG | 418 | S | S | 0 | Neutral | rs13097249 |
| 3,58512285,T,C | TAC TC[A/G] AAG | 404 | S | S | 0 | Neutral | rs13097249 |
| 3,129152089,G,A | TCA GG[C/T] AAA | 471 | G | G | 0 | Neutral | rs140696 |
| 3,129152089,G,A | TCA GG[C/T] AAA | 153 | G | G | 0 | Neutral | rs140696 |
| 3,129152089,G,A | TCA GG[C/T] AAA | 465 | G | G | 0 | Neutral | rs140696 |
| 3,129152089,G,A | TCA GG[C/T] AAA | 471 | G | G | 0 | Neutral | rs140696 |
| 3,129152089,G,A | TCA GG[C/T] AAA | 471 | G | G | 0 | Neutral | rs140696 |
| 3,48228212,G,A | GGC TC[C/T] TCC | 76 | S | S | 0 | Neutral | rs3731494 |
| 3,48228212,G,A | GGC TC[C/T] TCC | 76 | S | S | 0 | Neutral | rs3731494 |
| 3,48228212,G,A | GGC TC[C/T] TCC | 76 | S | S | 0 | Neutral | rs3731494 |
| 3,48228212,G,A | GGC TC[C/T] TCC | 76 | S | S | 0 | Neutral | rs3731494 |
| 3,142281612,A,G | TTA A[T/C]G GTT | 211 | M | T | -0.2 | Neutral | rs2227928 |
| 3,142281612,A,G | TTA A[T/C]G GTT | 211 | M | T | 0.02 | Neutral | rs2227928 |
| 3,14193889,C,T | TCC AG[G/A] GAC | 687 | R | R | 0 | Neutral | rs2227998 |
| 3,14193889,C,T | TCC AG[G/A] GAC | 650 | R | R | 0 | Neutral | rs2227998 |
| 3,14193889,C,T | CAG [G/A]GA CAC | 130 | G | R | 1.4 | Neutral | rs2227998 |
| 3,121154974,T,C | GAC C[A/G]A ACA | 2513 | Q | R | 0.77 | Neutral | rs1381057 |
| 3,121154974,T,C | GAC C[A/G]A ACA | 2649 | Q | R | 0.77 | Neutral | rs1381057 |
| 3,121154974,T,C | GAC C[A/G]A ACA | 2136 | Q | R | 0.84 | Neutral | rs1381057 |
| 3,14187449,G,T | GAG [C/A]AG CTG | 939 | Q | K | 1.67 | Neutral | rs2228001 |
| 3,14187449,G,T | GAG [C/A]AG CTG | 902 | Q | K | 1.67 | Neutral | rs2228001 |
| 3,121208833,G,C | CAG A[C/G]A TGT | 982 | T | R | -0.34 | Neutral | rs3218649 |
| 3,121208833,G,C | CAG A[C/G]A TGT | 1118 | T | R | -0.34 | Neutral | rs3218649 |
| 3,121208833,G,C | CAG A[C/G]A TGT | 605 | T | R | -0.28 | Neutral | rs3218649 |
| 3,142222284,A,G | CAT TA[T/C] CAT | 1736 | Y | Y | 0 | Neutral | rs2227931 |
| 3,142222284,A,G | CAT TA[T/C] CAT | 1672 | Y | Y | 0 | Neutral | rs2227931 |
| 3,142277536,A,G | GAT GA[T/C] GGC | 605 | D | D | 0 | Neutral | rs2227929 |
| 3,142277536,A,G | GAT GA[T/C] GGC | 541 | D | D | 0 | Neutral | rs2227929 |
| 3,142277536,A,G | GAT GA[T/C] GGC | 222 | D | D | 0 | Neutral | rs2227929 |
| 3,142277575,A,T | TGT GG[T/A] ATG | 592 | G | G | 0 | Neutral | rs2227930 |
| 3,142277575,A,T | TGT GG[T/A] ATG | 528 | G | G | 0 | Neutral | rs2227930 |
| 3,142277575,A,T | TGT GG[T/A] ATG | 209 | G | G | 0 | Neutral | rs2227930 |
| 4,39306504,C,G | ACC CG[G/C] AGT | 680 | R | R | 0 | Neutral | rs2066792 |
| 4,39306504,C,G | ACC CG[G/C] AGT | 681 | R | R | 0 | Neutral | rs2066792 |
| 4,39306504,C,G | ACC CG[G/C] AGT | 143 | R | R | 0 | Neutral | rs2066792 |
| 4,39302029,T,C | GGC CC[A/G] TTT | 847 | P | P | 0 | Neutral | rs2066786 |
| 4,39302029,T,C | GGC CC[A/G] TTT | 848 | P | P | 0 | Neutral | rs2066786 |
| 4,178231152,C,A | ATT CG[C/A] GCG | 15 | R | R | 0 | Neutral | rs10013040 |
| 4,178231152,C,A | ATT CG[C/A] GCG | 15 | R | R | 0 | Neutral | rs10013040 |
| 4,178272700,A,G | CCT [A/G]TT GAT | 346 | I | V | 0.09 | Neutral | rs17064676 |
| 4,178274819,A,C | AAA A[A/C]A CCG | 466 | K | T | -1.89 | Neutral | rs61739870 |
| 4,178274639,C,T | AAA A[C/T]A AAG | 406 | T | I | -0.39 | Neutral | rs61742279 |
| 4,103518700,A,G | GCT [A/G]TG CAG | 507 | M | V | -0.44 | Neutral | rs4648072 |
| 4,103518700,A,G | GCT [A/G]TG CAG | 506 | M | V | -0.44 | Neutral | rs4648072 |
| 4,103518700,A,G | GCT [A/G]TG CAG | 506 | M | V | -0.44 | Neutral | rs4648072 |
| 4,39306504,C,G | ACC CG[G/C] AGT | 680 | R | R | 0 | Neutral | rs2066792 |
| 4,39306504,C,G | ACC CG[G/C] AGT | 681 | R | R | 0 | Neutral | rs2066792 |
| 4,39306504,C,G | ACC CG[G/C] AGT | 143 | R | R | 0 | Neutral | rs2066792 |
| 4,122741807,C,T | CTA CA[G/A] AAT | 228 | Q | Q | 0 | Neutral | rs769243 |
| 4,103537628,C,T | AGC GG[C/T] GTG | 929 | G | G | 0 | Neutral |  |
| 4,103537628,C,T | AGC GG[C/T] GTG | 928 | G | G | 0 | Neutral |  |
| 4,103537628,C,T | AGC GG[C/T] GTG | 928 | G | G | 0 | Neutral |  |
| 4,122742217,T,C | ATT [A/G]TA TTA | 163 | I | V | 0.42 | Neutral | rs769242 |
| 4,178274565,A,G | AAC AG[A/G] AAA | 381 | R | R | 0 | Neutral | rs113130668 |
| 4,178274750,C,T | CAA C[C/T]A TCC | 443 | P | L | -0.36 | Neutral | rs13112358 |
| 4,103505872,G,A | AAA [G/A]AT ATT | 321 | D | N | -0.78 | Neutral |  |
| 4,103505872,G,A | AAA [G/A]AT ATT | 320 | D | N | -0.78 | Neutral |  |
| 4,103505872,G,A | AAA [G/A]AT ATT | 320 | D | N | -0.78 | Neutral |  |
| 4,103505872,G,A | AAA [G/A]AT ATT | 114 | D | N | -1.68 | Neutral |  |
| 4,39322966,G,A | GAA A[C/T]G TTT | 250 | T | M | -1.43 | Neutral |  |
| 4,39322966,G,A | GAA A[C/T]G TTT | 250 | T | M | -1.47 | Neutral |  |
| 4,122741807,C,T | CTA CA[G/A] AAT | 228 | Q | Q | 0 | Neutral | rs769243 |
| 4,103537628,C,T | AGC GG[C/T] GTG | 929 | G | G | 0 | Neutral |  |
| 4,103537628,C,T | AGC GG[C/T] GTG | 928 | G | G | 0 | Neutral |  |
| 4,103537628,C,T | AGC GG[C/T] GTG | 928 | G | G | 0 | Neutral |  |
| 4,122742217,T,C | ATT [A/G]TA TTA | 163 | I | V | 0.42 | Neutral | rs769242 |
| 4,39303925,A,G | GAT TC[T/C] CAC | 836 | S | S | 0 | Neutral | rs2066782 |
| 4,39303925,A,G | GAT TC[T/C] CAC | 837 | S | S | 0 | Neutral | rs2066782 |
| 4,178257364,G,C | AAA CA[G/C] AAA | 172 | Q | H | -2.36 | Neutral | rs17064658 |
| 4,178274835,A,C | GCC CA[A/C] TAC | 471 | Q | H | 1.51 | Neutral | rs13112390 |
| 4,178274565,A,G | AAC AG[A/G] AAA | 381 | R | R | 0 | Neutral | rs113130668 |
| 4,178231152,C,A | ATT CG[C/A] GCG | 15 | R | R | 0 | Neutral | rs10013040 |
| 4,178231152,C,A | ATT CG[C/A] GCG | 15 | R | R | 0 | Neutral | rs10013040 |
| 4,178272700,A,G | CCT [A/G]TT GAT | 346 | I | V | 0.09 | Neutral | rs17064676 |
| 4,178274639,C,T | AAA A[C/T]A AAG | 406 | T | I | -0.39 | Neutral | rs61742279 |
| 4,178274819,A,C | AAA A[A/C]A CCG | 466 | K | T | -1.89 | Neutral | rs61739870 |
| 4,103518700,A,G | GCT [A/G]TG CAG | 507 | M | V | -0.44 | Neutral | rs4648072 |
| 4,103518700,A,G | GCT [A/G]TG CAG | 506 | M | V | -0.44 | Neutral | rs4648072 |
| 4,103518700,A,G | GCT [A/G]TG CAG | 506 | M | V | -0.44 | Neutral | rs4648072 |
| 4,39306504,C,G | ACC CG[G/C] AGT | 680 | R | R | 0 | Neutral | rs2066792 |
| 4,39306504,C,G | ACC CG[G/C] AGT | 681 | R | R | 0 | Neutral | rs2066792 |
| 4,39306504,C,G | ACC CG[G/C] AGT | 143 | R | R | 0 | Neutral | rs2066792 |
| 4,178274565,A,G | AAC AG[A/G] AAA | 381 | R | R | 0 | Neutral | rs113130668 |
| 4,178274694,T,G | AGT GT[T/G] TGT | 424 | V | V | 0 | Neutral | rs10007075 |
| 4,178274750,C,T | CAA C[C/T]A TCC | 443 | P | L | -0.36 | Neutral | rs13112358 |
| 4,178274835,A,C | GCC CA[A/C] TAC | 471 | Q | H | 1.51 | Neutral | rs13112390 |
| 4,178262683,T,C | CGT CC[T/C] AAT | 252 | P | P | 0 | Neutral | rs17676249 |
| 4,178257364,G,C | AAA CA[G/C] AAA | 172 | Q | H | -2.36 | Neutral | rs17064658 |
| 4,178274694,T,G | AGT GT[T/G] TGT | 424 | V | V | 0 | Neutral | rs10007075 |
| 4,178274835,A,C | GCC CA[A/C] TAC | 471 | Q | H | 1.51 | Neutral | rs13112390 |
| 4,39303925,A,G | GAT TC[T/C] CAC | 836 | S | S | 0 | Neutral | rs2066782 |
| 4,39303925,A,G | GAT TC[T/C] CAC | 837 | S | S | 0 | Neutral | rs2066782 |
| 4,178274565,A,G | AAC AG[A/G] AAA | 381 | R | R | 0 | Neutral | rs113130668 |
| 4,178274750,C,T | CAA C[C/T]A TCC | 443 | P | L | -0.36 | Neutral | rs13112358 |
| 5,131951811,G,A | GTT TT[G/A] CAA | 1051 | L | L | 0 | Neutral | rs35800931 |
| 5,131951811,G,A | GTT TT[G/A] CAA | 912 | L | L | 0 | Neutral | rs35800931 |
| 5,131915574,C,T | GAA A[C/T]A CTT | 191 | T | I | -2.43 | Neutral | rs2230017 |
| 5,131915574,C,T | GAA A[C/T]A CTT | 52 | T | I | -2.43 | Neutral | rs2230017 |
| 5,131915574,C,T | GAA A[C/T]A CTT | 92 | T | I | -1.7 | Neutral | rs2230017 |
| 5,131915574,C,T | GAA A[C/T]A CTT | 191 | T | I | -2.4 | Neutral | rs2230017 |
| 5,131915574,C,T | GAA A[C/T]A CTT | 191 | T | I | -2.46 | Neutral | rs2230017 |
| 5,79950718,G,C | GCC [G/C]CA GCG | 58 | A | P | 1.64 | Neutral | rs148550291 |
| 5,79950724,G,C | GCG [G/C]CC GCA | 60 | A | P | 0.72 | Neutral | rs2001675 |
| 5,79950708,T,C | GCG GC[T/C] GCA | 54 | A | A | 0 | Neutral | rs2405875 |
| 5,79950708,T,C | GCG GC[T/C] GCA | 54 | A | A | 0 | Neutral | rs2405875 |
| 5,79950715,G,C | GCG [G/C]CC GCA | 57 | A | P | 0.48 | Neutral | rs144776112 |
| 5,131977996,C,T | AAC AT[C/T] GAT | 1293 | I | I | 0 | Neutral | rs28903094 |
| 5,131977996,C,T | AAC AT[C/T] GAT | 1154 | I | I | 0 | Neutral | rs28903094 |
| 5,80149981,A,G | GGA C[A/G]G AGT | 949 | Q | R | 0.58 | Neutral | rs184967 |
| 5,80149981,A,G | GGA C[A/G]G AGT | 940 | Q | R | 0.58 | Neutral | rs184967 |
| 5,80168937,G,A | GGC [G/A]CA GCA | 1045 | A | T | -0.1 | Neutral | rs26279 |
| 5,80168937,G,A | GGC [G/A]CA GCA | 1036 | A | T | -0.1 | Neutral | rs26279 |
| 5,68531253,C,T | AAG AA[C/T] ACC | 33 | N | N | 0 | Neutral | rs2972388 |
| 5,68531253,C,T | AAG AA[C/T] ACC | 33 | N | N | 0 | Neutral | rs2972388 |
| 5,68531253,C,T | AAG AA[C/T] ACC | 33 | N | N | 0 | Neutral | rs2972388 |
| 5,68531253,C,T | AAG AA[C/T] ACC | 33 | N | N | 0 | Neutral | rs2972388 |
| 5,68531253,C,T | AAG AA[C/T] ACC | 33 | N | N | 0 | Neutral | rs2972388 |
| 5,68531253,C,T | AAG AA[C/T] ACC | 33 | N | N | 0 | Neutral | rs2972388 |
| 5,68531253,C,T | AAG AA[C/T] ACC | 33 | N | N | 0 | Neutral | rs2972388 |
| 5,68531253,C,T | AAG AA[C/T] ACC | 33 | N | N | 0 | Neutral | rs2972388 |
| 5,131977963,T,C | GAA TA[T/C] GTG | 1282 | Y | Y | 0 | Neutral | rs1804670 |
| 5,131977963,T,C | GAA TA[T/C] GTG | 1143 | Y | Y | 0 | Neutral | rs1804670 |
| 5,137802651,G,C | TCC TC[G/C] TCC | 171 | S | S | 0 | Neutral | rs11953917 |
| 5,137802651,G,C | TCC TC[G/C] TCC | 171 | S | S | 0 | Neutral | rs11953917 |
| 5,131977963,T,C | GAA TA[T/C] GTG | 1282 | Y | Y | 0 | Neutral | rs1804670 |
| 5,131977963,T,C | GAA TA[T/C] GTG | 1143 | Y | Y | 0 | Neutral | rs1804670 |
| 5,86703905,T,C | GAG A[A/G]G GCA | 138 | K | R | -1.56 | Neutral | rs2266691 |
| 5,86703905,T,C | GAG A[A/G]G GCA | 64 | K | R | -1.6 | Neutral | rs2266691 |
| 5,86703905,T,C | GAG A[A/G]G GCA | 64 | K | R | -1.56 | Neutral | rs2266691 |
| 5,131951811,G,A | GTT TT[G/A] CAA | 1051 | L | L | 0 | Neutral | rs35800931 |
| 5,131951811,G,A | GTT TT[G/A] CAA | 912 | L | L | 0 | Neutral | rs35800931 |
| 5,80149981,A,G | GGA C[A/G]G AGT | 949 | Q | R | 0.58 | Neutral | rs184967 |
| 5,80149981,A,G | GGA C[A/G]G AGT | 940 | Q | R | 0.58 | Neutral | rs184967 |
| 5,131923338,C,A | AAG [C/A]AA ATG | 281 | Q | K | -0.8 | Neutral |  |
| 5,131923338,C,A | AAG [C/A]AA ATG | 142 | Q | K | -0.54 | Neutral |  |
| 5,131923338,C,A | AAG [C/A]AA ATG | 281 | Q | K | -1.54 | Neutral |  |
| 5,131923338,C,A | AAG [C/A]AA ATG | 281 | Q | K | -1.29 | Neutral |  |
| 5,80149981,A,G | GGA C[A/G]G AGT | 949 | Q | R | 0.58 | Neutral | rs184967 |
| 5,80149981,A,G | GGA C[A/G]G AGT | 940 | Q | R | 0.58 | Neutral | rs184967 |
| 5,86703905,T,C | GAG A[A/G]G GCA | 138 | K | R | -1.56 | Neutral | rs2266691 |
| 5,86703905,T,C | GAG A[A/G]G GCA | 64 | K | R | -1.6 | Neutral | rs2266691 |
| 5,86703905,T,C | GAG A[A/G]G GCA | 64 | K | R | -1.56 | Neutral | rs2266691 |
| 5,131951811,G,A | GTT TT[G/A] CAA | 1051 | L | L | 0 | Neutral | rs35800931 |
| 5,131951811,G,A | GTT TT[G/A] CAA | 912 | L | L | 0 | Neutral | rs35800931 |
| 5,80149981,A,G | GGA C[A/G]G AGT | 949 | Q | R | 0.58 | Neutral | rs184967 |
| 5,80149981,A,G | GGA C[A/G]G AGT | 940 | Q | R | 0.58 | Neutral | rs184967 |
| 5,86707121,T,C | GAA [A/G]TG ACA | 54 | M | V | -1.19 | Neutral | rs3093785 |
| 5,131923338,C,A | AAG [C/A]AA ATG | 281 | Q | K | -0.8 | Neutral |  |
| 5,131923338,C,A | AAG [C/A]AA ATG | 142 | Q | K | -0.54 | Neutral |  |
| 5,131923338,C,A | AAG [C/A]AA ATG | 281 | Q | K | -1.54 | Neutral |  |
| 5,131923338,C,A | AAG [C/A]AA ATG | 281 | Q | K | -1.29 | Neutral |  |
| 5,80149981,A,G | GGA C[A/G]G AGT | 949 | Q | R | 0.58 | Neutral | rs184967 |
| 5,80149981,A,G | GGA C[A/G]G AGT | 940 | Q | R | 0.58 | Neutral | rs184967 |
| 5,137802651,G,C | TCC TC[G/C] TCC | 171 | S | S | 0 | Neutral | rs11953917 |
| 5,137802651,G,C | TCC TC[G/C] TCC | 171 | S | S | 0 | Neutral | rs11953917 |
| 5,60200665,A,G | GTT TA[T/C] AGT | 145 | Y | Y | 0 | Neutral | rs4647100 |
| 5,60200665,A,G | GTT TA[T/C] AGT | 87 | Y | Y | 0 | Neutral | rs4647100 |
| 5,60200665,A,G | GTT TA[T/C] AGT | 87 | Y | Y | 0 | Neutral | rs4647100 |
| 5,60200665,A,G | GTT TA[T/C] AGT | 144 | Y | Y | 0 | Neutral | rs4647100 |
| 5,131977963,T,C | GAA TA[T/C] GTG | 1282 | Y | Y | 0 | Neutral | rs1804670 |
| 5,131977963,T,C | GAA TA[T/C] GTG | 1143 | Y | Y | 0 | Neutral | rs1804670 |
| 6,43582091,A,T | GAT [A/T]TG CCA | 647 | M | L | -0.23 | Neutral | rs6941583 |
| 6,43582091,A,T | GAT [A/T]TG CCA | 585 | M | L | -0.29 | Neutral | rs6941583 |
| 6,43581586,G,A | ACC AC[G/A] TCT | 478 | T | T | 0 | Neutral | rs3734690 |
| 6,43581586,G,A | ACC AC[G/A] TCT | 416 | T | T | 0 | Neutral | rs3734690 |
| 6,30877760,A,C | CAC AC[A/C] CAG | 98 | T | T | 0 | Neutral | rs114336365 |
| 6,30877760,A,C | CAC AC[A/C] CAG | 98 | T | T | 0 | Neutral | rs114336365 |
| 6,30877760,A,C | CAC AC[A/C] CAG | 98 | T | T | 0 | Neutral | rs114336365 |
| 6,30877760,A,C | CAC AC[A/C] CAG | 42 | T | T | 0 | Neutral | rs114336365 |
| 6,30881103,C,A | CCC CC[C/A] ACC | 385 | P | P | 0 | Neutral | rs1132408 |
| 6,30881103,C,A | CCC CC[C/A] ACC | 385 | P | P | 0 | Neutral | rs1132408 |
| 6,30877760,A,C | CAC AC[A/C] CAG | 98 | T | T | 0 | Neutral | rs114336365 |
| 6,30877760,A,C | CAC AC[A/C] CAG | 98 | T | T | 0 | Neutral | rs114336365 |
| 6,30877760,A,C | CAC AC[A/C] CAG | 98 | T | T | 0 | Neutral | rs114336365 |
| 6,30877760,A,C | CAC AC[A/C] CAG | 42 | T | T | 0 | Neutral | rs114336365 |
| 6,36651971,C,A | CTG AG[C/A] CGC | 31 | S | R | 0.79 | Neutral | rs1801270 |
| 6,36651971,C,A | CTG AG[C/A] CGC | 31 | S | R | 0.79 | Neutral | rs1801270 |
| 6,36651971,C,A | CTG AG[C/A] CGC | 31 | S | R | 0.79 | Neutral | rs1801270 |
| 6,36651971,C,A | CTG AG[C/A] CGC | 65 | S | R | 0.82 | Neutral | rs1801270 |
| 6,30877760,A,C | CAC AC[A/C] CAG | 98 | T | T | 0 | Neutral | rs114336365 |
| 6,30877760,A,C | CAC AC[A/C] CAG | 98 | T | T | 0 | Neutral | rs114336365 |
| 6,30877760,A,C | CAC AC[A/C] CAG | 98 | T | T | 0 | Neutral | rs114336365 |
| 6,30877760,A,C | CAC AC[A/C] CAG | 42 | T | T | 0 | Neutral | rs114336365 |
| 6,30880193,G,T | CGG GA[G/T] AGT | 349 | E | D | 0.15 | Neutral |  |
| 6,30880193,G,T | CGG GA[G/T] AGT | 349 | E | D | 0.15 | Neutral |  |
| 6,36651971,C,A | CTG AG[C/A] CGC | 31 | S | R | 0.79 | Neutral | rs1801270 |
| 6,36651971,C,A | CTG AG[C/A] CGC | 31 | S | R | 0.79 | Neutral | rs1801270 |
| 6,36651971,C,A | CTG AG[C/A] CGC | 31 | S | R | 0.79 | Neutral | rs1801270 |
| 6,36651971,C,A | CTG AG[C/A] CGC | 65 | S | R | 0.82 | Neutral | rs1801270 |
| 6,30877760,A,C | CAC AC[A/C] CAG | 98 | T | T | 0 | Neutral | rs114336365 |
| 6,30877760,A,C | CAC AC[A/C] CAG | 98 | T | T | 0 | Neutral | rs114336365 |
| 6,30877760,A,C | CAC AC[A/C] CAG | 98 | T | T | 0 | Neutral | rs114336365 |
| 6,30877760,A,C | CAC AC[A/C] CAG | 42 | T | T | 0 | Neutral | rs114336365 |
| 6,30880193,G,T | CGG GA[G/T] AGT | 349 | E | D | 0.15 | Neutral |  |
| 6,30880193,G,T | CGG GA[G/T] AGT | 349 | E | D | 0.15 | Neutral |  |
| 6,43582180,C,T | TCT GC[C/T] GTA | 676 | A | A | 0 | Neutral | rs140971385 |
| 6,43582180,C,T | TCT GC[C/T] GTA | 614 | A | A | 0 | Neutral | rs140971385 |
| 6,43581724,A,C | AGT CA[A/C] AGT | 524 | Q | H | -1.05 | Neutral | rs138752565 |
| 6,43581724,A,C | AGT CA[A/C] AGT | 462 | Q | H | -1.08 | Neutral | rs138752565 |
| 6,20490428,G,A | AGC [G/A]AT TGC | 389 | D | N | -0.01 | Neutral | rs4134982 |
| 6,20490428,G,A | AGC [G/A]AT TGC | 258 | D | N | 0.05 | Neutral | rs4134982 |
| 6,30881103,C,A | CCC CC[C/A] ACC | 385 | P | P | 0 | Neutral | rs1132408 |
| 6,30881103,C,A | CCC CC[C/A] ACC | 385 | P | P | 0 | Neutral | rs1132408 |
| 6,20490428,G,A | AGC [G/A]AT TGC | 389 | D | N | -0.01 | Neutral | rs4134982 |
| 6,20490428,G,A | AGC [G/A]AT TGC | 258 | D | N | 0.05 | Neutral | rs4134982 |
| 7,6013049,C,G | CTG G[G/C]T GTC | 857 | G | A | -0.79 | Neutral | rs1802683 |
| 7,6013049,C,G | CTG G[G/C]T GTC | 456 | G | A | -0.62 | Neutral | rs1802683 |
| 7,6013049,C,G | CTG G[G/C]T GTC | 810 | G | A | -0.89 | Neutral | rs1802683 |
| 7,6013049,C,G | CTG G[G/C]T GTC | 751 | G | A | -0.59 | Neutral | rs1802683 |
| 7,6026988,G,A | AGA [C/T]CT CAG | 470 | P | S | 0.2 | Neutral | rs1805321 |
| 7,6026988,G,A | AGA [C/T]CT CAG | 423 | P | S | 0.36 | Neutral | rs1805321 |
| 7,6026988,G,A | AGA [C/T]CT CAG | 470 | P | S | 0.27 | Neutral | rs1805321 |
| 7,6026988,G,A | AGA [C/T]CT CAG | 364 | P | S | 0.11 | Neutral | rs1805321 |
| 7,6026988,G,A | AGA [C/T]CT CAG | 470 | P | S | 0.2 | Neutral | rs1805321 |
| 7,6026988,G,A | AGA [C/T]CT CAG | 423 | P | S | 0.36 | Neutral | rs1805321 |
| 7,6026988,G,A | AGA [C/T]CT CAG | 470 | P | S | 0.27 | Neutral | rs1805321 |
| 7,6026988,G,A | AGA [C/T]CT CAG | 364 | P | S | 0.11 | Neutral | rs1805321 |
| 7,6026775,T,C | CCT [A/G]AA ACT | 541 | K | E | 0.76 | Neutral | rs2228006 |
| 7,6026775,T,C | CCT [A/G]AA ACT | 494 | K | E | 0.82 | Neutral | rs2228006 |
| 7,6026775,T,C | CCT [A/G]AA ACT | 541 | K | E | 1.25 | Neutral | rs2228006 |
| 7,6026775,T,C | CCT [A/G]AA ACT | 435 | K | E | 0.78 | Neutral | rs2228006 |
| 7,6026988,G,A | AGA [C/T]CT CAG | 470 | P | S | 0.2 | Neutral | rs1805321 |
| 7,6026988,G,A | AGA [C/T]CT CAG | 423 | P | S | 0.36 | Neutral | rs1805321 |
| 7,6026988,G,A | AGA [C/T]CT CAG | 470 | P | S | 0.27 | Neutral | rs1805321 |
| 7,6026988,G,A | AGA [C/T]CT CAG | 364 | P | S | 0.11 | Neutral | rs1805321 |
| 7,6022617,G,A | AAA A[C/T]G ATG | 671 | T | M | -1.85 | Neutral |  |
| 7,6022617,G,A | AAA A[C/T]G ATG | 270 | T | M | -1.86 | Neutral |  |
| 7,6022617,G,A | AAA A[C/T]G ATG | 624 | T | M | -1.88 | Neutral |  |
| 7,6022617,G,A | AAA A[C/T]G ATG | 565 | T | M | -1.75 | Neutral |  |
| 7,6026988,G,A | AGA [C/T]CT CAG | 470 | P | S | 0.2 | Neutral | rs1805321 |
| 7,6026988,G,A | AGA [C/T]CT CAG | 423 | P | S | 0.36 | Neutral | rs1805321 |
| 7,6026988,G,A | AGA [C/T]CT CAG | 470 | P | S | 0.27 | Neutral | rs1805321 |
| 7,6026988,G,A | AGA [C/T]CT CAG | 364 | P | S | 0.11 | Neutral | rs1805321 |
| 7,6013153,A,G | GCT CT[T/C] AAC | 822 | L | L | 0 | Neutral | rs10000 |
| 7,6013153,A,G | GCT CT[T/C] AAC | 421 | L | L | 0 | Neutral | rs10000 |
| 7,6013153,A,G | GCT CT[T/C] AAC | 775 | L | L | 0 | Neutral | rs10000 |
| 7,6013153,A,G | GCT CT[T/C] AAC | 716 | L | L | 0 | Neutral | rs10000 |
| 7,6026864,G,A | GAC A[C/T]G GGC | 511 | T | M | -0.87 | Neutral | rs74902811 |
| 7,6026864,G,A | GAC A[C/T]G GGC | 464 | T | M | -0.87 | Neutral | rs74902811 |
| 7,6026864,G,A | GAC A[C/T]G GGC | 511 | T | M | -0.07 | Neutral | rs74902811 |
| 7,6026864,G,A | GAC A[C/T]G GGC | 405 | T | M | -0.99 | Neutral | rs74902811 |
| 7,6026988,G,A | AGA [C/T]CT CAG | 470 | P | S | 0.2 | Neutral | rs1805321 |
| 7,6026988,G,A | AGA [C/T]CT CAG | 423 | P | S | 0.36 | Neutral | rs1805321 |
| 7,6026988,G,A | AGA [C/T]CT CAG | 470 | P | S | 0.27 | Neutral | rs1805321 |
| 7,6026988,G,A | AGA [C/T]CT CAG | 364 | P | S | 0.11 | Neutral | rs1805321 |
| 7,6013153,A,G | GCT CT[T/C] AAC | 822 | L | L | 0 | Neutral | rs10000 |
| 7,6013153,A,G | GCT CT[T/C] AAC | 421 | L | L | 0 | Neutral | rs10000 |
| 7,6013153,A,G | GCT CT[T/C] AAC | 775 | L | L | 0 | Neutral | rs10000 |
| 7,6013153,A,G | GCT CT[T/C] AAC | 716 | L | L | 0 | Neutral | rs10000 |
| 7,6013153,A,G | GCT CT[T/C] AAC | 822 | L | L | 0 | Neutral | rs10000 |
| 7,6013153,A,G | GCT CT[T/C] AAC | 421 | L | L | 0 | Neutral | rs10000 |
| 7,6013153,A,G | GCT CT[T/C] AAC | 775 | L | L | 0 | Neutral | rs10000 |
| 7,6013153,A,G | GCT CT[T/C] AAC | 716 | L | L | 0 | Neutral | rs10000 |
| 7,6013049,C,G | CTG G[G/C]T GTC | 857 | G | A | -0.79 | Neutral | rs1802683 |
| 7,6013049,C,G | CTG G[G/C]T GTC | 456 | G | A | -0.62 | Neutral | rs1802683 |
| 7,6013049,C,G | CTG G[G/C]T GTC | 810 | G | A | -0.89 | Neutral | rs1802683 |
| 7,6013049,C,G | CTG G[G/C]T GTC | 751 | G | A | -0.59 | Neutral | rs1802683 |
| 7,6026530,C,T | TCT AT[G/A] AGT | 622 | M | I | -0.96 | Neutral | rs1805324 |
| 7,6026530,C,T | TCT AT[G/A] AGT | 575 | M | I | -1.06 | Neutral | rs1805324 |
| 7,6026530,C,T | TCT AT[G/A] AGT | 516 | M | I | -1.08 | Neutral | rs1805324 |
| 7,6026775,T,C | CCT [A/G]AA ACT | 541 | K | E | 0.76 | Neutral | rs2228006 |
| 7,6026775,T,C | CCT [A/G]AA ACT | 494 | K | E | 0.82 | Neutral | rs2228006 |
| 7,6026775,T,C | CCT [A/G]AA ACT | 541 | K | E | 1.25 | Neutral | rs2228006 |
| 7,6026775,T,C | CCT [A/G]AA ACT | 435 | K | E | 0.78 | Neutral | rs2228006 |
| 8,90990479,C,G | GTT [G/C]AG TCC | 185 | E | Q | 0.53 | Neutral | rs1805794 |
| 8,90990479,C,G | GTT [G/C]AG TCC | 103 | E | Q | 0.56 | Neutral | rs1805794 |
| 8,90990479,C,G | GTT [G/C]AG TCC | 103 | E | Q | 0.69 | Neutral | rs1805794 |
| 8,90990479,C,G | GTT [G/C]AG TCC | 185 | E | Q | 0.63 | Neutral | rs1805794 |
| 8,90995019,C,T | ATT CT[G/A] ATT | 34 | L | L | 0 | Neutral | rs1063045 |
| 8,90995019,C,T | ATT CT[G/A] ATT | 34 | L | L | 0 | Neutral | rs1063045 |
| 8,90995019,C,T | ATT CT[G/A] ATT | 34 | L | L | 0 | Neutral | rs1063045 |
| 8,90995019,C,T | ATT CT[G/A] ATT | 34 | L | L | 0 | Neutral | rs1063045 |
| 8,90995019,C,T | ATT CT[G/A] ATT | 34 | L | L | 0 | Neutral | rs1063045 |
| 8,90967711,A,G | CAA GA[T/C] GCA | 399 | D | D | 0 | Neutral | rs709816 |
| 8,90967711,A,G | CAA GA[T/C] GCA | 317 | D | D | 0 | Neutral | rs709816 |
| 8,90967711,A,G | CAA GA[T/C] GCA | 399 | D | D | 0 | Neutral | rs709816 |
| 8,22548790,G,T | GCG CT[C/A] AGC | 120 | L | L | 0 | Neutral | rs3750192 |
| 8,22548790,G,T | GCG CT[C/A] AGC | 82 | L | L | 0 | Neutral | rs3750192 |
| 8,90967711,A,G | CAA GA[T/C] GCA | 399 | D | D | 0 | Neutral | rs709816 |
| 8,90967711,A,G | CAA GA[T/C] GCA | 317 | D | D | 0 | Neutral | rs709816 |
| 8,90967711,A,G | CAA GA[T/C] GCA | 399 | D | D | 0 | Neutral | rs709816 |
| 8,11640784,A,G | TCC CC[A/G] GTG | 188 | P | P | 0 | Neutral | rs8191642 |
| 8,11640784,A,G | TCC CC[A/G] GTG | 173 | P | P | 0 | Neutral | rs8191642 |
| 8,11640784,A,G | TCC CC[A/G] GTG | 127 | P | P | 0 | Neutral | rs8191642 |
| 8,11640784,A,G | TCC CC[A/G] GTG | 188 | P | P | 0 | Neutral | rs8191642 |
| 8,11640784,A,G | TCC CC[A/G] GTG | 188 | P | P | 0 | Neutral | rs8191642 |
| 8,11640784,A,G | TCC CC[A/G] GTG | 72 | P | P | 0 | Neutral | rs8191642 |
| 8,42220154,G,A | GTG [G/A]AG CAG | 216 | E | K | -1.25 | Neutral |  |
| 8,42220154,G,A | GTG [G/A]AG CAG | 32 | E | K | -1.31 | Neutral |  |
| 8,42220154,G,A | GTG [G/A]AG CAG | 74 | E | K | -1.16 | Neutral |  |
| 8,42220154,G,A | GTG [G/A]AG CAG | 251 | E | K | -1.11 | Neutral |  |
| 8,42220154,G,A | GTG [G/A]AG CAG | 62 | E | K | -1.16 | Neutral |  |
| 8,22548525,G,T | GAG [C/A]AC AAG | 209 | H | N | -1.35 | Neutral |  |
| 8,22548525,G,T | GAG [C/A]AC AAG | 50 | H | N | -0.57 | Neutral |  |
| 8,22548525,G,T | GAG [C/A]AC AAG | 171 | H | N | -1.49 | Neutral |  |
| 8,42220154,G,A | GTG [G/A]AG CAG | 216 | E | K | -1.25 | Neutral |  |
| 8,42220154,G,A | GTG [G/A]AG CAG | 32 | E | K | -1.31 | Neutral |  |
| 8,42220154,G,A | GTG [G/A]AG CAG | 74 | E | K | -1.16 | Neutral |  |
| 8,42220154,G,A | GTG [G/A]AG CAG | 251 | E | K | -1.11 | Neutral |  |
| 8,42220154,G,A | GTG [G/A]AG CAG | 62 | E | K | -1.16 | Neutral |  |
| 8,11640784,A,G | TCC CC[A/G] GTG | 188 | P | P | 0 | Neutral | rs8191642 |
| 8,11640784,A,G | TCC CC[A/G] GTG | 173 | P | P | 0 | Neutral | rs8191642 |
| 8,11640784,A,G | TCC CC[A/G] GTG | 127 | P | P | 0 | Neutral | rs8191642 |
| 8,11640784,A,G | TCC CC[A/G] GTG | 188 | P | P | 0 | Neutral | rs8191642 |
| 8,11640784,A,G | TCC CC[A/G] GTG | 188 | P | P | 0 | Neutral | rs8191642 |
| 8,11640784,A,G | TCC CC[A/G] GTG | 72 | P | P | 0 | Neutral | rs8191642 |
| 8,42220154,G,A | GTG [G/A]AG CAG | 216 | E | K | -1.25 | Neutral |  |
| 8,42220154,G,A | GTG [G/A]AG CAG | 32 | E | K | -1.31 | Neutral |  |
| 8,42220154,G,A | GTG [G/A]AG CAG | 74 | E | K | -1.16 | Neutral |  |
| 8,42220154,G,A | GTG [G/A]AG CAG | 251 | E | K | -1.11 | Neutral |  |
| 8,42220154,G,A | GTG [G/A]AG CAG | 62 | E | K | -1.16 | Neutral |  |
| 8,22548525,G,T | GAG [C/A]AC AAG | 209 | H | N | -1.35 | Neutral |  |
| 8,22548525,G,T | GAG [C/A]AC AAG | 50 | H | N | -0.57 | Neutral |  |
| 8,22548525,G,T | GAG [C/A]AC AAG | 171 | H | N | -1.49 | Neutral |  |
| 8,90967711,A,G | CAA GA[T/C] GCA | 399 | D | D | 0 | Neutral | rs709816 |
| 8,90967711,A,G | CAA GA[T/C] GCA | 317 | D | D | 0 | Neutral | rs709816 |
| 8,90967711,A,G | CAA GA[T/C] GCA | 399 | D | D | 0 | Neutral | rs709816 |
| 8,42220154,G,A | GTG [G/A]AG CAG | 216 | E | K | -1.25 | Neutral |  |
| 8,42220154,G,A | GTG [G/A]AG CAG | 32 | E | K | -1.31 | Neutral |  |
| 8,42220154,G,A | GTG [G/A]AG CAG | 74 | E | K | -1.16 | Neutral |  |
| 8,42220154,G,A | GTG [G/A]AG CAG | 251 | E | K | -1.11 | Neutral |  |
| 8,42220154,G,A | GTG [G/A]AG CAG | 62 | E | K | -1.16 | Neutral |  |
| 8,90967711,A,G | CAA GA[T/C] GCA | 399 | D | D | 0 | Neutral | rs709816 |
| 8,90967711,A,G | CAA GA[T/C] GCA | 317 | D | D | 0 | Neutral | rs709816 |
| 8,90967711,A,G | CAA GA[T/C] GCA | 399 | D | D | 0 | Neutral | rs709816 |
| 8,90967711,A,G | CAA GA[T/C] GCA | 399 | D | D | 0 | Neutral | rs709816 |
| 8,90967711,A,G | CAA GA[T/C] GCA | 317 | D | D | 0 | Neutral | rs709816 |
| 8,90967711,A,G | CAA GA[T/C] GCA | 399 | D | D | 0 | Neutral | rs709816 |
| 9,110068767,C,T | CCA AC[C/T] CCT | 112 | T | T | 0 | Neutral | rs1805333 |
| 9,110068767,C,T | CCA AC[C/T] CCT | 40 | T | T | 0 | Neutral | rs1805333 |
| 9,110068767,C,T | CCA AC[C/T] CCT | 40 | T | T | 0 | Neutral | rs1805333 |
| 9,110068767,C,T | CCA AC[C/T] CCT | 112 | T | T | 0 | Neutral | rs1805333 |
| 9,133761001,A,G | GGT CC[A/G] GCG | 1108 | P | P | 0 | Neutral | rs1056171 |
| 9,133761001,A,G | GGT CC[A/G] GCG | 1127 | P | P | 0 | Neutral | rs1056171 |
| 9,133761001,A,G | GGT CC[A/G] GCG | 923 | P | P | 0 | Neutral | rs1056171 |
| 9,133761001,A,G | GGT CC[A/G] GCG | 1108 | P | P | 0 | Neutral | rs1056171 |
| 9,133761001,A,G | GGT CC[A/G] GCG | 1127 | P | P | 0 | Neutral | rs1056171 |
| 9,133761001,A,G | GGT CC[A/G] GCG | 923 | P | P | 0 | Neutral | rs1056171 |
| 9,133759546,G,A | CGG GA[G/A] ATG | 623 | E | E | 0 | Neutral | rs34717358 |
| 9,133759546,G,A | CGG GA[G/A] ATG | 642 | E | E | 0 | Neutral | rs34717358 |
| 9,133759546,G,A | CGG GA[G/A] ATG | 438 | E | E | 0 | Neutral | rs34717358 |
| 9,133760592,C,T | CCG T[C/T]G GGG | 972 | S | L | -0.61 | Neutral | rs2229067 |
| 9,133760592,C,T | CCG T[C/T]G GGG | 991 | S | L | -0.61 | Neutral | rs2229067 |
| 9,133760592,C,T | CCG T[C/T]G GGG | 787 | S | L | -0.96 | Neutral | rs2229067 |
| 9,110068767,C,T | CCA AC[C/T] CCT | 112 | T | T | 0 | Neutral | rs1805333 |
| 9,110068767,C,T | CCA AC[C/T] CCT | 40 | T | T | 0 | Neutral | rs1805333 |
| 9,110068767,C,T | CCA AC[C/T] CCT | 40 | T | T | 0 | Neutral | rs1805333 |
| 9,110068767,C,T | CCA AC[C/T] CCT | 112 | T | T | 0 | Neutral | rs1805333 |
| 9,133761001,A,G | GGT CC[A/G] GCG | 1108 | P | P | 0 | Neutral | rs1056171 |
| 9,133761001,A,G | GGT CC[A/G] GCG | 1127 | P | P | 0 | Neutral | rs1056171 |
| 9,133761001,A,G | GGT CC[A/G] GCG | 923 | P | P | 0 | Neutral | rs1056171 |
| 9,21974738,G,A | CGG G[C/T]G CTG | 30 | A | V | -1.36 | Neutral |  |
| 9,21974738,G,A | CGG G[C/T]G CTG | 30 | A | V | -1.68 | Neutral |  |
| 9,21974738,G,A | CGG G[C/T]G CTG | 30 | A | V | -1.37 | Neutral |  |
| 9,21974738,G,A | CGG G[C/T]G CTG | 30 | A | V | -1.37 | Neutral |  |
| 9,110068767,C,T | CCA AC[C/T] CCT | 112 | T | T | 0 | Neutral | rs1805333 |
| 9,110068767,C,T | CCA AC[C/T] CCT | 40 | T | T | 0 | Neutral | rs1805333 |
| 9,110068767,C,T | CCA AC[C/T] CCT | 40 | T | T | 0 | Neutral | rs1805333 |
| 9,110068767,C,T | CCA AC[C/T] CCT | 112 | T | T | 0 | Neutral | rs1805333 |
| 9,133761001,A,G | GGT CC[A/G] GCG | 1108 | P | P | 0 | Neutral | rs1056171 |
| 9,133761001,A,G | GGT CC[A/G] GCG | 1127 | P | P | 0 | Neutral | rs1056171 |
| 9,133761001,A,G | GGT CC[A/G] GCG | 923 | P | P | 0 | Neutral | rs1056171 |
| 9,21974738,G,A | CGG G[C/T]G CTG | 30 | A | V | -1.36 | Neutral |  |
| 9,21974738,G,A | CGG G[C/T]G CTG | 30 | A | V | -1.68 | Neutral |  |
| 9,21974738,G,A | CGG G[C/T]G CTG | 30 | A | V | -1.37 | Neutral |  |
| 9,21974738,G,A | CGG G[C/T]G CTG | 30 | A | V | -1.37 | Neutral |  |
| 9,110068767,C,T | CCA AC[C/T] CCT | 112 | T | T | 0 | Neutral | rs1805333 |
| 9,110068767,C,T | CCA AC[C/T] CCT | 40 | T | T | 0 | Neutral | rs1805333 |
| 9,110068767,C,T | CCA AC[C/T] CCT | 40 | T | T | 0 | Neutral | rs1805333 |
| 9,110068767,C,T | CCA AC[C/T] CCT | 112 | T | T | 0 | Neutral | rs1805333 |
| 9,133760808,C,A | ATC T[C/A]T AGG | 859 | S | Y | -2.41 | Neutral |  |
| 9,110084328,C,T | GGG G[C/T]T CCT | 249 | A | V | -0.99 | Neutral | rs1805329 |
| 9,110084328,C,T | GGG G[C/T]T CCT | 177 | A | V | -0.96 | Neutral | rs1805329 |
| 9,133761001,A,G | GGT CC[A/G] GCG | 1108 | P | P | 0 | Neutral | rs1056171 |
| 9,133761001,A,G | GGT CC[A/G] GCG | 1127 | P | P | 0 | Neutral | rs1056171 |
| 9,133761001,A,G | GGT CC[A/G] GCG | 923 | P | P | 0 | Neutral | rs1056171 |
| 9,133755528,A,G | CAG GA[A/G] TCC | 499 | E | E | 0 | Neutral | rs2227985 |
| 9,133755528,A,G | CAG GA[A/G] TCC | 518 | E | E | 0 | Neutral | rs2227985 |
| 9,133755528,A,G | CAG GA[A/G] TCC | 314 | E | E | 0 | Neutral | rs2227985 |
| x,66765627,G,A | AGG GA[G/A] GCC | 213 | E | E | 0 | Neutral | rs6152 |
| x,66765627,G,A | AGG GA[G/A] GCC | 213 | E | E | 0 | Neutral | rs6152 |
| x,66765627,G,A | AGG GA[G/A] GCC | 213 | E | E | 0 | Neutral | rs6152 |
| x,66765627,G,A | AGG GA[G/A] GCC | 213 | E | E | 0 | Neutral | rs6152 |
| x,66765627,G,A | AGG GA[G/A] GCC | 23 | E | E | 0 | Neutral | rs6152 |
| x,66765627,G,A | AGG GA[G/A] GCC | 205 | E | E | 0 | Neutral | rs6152 |
| x,66765627,G,A | AGG GA[G/A] GCC | 213 | E | E | 0 | Neutral | rs6152 |
| x,66765627,G,A | AGG GA[G/A] GCC | 213 | E | E | 0 | Neutral | rs6152 |
| x,66765627,G,A | AGG GA[G/A] GCC | 213 | E | E | 0 | Neutral | rs6152 |
| x,66765627,G,A | AGG GA[G/A] GCC | 213 | E | E | 0 | Neutral | rs6152 |
| x,66765627,G,A | AGG GA[G/A] GCC | 23 | E | E | 0 | Neutral | rs6152 |
| x,66765627,G,A | AGG GA[G/A] GCC | 205 | E | E | 0 | Neutral | rs6152 |
| x,96139406,G,A | CCT [G/A]CT ATT | 33 | A | T | -0.4 | Neutral | rs2642219 |
| x,96139459,T,C | TGT AA[T/C] GTG | 50 | N | N | 0 | Neutral | rs2642218 |
| x,96139459,T,C | TGT AA[T/C] GTG | 50 | N | N | 0 | Neutral | rs2642218 |
| x,96139459,T,C | TGT AA[T/C] GTG | 50 | N | N | 0 | Neutral | rs2642218 |
| x,96139459,T,C | TGT AA[T/C] GTG | 50 | N | N | 0 | Neutral | rs2642218 |
| x,66765627,G,A | AGG GA[G/A] GCC | 213 | E | E | 0 | Neutral | rs6152 |
| x,66765627,G,A | AGG GA[G/A] GCC | 213 | E | E | 0 | Neutral | rs6152 |
| x,66765627,G,A | AGG GA[G/A] GCC | 213 | E | E | 0 | Neutral | rs6152 |
| x,66765627,G,A | AGG GA[G/A] GCC | 213 | E | E | 0 | Neutral | rs6152 |
| x,66765627,G,A | AGG GA[G/A] GCC | 23 | E | E | 0 | Neutral | rs6152 |
| x,66765627,G,A | AGG GA[G/A] GCC | 205 | E | E | 0 | Neutral | rs6152 |
| 1,133237569,C,T |  |  |  |  |  |  |  |
| 13,108860883,A,T |  |  |  |  |  |  |  |
