# Supplementary material for: Somatic mutations in the DNA repairome in prostate cancers in African Americans and Caucasians
Source: Oncogene. 2020 Apr 16;39(21):4299–311. doi: 10.1038/s41388-020-1280-x (PMC7239769; doi:10.1038/s41388-020-1280-x)
Supplement: Supplementary file 5 — Supplementary data 4 [file 41388_2020_1280_MOESM5_ESM.docx]

**Supplementary data 4.**

| \| INPUT \| \| --- \| | CODON_CHANGE | POS | RESIDUE_REF | RESIDUE_ALT | SCORE | PREDICTION (cutoff=-2.5) | dbSNP_ID |
| --- | --- | --- | --- | --- | --- | --- | --- | --- |
| 8,42218831,A,C | GGT G[A/C]C ATG | 190 | D | A | -6.68 | Deleterious |  |
|  | GGT G[A/C]C ATG | 6 | D | A | -8 | Deleterious |  |
|  | GGT G[A/C]C ATG | 121 | D | A | -6.42 | Deleterious |  |
|  | GGT G[A/C]C ATG | 48 | D | A | -6.78 | Deleterious |  |
|  | GGT G[A/C]C ATG | 36 | D | A | -7.73 | Deleterious |  |
|  | GGT G[A/C]C ATG | 225 | D | A | -6.94 | Deleterious |  |
|  | GGT G[A/C]C ATG | 36 | D | A | -6.75 | Deleterious |  |
| 11,125523641,G,T | CAG [G/T]TT ACT | 368 | V | F | -2.51 | Deleterious |  |
|  | CAG [G/T]TT ACT | 412 | V | F | -2.56 | Deleterious |  |
|  | CAG [G/T]TT ACT | 428 | V | F | -2.81 | Deleterious |  |
|  | CAG [G/T]TT ACT | 412 | V | F | -2.56 | Deleterious |  |
|  | CAG [G/T]TT ACT | 412 | V | F | -2.56 | Deleterious |  |
|  | CAG [G/T]TT ACT | 412 | V | F | -2.56 | Deleterious |  |
| 15,91326099,C,T | TTA C[C/T]G AAA | 868 | P | L | -6.25 | Deleterious | [rs11852361](http://www.ncbi.nlm.nih.gov/projects/SNP/snp_ref.cgi?rs=rs11852361) |
|  | TTA C[C/T]G AAA | 55 | P | L | -8.51 | Deleterious | [rs11852361](http://www.ncbi.nlm.nih.gov/projects/SNP/snp_ref.cgi?rs=rs11852361) |
|  | TTA C[C/T]G AAA | 521 | P | L | -6.58 | Deleterious | [rs11852361](http://www.ncbi.nlm.nih.gov/projects/SNP/snp_ref.cgi?rs=rs11852361) |
|  | TTA C[C/T]G AAA | 868 | P | L | -6.88 | Deleterious | [rs11852361](http://www.ncbi.nlm.nih.gov/projects/SNP/snp_ref.cgi?rs=rs11852361) |
| 12,133235981,G,T | AAG [C/A]GC CTG | 1059 | R | S | -5.55 | Deleterious |  |
|  | AAG [C/A]GC CTG | 994 | R | S | -5.71 | Deleterious |  |
|  | AAG [C/A]GC CTG | 1070 | R | S | -5.55 | Deleterious |  |
|  | AAG [C/A]GC CTG | 36 | R | S | -5.63 | Deleterious |  |
|  | AAG [C/A]GC CTG | 839 | R | S | -5.81 | Deleterious |  |
|  | AAG [C/A]GC CTG | 1032 | R | S | -5.55 | Deleterious |  |
| 12,133244192,A,C | AAG G[T/G]G GAA | 739 | V | G | -3.87 | Deleterious |  |
|  | AAG G[T/G]G GAA | 674 | V | G | -4.19 | Deleterious |  |
|  | AAG G[T/G]G GAA | 750 | V | G | -3.87 | Deleterious |  |
|  | AAG G[T/G]G GAA | 519 | V | G | -4 | Deleterious |  |
|  | AAG G[T/G]G GAA | 712 | V | G | -3.87 | Deleterious |  |
| 19,44050225,T,C | GGG G[A/G]C ACA | 487 | D | G | -4.16 | Deleterious |  |
|  | GGG G[A/G]C ACA | 501 | D | G | -4.16 | Deleterious |  |
|  | GGG G[A/G]C ACA | 456 | D | G | -4.17 | Deleterious |  |
| 12,133220526,T,C | TAC A[A/G]T CTC | 1396 | N | S | -3.34 | Deleterious | [rs5744934](http://www.ncbi.nlm.nih.gov/projects/SNP/snp_ref.cgi?rs=rs5744934) |
|  | TAC A[A/G]T CTC | 1407 | N | S | -3.34 | Deleterious | [rs5744934](http://www.ncbi.nlm.nih.gov/projects/SNP/snp_ref.cgi?rs=rs5744934) |
|  | TAC A[A/G]T CTC | 1369 | N | S | -3.34 | Deleterious | [rs5744934](http://www.ncbi.nlm.nih.gov/projects/SNP/snp_ref.cgi?rs=rs5744934) |
| 6,30878709,T,A | GGG C[T/A]C ATG | 184 | L | H | -6.73 | Deleterious |  |
|  | GGG C[T/A]C ATG | 184 | L | H | -6.73 | Deleterious |  |
|  | GGG C[T/A]C ATG | 128 | L | H | -6.84 | Deleterious |  |
| 17,41244435,T,C | AAA G[A/G]A GCC | 1038 | E | G | -5.59 | Deleterious | [rs16941](http://www.ncbi.nlm.nih.gov/projects/SNP/snp_ref.cgi?rs=rs16941) |
|  | AAA G[A/G]A GCC | 742 | E | G | -5.36 | Deleterious | [rs16941](http://www.ncbi.nlm.nih.gov/projects/SNP/snp_ref.cgi?rs=rs16941) |
|  | AAA G[A/G]A GCC | 1038 | E | G | -5.59 | Deleterious | [rs16941](http://www.ncbi.nlm.nih.gov/projects/SNP/snp_ref.cgi?rs=rs16941) |
|  | AAA G[A/G]A GCC | 1038 | E | G | -5.69 | Deleterious | [rs16941](http://www.ncbi.nlm.nih.gov/projects/SNP/snp_ref.cgi?rs=rs16941) |
|  | AAA G[A/G]A GCC | 1038 | E | G | -5.78 | Deleterious | [rs16941](http://www.ncbi.nlm.nih.gov/projects/SNP/snp_ref.cgi?rs=rs16941) |
|  | AAA G[A/G]A GCC | 991 | E | G | -5.65 | Deleterious | [rs16941](http://www.ncbi.nlm.nih.gov/projects/SNP/snp_ref.cgi?rs=rs16941) |
|  | AAA G[A/G]A GCC | 1038 | E | G | -5.78 | Deleterious | [rs16941](http://www.ncbi.nlm.nih.gov/projects/SNP/snp_ref.cgi?rs=rs16941) |
| 7,92300822,C,T | CCC [G/A]AA GTC | 189 | E | K | -3.9 | Deleterious |  |
|  | CCC [G/A]AA GTC | 189 | E | K | -3.9 | Deleterious |  |
| 20,10621566,G,A | ATA [C/T]GG GAT | 1022 | R | W | -3.69 | Deleterious |  |
|  | ATA [C/T]GG GAT | 863 | R | W | -3.46 | Deleterious |  |
| 10,103340056,G,A | GGC [C/T]GG TCC | 438 | R | W | -4 | Deleterious | [rs3730477](http://www.ncbi.nlm.nih.gov/projects/SNP/snp_ref.cgi?rs=rs3730477) |
|  | GGC [C/T]GG TCC | 161 | R | W | -4.6 | Deleterious | [rs3730477](http://www.ncbi.nlm.nih.gov/projects/SNP/snp_ref.cgi?rs=rs3730477) |
|  | GGC [C/T]GG TCC | 438 | R | W | -4.1 | Deleterious | [rs3730477](http://www.ncbi.nlm.nih.gov/projects/SNP/snp_ref.cgi?rs=rs3730477) |
|  | GGC [C/T]GG TCC | 163 | R | W | -4.6 | Deleterious | [rs3730477](http://www.ncbi.nlm.nih.gov/projects/SNP/snp_ref.cgi?rs=rs3730477) |
|  | GGC [C/T]GG TCC | 438 | R | W | -4 | Deleterious | [rs3730477](http://www.ncbi.nlm.nih.gov/projects/SNP/snp_ref.cgi?rs=rs3730477) |
|  | GGC [C/T]GG TCC | 111 | R | W | -4.84 | Deleterious | [rs3730477](http://www.ncbi.nlm.nih.gov/projects/SNP/snp_ref.cgi?rs=rs3730477) |
|  | GGC [C/T]GG TCC | 438 | R | W | -4 | Deleterious | [rs3730477](http://www.ncbi.nlm.nih.gov/projects/SNP/snp_ref.cgi?rs=rs3730477) |
|  | GGC [C/T]GG TCC | 350 | R | W | -4.44 | Deleterious | [rs3730477](http://www.ncbi.nlm.nih.gov/projects/SNP/snp_ref.cgi?rs=rs3730477) |
|  | GGC [C/T]GG TCC | 438 | R | W | -4.1 | Deleterious | [rs3730477](http://www.ncbi.nlm.nih.gov/projects/SNP/snp_ref.cgi?rs=rs3730477) |
|  | GGC [C/T]GG TCC | 175 | R | W | -4.6 | Deleterious | [rs3730477](http://www.ncbi.nlm.nih.gov/projects/SNP/snp_ref.cgi?rs=rs3730477) |
|  | GGC [C/T]GG TCC | 346 | R | W | -4.34 | Deleterious | [rs3730477](http://www.ncbi.nlm.nih.gov/projects/SNP/snp_ref.cgi?rs=rs3730477) |
|  | GGC [C/T]GG TCC | 161 | R | W | -4.75 | Deleterious | [rs3730477](http://www.ncbi.nlm.nih.gov/projects/SNP/snp_ref.cgi?rs=rs3730477) |
| 3,129151414,C,T | ATT [G/A]AG CTT | 533 | E | K | -3.3 | Deleterious |  |
|  | ATT [G/A]AG CTT | 215 | E | K | -3.71 | Deleterious |  |
|  | ATT [G/A]AG CTT | 527 | E | K | -3.3 | Deleterious |  |
|  | ATT [G/A]AG CTT | 533 | E | K | -3.3 | Deleterious |  |
|  | ATT [G/A]AG CTT | 533 | E | K | -3.36 | Deleterious |  |
| 10,49628258,G,A | TTC [G/A]GT CTG | 171 | G | S | -5.71 | Deleterious |  |
|  | TTC [G/A]GT CTG | 171 | G | S | -5.92 | Deleterious |  |
|  | TTC [G/A]GT CTG | 171 | G | S | -5.68 | Deleterious |  |
|  | TTC [G/A]GT CTG | 171 | G | S | -5.68 | Deleterious |  |
|  | TTC [G/A]GT CTG | 171 | G | S | -5.71 | Deleterious |  |
|  | TTC [G/A]GT CTG | 171 | G | S | -5.71 | Deleterious |  |
|  | TTC [G/A]GT CTG | 171 | G | S | -5.24 | Deleterious |  |
|  | TTC [G/A]GT CTG | 88 | G | S | -5.92 | Deleterious |  |
| 11,61071386,C,T | ATG C[G/A]C TCA | 928 | R | H | -4.3 | Deleterious |  |
|  | ATG C[G/A]C TCA | 239 | R | H | -4.34 | Deleterious |  |
|  | ATG C[G/A]C TCA | 94 | R | H | -4.37 | Deleterious |  |
|  | ATG C[G/A]C TCA | 928 | R | H | -4.3 | Deleterious |  |
| 11,108165687,G,T | TAT [G/T]AT GCA | 1604 | D | Y | -3.58 | Deleterious |  |
|  | TAT [G/T]AT GCA | 1604 | D | Y | -3.58 | Deleterious |  |
| 12,124144393,C,T | CCC [C/T]CA GTT | 205 | P | S | -2.54 | Deleterious |  |
|  | CCC [C/T]CA GTT | 132 | P | S | -3.05 | Deleterious |  |
|  | CCC [C/T]CA GTT | 203 | P | S | -3.27 | Deleterious |  |
| 8,42207528,C,T | ATT [C/T]GG CAG | 89 | R | W | -5.15 | Deleterious |  |
|  | ATT [C/T]GG CAG | 89 | R | W | -5.3 | Deleterious |  |
|  | ATT [C/T]GG CAG | 20 | R | W | -5.19 | Deleterious |  |
|  | ATT [C/T]GG CAG | 89 | R | W | -5.11 | Deleterious |  |
| 4,122740059,C,G | GGA [G/C]AA TTA | 338 | E | Q | -2.69 | Deleterious |  |
| 19,2477565,A,T | AAC C[A/T]G TGG | 150 | Q | L | -3.8 | Deleterious |  |
| 11,94219166,T,C | TTA [A/G]GA AAA | 80 | R | G | -6.63 | Deleterious |  |
|  | TTA [A/G]GA AAA | 80 | R | G | -6.63 | Deleterious |  |
|  | TTA [A/G]GA AAA | 80 | R | G | -6.63 | Deleterious |  |
|  | TTA [A/G]GA AAA | 83 | R | G | -6.63 | Deleterious |  |
|  | TTA [A/G]GA AAA | 80 | R | G | -6.87 | Deleterious |  |
|  | TTA [A/G]GA AAA | 80 | R | G | -6.86 | Deleterious |  |
|  | TTA [A/G]GA AAA | 80 | R | G | -6.87 | Deleterious |  |
| 2,48028241,T,G | AAC T[T/G]T GAT | 1040 | F | C | -7.71 | Deleterious |  |
|  | AAC T[T/G]T GAT | 738 | F | C | -7.58 | Deleterious |  |
|  | AAC T[T/G]T GAT | 1038 | F | C | -7.31 | Deleterious |  |
|  | AAC T[T/G]T GAT | 8 | F | C | -7.53 | Deleterious |  |
|  | AAC T[T/G]T GAT | 910 | F | C | -7.71 | Deleterious |  |
| 11,61093123,T,A | CAC A[A/T]T GGT | 241 | N | I | -5.05 | Deleterious |  |
|  | CAC A[A/T]T GGT | 241 | N | I | -5.05 | Deleterious |  |
|  | CAC A[A/T]T GGT | 56 | N | I | -5.33 | Deleterious |  |
|  | CAC A[A/T]T GGT | 24 | N | I | -5 | Deleterious |  |
| 8,22548374,C,T | CCC C[G/A]C AAG | 259 | R | H | -4.49 | Deleterious |  |
|  | CCC C[G/A]C AAG | 100 | R | H | -4.8 | Deleterious |  |
|  | CCC C[G/A]C AAG | 221 | R | H | -4.46 | Deleterious |  |
| 7,44156477,C,A | TCC C[G/T]G GTT | 240 | R | L | -6.11 | Deleterious |  |
|  | TCC C[G/T]G GTT | 240 | R | L | -6.11 | Deleterious |  |
|  | TCC C[G/T]G GTT | 240 | R | L | -6.11 | Deleterious |  |
|  | TCC C[G/T]G GTT | 158 | R | L | -6.05 | Deleterious |  |
|  | GGC [G/A]TG CAC | 639 | V | M | -2.53 | Deleterious | [rs115319252](http://www.ncbi.nlm.nih.gov/projects/SNP/snp_ref.cgi?rs=rs115319252) |
|  | GGC [G/A]TG CAC | 632 | V | M | -2.53 | Deleterious | [rs115319252](http://www.ncbi.nlm.nih.gov/projects/SNP/snp_ref.cgi?rs=rs115319252) |
| 2,73518703,G,A | TTC G[C/T]G CGC | 448 | A | V | -3.92 | Deleterious |  |
|  | TTC G[C/T]G CGC | 551 | A | V | -3.92 | Deleterious |  |
| 20,10626016,T,G | AAG [A/C]CC TGC | 701 | T | P | -5.66 | Deleterious | [rs79176844](http://www.ncbi.nlm.nih.gov/projects/SNP/snp_ref.cgi?rs=rs79176844) |
|  | AAG [A/C]CC TGC | 542 | T | P | -5.66 | Deleterious | [rs79176844](http://www.ncbi.nlm.nih.gov/projects/SNP/snp_ref.cgi?rs=rs79176844) |
| 2,217059669,A,C | GAG G[A/C]A GCC | 690 | E | A | -4.53 | Deleterious |  |
|  | GAG G[A/C]A GCC | 690 | E | A | -4.53 | Deleterious |  |
| 22,29091788,T,C | ACC T[A/G]C TTG | 361 | Y | C | -8.38 | Deleterious |  |
|  | ACC T[A/G]C TTG | 390 | Y | C | -8.66 | Deleterious |  |
|  | ACC T[A/G]C TTG | 299 | Y | C | -8.06 | Deleterious |  |
|  | ACC T[A/G]C TTG | 433 | Y | C | -8.71 | Deleterious |  |
|  | ACC T[A/G]C TTG | 361 | Y | C | -8.38 | Deleterious |  |
|  | ACC T[A/G]C TTG | 299 | Y | C | -8.06 | Deleterious |  |
|  | ACC T[A/G]C TTG | 390 | Y | C | -8.66 | Deleterious |  |
|  | ACC T[A/G]C TTG | 390 | Y | C | -8.66 | Deleterious |  |
|  | ACC T[A/G]C TTG | 134 | Y | C | -8.5 | Deleterious |  |
|  | ACC T[A/G]C TTG | 169 | Y | C | -8.1 | Deleterious |  |
| X,66905920,C,A | TGT [C/A]CA TCT | 613 | P | T | -6.08 | Deleterious |  |
|  | TGT [C/A]CA TCT | 81 | P | T | -6.8 | Deleterious |  |
|  | TGT [C/A]CA TCT | 613 | P | T | -4.99 | Deleterious |  |
|  | TGT [C/A]CA TCT | 613 | P | T | -5.33 | Deleterious |  |
|  | TGT [C/A]CA TCT | 423 | P | T | -6.26 | Deleterious |  |
|  | GAG [C/A]CT GGA | 135 | P | T | -2.83 | Deleterious |  |
|  | GAG [C/A]CT GGA | 135 | P | T | -2.84 | Deleterious |  |
|  | GAG [C/A]CT GGA | 127 | P | T | -3.34 | Deleterious |  |
| 13,32914091,A,T | TTT [A/T]CA GAC | 1867 | T | S | -2.59 | Deleterious |  |
|  | TTT [A/T]CA GAC | 1867 | T | S | -2.59 | Deleterious |  |
| 3,48207368,T,G | CAT [A/C]CA GTT | 349 | T | P | -5.88 | Deleterious |  |
|  | CAT [A/C]CA GTT | 309 | T | P | -5.79 | Deleterious |  |
| 3,142180920,C,T | AAA [G/A]AT GCA | 2352 | D | N | -3.14 | Deleterious |  |
|  | AAA [G/A]AT GCA | 2288 | D | N | -3.14 | Deleterious |  |
|  | AAA [G/A]AT GCA | 199 | D | N | -3.69 | Deleterious |  |
| 11,18359739,C,A | TGG G[C/A]C AAT | 144 | A | D | -2.92 | Deleterious |  |
|  | TGG G[C/A]C AAT | 144 | A | D | -2.92 | Deleterious |  |
|  | TGG G[C/A]C AAT | 28 | A | D | -3.09 | Deleterious |  |
| 2,190660571,C,A | ATG G[C/A]A ATG | 70 | A | E | -4.39 | Deleterious |  |
|  | ATG G[C/A]A ATG | 70 | A | E | -4.4 | Deleterious |  |
|  | ATG G[C/A]A ATG | 70 | A | E | -4.4 | Deleterious |  |
|  | ATG G[C/A]A ATG | 70 | A | E | -4.25 | Deleterious |  |
|  | ATG G[C/A]A ATG | 70 | A | E | -3.93 | Deleterious |  |
|  | ATG G[C/A]A ATG | 70 | A | E | -4.18 | Deleterious |  |
|  | ATG G[C/A]A ATG | 70 | A | E | -4.15 | Deleterious |  |
|  | ATG G[C/A]A ATG | 70 | A | E | -4.18 | Deleterious |  |
|  | ATG G[C/A]A ATG | 70 | A | E | -4.39 | Deleterious |  |
| 12,4398064,G,A | GCA [G/A]CC ATC | 210 | A | T | -3.47 | Deleterious |  |
| 13,108863591,G,A | CAA A[C/T]T GTT | 9 | T | I | -2.7 | Deleterious | [rs1805388](http://www.ncbi.nlm.nih.gov/projects/SNP/snp_ref.cgi?rs=rs1805388) |
|  | CAA A[C/T]T GTT | 9 | T | I | -2.7 | Deleterious | [rs1805388](http://www.ncbi.nlm.nih.gov/projects/SNP/snp_ref.cgi?rs=rs1805388) |
|  | CAA A[C/T]T GTT | 9 | T | I | -2.7 | Deleterious | [rs1805388](http://www.ncbi.nlm.nih.gov/projects/SNP/snp_ref.cgi?rs=rs1805388) |
|  | TTT C[G/A]T AAT | 337 | R | H | -3.16 | Deleterious |  |
| 3,9798462,C,T | TTC [C/T]GG AGC | 304 | R | W | -6.62 | Deleterious |  |
|  | TTC [C/T]GG AGC | 304 | R | W | -6.62 | Deleterious |  |
|  | TTC [C/T]GG AGC | 304 | R | W | -6.75 | Deleterious |  |
|  | TTC [C/T]GG AGC | 304 | R | W | -6.82 | Deleterious |  |
|  | TTC [C/T]GG AGC | 304 | R | W | -6.81 | Deleterious |  |
|  | TTC [C/T]GG AGC | 202 | R | W | -6.96 | Deleterious |  |
|  | TTC [C/T]GG AGC | 71 | R | W | -7.35 | Deleterious |  |
|  | TTC [C/T]GG AGC | 304 | R | W | -6.62 | Deleterious |  |
| 2,128015230,G,A | TCA T[C/T]G CGG | 764 | S | L | -2.88 | Deleterious | [rs189511674](http://www.ncbi.nlm.nih.gov/projects/SNP/snp_ref.cgi?rs=rs189511674) |
|  | TCA T[C/T]G CGG | 700 | S | L | -2.78 | Deleterious | [rs189511674](http://www.ncbi.nlm.nih.gov/projects/SNP/snp_ref.cgi?rs=rs189511674) |
|  | ATC T[C/T]C AAG | 382 | S | F | -3.1 | Deleterious | [rs41293475](http://www.ncbi.nlm.nih.gov/projects/SNP/snp_ref.cgi?rs=rs41293475) |
| 19,44057574,G,A | AGC [C/T]GG ATC | 194 | R | W | -4.42 | Deleterious | [rs1799782](http://www.ncbi.nlm.nih.gov/projects/SNP/snp_ref.cgi?rs=rs1799782) |
|  | AGC [C/T]GG ATC | 208 | R | W | -4.42 | Deleterious | [rs1799782](http://www.ncbi.nlm.nih.gov/projects/SNP/snp_ref.cgi?rs=rs1799782) |
|  | AGC [C/T]GG ATC | 163 | R | W | -3.68 | Deleterious | [rs1799782](http://www.ncbi.nlm.nih.gov/projects/SNP/snp_ref.cgi?rs=rs1799782) |
|  | AGC [C/T]GG ATC | 194 | R | W | -3.9 | Deleterious | [rs1799782](http://www.ncbi.nlm.nih.gov/projects/SNP/snp_ref.cgi?rs=rs1799782) |
|  | CAA [G/A]AT ACA | 24 | D | N | -3.18 | Deleterious | [rs1801516](http://www.ncbi.nlm.nih.gov/projects/SNP/snp_ref.cgi?rs=rs1801516) |
| 11,108173677,T,C | TGG A[T/C]A AAG | 1806 | I | T | -3.99 | Deleterious |  |
|  | TGG A[T/C]A AAG | 1806 | I | T | -3.99 | Deleterious |  |
| 17,41244435,T,C | AAA G[A/G]A GCC | 1038 | E | G | -5.59 | Deleterious | [rs16941](http://www.ncbi.nlm.nih.gov/projects/SNP/snp_ref.cgi?rs=rs16941) |
|  | AAA G[A/G]A GCC | 742 | E | G | -5.36 | Deleterious | [rs16941](http://www.ncbi.nlm.nih.gov/projects/SNP/snp_ref.cgi?rs=rs16941) |
|  | AAA G[A/G]A GCC | 1038 | E | G | -5.59 | Deleterious | [rs16941](http://www.ncbi.nlm.nih.gov/projects/SNP/snp_ref.cgi?rs=rs16941) |
|  | AAA G[A/G]A GCC | 1038 | E | G | -5.69 | Deleterious | [rs16941](http://www.ncbi.nlm.nih.gov/projects/SNP/snp_ref.cgi?rs=rs16941) |
|  | AAA G[A/G]A GCC | 1038 | E | G | -5.78 | Deleterious | [rs16941](http://www.ncbi.nlm.nih.gov/projects/SNP/snp_ref.cgi?rs=rs16941) |
|  | AAA G[A/G]A GCC | 991 | E | G | -5.65 | Deleterious | [rs16941](http://www.ncbi.nlm.nih.gov/projects/SNP/snp_ref.cgi?rs=rs16941) |
|  | AAA G[A/G]A GCC | 1038 | E | G | -5.78 | Deleterious | [rs16941](http://www.ncbi.nlm.nih.gov/projects/SNP/snp_ref.cgi?rs=rs16941) |
| 10,50678369,T,C | TGC [A/G]GA GAC | 1213 | R | G | -3.21 | Deleterious | [rs2228527](http://www.ncbi.nlm.nih.gov/projects/SNP/snp_ref.cgi?rs=rs2228527) |
|  | TGC [A/G]GA GAC | 590 | R | G | -3.31 | Deleterious | [rs2228527](http://www.ncbi.nlm.nih.gov/projects/SNP/snp_ref.cgi?rs=rs2228527) |
|  | TGC [A/G]GA GAC | 583 | R | G | -3.21 | Deleterious | [rs2228527](http://www.ncbi.nlm.nih.gov/projects/SNP/snp_ref.cgi?rs=rs2228527) |
| 20,10622501,G,C | ATA C[C/G]A GAT | 871 | P | R | -5.74 | Deleterious | [rs35761929](http://www.ncbi.nlm.nih.gov/projects/SNP/snp_ref.cgi?rs=rs35761929) |
|  | ATA C[C/G]A GAT | 712 | P | R | -5.44 | Deleterious | [rs35761929](http://www.ncbi.nlm.nih.gov/projects/SNP/snp_ref.cgi?rs=rs35761929) |
| 17,41244435,T,C | AAA G[A/G]A GCC | 1038 | E | G | -5.59 | Deleterious | [rs16941](http://www.ncbi.nlm.nih.gov/projects/SNP/snp_ref.cgi?rs=rs16941) |
|  | AAA G[A/G]A GCC | 742 | E | G | -5.36 | Deleterious | [rs16941](http://www.ncbi.nlm.nih.gov/projects/SNP/snp_ref.cgi?rs=rs16941) |
|  | AAA G[A/G]A GCC | 1038 | E | G | -5.59 | Deleterious | [rs16941](http://www.ncbi.nlm.nih.gov/projects/SNP/snp_ref.cgi?rs=rs16941) |
|  | AAA G[A/G]A GCC | 1038 | E | G | -5.69 | Deleterious | [rs16941](http://www.ncbi.nlm.nih.gov/projects/SNP/snp_ref.cgi?rs=rs16941) |
|  | AAA G[A/G]A GCC | 1038 | E | G | -5.78 | Deleterious | [rs16941](http://www.ncbi.nlm.nih.gov/projects/SNP/snp_ref.cgi?rs=rs16941) |
|  | AAA G[A/G]A GCC | 991 | E | G | -5.65 | Deleterious | [rs16941](http://www.ncbi.nlm.nih.gov/projects/SNP/snp_ref.cgi?rs=rs16941) |
|  | AAA G[A/G]A GCC | 1038 | E | G | -5.78 | Deleterious | [rs16941](http://www.ncbi.nlm.nih.gov/projects/SNP/snp_ref.cgi?rs=rs16941) |
| 5,86695274,A,G | GAA G[T/C]T GCT | 270 | V | A | -2.76 | Deleterious | [rs2230641](http://www.ncbi.nlm.nih.gov/projects/SNP/snp_ref.cgi?rs=rs2230641) |
|  | GAA G[T/C]T GCT | 196 | V | A | -2.82 | Deleterious | [rs2230641](http://www.ncbi.nlm.nih.gov/projects/SNP/snp_ref.cgi?rs=rs2230641) |
|  | GAA G[T/C]T GCT | 196 | V | A | -2.92 | Deleterious | [rs2230641](http://www.ncbi.nlm.nih.gov/projects/SNP/snp_ref.cgi?rs=rs2230641) |
| 13,108863591,G,A | CAA A[C/T]T GTT | 9 | T | I | -2.7 | Deleterious | [rs1805388](http://www.ncbi.nlm.nih.gov/projects/SNP/snp_ref.cgi?rs=rs1805388) |
|  | CAA A[C/T]T GTT | 9 | T | I | -2.7 | Deleterious | [rs1805388](http://www.ncbi.nlm.nih.gov/projects/SNP/snp_ref.cgi?rs=rs1805388) |
|  | CAA A[C/T]T GTT | 9 | T | I | -2.7 | Deleterious | [rs1805388](http://www.ncbi.nlm.nih.gov/projects/SNP/snp_ref.cgi?rs=rs1805388) |
| 4,178256913,C,G | TCT C[C/G]T GTT | 117 | P | R | -5.64 | Deleterious | [rs7689099](http://www.ncbi.nlm.nih.gov/projects/SNP/snp_ref.cgi?rs=rs7689099) |
|  | TGG [G/A]CG TGG | 14 | A | T | -3.63 | Deleterious | [rs34689457](http://www.ncbi.nlm.nih.gov/projects/SNP/snp_ref.cgi?rs=rs34689457) |
| 2,190719499,G,A | AGG [G/A]GA AAT | 325 | G | R | -5.48 | Deleterious | [rs1145232](http://www.ncbi.nlm.nih.gov/projects/SNP/snp_ref.cgi?rs=rs1145232) |
|  | AGG [G/A]GA AAT | 462 | G | R | -5.5 | Deleterious | [rs1145232](http://www.ncbi.nlm.nih.gov/projects/SNP/snp_ref.cgi?rs=rs1145232) |
|  | AGG [G/A]GA AAT | 286 | G | R | -3.96 | Deleterious | [rs1145232](http://www.ncbi.nlm.nih.gov/projects/SNP/snp_ref.cgi?rs=rs1145232) |
|  | AGG [G/A]GA AAT | 440 | G | R | -5.62 | Deleterious | [rs1145232](http://www.ncbi.nlm.nih.gov/projects/SNP/snp_ref.cgi?rs=rs1145232) |
|  | AGG [G/A]GA AAT | 325 | G | R | -5.21 | Deleterious | [rs1145232](http://www.ncbi.nlm.nih.gov/projects/SNP/snp_ref.cgi?rs=rs1145232) |
|  | AGG [G/A]GA AAT | 501 | G | R | -5.49 | Deleterious | [rs1145232](http://www.ncbi.nlm.nih.gov/projects/SNP/snp_ref.cgi?rs=rs1145232) |
|  | AGG [G/A]GA AAT | 325 | G | R | -5.21 | Deleterious | [rs1145232](http://www.ncbi.nlm.nih.gov/projects/SNP/snp_ref.cgi?rs=rs1145232) |
|  | AGG [G/A]GA AAT | 462 | G | R | -5.52 | Deleterious | [rs1145232](http://www.ncbi.nlm.nih.gov/projects/SNP/snp_ref.cgi?rs=rs1145232) |
|  | AGG [G/A]GA AAT | 501 | G | R | -5.83 | Deleterious | [rs1145232](http://www.ncbi.nlm.nih.gov/projects/SNP/snp_ref.cgi?rs=rs1145232) |
| 1,242048674,C,T | AAA C[C/T]T CTA | 757 | P | L | -4.15 | Deleterious | [rs9350](http://www.ncbi.nlm.nih.gov/projects/SNP/snp_ref.cgi?rs=rs9350) |
|  | AAA C[C/T]T CTA | 757 | P | L | -4.15 | Deleterious | [rs9350](http://www.ncbi.nlm.nih.gov/projects/SNP/snp_ref.cgi?rs=rs9350) |
|  | AAA C[C/T]T CTA | 122 | P | L | -7.83 | Deleterious | [rs9350](http://www.ncbi.nlm.nih.gov/projects/SNP/snp_ref.cgi?rs=rs9350) |
|  | AAA C[C/T]T CTA | 757 | P | L | -4.05 | Deleterious | [rs9350](http://www.ncbi.nlm.nih.gov/projects/SNP/snp_ref.cgi?rs=rs9350) |
| 4,178256852,G,A | TTC [G/A]GA ATG | 97 | G | R | -3.44 | Deleterious |  |
| 3,121228960,G,A | GGA G[C/T]G ATT | 581 | A | V | -3.1 | Deleterious | [rs487848](http://www.ncbi.nlm.nih.gov/projects/SNP/snp_ref.cgi?rs=rs487848) |
|  | GGA G[C/T]G ATT | 717 | A | V | -3.1 | Deleterious | [rs487848](http://www.ncbi.nlm.nih.gov/projects/SNP/snp_ref.cgi?rs=rs487848) |
|  | GGA G[C/T]G ATT | 204 | A | V | -2.8 | Deleterious | [rs487848](http://www.ncbi.nlm.nih.gov/projects/SNP/snp_ref.cgi?rs=rs487848) |
| 3,121190839,G,T | GCT A[C/A]A GGA | 2239 | T | K | -5.08 | Deleterious |  |
|  | GCT A[C/A]A GGA | 2375 | T | K | -5.08 | Deleterious |  |
|  | GCT A[C/A]A GGA | 1862 | T | K | -5.08 | Deleterious |  |
| 3,121228415,A,T | ATT C[T/A]C TAT | 651 | L | H | -5.26 | Deleterious |  |
|  | ATT C[T/A]C TAT | 787 | L | H | -5.26 | Deleterious |  |
|  | ATT C[T/A]C TAT | 274 | L | H | -5.26 | Deleterious |  |
| 5,131940521,C,T | AAT [C/T]GT AAG | 850 | R | C | -2.89 | Deleterious | [rs181961360](http://www.ncbi.nlm.nih.gov/projects/SNP/snp_ref.cgi?rs=rs181961360) |
|  | AAT [C/T]GT AAG | 711 | R | C | -3.09 | Deleterious | [rs181961360](http://www.ncbi.nlm.nih.gov/projects/SNP/snp_ref.cgi?rs=rs181961360) |
| 21,42866487,C,A | ATT [G/T]GA CCT | 12 | G | * | NA | NA |  |
|  | ATT [G/T]GA CCT | 49 | G | * | NA | NA |  |
|  | ATT [G/T]GA CCT | 12 | G | * | NA | NA |  |
|  | ATT [G/T]GA CCT | 12 | G | * | NA | NA |  |
|  | ATT [G/T]GA CCT | 12 | G | * | NA | NA |  |
|  | ATT [G/T]GA CCT | 12 | G | * | NA | NA |  |
| 3,121200503,G,A | GGG [C/T]GA TAC | 2043 | R | * | NA | NA |  |
|  | GGG [C/T]GA TAC | 2179 | R | * | NA | NA |  |
|  | GGG [C/T]GA TAC | 1666 | R | * | NA | NA |  |
| 22,42032159,C,T | GAT [C/T]GA GAT | 80 | R | * | NA | NA |  |
|  | GAT [C/T]GA GAT | 80 | R | * | NA | NA |  |
|  | GAT [C/T]GA GAT | 30 | R | * | NA | NA |  |
|  | GAT [C/T]GA GAT | 80 | R | * | NA | NA |  |
|  | GAT [C/T]GA GAT | 80 | R | * | NA | NA |  |
| 17,41256159,C,A | AGT [G/T]AA CCC | 141 | E | * | NA | NA |  |
|  | AGT [G/T]AA CCC | 141 | E | * | NA | NA |  |
|  | AGT [G/T]AA CCC | 141 | E | * | NA | NA |  |
|  | AGT [G/T]AA CCC | 141 | E | * | NA | NA |  |
|  | AGT [G/T]AA CCC | 141 | E | * | NA | NA |  |
|  | AGT [G/T]AA CCC | 94 | E | * | NA | NA |  |
|  | AGT [G/T]AA CCC | 141 | E | * | NA | NA |  |
|  | AGT [G/T]AA CCC | 141 | E | * | NA | NA |  |
|  | AGT [G/T]AA CCC | 141 | E | * | NA | NA |  |
|  | AGT [G/T]AA CCC | 57 | E | * | NA | NA |  |
|  | AGT [G/T]AA CCC | 94 | E | * | NA | NA |  |
|  | AGT [G/T]AA CCC | 94 | E | * | NA | NA |  |
|  | AGT [G/T]AA CCC | 141 | E | * | NA | NA |  |
|  | AGT [G/T]AA CCC | 141 | E | * | NA | NA |  |
|  | AGT [G/T]AA CCC | 141 | E | * | NA | NA |  |
|  | AGT [G/T]AA CCC | 57 | E | * | NA | NA |  |
|  | AGT [G/T]AA CCC | 115 | E | * | NA | NA |  |
|  | AGT [G/T]AA CCC | 49 | E | * | NA | NA |  |
|  | AGT [G/T]AA CCC | 141 | E | * | NA | NA |  |
|  | AGT [G/T]AA CCC | 141 | E | * | NA | NA |  |
| 9,100447247,G,A | AAC [C/T]GA GAA | 211 | R | * | NA | NA | [rs149226993](http://www.ncbi.nlm.nih.gov/projects/SNP/snp_ref.cgi?rs=rs149226993) |
|  | AAC [C/T]GA GAA | 211 | R | * | NA | NA | [rs149226993](http://www.ncbi.nlm.nih.gov/projects/SNP/snp_ref.cgi?rs=rs149226993) |
| 11,94204756,C,A | CCA [G/T]GA GAA | 277 | G | * | NA | NA |  |
|  | CCA [G/T]GA GAA | 277 | G | * | NA | NA |  |
|  | CCA [G/T]GA GAA | 277 | G | * | NA | NA |  |
|  | CCA [G/T]GA GAA | 280 | G | * | NA | NA |  |
| 18,20581641,C,T | TCC [C/T]AA GCA | 746 | Q | * | NA | NA |  |
|  | TCC [C/T]AA GCA | 751 | Q | * | NA | NA |  |
|  | TCC [C/T]AA GCA | 746 | Q | * | NA | NA |  |
|  | TCC [C/T]AA GCA | 746 | Q | * | NA | NA |  |
|  | TCC [C/T]AA GCA | 746 | Q | * | NA | NA |  |
| 3,58519862,C,A | TCT [G/T]GA GAC | 112 | G | * | NA | NA |  |
|  | TCT [G/T]GA GAC | 112 | G | * | NA | NA |  |
|  | TCT [G/T]GA GAC | 112 | G | * | NA | NA |  |
| 17,7579906,C,A | GAG [G/T]AG CCG | 3 | E | * | NA | NA |  |
|  | GAG [G/T]AG CCG | 3 | E | * | NA | NA |  |
|  | GAG [G/T]AG CCG | 3 | E | * | NA | NA |  |
|  | GAG [G/T]AG CCG | 3 | E | * | NA | NA |  |
|  | GAG [G/T]AG CCG | 3 | E | * | NA | NA |  |
|  | GAG [G/T]AG CCG | 3 | E | * | NA | NA |  |
|  | GAG [G/T]AG CCG | 3 | E | * | NA | NA |  |
|  | GAG [G/T]AG CCG | 3 | E | * | NA | NA |  |
|  | GAG [G/T]AG CCG | 3 | E | * | NA | NA |  |
|  | GAG [G/T]AG CCG | 3 | E | * | NA | NA |  |
|  | GAG [G/T]AG CCG | 3 | E | * | NA | NA |  |
| 6,41905126,C,A | TGG [G/T]AG GTG | 91 | E | * | NA | NA |  |
|  | TGG [G/T]AG GTG | 60 | E | * | NA | NA |  |
|  | TGG [G/T]AG GTG | 141 | E | * | NA | NA |  |
|  | TGG [G/T]AG GTG | 69 | E | * | NA | NA |  |
|  | TGG [G/T]AG GTG | 76 | E | * | NA | NA |  |
|  | TGG [G/T]AG GTG | 60 | E | * | NA | NA |  |
|  | TGG [G/T]AG GTG | 60 | E | * | NA | NA |  |
|  | TGG [G/T]AG GTG | 60 | E | * | NA | NA |  |
| 18,20516825,C,A | ATC T[C/A]G GGA | 4 | S | * | NA | NA |  |
|  | ATC T[C/A]G GGA | 4 | S | * | NA | NA |  |
|  | ATC T[C/A]G GGA | 4 | S | * | NA | NA |  |
|  | ATC T[C/A]G GGA | 4 | S | * | NA | NA |  |
|  | ATC T[C/A]G GGA | 4 | S | * | NA | NA |  |
| 7,44113600,G,A | CAC [C/T]AG CAC | 366 | Q | * | NA | NA |  |
|  | CAC [C/T]AG CAC | 329 | Q | * | NA | NA |  |
|  | CAC [C/T]AG CAC | 286 | Q | * | NA | NA |  |
| 22,42032159,C,T | GAT [C/T]GA GAT | 80 | R | * | NA | NA |  |
|  | GAT [C/T]GA GAT | 80 | R | * | NA | NA |  |
|  | GAT [C/T]GA GAT | 30 | R | * | NA | NA |  |
|  | GAT [C/T]GA GAT | 80 | R | * | NA | NA |  |
|  | GAT [C/T]GA GAT | 80 | R | * | NA | NA |  |
|  | CGG [C/T]AA AAT | 36 | Q | * | NA | NA | [rs140696](http://www.ncbi.nlm.nih.gov/projects/SNP/snp_ref.cgi?rs=rs140696) |
| 2,47690264,C,A | CAG T[C/A]A ACA | 494 | S | * | NA | NA |  |
|  | CAG T[C/A]A ACA | 494 | S | * | NA | NA |  |
|  | CAG T[C/A]A ACA | 144 | S | * | NA | NA |  |
|  | CAG T[C/A]A ACA | 494 | S | * | NA | NA |  |
|  | CAG T[C/A]A ACA | 494 | S | * | NA | NA |  |
|  | CAG T[C/A]A ACA | 280 | S | * | NA | NA |  |
|  | CAG T[C/A]A ACA | 428 | S | * | NA | NA |  |
| 8,42220154,G,A | GTG [G/A]AG CAG | 216 | E | K | -1.25 | Neutral |  |
|  | GTG [G/A]AG CAG | 32 | E | K | -1.31 | Neutral |  |
|  | GTG [G/A]AG CAG | 74 | E | K | -1.16 | Neutral |  |
|  | GTG [G/A]AG CAG | 251 | E | K | -1.11 | Neutral |  |
|  | GTG [G/A]AG CAG | 62 | E | K | -1.16 | Neutral |  |
| 13,32929232,A,G | AAA TC[A/G] CAT | 2414 | S | S | 0 | Neutral | [rs1799955](http://www.ncbi.nlm.nih.gov/projects/SNP/snp_ref.cgi?rs=rs1799955) |
|  | AAA TC[A/G] CAT | 2414 | S | S | 0 | Neutral | [rs1799955](http://www.ncbi.nlm.nih.gov/projects/SNP/snp_ref.cgi?rs=rs1799955) |
| 14,38061715,C,T | GGC [G/A]CC ATG | 92 | A | T | -1.2 | Neutral |  |
|  | GGC [G/A]CC ATG | 59 | A | T | -1.2 | Neutral |  |
| 9,139407932,A,G | AAC AA[T/C] GAG | 755 | N | N | 0 | Neutral | [rs2229971](http://www.ncbi.nlm.nih.gov/projects/SNP/snp_ref.cgi?rs=rs2229971) |
| 9,139391636,G,A | CAG GA[C/T] GGC | 2185 | D | D | 0 | Neutral | [rs2229974](http://www.ncbi.nlm.nih.gov/projects/SNP/snp_ref.cgi?rs=rs2229974) |
| 3,48200873,G,T | TAT GT[C/A] CTG | 465 | V | V | 0 | Neutral |  |
|  | TAT GT[C/A] CTG | 425 | V | V | 0 | Neutral |  |
| 2,47639574,C,T | ATT [C/T]TG ATC | 223 | L | L | 0 | Neutral |  |
|  | ATT [C/T]TG ATC | 223 | L | L | 0 | Neutral |  |
|  | ATT [C/T]TG ATC | 223 | L | L | 0 | Neutral |  |
|  | ATT [C/T]TG ATC | 223 | L | L | 0 | Neutral |  |
|  | ATT [C/T]TG ATC | 223 | L | L | 0 | Neutral |  |
|  | ATT [C/T]TG ATC | 223 | L | L | 0 | Neutral |  |
|  | ATT [C/T]TG ATC | 59 | L | L | 0 | Neutral |  |
|  | ATT [C/T]TG ATC | 223 | L | L | 0 | Neutral |  |
|  | ATT [C/T]TG ATC | 157 | L | L | 0 | Neutral |  |
| 7,6026864,G,A | GAC A[C/T]G GGC | 511 | T | M | -0.87 | Neutral | [rs74902811](http://www.ncbi.nlm.nih.gov/projects/SNP/snp_ref.cgi?rs=rs74902811) |
|  | GAC A[C/T]G GGC | 464 | T | M | -0.87 | Neutral | [rs74902811](http://www.ncbi.nlm.nih.gov/projects/SNP/snp_ref.cgi?rs=rs74902811) |
|  | GAC A[C/T]G GGC | 511 | T | M | -0.07 | Neutral | [rs74902811](http://www.ncbi.nlm.nih.gov/projects/SNP/snp_ref.cgi?rs=rs74902811) |
|  | GAC A[C/T]G GGC | 405 | T | M | -0.99 | Neutral | [rs74902811](http://www.ncbi.nlm.nih.gov/projects/SNP/snp_ref.cgi?rs=rs74902811) |
| 2,190670446,C,T | GGC CA[C/T] ATA | 128 | H | H | 0 | Neutral |  |
|  | GGC CA[C/T] ATA | 128 | H | H | 0 | Neutral |  |
|  | GGC CA[C/T] ATA | 128 | H | H | 0 | Neutral |  |
|  | GGC CA[C/T] ATA | 67 | H | H | 0 | Neutral |  |
|  | GGC CA[C/T] ATA | 128 | H | H | 0 | Neutral |  |
|  | GGC CA[C/T] ATA | 128 | H | H | 0 | Neutral |  |
|  | GGC CA[C/T] ATA | 128 | H | H | 0 | Neutral |  |
|  | GGC CA[C/T] ATA | 128 | H | H | 0 | Neutral |  |
| 9,139402533,C,T | AAC AC[G/A] CAC | 1128 | T | T | 0 | Neutral |  |
| 11,108138045,C,T | GAA [C/T]CT GGA | 872 | P | S | -0.2 | Neutral | [rs3218673](http://www.ncbi.nlm.nih.gov/projects/SNP/snp_ref.cgi?rs=rs3218673) |
|  | GAA [C/T]CT GGA | 872 | P | S | -0.2 | Neutral | [rs3218673](http://www.ncbi.nlm.nih.gov/projects/SNP/snp_ref.cgi?rs=rs3218673) |
|  | GAA [C/T]CT GGA | 132 | P | S | -0.25 | Neutral | [rs3218673](http://www.ncbi.nlm.nih.gov/projects/SNP/snp_ref.cgi?rs=rs3218673) |
|  | GAA [C/T]CT GGA | 872 | P | S | -0.55 | Neutral | [rs3218673](http://www.ncbi.nlm.nih.gov/projects/SNP/snp_ref.cgi?rs=rs3218673) |
| 3,14193889,C,T | TCC AG[G/A] GAC | 687 | R | R | 0 | Neutral | [rs2227998](http://www.ncbi.nlm.nih.gov/projects/SNP/snp_ref.cgi?rs=rs2227998) |
|  | TCC AG[G/A] GAC | 650 | R | R | 0 | Neutral | [rs2227998](http://www.ncbi.nlm.nih.gov/projects/SNP/snp_ref.cgi?rs=rs2227998) |
|  | CAG [G/A]GA CAC | 130 | G | R | 1.4 | Neutral | [rs2227998](http://www.ncbi.nlm.nih.gov/projects/SNP/snp_ref.cgi?rs=rs2227998) |
| 3,121208833,G,C | CAG A[C/G]A TGT | 982 | T | R | -0.34 | Neutral | [rs3218649](http://www.ncbi.nlm.nih.gov/projects/SNP/snp_ref.cgi?rs=rs3218649) |
|  | CAG A[C/G]A TGT | 1118 | T | R | -0.34 | Neutral | [rs3218649](http://www.ncbi.nlm.nih.gov/projects/SNP/snp_ref.cgi?rs=rs3218649) |
|  | CAG A[C/G]A TGT | 605 | T | R | -0.28 | Neutral | [rs3218649](http://www.ncbi.nlm.nih.gov/projects/SNP/snp_ref.cgi?rs=rs3218649) |
| 5,74877266,C,T | ACA GC[C/T] AGT | 309 | A | A | 0 | Neutral | [rs3213801](http://www.ncbi.nlm.nih.gov/projects/SNP/snp_ref.cgi?rs=rs3213801) |
|  | ACA GC[C/T] AGT | 309 | A | A | 0 | Neutral | [rs3213801](http://www.ncbi.nlm.nih.gov/projects/SNP/snp_ref.cgi?rs=rs3213801) |
|  | ACA GC[C/T] AGT | 219 | A | A | 0 | Neutral | [rs3213801](http://www.ncbi.nlm.nih.gov/projects/SNP/snp_ref.cgi?rs=rs3213801) |
|  | ACA GC[C/T] AGT | 243 | A | A | 0 | Neutral | [rs3213801](http://www.ncbi.nlm.nih.gov/projects/SNP/snp_ref.cgi?rs=rs3213801) |
|  | ACA GC[C/T] AGT | 219 | A | A | 0 | Neutral | [rs3213801](http://www.ncbi.nlm.nih.gov/projects/SNP/snp_ref.cgi?rs=rs3213801) |
|  | ACA GC[C/T] AGT | 309 | A | A | 0 | Neutral | [rs3213801](http://www.ncbi.nlm.nih.gov/projects/SNP/snp_ref.cgi?rs=rs3213801) |
|  | ACA GC[C/T] AGT | 309 | A | A | 0 | Neutral | [rs3213801](http://www.ncbi.nlm.nih.gov/projects/SNP/snp_ref.cgi?rs=rs3213801) |
|  | ACA GC[C/T] AGT | 309 | A | A | 0 | Neutral | [rs3213801](http://www.ncbi.nlm.nih.gov/projects/SNP/snp_ref.cgi?rs=rs3213801) |
|  | ACA GC[C/T] AGT | 309 | A | A | 0 | Neutral | [rs3213801](http://www.ncbi.nlm.nih.gov/projects/SNP/snp_ref.cgi?rs=rs3213801) |
|  | ACA GC[C/T] AGT | 309 | A | A | 0 | Neutral | [rs3213801](http://www.ncbi.nlm.nih.gov/projects/SNP/snp_ref.cgi?rs=rs3213801) |
| 5,80149981,A,G | GGA C[A/G]G AGT | 949 | Q | R | 0.58 | Neutral | [rs184967](http://www.ncbi.nlm.nih.gov/projects/SNP/snp_ref.cgi?rs=rs184967) |
|  | GGA C[A/G]G AGT | 940 | Q | R | 0.58 | Neutral | [rs184967](http://www.ncbi.nlm.nih.gov/projects/SNP/snp_ref.cgi?rs=rs184967) |
| 8,90958422,T,C | AAT CC[A/G] TCT | 672 | P | P | 0 | Neutral | [rs1061302](http://www.ncbi.nlm.nih.gov/projects/SNP/snp_ref.cgi?rs=rs1061302) |
|  | AAT CC[A/G] TCT | 590 | P | P | 0 | Neutral | [rs1061302](http://www.ncbi.nlm.nih.gov/projects/SNP/snp_ref.cgi?rs=rs1061302) |
| 12,124144395,A,G | CCC CC[A/G] GTT | 205 | P | P | 0 | Neutral | [rs1051793](http://www.ncbi.nlm.nih.gov/projects/SNP/snp_ref.cgi?rs=rs1051793) |
|  | CCC CC[A/G] GTT | 132 | P | P | 0 | Neutral | [rs1051793](http://www.ncbi.nlm.nih.gov/projects/SNP/snp_ref.cgi?rs=rs1051793) |
|  | CCC [A/G]GT TCA | 198 | S | G | -0.87 | Neutral | [rs1051793](http://www.ncbi.nlm.nih.gov/projects/SNP/snp_ref.cgi?rs=rs1051793) |
|  | CCC CC[A/G] GTT | 196 | P | P | 0 | Neutral | [rs1051793](http://www.ncbi.nlm.nih.gov/projects/SNP/snp_ref.cgi?rs=rs1051793) |
|  | CCC CC[A/G] GTT | 203 | P | P | 0 | Neutral | [rs1051793](http://www.ncbi.nlm.nih.gov/projects/SNP/snp_ref.cgi?rs=rs1051793) |
|  | CCC CC[A/G] GTT | 246 | P | P | 0 | Neutral | [rs1051793](http://www.ncbi.nlm.nih.gov/projects/SNP/snp_ref.cgi?rs=rs1051793) |
| 8,42220154,G,A | GTG [G/A]AG CAG | 216 | E | K | -1.25 | Neutral |  |
|  | GTG [G/A]AG CAG | 32 | E | K | -1.31 | Neutral |  |
|  | GTG [G/A]AG CAG | 74 | E | K | -1.16 | Neutral |  |
|  | GTG [G/A]AG CAG | 251 | E | K | -1.11 | Neutral |  |
|  | GTG [G/A]AG CAG | 62 | E | K | -1.16 | Neutral |  |
| 4,178231152,C,A | ATT CG[C/A] GCG | 15 | R | R | 0 | Neutral | [rs10013040](http://www.ncbi.nlm.nih.gov/projects/SNP/snp_ref.cgi?rs=rs10013040) |
|  | ATT CG[C/A] GCG | 15 | R | R | 0 | Neutral | [rs10013040](http://www.ncbi.nlm.nih.gov/projects/SNP/snp_ref.cgi?rs=rs10013040) |
| 10,103340081,G,A | GTC GA[C/T] GTG | 429 | D | D | 0 | Neutral | [rs41562219](http://www.ncbi.nlm.nih.gov/projects/SNP/snp_ref.cgi?rs=rs41562219) |
|  | GTC GA[C/T] GTG | 152 | D | D | 0 | Neutral | [rs41562219](http://www.ncbi.nlm.nih.gov/projects/SNP/snp_ref.cgi?rs=rs41562219) |
|  | GTC GA[C/T] GTG | 429 | D | D | 0 | Neutral | [rs41562219](http://www.ncbi.nlm.nih.gov/projects/SNP/snp_ref.cgi?rs=rs41562219) |
|  | GTC GA[C/T] GTG | 154 | D | D | 0 | Neutral | [rs41562219](http://www.ncbi.nlm.nih.gov/projects/SNP/snp_ref.cgi?rs=rs41562219) |
|  | GTC GA[C/T] GTG | 429 | D | D | 0 | Neutral | [rs41562219](http://www.ncbi.nlm.nih.gov/projects/SNP/snp_ref.cgi?rs=rs41562219) |
|  | GTC GA[C/T] GTG | 102 | D | D | 0 | Neutral | [rs41562219](http://www.ncbi.nlm.nih.gov/projects/SNP/snp_ref.cgi?rs=rs41562219) |
|  | GTC GA[C/T] GTG | 429 | D | D | 0 | Neutral | [rs41562219](http://www.ncbi.nlm.nih.gov/projects/SNP/snp_ref.cgi?rs=rs41562219) |
|  | GTC GA[C/T] GTG | 341 | D | D | 0 | Neutral | [rs41562219](http://www.ncbi.nlm.nih.gov/projects/SNP/snp_ref.cgi?rs=rs41562219) |
|  | GTC GA[C/T] GTG | 429 | D | D | 0 | Neutral | [rs41562219](http://www.ncbi.nlm.nih.gov/projects/SNP/snp_ref.cgi?rs=rs41562219) |
|  | GTC GA[C/T] GTG | 166 | D | D | 0 | Neutral | [rs41562219](http://www.ncbi.nlm.nih.gov/projects/SNP/snp_ref.cgi?rs=rs41562219) |
|  | GTC GA[C/T] GTG | 337 | D | D | 0 | Neutral | [rs41562219](http://www.ncbi.nlm.nih.gov/projects/SNP/snp_ref.cgi?rs=rs41562219) |
|  | GTC GA[C/T] GTG | 152 | D | D | 0 | Neutral | [rs41562219](http://www.ncbi.nlm.nih.gov/projects/SNP/snp_ref.cgi?rs=rs41562219) |
| 7,6013153,A,G | GCT CT[T/C] AAC | 822 | L | L | 0 | Neutral | [rs10000](http://www.ncbi.nlm.nih.gov/projects/SNP/snp_ref.cgi?rs=rs10000) |
|  | GCT CT[T/C] AAC | 421 | L | L | 0 | Neutral | [rs10000](http://www.ncbi.nlm.nih.gov/projects/SNP/snp_ref.cgi?rs=rs10000) |
|  | GCT CT[T/C] AAC | 775 | L | L | 0 | Neutral | [rs10000](http://www.ncbi.nlm.nih.gov/projects/SNP/snp_ref.cgi?rs=rs10000) |
|  | GCT CT[T/C] AAC | 716 | L | L | 0 | Neutral | [rs10000](http://www.ncbi.nlm.nih.gov/projects/SNP/snp_ref.cgi?rs=rs10000) |
| 19,48621036,C,G | AAG GC[G/C] CTG | 814 | A | A | 0 | Neutral | [rs13436](http://www.ncbi.nlm.nih.gov/projects/SNP/snp_ref.cgi?rs=rs13436) |
|  | AAG GC[G/C] CTG | 746 | A | A | 0 | Neutral | [rs13436](http://www.ncbi.nlm.nih.gov/projects/SNP/snp_ref.cgi?rs=rs13436) |
|  | AAG GC[G/C] CTG | 783 | A | A | 0 | Neutral | [rs13436](http://www.ncbi.nlm.nih.gov/projects/SNP/snp_ref.cgi?rs=rs13436) |
| 8,42220154,G,A | GTG [G/A]AG CAG | 216 | E | K | -1.25 | Neutral |  |
|  | GTG [G/A]AG CAG | 32 | E | K | -1.31 | Neutral |  |
|  | GTG [G/A]AG CAG | 74 | E | K | -1.16 | Neutral |  |
|  | GTG [G/A]AG CAG | 251 | E | K | -1.11 | Neutral |  |
|  | GTG [G/A]AG CAG | 62 | E | K | -1.16 | Neutral |  |
| 3,58502941,G,A | CTC CG[C/T] CTG | 614 | R | R | 0 | Neutral |  |
|  | CTC CG[C/T] CTG | 58 | R | R | 0 | Neutral |  |
|  | CTC CG[C/T] CTG | 600 | R | R | 0 | Neutral |  |
| 18,20596832,G,A | GTT C[G/A]G AAA | 800 | R | Q | -1.49 | Neutral |  |
|  | GTT C[G/A]G AAA | 805 | R | Q | -1.49 | Neutral |  |
|  | GTT C[G/A]G AAA | 800 | R | Q | -1.49 | Neutral |  |
| 15,41020960,G,A | CGA GC[G/A] TTC | 194 | A | A | 0 | Neutral |  |
|  | CGA GC[G/A] TTC | 97 | A | A | 0 | Neutral |  |
|  | CGA GC[G/A] TTC | 195 | A | A | 0 | Neutral |  |
|  | CGA GC[G/A] TTC | 194 | A | A | 0 | Neutral |  |
|  | CGA GC[G/A] TTC | 195 | A | A | 0 | Neutral |  |
|  | CGA GC[G/A] TTC | 97 | A | A | 0 | Neutral |  |
| X,55033829,C,T | CCC TC[C/T] TCC | 506 | S | S | 0 | Neutral |  |
| 19,44050240,G,A | GGG G[C/T]G GAA | 482 | A | V | -1.28 | Neutral |  |
|  | GGG G[C/T]G GAA | 496 | A | V | -1.28 | Neutral |  |
|  | GGG G[C/T]G GAA | 451 | A | V | -1.16 | Neutral |  |
| 3,14193889,C,T | TCC AG[G/A] GAC | 687 | R | R | 0 | Neutral | [rs2227998](http://www.ncbi.nlm.nih.gov/projects/SNP/snp_ref.cgi?rs=rs2227998) |
|  | TCC AG[G/A] GAC | 650 | R | R | 0 | Neutral | [rs2227998](http://www.ncbi.nlm.nih.gov/projects/SNP/snp_ref.cgi?rs=rs2227998) |
|  | CAG [G/A]GA CAC | 130 | G | R | 1.4 | Neutral | [rs2227998](http://www.ncbi.nlm.nih.gov/projects/SNP/snp_ref.cgi?rs=rs2227998) |
| 10,50678317,C,G | AGG C[G/C]T TAC | 1230 | R | P | -2.29 | Neutral | [rs4253211](http://www.ncbi.nlm.nih.gov/projects/SNP/snp_ref.cgi?rs=rs4253211) |
|  | AGG C[G/C]T TAC | 607 | R | P | -2.42 | Neutral | [rs4253211](http://www.ncbi.nlm.nih.gov/projects/SNP/snp_ref.cgi?rs=rs4253211) |
|  | AGG C[G/C]T TAC | 600 | R | P | -2.42 | Neutral | [rs4253211](http://www.ncbi.nlm.nih.gov/projects/SNP/snp_ref.cgi?rs=rs4253211) |
| 10,50740876,G,C | TAC CT[C/G] TCC | 45 | L | L | 0 | Neutral | [rs2228524](http://www.ncbi.nlm.nih.gov/projects/SNP/snp_ref.cgi?rs=rs2228524) |
|  | TAC CT[C/G] TCC | 45 | L | L | 0 | Neutral | [rs2228524](http://www.ncbi.nlm.nih.gov/projects/SNP/snp_ref.cgi?rs=rs2228524) |
|  | TAC CT[C/G] TCC | 45 | L | L | 0 | Neutral | [rs2228524](http://www.ncbi.nlm.nih.gov/projects/SNP/snp_ref.cgi?rs=rs2228524) |
|  | TAC CT[C/G] TCC | 45 | L | L | 0 | Neutral | [rs2228524](http://www.ncbi.nlm.nih.gov/projects/SNP/snp_ref.cgi?rs=rs2228524) |
| 14,35872926,G,A | CTG GC[C/T] TTC | 102 | A | A | 0 | Neutral | [rs1050851](http://www.ncbi.nlm.nih.gov/projects/SNP/snp_ref.cgi?rs=rs1050851) |
|  | CTG GC[C/T] TTC | 12 | A | A | 0 | Neutral | [rs1050851](http://www.ncbi.nlm.nih.gov/projects/SNP/snp_ref.cgi?rs=rs1050851) |
|  | CTG GC[C/T] TTC | 102 | A | A | 0 | Neutral | [rs1050851](http://www.ncbi.nlm.nih.gov/projects/SNP/snp_ref.cgi?rs=rs1050851) |
| 13,32906729,A,C | GCA [A/C]AT CAG | 372 | N | H | -0.6 | Neutral | [rs144848](http://www.ncbi.nlm.nih.gov/projects/SNP/snp_ref.cgi?rs=rs144848) |
|  | GCA [A/C]AT CAG | 370 | N | H | -1.55 | Neutral | [rs144848](http://www.ncbi.nlm.nih.gov/projects/SNP/snp_ref.cgi?rs=rs144848) |
|  | GCA [A/C]AT CAG | 372 | N | H | -0.6 | Neutral | [rs144848](http://www.ncbi.nlm.nih.gov/projects/SNP/snp_ref.cgi?rs=rs144848) |
| 10,50681033,G,A | GTG GG[C/T] GGC | 917 | G | G | 0 | Neutral | [rs2229760](http://www.ncbi.nlm.nih.gov/projects/SNP/snp_ref.cgi?rs=rs2229760) |
|  | GTG GG[C/T] GGC | 294 | G | G | 0 | Neutral | [rs2229760](http://www.ncbi.nlm.nih.gov/projects/SNP/snp_ref.cgi?rs=rs2229760) |
|  | GTG GG[C/T] GGC | 287 | G | G | 0 | Neutral | [rs2229760](http://www.ncbi.nlm.nih.gov/projects/SNP/snp_ref.cgi?rs=rs2229760) |
| 20,10653469,C,T | GCC GG[G/A] GGG | 89 | G | G | 0 | Neutral | [rs1051415](http://www.ncbi.nlm.nih.gov/projects/SNP/snp_ref.cgi?rs=rs1051415) |
| 14,35873770,G,A | CTG GA[C/T] GAC | 27 | D | D | 0 | Neutral | [rs1957106](http://www.ncbi.nlm.nih.gov/projects/SNP/snp_ref.cgi?rs=rs1957106) |
|  | CTG GA[C/T] GAC | 27 | D | D | 0 | Neutral | [rs1957106](http://www.ncbi.nlm.nih.gov/projects/SNP/snp_ref.cgi?rs=rs1957106) |
|  | CTG GA[C/T] GAC | 27 | D | D | 0 | Neutral | [rs1957106](http://www.ncbi.nlm.nih.gov/projects/SNP/snp_ref.cgi?rs=rs1957106) |
|  | CTG GA[C/T] GAC | 27 | D | D | 0 | Neutral | [rs1957106](http://www.ncbi.nlm.nih.gov/projects/SNP/snp_ref.cgi?rs=rs1957106) |
| 19,44047550,T,C | CCG CA[A/G] GCC | 632 | Q | Q | 0 | Neutral | [rs3547](http://www.ncbi.nlm.nih.gov/projects/SNP/snp_ref.cgi?rs=rs3547) |
|  | CCG CA[A/G] GCC | 646 | Q | Q | 0 | Neutral | [rs3547](http://www.ncbi.nlm.nih.gov/projects/SNP/snp_ref.cgi?rs=rs3547) |
|  | CCG CA[A/G] GCC | 601 | Q | Q | 0 | Neutral | [rs3547](http://www.ncbi.nlm.nih.gov/projects/SNP/snp_ref.cgi?rs=rs3547) |
| 19,30314666,C,T | AGC AG[C/T] GGG | 405 | S | S | 0 | Neutral | [rs7257694](http://www.ncbi.nlm.nih.gov/projects/SNP/snp_ref.cgi?rs=rs7257694) |
|  | AGC AG[C/T] GGG | 362 | S | S | 0 | Neutral | [rs7257694](http://www.ncbi.nlm.nih.gov/projects/SNP/snp_ref.cgi?rs=rs7257694) |
|  | AGC AG[C/T] GGG | 390 | S | S | 0 | Neutral | [rs7257694](http://www.ncbi.nlm.nih.gov/projects/SNP/snp_ref.cgi?rs=rs7257694) |
| 8,42220154,G,A | GTG [G/A]AG CAG | 216 | E | K | -1.25 | Neutral |  |
|  | GTG [G/A]AG CAG | 32 | E | K | -1.31 | Neutral |  |
|  | GTG [G/A]AG CAG | 74 | E | K | -1.16 | Neutral |  |
|  | GTG [G/A]AG CAG | 251 | E | K | -1.11 | Neutral |  |
|  | GTG [G/A]AG CAG | 62 | E | K | -1.16 | Neutral |  |
| 8,11640784,A,G | TCC CC[A/G] GTG | 188 | P | P | 0 | Neutral | [rs8191642](http://www.ncbi.nlm.nih.gov/projects/SNP/snp_ref.cgi?rs=rs8191642) |
|  | TCC CC[A/G] GTG | 173 | P | P | 0 | Neutral | [rs8191642](http://www.ncbi.nlm.nih.gov/projects/SNP/snp_ref.cgi?rs=rs8191642) |
|  | TCC CC[A/G] GTG | 127 | P | P | 0 | Neutral | [rs8191642](http://www.ncbi.nlm.nih.gov/projects/SNP/snp_ref.cgi?rs=rs8191642) |
|  | TCC CC[A/G] GTG | 188 | P | P | 0 | Neutral | [rs8191642](http://www.ncbi.nlm.nih.gov/projects/SNP/snp_ref.cgi?rs=rs8191642) |
|  | TCC CC[A/G] GTG | 188 | P | P | 0 | Neutral | [rs8191642](http://www.ncbi.nlm.nih.gov/projects/SNP/snp_ref.cgi?rs=rs8191642) |
|  | TCC CC[A/G] GTG | 72 | P | P | 0 | Neutral | [rs8191642](http://www.ncbi.nlm.nih.gov/projects/SNP/snp_ref.cgi?rs=rs8191642) |
| 1,45797505,C,G | GGA CA[G/C] TGC | 311 | Q | H | -1.03 | Neutral | [rs3219489](http://www.ncbi.nlm.nih.gov/projects/SNP/snp_ref.cgi?rs=rs3219489) |
|  | GGA CA[G/C] TGC | 310 | Q | H | -1.03 | Neutral | [rs3219489](http://www.ncbi.nlm.nih.gov/projects/SNP/snp_ref.cgi?rs=rs3219489) |
|  | GGA CA[G/C] TGC | 335 | Q | H | -1.03 | Neutral | [rs3219489](http://www.ncbi.nlm.nih.gov/projects/SNP/snp_ref.cgi?rs=rs3219489) |
|  | GGA CA[G/C] TGC | 321 | Q | H | -1.03 | Neutral | [rs3219489](http://www.ncbi.nlm.nih.gov/projects/SNP/snp_ref.cgi?rs=rs3219489) |
|  | GGA CA[G/C] TGC | 310 | Q | H | -1.03 | Neutral | [rs3219489](http://www.ncbi.nlm.nih.gov/projects/SNP/snp_ref.cgi?rs=rs3219489) |
|  | GGA CA[G/C] TGC | 325 | Q | H | -1.03 | Neutral | [rs3219489](http://www.ncbi.nlm.nih.gov/projects/SNP/snp_ref.cgi?rs=rs3219489) |
|  | GGA CA[G/C] TGC | 324 | Q | H | -1.03 | Neutral | [rs3219489](http://www.ncbi.nlm.nih.gov/projects/SNP/snp_ref.cgi?rs=rs3219489) |
|  | GGA CA[G/C] TGC | 310 | Q | H | -1.03 | Neutral | [rs3219489](http://www.ncbi.nlm.nih.gov/projects/SNP/snp_ref.cgi?rs=rs3219489) |
|  | GGA CA[G/C] TGC | 338 | Q | H | -1.03 | Neutral | [rs3219489](http://www.ncbi.nlm.nih.gov/projects/SNP/snp_ref.cgi?rs=rs3219489) |
|  | GGA CA[G/C] TGC | 321 | Q | H | -1.03 | Neutral | [rs3219489](http://www.ncbi.nlm.nih.gov/projects/SNP/snp_ref.cgi?rs=rs3219489) |
|  | GGA CA[G/C] TGC | 182 | Q | H | -1.03 | Neutral | [rs3219489](http://www.ncbi.nlm.nih.gov/projects/SNP/snp_ref.cgi?rs=rs3219489) |
|  | GGA CA[G/C] TGC | 182 | Q | H | -1.03 | Neutral | [rs3219489](http://www.ncbi.nlm.nih.gov/projects/SNP/snp_ref.cgi?rs=rs3219489) |
|  | GAC A[G/C]T GCC | 116 | S | T | 0.02 | Neutral | [rs3219489](http://www.ncbi.nlm.nih.gov/projects/SNP/snp_ref.cgi?rs=rs3219489) |
| 1,242042545,A,G | GAA G[A/G]G GCA | 670 | E | G | -1.55 | Neutral | [rs1776148](http://www.ncbi.nlm.nih.gov/projects/SNP/snp_ref.cgi?rs=rs1776148) |
|  | GAA G[A/G]G GCA | 670 | E | G | -1.55 | Neutral | [rs1776148](http://www.ncbi.nlm.nih.gov/projects/SNP/snp_ref.cgi?rs=rs1776148) |
|  | GAA G[A/G]G GCA | 69 | E | G | -0.79 | Neutral | [rs1776148](http://www.ncbi.nlm.nih.gov/projects/SNP/snp_ref.cgi?rs=rs1776148) |
|  | GAA G[A/G]G GCA | 670 | E | G | -1.51 | Neutral | [rs1776148](http://www.ncbi.nlm.nih.gov/projects/SNP/snp_ref.cgi?rs=rs1776148) |
| 3,14193889,C,T | TCC AG[G/A] GAC | 687 | R | R | 0 | Neutral | [rs2227998](http://www.ncbi.nlm.nih.gov/projects/SNP/snp_ref.cgi?rs=rs2227998) |
|  | TCC AG[G/A] GAC | 650 | R | R | 0 | Neutral | [rs2227998](http://www.ncbi.nlm.nih.gov/projects/SNP/snp_ref.cgi?rs=rs2227998) |
|  | CAG [G/A]GA CAC | 130 | G | R | 1.4 | Neutral | [rs2227998](http://www.ncbi.nlm.nih.gov/projects/SNP/snp_ref.cgi?rs=rs2227998) |
| X,66765627,G,A | AGG GA[G/A] GCC | 213 | E | E | 0 | Neutral | [rs6152](http://www.ncbi.nlm.nih.gov/projects/SNP/snp_ref.cgi?rs=rs6152) |
|  | AGG GA[G/A] GCC | 213 | E | E | 0 | Neutral | [rs6152](http://www.ncbi.nlm.nih.gov/projects/SNP/snp_ref.cgi?rs=rs6152) |
|  | AGG GA[G/A] GCC | 213 | E | E | 0 | Neutral | [rs6152](http://www.ncbi.nlm.nih.gov/projects/SNP/snp_ref.cgi?rs=rs6152) |
|  | AGG GA[G/A] GCC | 213 | E | E | 0 | Neutral | [rs6152](http://www.ncbi.nlm.nih.gov/projects/SNP/snp_ref.cgi?rs=rs6152) |
|  | AGG GA[G/A] GCC | 23 | E | E | 0 | Neutral | [rs6152](http://www.ncbi.nlm.nih.gov/projects/SNP/snp_ref.cgi?rs=rs6152) |
|  | AGG GA[G/A] GCC | 205 | E | E | 0 | Neutral | [rs6152](http://www.ncbi.nlm.nih.gov/projects/SNP/snp_ref.cgi?rs=rs6152) |
| 20,10620386,A,G | GAT TA[T/C] GAG | 1139 | Y | Y | 0 | Neutral | [rs1051419](http://www.ncbi.nlm.nih.gov/projects/SNP/snp_ref.cgi?rs=rs1051419) |
|  | GAT TA[T/C] GAG | 980 | Y | Y | 0 | Neutral | [rs1051419](http://www.ncbi.nlm.nih.gov/projects/SNP/snp_ref.cgi?rs=rs1051419) |
| 21,42845383,A,G | AGG AT[T/C] GTG | 256 | I | I | 0 | Neutral | [rs17854725](http://www.ncbi.nlm.nih.gov/projects/SNP/snp_ref.cgi?rs=rs17854725) |
|  | AGG AT[T/C] GTG | 293 | I | I | 0 | Neutral | [rs17854725](http://www.ncbi.nlm.nih.gov/projects/SNP/snp_ref.cgi?rs=rs17854725) |
|  | AGG AT[T/C] GTG | 256 | I | I | 0 | Neutral | [rs17854725](http://www.ncbi.nlm.nih.gov/projects/SNP/snp_ref.cgi?rs=rs17854725) |
|  | AGG AT[T/C] GTG | 256 | I | I | 0 | Neutral | [rs17854725](http://www.ncbi.nlm.nih.gov/projects/SNP/snp_ref.cgi?rs=rs17854725) |
|  | AGG AT[T/C] GTG | 216 | I | I | 0 | Neutral | [rs17854725](http://www.ncbi.nlm.nih.gov/projects/SNP/snp_ref.cgi?rs=rs17854725) |
| 12,124144395,A,G | CCC CC[A/G] GTT | 205 | P | P | 0 | Neutral | [rs1051793](http://www.ncbi.nlm.nih.gov/projects/SNP/snp_ref.cgi?rs=rs1051793) |
|  | CCC CC[A/G] GTT | 132 | P | P | 0 | Neutral | [rs1051793](http://www.ncbi.nlm.nih.gov/projects/SNP/snp_ref.cgi?rs=rs1051793) |
|  | CCC [A/G]GT TCA | 198 | S | G | -0.87 | Neutral | [rs1051793](http://www.ncbi.nlm.nih.gov/projects/SNP/snp_ref.cgi?rs=rs1051793) |
|  | CCC CC[A/G] GTT | 196 | P | P | 0 | Neutral | [rs1051793](http://www.ncbi.nlm.nih.gov/projects/SNP/snp_ref.cgi?rs=rs1051793) |
|  | CCC CC[A/G] GTT | 203 | P | P | 0 | Neutral | [rs1051793](http://www.ncbi.nlm.nih.gov/projects/SNP/snp_ref.cgi?rs=rs1051793) |
|  | CCC CC[A/G] GTT | 246 | P | P | 0 | Neutral | [rs1051793](http://www.ncbi.nlm.nih.gov/projects/SNP/snp_ref.cgi?rs=rs1051793) |
| 1,242030151,A,G | AGT C[A/G]T AGT | 354 | H | R | -0.92 | Neutral | [rs735943](http://www.ncbi.nlm.nih.gov/projects/SNP/snp_ref.cgi?rs=rs735943) |
|  | AGT C[A/G]T AGT | 354 | H | R | -0.92 | Neutral | [rs735943](http://www.ncbi.nlm.nih.gov/projects/SNP/snp_ref.cgi?rs=rs735943) |
|  | AGT C[A/G]T AGT | 354 | H | R | -0.92 | Neutral | [rs735943](http://www.ncbi.nlm.nih.gov/projects/SNP/snp_ref.cgi?rs=rs735943) |
| 7,6026775,T,C | CCT [A/G]AA ACT | 541 | K | E | 0.76 | Neutral | [rs2228006](http://www.ncbi.nlm.nih.gov/projects/SNP/snp_ref.cgi?rs=rs2228006) |
|  | CCT [A/G]AA ACT | 494 | K | E | 0.82 | Neutral | [rs2228006](http://www.ncbi.nlm.nih.gov/projects/SNP/snp_ref.cgi?rs=rs2228006) |
|  | CCT [A/G]AA ACT | 541 | K | E | 1.25 | Neutral | [rs2228006](http://www.ncbi.nlm.nih.gov/projects/SNP/snp_ref.cgi?rs=rs2228006) |
|  | CCT [A/G]AA ACT | 435 | K | E | 0.78 | Neutral | [rs2228006](http://www.ncbi.nlm.nih.gov/projects/SNP/snp_ref.cgi?rs=rs2228006) |
| 18,51820805,G,A | AGA [G/A]CA GGA | 731 | A | T | -0.4 | Neutral | [rs8305](http://www.ncbi.nlm.nih.gov/projects/SNP/snp_ref.cgi?rs=rs8305) |
|  | AGA [G/A]CA GGA | 652 | A | T | -0.42 | Neutral | [rs8305](http://www.ncbi.nlm.nih.gov/projects/SNP/snp_ref.cgi?rs=rs8305) |
| 4,39302029,T,C | GGC CC[A/G] TTT | 847 | P | P | 0 | Neutral | [rs2066786](http://www.ncbi.nlm.nih.gov/projects/SNP/snp_ref.cgi?rs=rs2066786) |
|  | GGC CC[A/G] TTT | 848 | P | P | 0 | Neutral | [rs2066786](http://www.ncbi.nlm.nih.gov/projects/SNP/snp_ref.cgi?rs=rs2066786) |
| 2,217012901,A,G | ACA AC[A/G] AAA | 524 | T | T | 0 | Neutral | [rs207906](http://www.ncbi.nlm.nih.gov/projects/SNP/snp_ref.cgi?rs=rs207906) |
|  | ACA AC[A/G] AAA | 524 | T | T | 0 | Neutral | [rs207906](http://www.ncbi.nlm.nih.gov/projects/SNP/snp_ref.cgi?rs=rs207906) |
| 1,45797505,C,G | GGA CA[G/C] TGC | 311 | Q | H | -1.03 | Neutral | [rs3219489](http://www.ncbi.nlm.nih.gov/projects/SNP/snp_ref.cgi?rs=rs3219489) |
|  | GGA CA[G/C] TGC | 310 | Q | H | -1.03 | Neutral | [rs3219489](http://www.ncbi.nlm.nih.gov/projects/SNP/snp_ref.cgi?rs=rs3219489) |
|  | GGA CA[G/C] TGC | 335 | Q | H | -1.03 | Neutral | [rs3219489](http://www.ncbi.nlm.nih.gov/projects/SNP/snp_ref.cgi?rs=rs3219489) |
|  | GGA CA[G/C] TGC | 321 | Q | H | -1.03 | Neutral | [rs3219489](http://www.ncbi.nlm.nih.gov/projects/SNP/snp_ref.cgi?rs=rs3219489) |
|  | GGA CA[G/C] TGC | 310 | Q | H | -1.03 | Neutral | [rs3219489](http://www.ncbi.nlm.nih.gov/projects/SNP/snp_ref.cgi?rs=rs3219489) |
|  | GGA CA[G/C] TGC | 325 | Q | H | -1.03 | Neutral | [rs3219489](http://www.ncbi.nlm.nih.gov/projects/SNP/snp_ref.cgi?rs=rs3219489) |
|  | GGA CA[G/C] TGC | 324 | Q | H | -1.03 | Neutral | [rs3219489](http://www.ncbi.nlm.nih.gov/projects/SNP/snp_ref.cgi?rs=rs3219489) |
|  | GGA CA[G/C] TGC | 310 | Q | H | -1.03 | Neutral | [rs3219489](http://www.ncbi.nlm.nih.gov/projects/SNP/snp_ref.cgi?rs=rs3219489) |
|  | GGA CA[G/C] TGC | 338 | Q | H | -1.03 | Neutral | [rs3219489](http://www.ncbi.nlm.nih.gov/projects/SNP/snp_ref.cgi?rs=rs3219489) |
|  | GGA CA[G/C] TGC | 321 | Q | H | -1.03 | Neutral | [rs3219489](http://www.ncbi.nlm.nih.gov/projects/SNP/snp_ref.cgi?rs=rs3219489) |
|  | GGA CA[G/C] TGC | 182 | Q | H | -1.03 | Neutral | [rs3219489](http://www.ncbi.nlm.nih.gov/projects/SNP/snp_ref.cgi?rs=rs3219489) |
|  | GGA CA[G/C] TGC | 182 | Q | H | -1.03 | Neutral | [rs3219489](http://www.ncbi.nlm.nih.gov/projects/SNP/snp_ref.cgi?rs=rs3219489) |
|  | GAC A[G/C]T GCC | 116 | S | T | 0.02 | Neutral | [rs3219489](http://www.ncbi.nlm.nih.gov/projects/SNP/snp_ref.cgi?rs=rs3219489) |
| 8,42220154,G,A | GTG [G/A]AG CAG | 216 | E | K | -1.25 | Neutral |  |
|  | GTG [G/A]AG CAG | 32 | E | K | -1.31 | Neutral |  |
|  | GTG [G/A]AG CAG | 74 | E | K | -1.16 | Neutral |  |
|  | GTG [G/A]AG CAG | 251 | E | K | -1.11 | Neutral |  |
|  | GTG [G/A]AG CAG | 62 | E | K | -1.16 | Neutral |  |
| 3,129155670,C,T | GAT [G/A]CT GAA | 273 | A | T | -0.9 | Neutral | [rs10342](http://www.ncbi.nlm.nih.gov/projects/SNP/snp_ref.cgi?rs=rs10342) |
|  | GAT [G/A]CT GAA | 273 | A | T | -0.9 | Neutral | [rs10342](http://www.ncbi.nlm.nih.gov/projects/SNP/snp_ref.cgi?rs=rs10342) |
|  | GAT [G/A]CT GAA | 273 | A | T | -0.93 | Neutral | [rs10342](http://www.ncbi.nlm.nih.gov/projects/SNP/snp_ref.cgi?rs=rs10342) |
|  | GAT [G/A]CT GAA | 273 | A | T | -0.8 | Neutral | [rs10342](http://www.ncbi.nlm.nih.gov/projects/SNP/snp_ref.cgi?rs=rs10342) |
| 17,41244000,T,C | CAG A[A/G]A GGA | 1183 | K | R | 0.49 | Neutral | [rs16942](http://www.ncbi.nlm.nih.gov/projects/SNP/snp_ref.cgi?rs=rs16942) |
|  | CAG A[A/G]A GGA | 887 | K | R | 0.29 | Neutral | [rs16942](http://www.ncbi.nlm.nih.gov/projects/SNP/snp_ref.cgi?rs=rs16942) |
|  | CAG A[A/G]A GGA | 1183 | K | R | 0.49 | Neutral | [rs16942](http://www.ncbi.nlm.nih.gov/projects/SNP/snp_ref.cgi?rs=rs16942) |
|  | CAG A[A/G]A GGA | 1183 | K | R | 0.4 | Neutral | [rs16942](http://www.ncbi.nlm.nih.gov/projects/SNP/snp_ref.cgi?rs=rs16942) |
|  | CAG A[A/G]A GGA | 1183 | K | R | 0.42 | Neutral | [rs16942](http://www.ncbi.nlm.nih.gov/projects/SNP/snp_ref.cgi?rs=rs16942) |
|  | CAG A[A/G]A GGA | 1136 | K | R | 0.38 | Neutral | [rs16942](http://www.ncbi.nlm.nih.gov/projects/SNP/snp_ref.cgi?rs=rs16942) |
|  | CAG A[A/G]A GGA | 1183 | K | R | 0.42 | Neutral | [rs16942](http://www.ncbi.nlm.nih.gov/projects/SNP/snp_ref.cgi?rs=rs16942) |
| 13,32906729,A,C | GCA [A/C]AT CAG | 372 | N | H | -0.6 | Neutral | [rs144848](http://www.ncbi.nlm.nih.gov/projects/SNP/snp_ref.cgi?rs=rs144848) |
|  | GCA [A/C]AT CAG | 370 | N | H | -1.55 | Neutral | [rs144848](http://www.ncbi.nlm.nih.gov/projects/SNP/snp_ref.cgi?rs=rs144848) |
|  | GCA [A/C]AT CAG | 372 | N | H | -0.6 | Neutral | [rs144848](http://www.ncbi.nlm.nih.gov/projects/SNP/snp_ref.cgi?rs=rs144848) |
| 3,186509517,G,A | ATT GC[C/T] GGG | 266 | A | A | 0 | Neutral | [rs187868](http://www.ncbi.nlm.nih.gov/projects/SNP/snp_ref.cgi?rs=rs187868) |
|  | ATT GC[C/T] GGG | 266 | A | A | 0 | Neutral | [rs187868](http://www.ncbi.nlm.nih.gov/projects/SNP/snp_ref.cgi?rs=rs187868) |
|  | ATT GC[C/T] GGG | 266 | A | A | 0 | Neutral | [rs187868](http://www.ncbi.nlm.nih.gov/projects/SNP/snp_ref.cgi?rs=rs187868) |
|  | ATT GC[C/T] GGG | 41 | A | A | 0 | Neutral | [rs187868](http://www.ncbi.nlm.nih.gov/projects/SNP/snp_ref.cgi?rs=rs187868) |
| 1,242042545,A,G | GAA G[A/G]G GCA | 670 | E | G | -1.55 | Neutral | [rs1776148](http://www.ncbi.nlm.nih.gov/projects/SNP/snp_ref.cgi?rs=rs1776148) |
|  | GAA G[A/G]G GCA | 670 | E | G | -1.55 | Neutral | [rs1776148](http://www.ncbi.nlm.nih.gov/projects/SNP/snp_ref.cgi?rs=rs1776148) |
|  | GAA G[A/G]G GCA | 69 | E | G | -0.79 | Neutral | [rs1776148](http://www.ncbi.nlm.nih.gov/projects/SNP/snp_ref.cgi?rs=rs1776148) |
|  | GAA G[A/G]G GCA | 670 | E | G | -1.51 | Neutral | [rs1776148](http://www.ncbi.nlm.nih.gov/projects/SNP/snp_ref.cgi?rs=rs1776148) |
| 19,50905310,G,A | ATC A[G/A]C CGG | 174 | S | N | -0.92 | Neutral | [rs1726803](http://www.ncbi.nlm.nih.gov/projects/SNP/snp_ref.cgi?rs=rs1726803) |
|  | ATC A[G/A]C CGG | 173 | S | N | -1.03 | Neutral | [rs1726803](http://www.ncbi.nlm.nih.gov/projects/SNP/snp_ref.cgi?rs=rs1726803) |
| 10,50681033,G,A | GTG GG[C/T] GGC | 917 | G | G | 0 | Neutral | [rs2229760](http://www.ncbi.nlm.nih.gov/projects/SNP/snp_ref.cgi?rs=rs2229760) |
|  | GTG GG[C/T] GGC | 294 | G | G | 0 | Neutral | [rs2229760](http://www.ncbi.nlm.nih.gov/projects/SNP/snp_ref.cgi?rs=rs2229760) |
|  | GTG GG[C/T] GGC | 287 | G | G | 0 | Neutral | [rs2229760](http://www.ncbi.nlm.nih.gov/projects/SNP/snp_ref.cgi?rs=rs2229760) |
| 18,51820805,G,A | AGA [G/A]CA GGA | 731 | A | T | -0.4 | Neutral | [rs8305](http://www.ncbi.nlm.nih.gov/projects/SNP/snp_ref.cgi?rs=rs8305) |
|  | AGA [G/A]CA GGA | 652 | A | T | -0.42 | Neutral | [rs8305](http://www.ncbi.nlm.nih.gov/projects/SNP/snp_ref.cgi?rs=rs8305) |
| 3,121208338,G,A | GTG A[C/T]T TGT | 1147 | T | I | -1.69 | Neutral |  |
|  | GTG A[C/T]T TGT | 1283 | T | I | -1.69 | Neutral |  |
|  | GTG A[C/T]T TGT | 770 | T | I | -1.69 | Neutral |  |
| 19,45868309,T,G | TGC CG[A/C] TTC | 132 | R | R | 0 | Neutral | [rs238406](http://www.ncbi.nlm.nih.gov/projects/SNP/snp_ref.cgi?rs=rs238406) |
|  | TGC CG[A/C] TTC | 106 | R | R | 0 | Neutral | [rs238406](http://www.ncbi.nlm.nih.gov/projects/SNP/snp_ref.cgi?rs=rs238406) |
|  | TGC CG[A/C] TTC | 132 | R | R | 0 | Neutral | [rs238406](http://www.ncbi.nlm.nih.gov/projects/SNP/snp_ref.cgi?rs=rs238406) |
|  | TGC CG[A/C] TTC | 156 | R | R | 0 | Neutral | [rs238406](http://www.ncbi.nlm.nih.gov/projects/SNP/snp_ref.cgi?rs=rs238406) |
|  | TGC CG[A/C] TTC | 132 | R | R | 0 | Neutral | [rs238406](http://www.ncbi.nlm.nih.gov/projects/SNP/snp_ref.cgi?rs=rs238406) |
| 1,45797505,C,G | GGA CA[G/C] TGC | 311 | Q | H | -1.03 | Neutral | [rs3219489](http://www.ncbi.nlm.nih.gov/projects/SNP/snp_ref.cgi?rs=rs3219489) |
|  | GGA CA[G/C] TGC | 310 | Q | H | -1.03 | Neutral | [rs3219489](http://www.ncbi.nlm.nih.gov/projects/SNP/snp_ref.cgi?rs=rs3219489) |
|  | GGA CA[G/C] TGC | 335 | Q | H | -1.03 | Neutral | [rs3219489](http://www.ncbi.nlm.nih.gov/projects/SNP/snp_ref.cgi?rs=rs3219489) |
|  | GGA CA[G/C] TGC | 321 | Q | H | -1.03 | Neutral | [rs3219489](http://www.ncbi.nlm.nih.gov/projects/SNP/snp_ref.cgi?rs=rs3219489) |
|  | GGA CA[G/C] TGC | 310 | Q | H | -1.03 | Neutral | [rs3219489](http://www.ncbi.nlm.nih.gov/projects/SNP/snp_ref.cgi?rs=rs3219489) |
|  | GGA CA[G/C] TGC | 325 | Q | H | -1.03 | Neutral | [rs3219489](http://www.ncbi.nlm.nih.gov/projects/SNP/snp_ref.cgi?rs=rs3219489) |
|  | GGA CA[G/C] TGC | 324 | Q | H | -1.03 | Neutral | [rs3219489](http://www.ncbi.nlm.nih.gov/projects/SNP/snp_ref.cgi?rs=rs3219489) |
|  | GGA CA[G/C] TGC | 310 | Q | H | -1.03 | Neutral | [rs3219489](http://www.ncbi.nlm.nih.gov/projects/SNP/snp_ref.cgi?rs=rs3219489) |
|  | GGA CA[G/C] TGC | 338 | Q | H | -1.03 | Neutral | [rs3219489](http://www.ncbi.nlm.nih.gov/projects/SNP/snp_ref.cgi?rs=rs3219489) |
|  | GGA CA[G/C] TGC | 321 | Q | H | -1.03 | Neutral | [rs3219489](http://www.ncbi.nlm.nih.gov/projects/SNP/snp_ref.cgi?rs=rs3219489) |
|  | GGA CA[G/C] TGC | 182 | Q | H | -1.03 | Neutral | [rs3219489](http://www.ncbi.nlm.nih.gov/projects/SNP/snp_ref.cgi?rs=rs3219489) |
|  | GGA CA[G/C] TGC | 182 | Q | H | -1.03 | Neutral | [rs3219489](http://www.ncbi.nlm.nih.gov/projects/SNP/snp_ref.cgi?rs=rs3219489) |
|  | GAC A[G/C]T GCC | 116 | S | T | 0.02 | Neutral | [rs3219489](http://www.ncbi.nlm.nih.gov/projects/SNP/snp_ref.cgi?rs=rs3219489) |
| 17,33329049,G,A | TCC C[G/A]C AAG | 867 | R | H | -0.71 | Neutral | [rs3136025](http://www.ncbi.nlm.nih.gov/projects/SNP/snp_ref.cgi?rs=rs3136025) |
|  | TCC C[G/A]C AAG | 867 | R | H | -0.65 | Neutral | [rs3136025](http://www.ncbi.nlm.nih.gov/projects/SNP/snp_ref.cgi?rs=rs3136025) |
| 8,42220154,G,A | GTG [G/A]AG CAG | 216 | E | K | -1.25 | Neutral |  |
|  | GTG [G/A]AG CAG | 32 | E | K | -1.31 | Neutral |  |
|  | GTG [G/A]AG CAG | 74 | E | K | -1.16 | Neutral |  |
|  | GTG [G/A]AG CAG | 251 | E | K | -1.11 | Neutral |  |
|  | GTG [G/A]AG CAG | 62 | E | K | -1.16 | Neutral |  |
| 10,103344589,T,G | CCC [A/C]CC TCC | 221 | T | P | -0.8 | Neutral | [rs3730463](http://www.ncbi.nlm.nih.gov/projects/SNP/snp_ref.cgi?rs=rs3730463) |
|  | CCC [A/C]CC TCC | 221 | T | P | -0.89 | Neutral | [rs3730463](http://www.ncbi.nlm.nih.gov/projects/SNP/snp_ref.cgi?rs=rs3730463) |
|  | CCC [A/C]CC TCC | 221 | T | P | -0.8 | Neutral | [rs3730463](http://www.ncbi.nlm.nih.gov/projects/SNP/snp_ref.cgi?rs=rs3730463) |
|  | ACC CC[A/C] CCT | 78 | P | P | 0 | Neutral | [rs3730463](http://www.ncbi.nlm.nih.gov/projects/SNP/snp_ref.cgi?rs=rs3730463) |
|  | CCC [A/C]CC TCC | 221 | T | P | -0.8 | Neutral | [rs3730463](http://www.ncbi.nlm.nih.gov/projects/SNP/snp_ref.cgi?rs=rs3730463) |
|  | CCC [A/C]CC TCC | 133 | T | P | -0.8 | Neutral | [rs3730463](http://www.ncbi.nlm.nih.gov/projects/SNP/snp_ref.cgi?rs=rs3730463) |
|  | CCC [A/C]CC TCC | 221 | T | P | -0.89 | Neutral | [rs3730463](http://www.ncbi.nlm.nih.gov/projects/SNP/snp_ref.cgi?rs=rs3730463) |
|  | ACC CC[A/C] CCT | 78 | P | P | 0 | Neutral | [rs3730463](http://www.ncbi.nlm.nih.gov/projects/SNP/snp_ref.cgi?rs=rs3730463) |
|  | CCC [A/C]CC TCC | 221 | T | P | -0.8 | Neutral | [rs3730463](http://www.ncbi.nlm.nih.gov/projects/SNP/snp_ref.cgi?rs=rs3730463) |
|  | ACC CC[A/C] CCT | 78 | P | P | 0 | Neutral | [rs3730463](http://www.ncbi.nlm.nih.gov/projects/SNP/snp_ref.cgi?rs=rs3730463) |
|  | CCC [A/C]CC TCC | 232 | T | P | -0.96 | Neutral | [rs3730463](http://www.ncbi.nlm.nih.gov/projects/SNP/snp_ref.cgi?rs=rs3730463) |
| 7,6036980,G,C | TGT TC[C/G] GAT | 260 | S | S | 0 | Neutral | [rs1805319](http://www.ncbi.nlm.nih.gov/projects/SNP/snp_ref.cgi?rs=rs1805319) |
|  | TGT TC[C/G] GAT | 260 | S | S | 0 | Neutral | [rs1805319](http://www.ncbi.nlm.nih.gov/projects/SNP/snp_ref.cgi?rs=rs1805319) |
|  | TGT TC[C/G] GAT | 213 | S | S | 0 | Neutral | [rs1805319](http://www.ncbi.nlm.nih.gov/projects/SNP/snp_ref.cgi?rs=rs1805319) |
|  | TGT TC[C/G] GAT | 260 | S | S | 0 | Neutral | [rs1805319](http://www.ncbi.nlm.nih.gov/projects/SNP/snp_ref.cgi?rs=rs1805319) |
|  | TGT TC[C/G] GAT | 154 | S | S | 0 | Neutral | [rs1805319](http://www.ncbi.nlm.nih.gov/projects/SNP/snp_ref.cgi?rs=rs1805319) |
| 2,217001864,G,A | GAC AT[G/A] GTG | 389 | M | I | -2.4 | Neutral |  |
|  | GAC AT[G/A] GTG | 389 | M | I | -2.4 | Neutral |  |
| 3,129155801,C,T | GTT A[G/A]A AAG | 229 | R | K | -0.76 | Neutral |  |
|  | GTT A[G/A]A AAG | 229 | R | K | -0.63 | Neutral |  |
|  | GTT A[G/A]A AAG | 229 | R | K | -0.76 | Neutral |  |
|  | GTT A[G/A]A AAG | 229 | R | K | -0.66 | Neutral |  |
| 3,186522400,C,T | AAA [G/A]CC AAA | 35 | A | T | -0.31 | Neutral |  |
|  | AAA [G/A]CC AAA | 35 | A | T | -0.31 | Neutral |  |
|  | AAA [G/A]CC AAA | 35 | A | T | -0.2 | Neutral |  |
|  | AAA [G/A]CC AAA | 35 | A | T | -0.73 | Neutral |  |
|  | AAA [G/A]CC AAA | 35 | A | T | -0.24 | Neutral |  |
|  | AAA [G/A]CC AAA | 35 | A | T | -0.14 | Neutral |  |
|  | AAA [G/A]CC AAA | 35 | A | T | -0.31 | Neutral |  |
|  | AAA [G/A]CC AAA | 35 | A | T | -0.2 | Neutral |  |
|  | AAA [G/A]CC AAA | 35 | A | T | -0.2 | Neutral |  |
| 11,108137925,C,T | GAC [C/T]GT GGA | 832 | R | C | -0.84 | Neutral | [rs146482111](http://www.ncbi.nlm.nih.gov/projects/SNP/snp_ref.cgi?rs=rs146482111) |
|  | GAC [C/T]GT GGA | 832 | R | C | -0.84 | Neutral | [rs146482111](http://www.ncbi.nlm.nih.gov/projects/SNP/snp_ref.cgi?rs=rs146482111) |
|  | GAC [C/T]GT GGA | 92 | R | C | 0.29 | Neutral | [rs146482111](http://www.ncbi.nlm.nih.gov/projects/SNP/snp_ref.cgi?rs=rs146482111) |
|  | GAC [C/T]GT GGA | 832 | R | C | -0.86 | Neutral | [rs146482111](http://www.ncbi.nlm.nih.gov/projects/SNP/snp_ref.cgi?rs=rs146482111) |
| 10,50686412,A,T | CCA GA[T/A] AAA | 758 | D | E | -2.3 | Neutral |  |
|  | CCA GA[T/A] AAA | 167 | D | E | -2.4 | Neutral |  |
|  | CCA GA[T/A] AAA | 7 | D | E | -1.87 | Neutral |  |
|  | CCA GA[T/A] AAA | 128 | D | E | -2.17 | Neutral |  |
| 11,108165685,A,T | GTT T[A/T]T GAT | 1603 | Y | F | -1.26 | Neutral |  |
|  | GTT T[A/T]T GAT | 1603 | Y | F | -1.26 | Neutral |  |
| 2,128044513,A,T | GTT [T/A]CT GTG | 370 | S | T | -2.44 | Neutral |  |
|  | GTT [T/A]CT GTG | 306 | S | T | -2.47 | Neutral |  |
|  | ACC C[C/T]C AGT | 197 | P | L | 0.25 | Neutral |  |
|  | CCC [C/T]CA GTT | 196 | P | S | -2.49 | Neutral |  |
|  | CCC [C/T]CA GTT | 246 | P | S | -2.24 | Neutral |  |
| 12,124144402,G,A | CAT [G/A]TT GAC | 208 | V | I | -0.81 | Neutral |  |
|  | CAT [G/A]TT GAC | 135 | V | I | -0.79 | Neutral |  |
|  | TCA T[G/A]T | 200 | C | Y | 3 | Neutral |  |
|  | CAT [G/A]TT GAC | 199 | V | I | -0.82 | Neutral |  |
|  | CAT [G/A]TT GAC | 206 | V | I | -0.88 | Neutral |  |
|  | CAT [G/A]TT GAC | 249 | V | I | -0.81 | Neutral |  |
| 6,36652219,C,T | CTG T[C/T]A CTG | 114 | S | L | -1.87 | Neutral |  |
|  | CTG T[C/T]A CTG | 114 | S | L | -1.87 | Neutral |  |
|  | CTG T[C/T]A CTG | 114 | S | L | -1.87 | Neutral |  |
|  | CTG T[C/T]A CTG | 148 | S | L | -1.67 | Neutral |  |
| 12,133220120,C,T | CTG GT[G/A] CAC | 1439 | V | V | 0 | Neutral |  |
|  | CTG GT[G/A] CAC | 1450 | V | V | 0 | Neutral |  |
|  | CTG GT[G/A] CAC | 1412 | V | V | 0 | Neutral |  |
| 11,61077853,G,A | TTC T[C/T]C AGC | 772 | S | F | -1.35 | Neutral |  |
|  | TTC T[C/T]C AGC | 83 | S | F | -1.29 | Neutral |  |
|  | TTC T[C/T]C AGC | 772 | S | F | -1.35 | Neutral |  |
|  | TTC T[C/T]C AGC | 239 | S | F | -1.9 | Neutral |  |
|  | TTC T[C/T]C AGC | 83 | S | F | -0.33 | Neutral |  |
| 4,103517361,T,A | ACT G[T/A]A AAC | 456 | V | E | 0.95 | Neutral |  |
|  | ACT G[T/A]A AAC | 455 | V | E | 0.95 | Neutral |  |
|  | ACT G[T/A]A AAC | 455 | V | E | 0.95 | Neutral |  |
| 1,45799121,G,A | TGG TA[C/T] GAC | 77 | Y | Y | 0 | Neutral | [rs121908380](http://www.ncbi.nlm.nih.gov/projects/SNP/snp_ref.cgi?rs=rs121908380) |
|  | TGG TA[C/T] GAC | 76 | Y | Y | 0 | Neutral | [rs121908380](http://www.ncbi.nlm.nih.gov/projects/SNP/snp_ref.cgi?rs=rs121908380) |
|  | TGG TA[C/T] GAC | 101 | Y | Y | 0 | Neutral | [rs121908380](http://www.ncbi.nlm.nih.gov/projects/SNP/snp_ref.cgi?rs=rs121908380) |
|  | TGG TA[C/T] GAC | 87 | Y | Y | 0 | Neutral | [rs121908380](http://www.ncbi.nlm.nih.gov/projects/SNP/snp_ref.cgi?rs=rs121908380) |
|  | TGG TA[C/T] GAC | 76 | Y | Y | 0 | Neutral | [rs121908380](http://www.ncbi.nlm.nih.gov/projects/SNP/snp_ref.cgi?rs=rs121908380) |
|  | TGG TA[C/T] GAC | 91 | Y | Y | 0 | Neutral | [rs121908380](http://www.ncbi.nlm.nih.gov/projects/SNP/snp_ref.cgi?rs=rs121908380) |
|  | TGG TA[C/T] GAC | 90 | Y | Y | 0 | Neutral | [rs121908380](http://www.ncbi.nlm.nih.gov/projects/SNP/snp_ref.cgi?rs=rs121908380) |
|  | TGG TA[C/T] GAC | 87 | Y | Y | 0 | Neutral | [rs121908380](http://www.ncbi.nlm.nih.gov/projects/SNP/snp_ref.cgi?rs=rs121908380) |
|  | TGG TA[C/T] GAC | 76 | Y | Y | 0 | Neutral | [rs121908380](http://www.ncbi.nlm.nih.gov/projects/SNP/snp_ref.cgi?rs=rs121908380) |
|  | TGG TA[C/T] GAC | 104 | Y | Y | 0 | Neutral | [rs121908380](http://www.ncbi.nlm.nih.gov/projects/SNP/snp_ref.cgi?rs=rs121908380) |
|  | TGG TA[C/T] GAC | 87 | Y | Y | 0 | Neutral | [rs121908380](http://www.ncbi.nlm.nih.gov/projects/SNP/snp_ref.cgi?rs=rs121908380) |
|  | TGG TA[C/T] GAC | 90 | Y | Y | 0 | Neutral | [rs121908380](http://www.ncbi.nlm.nih.gov/projects/SNP/snp_ref.cgi?rs=rs121908380) |
|  | TGG TA[C/T] GAC | 77 | Y | Y | 0 | Neutral | [rs121908380](http://www.ncbi.nlm.nih.gov/projects/SNP/snp_ref.cgi?rs=rs121908380) |
|  | TGG TA[C/T] GAC | 82 | Y | Y | 0 | Neutral | [rs121908380](http://www.ncbi.nlm.nih.gov/projects/SNP/snp_ref.cgi?rs=rs121908380) |
| 7,73651764,G,A | TGT GA[C/T] GAG | 256 | D | D | 0 | Neutral |  |
|  | TGT GA[C/T] GAG | 222 | D | D | 0 | Neutral |  |
|  | TGT GA[C/T] GAG | 49 | D | D | 0 | Neutral |  |
|  | TGT GA[C/T] GAG | 112 | D | D | 0 | Neutral |  |
| 10,103344424,C,A | AGG [G/T]CC CTG | 276 | A | S | -1.35 | Neutral |  |
|  | AGG [G/T]CC CTG | 276 | A | S | -0.85 | Neutral |  |
|  | AGG [G/T]CC CTG | 276 | A | S | -1.35 | Neutral |  |
|  | GGA GG[G/T] CCC | 133 | G | G | 0 | Neutral |  |
|  | AGG [G/T]CC CTG | 276 | A | S | -1.35 | Neutral |  |
|  | AGG [G/T]CC CTG | 188 | A | S | -1.39 | Neutral |  |
|  | AGG [G/T]CC CTG | 276 | A | S | -0.85 | Neutral |  |
|  | GGA GG[G/T] CCC | 133 | G | G | 0 | Neutral |  |
|  | GGA GG[G/T] CCC | 133 | G | G | 0 | Neutral |  |
|  | AGG [G/T]CC CTG | 287 | A | S | -1.26 | Neutral |  |
| 5,137803081,C,T | CAG [C/T]TC ATC | 315 | L | F | -1.53 | Neutral |  |
| 17,33329049,G,A | TCC C[G/A]C AAG | 867 | R | H | -0.71 | Neutral | [rs3136025](http://www.ncbi.nlm.nih.gov/projects/SNP/snp_ref.cgi?rs=rs3136025) |
|  | TCC C[G/A]C AAG | 867 | R | H | -0.65 | Neutral | [rs3136025](http://www.ncbi.nlm.nih.gov/projects/SNP/snp_ref.cgi?rs=rs3136025) |
| 1,226576430,C,T | GAT G[G/A]A GTG | 215 | G | E | -0.69 | Neutral |  |
|  | GAT G[G/A]A GTG | 215 | G | E | -1.27 | Neutral |  |
| 22,42046729,C,T | CAG AT[C/T] TAT | 321 | I | I | 0 | Neutral |  |
|  | CAG AT[C/T] TAT | 321 | I | I | 0 | Neutral |  |
|  | CAG AT[C/T] TAT | 271 | I | I | 0 | Neutral |  |
|  | CAG AT[C/T] TAT | 321 | I | I | 0 | Neutral |  |
|  | CAG AT[C/T] TAT | 321 | I | I | 0 | Neutral |  |
|  | CAG AT[C/T] TAT | 280 | I | I | 0 | Neutral |  |
|  | CAG AT[C/T] TAT | 188 | I | I | 0 | Neutral |  |
| 2,47707976,A,G | ATG G[A/G]A CCA | 867 | E | G | -2.3 | Neutral |  |
|  | ATG G[A/G]A CCA | 867 | E | G | -1.6 | Neutral |  |
|  | ATG G[A/G]A CCA | 653 | E | G | -2.38 | Neutral |  |
|  | ATG G[A/G]A CCA | 801 | E | G | -2.1 | Neutral |  |
| 1,45797721,G,A | CTG T[C/T]G GGC | 297 | S | L | -1.01 | Neutral |  |
|  | CTG T[C/T]G GGC | 296 | S | L | -1.01 | Neutral |  |
|  | CTG T[C/T]G GGC | 321 | S | L | -1.09 | Neutral |  |
|  | CTG T[C/T]G GGC | 307 | S | L | -1.01 | Neutral |  |
|  | CTG T[C/T]G GGC | 296 | S | L | -1.01 | Neutral |  |
|  | CTG T[C/T]G GGC | 311 | S | L | -1.01 | Neutral |  |
|  | CTG T[C/T]G GGC | 310 | S | L | -1.01 | Neutral |  |
|  | CTG T[C/T]G GGC | 296 | S | L | -1.01 | Neutral |  |
|  | CTG T[C/T]G GGC | 324 | S | L | -1.09 | Neutral |  |
|  | CTG T[C/T]G GGC | 307 | S | L | -1.01 | Neutral |  |
|  | CTG T[C/T]G GGC | 168 | S | L | -1.29 | Neutral |  |
|  | CTG T[C/T]G GGC | 51 | S | L | -0.19 | Neutral |  |
|  | CTG T[C/T]G GGC | 168 | S | L | -1.29 | Neutral |  |
|  | CTG T[C/T]G GGC | 94 | S | L | -1.5 | Neutral |  |
| 8,42207527,T,A | AAG AT[T/A] CGG | 88 | I | I | 0 | Neutral |  |
|  | AAG AT[T/A] CGG | 88 | I | I | 0 | Neutral |  |
|  | AAG AT[T/A] CGG | 19 | I | I | 0 | Neutral |  |
|  | AAG AT[T/A] CGG | 88 | I | I | 0 | Neutral |  |
| 8,42207525,A,C | AAG [A/C]TT CGG | 88 | I | L | -1.45 | Neutral |  |
|  | AAG [A/C]TT CGG | 88 | I | L | -1.28 | Neutral |  |
|  | AAG [A/C]TT CGG | 19 | I | L | -1.41 | Neutral |  |
|  | AAG [A/C]TT CGG | 88 | I | L | -1.23 | Neutral |  |
| 12,133256585,G,T | CAG GG[C/A] AAA | 126 | G | G | 0 | Neutral |  |
|  | CAG GG[C/A] AAA | 61 | G | G | 0 | Neutral |  |
|  | CAG GG[C/A] AAA | 137 | G | G | 0 | Neutral |  |
|  | CAG GG[C/A] AAA | 126 | G | G | 0 | Neutral |  |
|  | CAG GG[C/A] AAA | 99 | G | G | 0 | Neutral |  |
| 22,42032173,T,A | TTG GC[T/A] GTG | 84 | A | A | 0 | Neutral |  |
|  | TTG GC[T/A] GTG | 84 | A | A | 0 | Neutral |  |
|  | TTG GC[T/A] GTG | 34 | A | A | 0 | Neutral |  |
|  | TTG GC[T/A] GTG | 84 | A | A | 0 | Neutral |  |
|  | TTG GC[T/A] GTG | 84 | A | A | 0 | Neutral |  |
| 3,58512237,A,G | TCC TG[T/C] ACC | 434 | C | C | 0 | Neutral | [rs1127745](http://www.ncbi.nlm.nih.gov/projects/SNP/snp_ref.cgi?rs=rs1127745) |
|  | TCC TG[T/C] ACC | 420 | C | C | 0 | Neutral | [rs1127745](http://www.ncbi.nlm.nih.gov/projects/SNP/snp_ref.cgi?rs=rs1127745) |
| 8,42220154,G,A | GTG [G/A]AG CAG | 216 | E | K | -1.25 | Neutral |  |
|  | GTG [G/A]AG CAG | 32 | E | K | -1.31 | Neutral |  |
|  | GTG [G/A]AG CAG | 74 | E | K | -1.16 | Neutral |  |
|  | GTG [G/A]AG CAG | 251 | E | K | -1.11 | Neutral |  |
|  | GTG [G/A]AG CAG | 62 | E | K | -1.16 | Neutral |  |
| 7,44155782,C,A | TTC CC[G/T] CTG | 317 | P | P | 0 | Neutral |  |
|  | TTC CC[G/T] CTG | 317 | P | P | 0 | Neutral |  |
|  | TTC CC[G/T] CTG | 317 | P | P | 0 | Neutral |  |
|  | TTC CC[G/T] CTG | 36 | P | P | 0 | Neutral |  |
|  | TTC CC[G/T] CTG | 235 | P | P | 0 | Neutral |  |
| 13,32906729,A,C | GCA [A/C]AT CAG | 372 | N | H | -0.6 | Neutral | [rs144848](http://www.ncbi.nlm.nih.gov/projects/SNP/snp_ref.cgi?rs=rs144848) |
|  | GCA [A/C]AT CAG | 370 | N | H | -1.55 | Neutral | [rs144848](http://www.ncbi.nlm.nih.gov/projects/SNP/snp_ref.cgi?rs=rs144848) |
|  | GCA [A/C]AT CAG | 372 | N | H | -0.6 | Neutral | [rs144848](http://www.ncbi.nlm.nih.gov/projects/SNP/snp_ref.cgi?rs=rs144848) |
| 2,48028242,T,C | AAC TT[T/C] GAT | 1040 | F | F | 0 | Neutral |  |
|  | AAC TT[T/C] GAT | 738 | F | F | 0 | Neutral |  |
|  | AAC TT[T/C] GAT | 1038 | F | F | 0 | Neutral |  |
|  | AAC TT[T/C] GAT | 8 | F | F | 0 | Neutral |  |
|  | AAC TT[T/C] GAT | 910 | F | F | 0 | Neutral |  |
| 13,32936670,G,A | TGT [G/A]AC ACT | 2606 | D | N | -1.33 | Neutral |  |
|  | TGT [G/A]AC ACT | 2606 | D | N | -1.33 | Neutral |  |
| 22,29091781,C,A | TTG GC[G/T] CCT | 363 | A | A | 0 | Neutral |  |
|  | TTG GC[G/T] CCT | 392 | A | A | 0 | Neutral |  |
|  | TTG GC[G/T] CCT | 301 | A | A | 0 | Neutral |  |
|  | TTG GC[G/T] CCT | 435 | A | A | 0 | Neutral |  |
|  | TTG GC[G/T] CCT | 363 | A | A | 0 | Neutral |  |
|  | TTG GC[G/T] CCT | 301 | A | A | 0 | Neutral |  |
|  | TTG GC[G/T] CCT | 392 | A | A | 0 | Neutral |  |
|  | TTG GC[G/T] CCT | 392 | A | A | 0 | Neutral |  |
|  | TTG GC[G/T] CCT | 136 | A | A | 0 | Neutral |  |
|  | TTG GC[G/T] CCT | 171 | A | A | 0 | Neutral |  |
| 13,108862426,T,A | ATT CC[A/T] GGT | 397 | P | P | 0 | Neutral |  |
|  | ATT CC[A/T] GGT | 397 | P | P | 0 | Neutral |  |
|  | ATT CC[A/T] GGT | 397 | P | P | 0 | Neutral |  |
| 10,50669597,C,T | GGC [G/A]TG CAC | 1262 | V | M | -2.46 | Neutral | [rs115319252](http://www.ncbi.nlm.nih.gov/projects/SNP/snp_ref.cgi?rs=rs115319252) |
| 19,45855524,G,A | GTG GA[C/T] GAG | 661 | D | D | 0 | Neutral | [rs1052555](http://www.ncbi.nlm.nih.gov/projects/SNP/snp_ref.cgi?rs=rs1052555) |
|  | GTG GA[C/T] GAG | 687 | D | D | 0 | Neutral | [rs1052555](http://www.ncbi.nlm.nih.gov/projects/SNP/snp_ref.cgi?rs=rs1052555) |
|  | GTG GA[C/T] GAG | 633 | D | D | 0 | Neutral | [rs1052555](http://www.ncbi.nlm.nih.gov/projects/SNP/snp_ref.cgi?rs=rs1052555) |
|  | GTG GA[C/T] GAG | 711 | D | D | 0 | Neutral | [rs1052555](http://www.ncbi.nlm.nih.gov/projects/SNP/snp_ref.cgi?rs=rs1052555) |
| 2,216995680,C,T | TGC TT[C/T] TCT | 340 | F | F | 0 | Neutral | [rs61762971](http://www.ncbi.nlm.nih.gov/projects/SNP/snp_ref.cgi?rs=rs61762971) |
|  | TGC TT[C/T] TCT | 340 | F | F | 0 | Neutral | [rs61762971](http://www.ncbi.nlm.nih.gov/projects/SNP/snp_ref.cgi?rs=rs61762971) |
| 12,109535491,G,A | ATC [G/A]GC CAG | 3 | G | S | -1.47 | Neutral |  |
| 2,217059670,A,T | GAG GA[A/T] GCC | 690 | E | D | -2.1 | Neutral |  |
|  | GAG GA[A/T] GCC | 690 | E | D | -2.1 | Neutral |  |
| 2,217059671,G,T | GAA [G/T]CC TCT | 691 | A | S | -0.22 | Neutral |  |
|  | GAA [G/T]CC TCT | 691 | A | S | -0.22 | Neutral |  |
| 22,29091808,G,A | AGA AC[C/T] TTA | 354 | T | T | 0 | Neutral |  |
|  | AGA AC[C/T] TTA | 383 | T | T | 0 | Neutral |  |
|  | AGA AC[C/T] TTA | 292 | T | T | 0 | Neutral |  |
|  | AGA AC[C/T] TTA | 426 | T | T | 0 | Neutral |  |
|  | AGA AC[C/T] TTA | 354 | T | T | 0 | Neutral |  |
|  | AGA AC[C/T] TTA | 292 | T | T | 0 | Neutral |  |
|  | AGA AC[C/T] TTA | 383 | T | T | 0 | Neutral |  |
|  | AGA AC[C/T] TTA | 383 | T | T | 0 | Neutral |  |
|  | AGA AC[C/T] TTA | 127 | T | T | 0 | Neutral |  |
|  | AGA AC[C/T] TTA | 162 | T | T | 0 | Neutral |  |
| 4,178274806,C,A | AGT [C/A]CA GCA | 462 | P | T | -1.39 | Neutral |  |
| 17,7579472,G,C | CCC C[C/G]C GTG | 72 | P | R | -0.23 | Neutral | [rs1042522](http://www.ncbi.nlm.nih.gov/projects/SNP/snp_ref.cgi?rs=rs1042522) |
|  | CCC C[C/G]C GTG | 72 | P | R | -0.19 | Neutral | [rs1042522](http://www.ncbi.nlm.nih.gov/projects/SNP/snp_ref.cgi?rs=rs1042522) |
|  | CCC C[C/G]C GTG | 72 | P | R | -0.23 | Neutral | [rs1042522](http://www.ncbi.nlm.nih.gov/projects/SNP/snp_ref.cgi?rs=rs1042522) |
|  | CCC C[C/G]C GTG | 72 | P | R | -0.19 | Neutral | [rs1042522](http://www.ncbi.nlm.nih.gov/projects/SNP/snp_ref.cgi?rs=rs1042522) |
|  | CCC C[C/G]C GTG | 72 | P | R | -0.23 | Neutral | [rs1042522](http://www.ncbi.nlm.nih.gov/projects/SNP/snp_ref.cgi?rs=rs1042522) |
|  | CCC C[C/G]C GTG | 72 | P | R | -0.19 | Neutral | [rs1042522](http://www.ncbi.nlm.nih.gov/projects/SNP/snp_ref.cgi?rs=rs1042522) |
|  | CCC C[C/G]C GTG | 72 | P | R | -0.29 | Neutral | [rs1042522](http://www.ncbi.nlm.nih.gov/projects/SNP/snp_ref.cgi?rs=rs1042522) |
|  | CCC C[C/G]C GTG | 72 | P | R | -0.73 | Neutral | [rs1042522](http://www.ncbi.nlm.nih.gov/projects/SNP/snp_ref.cgi?rs=rs1042522) |
|  | CCC C[C/G]C GTG | 72 | P | R | -0.24 | Neutral | [rs1042522](http://www.ncbi.nlm.nih.gov/projects/SNP/snp_ref.cgi?rs=rs1042522) |
| 11,61093115,T,C | GAC [A/G]AA TAC | 244 | K | E | -0.18 | Neutral |  |
|  | GAC [A/G]AA TAC | 244 | K | E | -0.18 | Neutral |  |
|  | GAC [A/G]AA TAC | 59 | K | E | -0.31 | Neutral |  |
|  | GAC [A/G]AA TAC | 27 | K | E | -0.18 | Neutral |  |
| 11,108183187,A,T | TTG [A/T]GT GAA | 1990 | S | C | -2.38 | Neutral |  |
|  | TTG [A/T]GT GAA | 1990 | S | C | -2.38 | Neutral |  |
| 11,108163500,C,A | GAG [C/A]AG GTG | 1531 | Q | K | -1.17 | Neutral |  |
|  | GAG [C/A]AG GTG | 1531 | Q | K | -1.17 | Neutral |  |
| 11,108160469,A,G | GGA GG[A/G] GCT | 1459 | G | G | 0 | Neutral |  |
|  | GGA GG[A/G] GCT | 111 | G | G | 0 | Neutral |  |
|  | GGA GG[A/G] GCT | 1459 | G | G | 0 | Neutral |  |
|  | GGA GG[A/G] GCT | 129 | G | G | 0 | Neutral |  |
| x,66765391,C,A | GAG [C/A]CT GGA | 135 | P | T | -2.07 | Neutral |  |
|  | GAG [C/A]CT GGA | 135 | P | T | -2.48 | Neutral |  |
| 11,108160470,G,T | GGA [G/T]CT TGG | 1460 | A | S | -0.77 | Neutral |  |
|  | GGA [G/T]CT TGG | 112 | A | S | -1.05 | Neutral |  |
|  | GGA [G/T]CT TGG | 1460 | A | S | -0.77 | Neutral |  |
|  | GGA [G/T]CT TGG | 130 | A | S | -0.94 | Neutral |  |
| 22,42057388,A,G | TTT [A/G]AG GAG | 526 | K | E | -1.93 | Neutral |  |
|  | TTT [A/G]AG GAG | 526 | K | E | -1.93 | Neutral |  |
|  | TTT [A/G]AG GAG | 476 | K | E | -2.09 | Neutral |  |
|  | TTT [A/G]AG GAG | 526 | K | E | -1.89 | Neutral |  |
|  | TTT [A/G]AG GAG | 526 | K | E | -1.93 | Neutral |  |
|  | TTT [A/G]AG GAG | 485 | K | E | -2.13 | Neutral |  |
|  | TTT [A/G]AG GAG | 393 | K | E | -1.93 | Neutral |  |
| 19,48621036,C,G | AAG GC[G/C] CTG | 814 | A | A | 0 | Neutral | [rs13436](http://www.ncbi.nlm.nih.gov/projects/SNP/snp_ref.cgi?rs=rs13436) |
|  | AAG GC[G/C] CTG | 746 | A | A | 0 | Neutral | [rs13436](http://www.ncbi.nlm.nih.gov/projects/SNP/snp_ref.cgi?rs=rs13436) |
|  | AAG GC[G/C] CTG | 783 | A | A | 0 | Neutral | [rs13436](http://www.ncbi.nlm.nih.gov/projects/SNP/snp_ref.cgi?rs=rs13436) |
| 20,10621882,G,A | ACT A[C/T]G GAG | 976 | T | M | -0.2 | Neutral |  |
|  | ACT A[C/T]G GAG | 817 | T | M | -0.24 | Neutral |  |
| 18,20602225,T,A | TGT A[T/A]G GAA | 863 | M | K | -1.32 | Neutral |  |
|  | TGT A[T/A]G GAA | 868 | M | K | -1.32 | Neutral |  |
|  | GTA [T/A]GG AAA | 831 | W | R | 0.9 | Neutral |  |
|  | TGT A[T/A]G GAA | 863 | M | K | -1.32 | Neutral |  |
|  | GTA [T/A]GG AAA | 831 | W | R | 1.46 | Neutral |  |
| 2,217059681,G,A | GGA A[G/A]T TCT | 694 | S | N | -2.29 | Neutral |  |
|  | GGA A[G/A]T TCT | 694 | S | N | -2.29 | Neutral |  |
| 19,44056955,G,A | AGA [C/T]CT AAA | 273 | P | S | -1.14 | Neutral |  |
|  | AGA [C/T]CT AAA | 287 | P | S | -1.14 | Neutral |  |
|  | AGA [C/T]CT AAA | 242 | P | S | -1.2 | Neutral |  |
|  | AGA [C/T]CT AAA | 273 | P | S | -1.11 | Neutral |  |
| 19,45868309,T,G | TGC CG[A/C] TTC | 132 | R | R | 0 | Neutral | [rs238406](http://www.ncbi.nlm.nih.gov/projects/SNP/snp_ref.cgi?rs=rs238406) |
|  | TGC CG[A/C] TTC | 106 | R | R | 0 | Neutral | [rs238406](http://www.ncbi.nlm.nih.gov/projects/SNP/snp_ref.cgi?rs=rs238406) |
|  | TGC CG[A/C] TTC | 132 | R | R | 0 | Neutral | [rs238406](http://www.ncbi.nlm.nih.gov/projects/SNP/snp_ref.cgi?rs=rs238406) |
|  | TGC CG[A/C] TTC | 156 | R | R | 0 | Neutral | [rs238406](http://www.ncbi.nlm.nih.gov/projects/SNP/snp_ref.cgi?rs=rs238406) |
|  | TGC CG[A/C] TTC | 132 | R | R | 0 | Neutral | [rs238406](http://www.ncbi.nlm.nih.gov/projects/SNP/snp_ref.cgi?rs=rs238406) |
| 5,68572496,A,G | AAA [A/G]GA ACA | 331 | R | G | -1.85 | Neutral |  |
|  | AAA [A/G]GA ACA | 238 | R | G | -1.59 | Neutral |  |
|  | AAA [A/G]GA ACA | 294 | R | G | -1.81 | Neutral |  |
| 2,48010468,C,A | GGC GG[C/A] CGT | 32 | G | G | 0 | Neutral |  |
|  | GGC GG[C/A] CGT | 32 | G | G | 0 | Neutral |  |
|  | GGC GG[C/A] CGT | 32 | G | G | 0 | Neutral |  |
|  | GGC GG[C/A] CGT | 32 | G | G | 0 | Neutral |  |
|  | GGC GG[C/A] CGT | 32 | G | G | 0 | Neutral |  |
|  | GGC GG[C/A] CGT | 32 | G | G | 0 | Neutral |  |
| 16,14041958,T,C | GAT TC[T/C] GAA | 835 | S | S | 0 | Neutral | [rs1799801](http://www.ncbi.nlm.nih.gov/projects/SNP/snp_ref.cgi?rs=rs1799801) |
|  | GAT TC[T/C] GAA | 823 | S | S | 0 | Neutral | [rs1799801](http://www.ncbi.nlm.nih.gov/projects/SNP/snp_ref.cgi?rs=rs1799801) |
| 4,178262713,T,A | ATA AC[T/A] GTG | 262 | T | T | 0 | Neutral |  |
| 7,73651704,G,A | ATT GA[C/T] GAA | 276 | D | D | 0 | Neutral | [rs148392548](http://www.ncbi.nlm.nih.gov/projects/SNP/snp_ref.cgi?rs=rs148392548) |
|  | ATT GA[C/T] GAA | 242 | D | D | 0 | Neutral | [rs148392548](http://www.ncbi.nlm.nih.gov/projects/SNP/snp_ref.cgi?rs=rs148392548) |
|  | ATT GA[C/T] GAA | 69 | D | D | 0 | Neutral | [rs148392548](http://www.ncbi.nlm.nih.gov/projects/SNP/snp_ref.cgi?rs=rs148392548) |
|  | ATT GA[C/T] GAA | 132 | D | D | 0 | Neutral | [rs148392548](http://www.ncbi.nlm.nih.gov/projects/SNP/snp_ref.cgi?rs=rs148392548) |
| 8,90967711,A,G | CAA GA[T/C] GCA | 399 | D | D | 0 | Neutral | [rs709816](http://www.ncbi.nlm.nih.gov/projects/SNP/snp_ref.cgi?rs=rs709816) |
|  | CAA GA[T/C] GCA | 317 | D | D | 0 | Neutral | [rs709816](http://www.ncbi.nlm.nih.gov/projects/SNP/snp_ref.cgi?rs=rs709816) |
|  | CAA GA[T/C] GCA | 399 | D | D | 0 | Neutral | [rs709816](http://www.ncbi.nlm.nih.gov/projects/SNP/snp_ref.cgi?rs=rs709816) |
| 16,14038656,G,T | ACA [G/T]CA TCT | 661 | A | S | 0.5 | Neutral |  |
|  | ACA [G/T]CA TCT | 649 | A | S | 0.5 | Neutral |  |
| 13,108863609,G,A | GCT G[C/T]C TCA | 3 | A | V | -0.54 | Neutral | [rs1805389](http://www.ncbi.nlm.nih.gov/projects/SNP/snp_ref.cgi?rs=rs1805389) |
|  | GCT G[C/T]C TCA | 3 | A | V | -0.54 | Neutral | [rs1805389](http://www.ncbi.nlm.nih.gov/projects/SNP/snp_ref.cgi?rs=rs1805389) |
|  | GCT G[C/T]C TCA | 3 | A | V | -0.54 | Neutral | [rs1805389](http://www.ncbi.nlm.nih.gov/projects/SNP/snp_ref.cgi?rs=rs1805389) |
| 11,108117799,G,A | TTT C[G/A]T AAT | 337 | R | H | -2.43 | Neutral |  |
|  | TTT C[G/A]T AAT | 337 | R | H | -2.43 | Neutral |  |
| 8,42220154,G,A | GTG [G/A]AG CAG | 216 | E | K | -1.25 | Neutral |  |
|  | GTG [G/A]AG CAG | 32 | E | K | -1.31 | Neutral |  |
|  | GTG [G/A]AG CAG | 74 | E | K | -1.16 | Neutral |  |
|  | GTG [G/A]AG CAG | 251 | E | K | -1.11 | Neutral |  |
|  | GTG [G/A]AG CAG | 62 | E | K | -1.16 | Neutral |  |
| 5,60200665,A,G | GTT TA[T/C] AGT | 145 | Y | Y | 0 | Neutral | [rs4647100](http://www.ncbi.nlm.nih.gov/projects/SNP/snp_ref.cgi?rs=rs4647100) |
|  | GTT TA[T/C] AGT | 87 | Y | Y | 0 | Neutral | [rs4647100](http://www.ncbi.nlm.nih.gov/projects/SNP/snp_ref.cgi?rs=rs4647100) |
|  | GTT TA[T/C] AGT | 87 | Y | Y | 0 | Neutral | [rs4647100](http://www.ncbi.nlm.nih.gov/projects/SNP/snp_ref.cgi?rs=rs4647100) |
|  | GTT TA[T/C] AGT | 144 | Y | Y | 0 | Neutral | [rs4647100](http://www.ncbi.nlm.nih.gov/projects/SNP/snp_ref.cgi?rs=rs4647100) |
| 1,3624171,C,T | TCG G[C/T]C AGC | 82 | A | V | -1.12 | Neutral |  |
|  | TCG G[C/T]C AGC | 82 | A | V | -1.12 | Neutral |  |
|  | TCG G[C/T]C AGC | 82 | A | V | -1.12 | Neutral |  |
|  | TCG G[C/T]C AGC | 33 | A | V | -1.03 | Neutral |  |
|  | TCG G[C/T]C AGC | 33 | A | V | -0.94 | Neutral |  |
|  | TCG G[C/T]C AGC | 33 | A | V | -1.04 | Neutral |  |
|  | TCG G[C/T]C AGC | 11 | A | V | -0.75 | Neutral |  |
|  | TCG G[C/T]C AGC | 82 | A | V | -1.29 | Neutral |  |
| 21,42866332,G,A | AAC CC[C/T] GTC | 63 | P | P | 0 | Neutral | [rs61735792](http://www.ncbi.nlm.nih.gov/projects/SNP/snp_ref.cgi?rs=rs61735792) |
|  | AAC CC[C/T] GTC | 100 | P | P | 0 | Neutral | [rs61735792](http://www.ncbi.nlm.nih.gov/projects/SNP/snp_ref.cgi?rs=rs61735792) |
|  | AAC CC[C/T] GTC | 63 | P | P | 0 | Neutral | [rs61735792](http://www.ncbi.nlm.nih.gov/projects/SNP/snp_ref.cgi?rs=rs61735792) |
|  | AAC CC[C/T] GTC | 63 | P | P | 0 | Neutral | [rs61735792](http://www.ncbi.nlm.nih.gov/projects/SNP/snp_ref.cgi?rs=rs61735792) |
|  | AAC CC[C/T] GTC | 63 | P | P | 0 | Neutral | [rs61735792](http://www.ncbi.nlm.nih.gov/projects/SNP/snp_ref.cgi?rs=rs61735792) |
|  | AAC CC[C/T] GTC | 63 | P | P | 0 | Neutral | [rs61735792](http://www.ncbi.nlm.nih.gov/projects/SNP/snp_ref.cgi?rs=rs61735792) |
| 3,142272757,T,C | ATG GA[A/G] GAT | 814 | E | E | 0 | Neutral | [rs55895932](http://www.ncbi.nlm.nih.gov/projects/SNP/snp_ref.cgi?rs=rs55895932) |
|  | ATG GA[A/G] GAT | 750 | E | E | 0 | Neutral | [rs55895932](http://www.ncbi.nlm.nih.gov/projects/SNP/snp_ref.cgi?rs=rs55895932) |
| 4,178281712,C,T | CCT [C/T]GC TGC | 506 | R | C | -1.92 | Neutral | [rs144742737](http://www.ncbi.nlm.nih.gov/projects/SNP/snp_ref.cgi?rs=rs144742737) |
| 11,18369422,G,A | GAT [G/A]GA AAT | 337 | G | R | -1.68 | Neutral |  |
|  | GAT [G/A]GA AAT | 337 | G | R | -1.68 | Neutral |  |
|  | GAT [G/A]GA AAT | 25 | G | R | -1.96 | Neutral |  |
|  | GAT [G/A]GA AAT | 221 | G | R | -1.67 | Neutral |  |
|  | GAT [G/A]GA AAT | 133 | G | R | -1.47 | Neutral |  |
| 19,48619142,G,T | GCC AC[C/A] ACC | 888 | T | T | 0 | Neutral |  |
|  | GCC AC[C/A] ACC | 820 | T | T | 0 | Neutral |  |
|  | GCC AC[C/A] ACC | 857 | T | T | 0 | Neutral |  |
|  | TTT TC[C/T] GGA | 96 | S | S | 0 | Neutral |  |
| 19,50370425,C,G | CTC [G/C]AG AGC | 13 | E | Q | 0.13 | Neutral |  |
| 13,32912299,T,C | TCT GT[T/C] GTT | 1269 | V | V | 0 | Neutral | [rs543304](http://www.ncbi.nlm.nih.gov/projects/SNP/snp_ref.cgi?rs=rs543304) |
|  | TCT GT[T/C] GTT | 1269 | V | V | 0 | Neutral | [rs543304](http://www.ncbi.nlm.nih.gov/projects/SNP/snp_ref.cgi?rs=rs543304) |
| 1,242045275,C,T | CTA [C/T]GT TTA | 723 | R | C | -1.22 | Neutral | [rs1635498](http://www.ncbi.nlm.nih.gov/projects/SNP/snp_ref.cgi?rs=rs1635498) |
|  | CTA [C/T]GT TTA | 723 | R | C | -1.22 | Neutral | [rs1635498](http://www.ncbi.nlm.nih.gov/projects/SNP/snp_ref.cgi?rs=rs1635498) |
|  | CTA [C/T]GT TTA | 723 | R | C | -1.22 | Neutral | [rs1635498](http://www.ncbi.nlm.nih.gov/projects/SNP/snp_ref.cgi?rs=rs1635498) |
| 21,42845374,G,A | GGC GG[C/T] GAG | 259 | G | G | 0 | Neutral | [rs2298659](http://www.ncbi.nlm.nih.gov/projects/SNP/snp_ref.cgi?rs=rs2298659) |
|  | GGC GG[C/T] GAG | 296 | G | G | 0 | Neutral | [rs2298659](http://www.ncbi.nlm.nih.gov/projects/SNP/snp_ref.cgi?rs=rs2298659) |
|  | GGC GG[C/T] GAG | 259 | G | G | 0 | Neutral | [rs2298659](http://www.ncbi.nlm.nih.gov/projects/SNP/snp_ref.cgi?rs=rs2298659) |
|  | GGC GG[C/T] GAG | 259 | G | G | 0 | Neutral | [rs2298659](http://www.ncbi.nlm.nih.gov/projects/SNP/snp_ref.cgi?rs=rs2298659) |
|  | GGC GG[C/T] | 219 | G | G | 0 | Neutral | [rs2298659](http://www.ncbi.nlm.nih.gov/projects/SNP/snp_ref.cgi?rs=rs2298659) |
| 7,6013173,C,T | TCG [G/A]TG ATG | 816 | V | M | -2.27 | Neutral |  |
|  | TCG [G/A]TG ATG | 415 | V | M | -2.22 | Neutral |  |
|  | TCG [G/A]TG ATG | 769 | V | M | -2.27 | Neutral |  |
|  | TCG [G/A]TG ATG | 710 | V | M | -2.17 | Neutral |  |
| 19,50902310,G,A | GAC [G/A]GG CAG | 69 | G | R | -1.12 | Neutral |  |
|  | GAC [G/A]GG CAG | 68 | G | R | -1.55 | Neutral |  |
| 7,6013153,A,G | GCT CT[T/C] AAC | 822 | L | L | 0 | Neutral | [rs10000](http://www.ncbi.nlm.nih.gov/projects/SNP/snp_ref.cgi?rs=rs10000) |
|  | GCT CT[T/C] AAC | 421 | L | L | 0 | Neutral | [rs10000](http://www.ncbi.nlm.nih.gov/projects/SNP/snp_ref.cgi?rs=rs10000) |
|  | GCT CT[T/C] AAC | 775 | L | L | 0 | Neutral | [rs10000](http://www.ncbi.nlm.nih.gov/projects/SNP/snp_ref.cgi?rs=rs10000) |
|  | GCT CT[T/C] AAC | 716 | L | L | 0 | Neutral | [rs10000](http://www.ncbi.nlm.nih.gov/projects/SNP/snp_ref.cgi?rs=rs10000) |
| 2,217006039,T,C | TTA TT[T/C] CAG | 491 | F | F | 0 | Neutral | [rs41257924](http://www.ncbi.nlm.nih.gov/projects/SNP/snp_ref.cgi?rs=rs41257924) |
|  | TTA TT[T/C] CAG | 491 | F | F | 0 | Neutral | [rs41257924](http://www.ncbi.nlm.nih.gov/projects/SNP/snp_ref.cgi?rs=rs41257924) |
| 13,32906766,C,T | ATC T[C/T]C AAG | 384 | S | F | -1.15 | Neutral | [rs41293475](http://www.ncbi.nlm.nih.gov/projects/SNP/snp_ref.cgi?rs=rs41293475) |
|  | ATC T[C/T]C AAG | 384 | S | F | -1.15 | Neutral | [rs41293475](http://www.ncbi.nlm.nih.gov/projects/SNP/snp_ref.cgi?rs=rs41293475) |
| 13,32929232,A,G | AAA TC[A/G] CAT | 2414 | S | S | 0 | Neutral | [rs1799955](http://www.ncbi.nlm.nih.gov/projects/SNP/snp_ref.cgi?rs=rs1799955) |
|  | AAA TC[A/G] CAT | 2414 | S | S | 0 | Neutral | [rs1799955](http://www.ncbi.nlm.nih.gov/projects/SNP/snp_ref.cgi?rs=rs1799955) |
| 19,30314666,C,T | AGC AG[C/T] GGG | 405 | S | S | 0 | Neutral | [rs7257694](http://www.ncbi.nlm.nih.gov/projects/SNP/snp_ref.cgi?rs=rs7257694) |
|  | AGC AG[C/T] GGG | 362 | S | S | 0 | Neutral | [rs7257694](http://www.ncbi.nlm.nih.gov/projects/SNP/snp_ref.cgi?rs=rs7257694) |
|  | AGC AG[C/T] GGG | 390 | S | S | 0 | Neutral | [rs7257694](http://www.ncbi.nlm.nih.gov/projects/SNP/snp_ref.cgi?rs=rs7257694) |
| 12,124144395,A,G | CCC CC[A/G] GTT | 205 | P | P | 0 | Neutral | [rs1051793](http://www.ncbi.nlm.nih.gov/projects/SNP/snp_ref.cgi?rs=rs1051793) |
|  | CCC CC[A/G] GTT | 132 | P | P | 0 | Neutral | [rs1051793](http://www.ncbi.nlm.nih.gov/projects/SNP/snp_ref.cgi?rs=rs1051793) |
|  | CCC [A/G]GT TCA | 198 | S | G | -0.87 | Neutral | [rs1051793](http://www.ncbi.nlm.nih.gov/projects/SNP/snp_ref.cgi?rs=rs1051793) |
|  | CCC CC[A/G] GTT | 196 | P | P | 0 | Neutral | [rs1051793](http://www.ncbi.nlm.nih.gov/projects/SNP/snp_ref.cgi?rs=rs1051793) |
|  | CCC CC[A/G] GTT | 203 | P | P | 0 | Neutral | [rs1051793](http://www.ncbi.nlm.nih.gov/projects/SNP/snp_ref.cgi?rs=rs1051793) |
|  | CCC CC[A/G] GTT | 246 | P | P | 0 | Neutral | [rs1051793](http://www.ncbi.nlm.nih.gov/projects/SNP/snp_ref.cgi?rs=rs1051793) |
| 13,108861913,A,G | AGT GA[T/C] ATG | 568 | D | D | 0 | Neutral | [rs1805386](http://www.ncbi.nlm.nih.gov/projects/SNP/snp_ref.cgi?rs=rs1805386) |
|  | AGT GA[T/C] ATG | 568 | D | D | 0 | Neutral | [rs1805386](http://www.ncbi.nlm.nih.gov/projects/SNP/snp_ref.cgi?rs=rs1805386) |
|  | AGT GA[T/C] ATG | 568 | D | D | 0 | Neutral | [rs1805386](http://www.ncbi.nlm.nih.gov/projects/SNP/snp_ref.cgi?rs=rs1805386) |
| 3,37053568,A,G | TCC [A/G]TC TTT | 219 | I | V | -0.46 | Neutral | [rs1799977](http://www.ncbi.nlm.nih.gov/projects/SNP/snp_ref.cgi?rs=rs1799977) |
|  | TCC [A/G]TC TTT | 83 | I | V | -0.36 | Neutral | [rs1799977](http://www.ncbi.nlm.nih.gov/projects/SNP/snp_ref.cgi?rs=rs1799977) |
|  | TCC [A/G]TC TTT | 13 | I | V | -0.3 | Neutral | [rs1799977](http://www.ncbi.nlm.nih.gov/projects/SNP/snp_ref.cgi?rs=rs1799977) |
|  | TCC [A/G]TC TTT | 121 | I | V | -0.43 | Neutral | [rs1799977](http://www.ncbi.nlm.nih.gov/projects/SNP/snp_ref.cgi?rs=rs1799977) |
|  | TCC [A/G]TC TTT | 185 | I | V | -0.4 | Neutral | [rs1799977](http://www.ncbi.nlm.nih.gov/projects/SNP/snp_ref.cgi?rs=rs1799977) |
|  | TCC [A/G]TC TTT | 211 | I | V | -0.39 | Neutral | [rs1799977](http://www.ncbi.nlm.nih.gov/projects/SNP/snp_ref.cgi?rs=rs1799977) |
|  | TCC [A/G]TC TTT | 185 | I | V | -0.46 | Neutral | [rs1799977](http://www.ncbi.nlm.nih.gov/projects/SNP/snp_ref.cgi?rs=rs1799977) |
| 1,226555302,A,G | AAG G[T/C]G GAA | 762 | V | A | -1.33 | Neutral | [rs1136410](http://www.ncbi.nlm.nih.gov/projects/SNP/snp_ref.cgi?rs=rs1136410) |
| 7,6043658,A,G | GAT CT[T/C] ATT | 65 | L | L | 0 | Neutral |  |
|  | GAT CT[T/C] ATT | 65 | L | L | 0 | Neutral |  |
|  | GAT CT[T/C] ATT | 18 | L | L | 0 | Neutral |  |
|  | GAT CT[T/C] ATT | 65 | L | L | 0 | Neutral |  |
| 12,133219831,T,C | AGG GC[A/G] TCC | 1510 | A | A | 0 | Neutral | [rs5744944](http://www.ncbi.nlm.nih.gov/projects/SNP/snp_ref.cgi?rs=rs5744944) |
|  | AGG GC[A/G] TCC | 1521 | A | A | 0 | Neutral | [rs5744944](http://www.ncbi.nlm.nih.gov/projects/SNP/snp_ref.cgi?rs=rs5744944) |
|  | AGG GC[A/G] TCC | 1483 | A | A | 0 | Neutral | [rs5744944](http://www.ncbi.nlm.nih.gov/projects/SNP/snp_ref.cgi?rs=rs5744944) |
| 3,121208833,G,C | CAG A[C/G]A TGT | 982 | T | R | -0.34 | Neutral | [rs3218649](http://www.ncbi.nlm.nih.gov/projects/SNP/snp_ref.cgi?rs=rs3218649) |
|  | CAG A[C/G]A TGT | 1118 | T | R | -0.34 | Neutral | [rs3218649](http://www.ncbi.nlm.nih.gov/projects/SNP/snp_ref.cgi?rs=rs3218649) |
|  | CAG A[C/G]A TGT | 605 | T | R | -0.28 | Neutral | [rs3218649](http://www.ncbi.nlm.nih.gov/projects/SNP/snp_ref.cgi?rs=rs3218649) |
| 17,1733399,A,G | GGC CA[A/G] CTG | 4 | Q | Q | 0 | Neutral | [rs5030749](http://www.ncbi.nlm.nih.gov/projects/SNP/snp_ref.cgi?rs=rs5030749) |
| 21,42845374,G,A | GGC GG[C/T] GAG | 259 | G | G | 0 | Neutral | [rs2298659](http://www.ncbi.nlm.nih.gov/projects/SNP/snp_ref.cgi?rs=rs2298659) |
|  | GGC GG[C/T] GAG | 296 | G | G | 0 | Neutral | [rs2298659](http://www.ncbi.nlm.nih.gov/projects/SNP/snp_ref.cgi?rs=rs2298659) |
|  | GGC GG[C/T] GAG | 259 | G | G | 0 | Neutral | [rs2298659](http://www.ncbi.nlm.nih.gov/projects/SNP/snp_ref.cgi?rs=rs2298659) |
|  | GGC GG[C/T] GAG | 259 | G | G | 0 | Neutral | [rs2298659](http://www.ncbi.nlm.nih.gov/projects/SNP/snp_ref.cgi?rs=rs2298659) |
|  | GGC GG[C/T] | 219 | G | G | 0 | Neutral | [rs2298659](http://www.ncbi.nlm.nih.gov/projects/SNP/snp_ref.cgi?rs=rs2298659) |
| 21,42845383,A,G | AGG AT[T/C] GTG | 256 | I | I | 0 | Neutral | [rs17854725](http://www.ncbi.nlm.nih.gov/projects/SNP/snp_ref.cgi?rs=rs17854725) |
|  | AGG AT[T/C] GTG | 293 | I | I | 0 | Neutral | [rs17854725](http://www.ncbi.nlm.nih.gov/projects/SNP/snp_ref.cgi?rs=rs17854725) |
|  | AGG AT[T/C] GTG | 256 | I | I | 0 | Neutral | [rs17854725](http://www.ncbi.nlm.nih.gov/projects/SNP/snp_ref.cgi?rs=rs17854725) |
|  | AGG AT[T/C] GTG | 256 | I | I | 0 | Neutral | [rs17854725](http://www.ncbi.nlm.nih.gov/projects/SNP/snp_ref.cgi?rs=rs17854725) |
|  | AGG AT[T/C] GTG | 216 | I | I | 0 | Neutral | [rs17854725](http://www.ncbi.nlm.nih.gov/projects/SNP/snp_ref.cgi?rs=rs17854725) |
| 17,7579472,G,C | CCC C[C/G]C GTG | 72 | P | R | -0.23 | Neutral | [rs1042522](http://www.ncbi.nlm.nih.gov/projects/SNP/snp_ref.cgi?rs=rs1042522) |
|  | CCC C[C/G]C GTG | 72 | P | R | -0.19 | Neutral | [rs1042522](http://www.ncbi.nlm.nih.gov/projects/SNP/snp_ref.cgi?rs=rs1042522) |
|  | CCC C[C/G]C GTG | 72 | P | R | -0.23 | Neutral | [rs1042522](http://www.ncbi.nlm.nih.gov/projects/SNP/snp_ref.cgi?rs=rs1042522) |
|  | CCC C[C/G]C GTG | 72 | P | R | -0.19 | Neutral | [rs1042522](http://www.ncbi.nlm.nih.gov/projects/SNP/snp_ref.cgi?rs=rs1042522) |
|  | CCC C[C/G]C GTG | 72 | P | R | -0.23 | Neutral | [rs1042522](http://www.ncbi.nlm.nih.gov/projects/SNP/snp_ref.cgi?rs=rs1042522) |
|  | CCC C[C/G]C GTG | 72 | P | R | -0.19 | Neutral | [rs1042522](http://www.ncbi.nlm.nih.gov/projects/SNP/snp_ref.cgi?rs=rs1042522) |
|  | CCC C[C/G]C GTG | 72 | P | R | -0.29 | Neutral | [rs1042522](http://www.ncbi.nlm.nih.gov/projects/SNP/snp_ref.cgi?rs=rs1042522) |
|  | CCC C[C/G]C GTG | 72 | P | R | -0.73 | Neutral | [rs1042522](http://www.ncbi.nlm.nih.gov/projects/SNP/snp_ref.cgi?rs=rs1042522) |
|  | CCC C[C/G]C GTG | 72 | P | R | -0.24 | Neutral | [rs1042522](http://www.ncbi.nlm.nih.gov/projects/SNP/snp_ref.cgi?rs=rs1042522) |
| 22,42032173,T,A | TTG GC[T/A] GTG | 84 | A | A | 0 | Neutral |  |
|  | TTG GC[T/A] GTG | 84 | A | A | 0 | Neutral |  |
|  | TTG GC[T/A] GTG | 34 | A | A | 0 | Neutral |  |
|  | TTG GC[T/A] GTG | 84 | A | A | 0 | Neutral |  |
|  | TTG GC[T/A] GTG | 84 | A | A | 0 | Neutral |  |
| 9,133761001,A,G | GGT CC[A/G] GCG | 1108 | P | P | 0 | Neutral | [rs1056171](http://www.ncbi.nlm.nih.gov/projects/SNP/snp_ref.cgi?rs=rs1056171) |
|  | GGT CC[A/G] GCG | 1127 | P | P | 0 | Neutral | [rs1056171](http://www.ncbi.nlm.nih.gov/projects/SNP/snp_ref.cgi?rs=rs1056171) |
|  | GGT CC[A/G] GCG | 923 | P | P | 0 | Neutral | [rs1056171](http://www.ncbi.nlm.nih.gov/projects/SNP/snp_ref.cgi?rs=rs1056171) |
| 1,23847464,C,A | GAC CA[G/T] CTC | 226 | Q | H | -1.61 | Neutral | [rs2075995](http://www.ncbi.nlm.nih.gov/projects/SNP/snp_ref.cgi?rs=rs2075995) |
| 14,75483812,T,C | CAG CA[A/G] TCC | 1421 | Q | Q | 0 | Neutral | [rs13712](http://www.ncbi.nlm.nih.gov/projects/SNP/snp_ref.cgi?rs=rs13712) |
|  | CAG CA[A/G] TCC | 1445 | Q | Q | 0 | Neutral | [rs13712](http://www.ncbi.nlm.nih.gov/projects/SNP/snp_ref.cgi?rs=rs13712) |
|  | CAG CA[A/G] TCC | 383 | Q | Q | 0 | Neutral | [rs13712](http://www.ncbi.nlm.nih.gov/projects/SNP/snp_ref.cgi?rs=rs13712) |
|  | CAG CA[A/G] TCC | 116 | Q | Q | 0 | Neutral | [rs13712](http://www.ncbi.nlm.nih.gov/projects/SNP/snp_ref.cgi?rs=rs13712) |
|  | CAG CA[A/G] TCC | 469 | Q | Q | 0 | Neutral | [rs13712](http://www.ncbi.nlm.nih.gov/projects/SNP/snp_ref.cgi?rs=rs13712) |
|  | CAG CA[A/G] TCC | 1267 | Q | Q | 0 | Neutral | [rs13712](http://www.ncbi.nlm.nih.gov/projects/SNP/snp_ref.cgi?rs=rs13712) |
|  | CAG CA[A/G] TCC | 1445 | Q | Q | 0 | Neutral | [rs13712](http://www.ncbi.nlm.nih.gov/projects/SNP/snp_ref.cgi?rs=rs13712) |
| 5,80168937,G,A | GGC [G/A]CA GCA | 1045 | A | T | -0.1 | Neutral | [rs26279](http://www.ncbi.nlm.nih.gov/projects/SNP/snp_ref.cgi?rs=rs26279) |
|  | GGC [G/A]CA GCA | 1036 | A | T | -0.1 | Neutral | [rs26279](http://www.ncbi.nlm.nih.gov/projects/SNP/snp_ref.cgi?rs=rs26279) |
| 4,178274694,T,G | AGT GT[T/G] TGT | 424 | V | V | 0 | Neutral | [rs10007075](http://www.ncbi.nlm.nih.gov/projects/SNP/snp_ref.cgi?rs=rs10007075) |
| 1,3638674,C,T | GGC AC[C/T] GCC | 173 | T | T | 0 | Neutral | [rs1801174](http://www.ncbi.nlm.nih.gov/projects/SNP/snp_ref.cgi?rs=rs1801174) |
|  | GGC AC[C/T] GCC | 173 | T | T | 0 | Neutral | [rs1801174](http://www.ncbi.nlm.nih.gov/projects/SNP/snp_ref.cgi?rs=rs1801174) |
|  | GGC AC[C/T] GCC | 173 | T | T | 0 | Neutral | [rs1801174](http://www.ncbi.nlm.nih.gov/projects/SNP/snp_ref.cgi?rs=rs1801174) |
|  | GGC AC[C/T] GCC | 124 | T | T | 0 | Neutral | [rs1801174](http://www.ncbi.nlm.nih.gov/projects/SNP/snp_ref.cgi?rs=rs1801174) |
|  | GGC AC[C/T] GCC | 124 | T | T | 0 | Neutral | [rs1801174](http://www.ncbi.nlm.nih.gov/projects/SNP/snp_ref.cgi?rs=rs1801174) |
|  | GGC AC[C/T] GCC | 124 | T | T | 0 | Neutral | [rs1801174](http://www.ncbi.nlm.nih.gov/projects/SNP/snp_ref.cgi?rs=rs1801174) |
|  | GGC AC[C/T] GCC | 102 | T | T | 0 | Neutral | [rs1801174](http://www.ncbi.nlm.nih.gov/projects/SNP/snp_ref.cgi?rs=rs1801174) |
|  | GGC AC[C/T] GCC | 173 | T | T | 0 | Neutral | [rs1801174](http://www.ncbi.nlm.nih.gov/projects/SNP/snp_ref.cgi?rs=rs1801174) |
| 11,108175462,G,A | CAA [G/A]AT ACA | 1853 | D | N | -1.31 | Neutral | [rs1801516](http://www.ncbi.nlm.nih.gov/projects/SNP/snp_ref.cgi?rs=rs1801516) |
|  | CAA [G/A]AT ACA | 1853 | D | N | -1.31 | Neutral | [rs1801516](http://www.ncbi.nlm.nih.gov/projects/SNP/snp_ref.cgi?rs=rs1801516) |
| 3,142266595,G,T | GGC C[C/A]G AGA | 1110 | P | Q | -1.98 | Neutral |  |
|  | GGC C[C/A]G AGA | 1046 | P | Q | -1.98 | Neutral |  |
| 15,91312789,C,A | CTG A[C/A]T CAG | 843 | T | N | 1.5 | Neutral |  |
|  | CTG A[C/A]T CAG | 30 | T | N | 1.15 | Neutral |  |
|  | CTG A[C/A]T CAG | 496 | T | N | 1.33 | Neutral |  |
|  | CTG A[C/A]T CAG | 843 | T | N | 1.16 | Neutral |  |
| 17,41245237,A,G | TCA [T/C]TG GTA | 771 | L | L | 0 | Neutral | [rs16940](http://www.ncbi.nlm.nih.gov/projects/SNP/snp_ref.cgi?rs=rs16940) |
|  | TCA [T/C]TG GTA | 475 | L | L | 0 | Neutral | [rs16940](http://www.ncbi.nlm.nih.gov/projects/SNP/snp_ref.cgi?rs=rs16940) |
|  | TCA [T/C]TG GTA | 771 | L | L | 0 | Neutral | [rs16940](http://www.ncbi.nlm.nih.gov/projects/SNP/snp_ref.cgi?rs=rs16940) |
|  | TCA [T/C]TG GTA | 771 | L | L | 0 | Neutral | [rs16940](http://www.ncbi.nlm.nih.gov/projects/SNP/snp_ref.cgi?rs=rs16940) |
|  | TCA [T/C]TG GTA | 771 | L | L | 0 | Neutral | [rs16940](http://www.ncbi.nlm.nih.gov/projects/SNP/snp_ref.cgi?rs=rs16940) |
|  | TCA [T/C]TG GTA | 724 | L | L | 0 | Neutral | [rs16940](http://www.ncbi.nlm.nih.gov/projects/SNP/snp_ref.cgi?rs=rs16940) |
|  | TCA [T/C]TG GTA | 771 | L | L | 0 | Neutral | [rs16940](http://www.ncbi.nlm.nih.gov/projects/SNP/snp_ref.cgi?rs=rs16940) |
| 17,41245466,G,A | GAC AG[C/T] GAT | 694 | S | S | 0 | Neutral | [rs1799949](http://www.ncbi.nlm.nih.gov/projects/SNP/snp_ref.cgi?rs=rs1799949) |
|  | GAC AG[C/T] GAT | 398 | S | S | 0 | Neutral | [rs1799949](http://www.ncbi.nlm.nih.gov/projects/SNP/snp_ref.cgi?rs=rs1799949) |
|  | GAC AG[C/T] GAT | 694 | S | S | 0 | Neutral | [rs1799949](http://www.ncbi.nlm.nih.gov/projects/SNP/snp_ref.cgi?rs=rs1799949) |
|  | GAC AG[C/T] GAT | 694 | S | S | 0 | Neutral | [rs1799949](http://www.ncbi.nlm.nih.gov/projects/SNP/snp_ref.cgi?rs=rs1799949) |
|  | GAC AG[C/T] GAT | 694 | S | S | 0 | Neutral | [rs1799949](http://www.ncbi.nlm.nih.gov/projects/SNP/snp_ref.cgi?rs=rs1799949) |
|  | GAC AG[C/T] GAT | 647 | S | S | 0 | Neutral | [rs1799949](http://www.ncbi.nlm.nih.gov/projects/SNP/snp_ref.cgi?rs=rs1799949) |
|  | GAC AG[C/T] GAT | 694 | S | S | 0 | Neutral | [rs1799949](http://www.ncbi.nlm.nih.gov/projects/SNP/snp_ref.cgi?rs=rs1799949) |
| 17,41223094,T,C | CAG [A/G]GT CCA | 1317 | S | G | -0.44 | Neutral | [rs1799966](http://www.ncbi.nlm.nih.gov/projects/SNP/snp_ref.cgi?rs=rs1799966) |
|  | CAG [A/G]GT CCA | 471 | S | G | -2.09 | Neutral | [rs1799966](http://www.ncbi.nlm.nih.gov/projects/SNP/snp_ref.cgi?rs=rs1799966) |
|  | CAG [A/G]GT CCA | 430 | S | G | -1.95 | Neutral | [rs1799966](http://www.ncbi.nlm.nih.gov/projects/SNP/snp_ref.cgi?rs=rs1799966) |
|  | CAG [A/G]GT CCA | 1613 | S | G | -0.51 | Neutral | [rs1799966](http://www.ncbi.nlm.nih.gov/projects/SNP/snp_ref.cgi?rs=rs1799966) |
|  | CAG [A/G]GT CCA | 462 | S | G | -1.99 | Neutral | [rs1799966](http://www.ncbi.nlm.nih.gov/projects/SNP/snp_ref.cgi?rs=rs1799966) |
|  | CAG [A/G]GT CCA | 1634 | S | G | -0.51 | Neutral | [rs1799966](http://www.ncbi.nlm.nih.gov/projects/SNP/snp_ref.cgi?rs=rs1799966) |
|  | CAG [A/G]GT CCA | 509 | S | G | -1.86 | Neutral | [rs1799966](http://www.ncbi.nlm.nih.gov/projects/SNP/snp_ref.cgi?rs=rs1799966) |
|  | CAG [A/G]GT CCA | 1566 | S | G | -0.5 | Neutral | [rs1799966](http://www.ncbi.nlm.nih.gov/projects/SNP/snp_ref.cgi?rs=rs1799966) |
|  | CAG [A/G]GT CCA | 463 | S | G | -1.83 | Neutral | [rs1799966](http://www.ncbi.nlm.nih.gov/projects/SNP/snp_ref.cgi?rs=rs1799966) |
|  | CAG [A/G]GT CCA | 1635 | S | G | -0.51 | Neutral | [rs1799966](http://www.ncbi.nlm.nih.gov/projects/SNP/snp_ref.cgi?rs=rs1799966) |
|  | CAG [A/G]GT CCA | 384 | S | G | -1.76 | Neutral | [rs1799966](http://www.ncbi.nlm.nih.gov/projects/SNP/snp_ref.cgi?rs=rs1799966) |
|  | CAG [A/G]GT CCA | 509 | S | G | -1.45 | Neutral | [rs1799966](http://www.ncbi.nlm.nih.gov/projects/SNP/snp_ref.cgi?rs=rs1799966) |
|  | CAG [A/G]GT CCA | 509 | S | G | -1.99 | Neutral | [rs1799966](http://www.ncbi.nlm.nih.gov/projects/SNP/snp_ref.cgi?rs=rs1799966) |
| 17,41234470,A,G | GAC TC[T/C] TCT | 1436 | S | S | 0 | Neutral | [rs1060915](http://www.ncbi.nlm.nih.gov/projects/SNP/snp_ref.cgi?rs=rs1060915) |
|  | GAC TC[T/C] TCT | 1140 | S | S | 0 | Neutral | [rs1060915](http://www.ncbi.nlm.nih.gov/projects/SNP/snp_ref.cgi?rs=rs1060915) |
|  | GAC TC[T/C] TCT | 294 | S | S | 0 | Neutral | [rs1060915](http://www.ncbi.nlm.nih.gov/projects/SNP/snp_ref.cgi?rs=rs1060915) |
|  | GAC TC[T/C] TCT | 1436 | S | S | 0 | Neutral | [rs1060915](http://www.ncbi.nlm.nih.gov/projects/SNP/snp_ref.cgi?rs=rs1060915) |
|  | GAC TC[T/C] TCT | 253 | S | S | 0 | Neutral | [rs1060915](http://www.ncbi.nlm.nih.gov/projects/SNP/snp_ref.cgi?rs=rs1060915) |
|  | GAC TC[T/C] TCT | 1436 | S | S | 0 | Neutral | [rs1060915](http://www.ncbi.nlm.nih.gov/projects/SNP/snp_ref.cgi?rs=rs1060915) |
|  | GAC TC[T/C] TCT | 286 | S | S | 0 | Neutral | [rs1060915](http://www.ncbi.nlm.nih.gov/projects/SNP/snp_ref.cgi?rs=rs1060915) |
|  | GAC TC[T/C] TCT | 1436 | S | S | 0 | Neutral | [rs1060915](http://www.ncbi.nlm.nih.gov/projects/SNP/snp_ref.cgi?rs=rs1060915) |
|  | GAC TC[T/C] TCT | 333 | S | S | 0 | Neutral | [rs1060915](http://www.ncbi.nlm.nih.gov/projects/SNP/snp_ref.cgi?rs=rs1060915) |
|  | GAC TC[T/C] TCT | 201 | S | S | 0 | Neutral | [rs1060915](http://www.ncbi.nlm.nih.gov/projects/SNP/snp_ref.cgi?rs=rs1060915) |
|  | GAC TC[T/C] TCT | 208 | S | S | 0 | Neutral | [rs1060915](http://www.ncbi.nlm.nih.gov/projects/SNP/snp_ref.cgi?rs=rs1060915) |
|  | GAC TC[T/C] TCT | 1389 | S | S | 0 | Neutral | [rs1060915](http://www.ncbi.nlm.nih.gov/projects/SNP/snp_ref.cgi?rs=rs1060915) |
|  | GAC TC[T/C] TCT | 286 | S | S | 0 | Neutral | [rs1060915](http://www.ncbi.nlm.nih.gov/projects/SNP/snp_ref.cgi?rs=rs1060915) |
|  | GAC TC[T/C] TCT | 1436 | S | S | 0 | Neutral | [rs1060915](http://www.ncbi.nlm.nih.gov/projects/SNP/snp_ref.cgi?rs=rs1060915) |
|  | GAC TC[T/C] TCT | 207 | S | S | 0 | Neutral | [rs1060915](http://www.ncbi.nlm.nih.gov/projects/SNP/snp_ref.cgi?rs=rs1060915) |
|  | GAC TC[T/C] TCT | 332 | S | S | 0 | Neutral | [rs1060915](http://www.ncbi.nlm.nih.gov/projects/SNP/snp_ref.cgi?rs=rs1060915) |
|  | GAC TC[T/C] TCT | 333 | S | S | 0 | Neutral | [rs1060915](http://www.ncbi.nlm.nih.gov/projects/SNP/snp_ref.cgi?rs=rs1060915) |
| 17,41244000,T,C | CAG A[A/G]A GGA | 1183 | K | R | 0.49 | Neutral | [rs16942](http://www.ncbi.nlm.nih.gov/projects/SNP/snp_ref.cgi?rs=rs16942) |
|  | CAG A[A/G]A GGA | 887 | K | R | 0.29 | Neutral | [rs16942](http://www.ncbi.nlm.nih.gov/projects/SNP/snp_ref.cgi?rs=rs16942) |
|  | CAG A[A/G]A GGA | 1183 | K | R | 0.49 | Neutral | [rs16942](http://www.ncbi.nlm.nih.gov/projects/SNP/snp_ref.cgi?rs=rs16942) |
|  | CAG A[A/G]A GGA | 1183 | K | R | 0.4 | Neutral | [rs16942](http://www.ncbi.nlm.nih.gov/projects/SNP/snp_ref.cgi?rs=rs16942) |
|  | CAG A[A/G]A GGA | 1183 | K | R | 0.42 | Neutral | [rs16942](http://www.ncbi.nlm.nih.gov/projects/SNP/snp_ref.cgi?rs=rs16942) |
|  | CAG A[A/G]A GGA | 1136 | K | R | 0.38 | Neutral | [rs16942](http://www.ncbi.nlm.nih.gov/projects/SNP/snp_ref.cgi?rs=rs16942) |
|  | CAG A[A/G]A GGA | 1183 | K | R | 0.42 | Neutral | [rs16942](http://www.ncbi.nlm.nih.gov/projects/SNP/snp_ref.cgi?rs=rs16942) |
| 17,41245471,C,T | CAT [G/A]AC AGC | 693 | D | N | 0.03 | Neutral | [rs4986850](http://www.ncbi.nlm.nih.gov/projects/SNP/snp_ref.cgi?rs=rs4986850) |
|  | CAT [G/A]AC AGC | 397 | D | N | 0.04 | Neutral | [rs4986850](http://www.ncbi.nlm.nih.gov/projects/SNP/snp_ref.cgi?rs=rs4986850) |
|  | CAT [G/A]AC AGC | 693 | D | N | 0.03 | Neutral | [rs4986850](http://www.ncbi.nlm.nih.gov/projects/SNP/snp_ref.cgi?rs=rs4986850) |
|  | CAT [G/A]AC AGC | 693 | D | N | 0.03 | Neutral | [rs4986850](http://www.ncbi.nlm.nih.gov/projects/SNP/snp_ref.cgi?rs=rs4986850) |
|  | CAT [G/A]AC AGC | 693 | D | N | 0.02 | Neutral | [rs4986850](http://www.ncbi.nlm.nih.gov/projects/SNP/snp_ref.cgi?rs=rs4986850) |
|  | CAT [G/A]AC AGC | 646 | D | N | 0.03 | Neutral | [rs4986850](http://www.ncbi.nlm.nih.gov/projects/SNP/snp_ref.cgi?rs=rs4986850) |
|  | CAT [G/A]AC AGC | 693 | D | N | 0.02 | Neutral | [rs4986850](http://www.ncbi.nlm.nih.gov/projects/SNP/snp_ref.cgi?rs=rs4986850) |
| 1,51439777,C,T | GAA GG[C/T] CAC | 114 | G | G | 0 | Neutral | [rs1043141](http://www.ncbi.nlm.nih.gov/projects/SNP/snp_ref.cgi?rs=rs1043141) |
|  | GAA GG[C/T] CAC | 114 | G | G | 0 | Neutral | [rs1043141](http://www.ncbi.nlm.nih.gov/projects/SNP/snp_ref.cgi?rs=rs1043141) |
|  | GAA GG[C/T] CAC | 114 | G | G | 0 | Neutral | [rs1043141](http://www.ncbi.nlm.nih.gov/projects/SNP/snp_ref.cgi?rs=rs1043141) |
| 6,20490428,G,A | AGC [G/A]AT TGC | 389 | D | N | -0.01 | Neutral | [rs4134982](http://www.ncbi.nlm.nih.gov/projects/SNP/snp_ref.cgi?rs=rs4134982) |
|  | AGC [G/A]AT TGC | 258 | D | N | 0.05 | Neutral | [rs4134982](http://www.ncbi.nlm.nih.gov/projects/SNP/snp_ref.cgi?rs=rs4134982) |
| 19,45854919,T,G | CTG [A/C]AG AGG | 701 | K | Q | -0.81 | Neutral | [rs13181](http://www.ncbi.nlm.nih.gov/projects/SNP/snp_ref.cgi?rs=rs13181) |
|  | CTG [A/C]AG AGG | 727 | K | Q | -0.83 | Neutral | [rs13181](http://www.ncbi.nlm.nih.gov/projects/SNP/snp_ref.cgi?rs=rs13181) |
|  | CTG [A/C]AG AGG | 673 | K | Q | -0.62 | Neutral | [rs13181](http://www.ncbi.nlm.nih.gov/projects/SNP/snp_ref.cgi?rs=rs13181) |
|  | CTG [A/C]AG AGG | 751 | K | Q | -0.94 | Neutral | [rs13181](http://www.ncbi.nlm.nih.gov/projects/SNP/snp_ref.cgi?rs=rs13181) |
| 10,50678717,T,C | CAC [A/G]TG AGT | 1097 | M | V | 0.32 | Neutral | [rs2228526](http://www.ncbi.nlm.nih.gov/projects/SNP/snp_ref.cgi?rs=rs2228526) |
|  | CAC [A/G]TG AGT | 474 | M | V | 0.2 | Neutral | [rs2228526](http://www.ncbi.nlm.nih.gov/projects/SNP/snp_ref.cgi?rs=rs2228526) |
|  | CAC [A/G]TG AGT | 467 | M | V | 0.17 | Neutral | [rs2228526](http://www.ncbi.nlm.nih.gov/projects/SNP/snp_ref.cgi?rs=rs2228526) |
| 10,50732318,G,C | GAT GA[C/G] GAG | 386 | D | E | 0.8 | Neutral | [rs141391984](http://www.ncbi.nlm.nih.gov/projects/SNP/snp_ref.cgi?rs=rs141391984) |
|  | GAT GA[C/G] GAG | 386 | D | E | 0.49 | Neutral | [rs141391984](http://www.ncbi.nlm.nih.gov/projects/SNP/snp_ref.cgi?rs=rs141391984) |
|  | GAT GA[C/G] GAG | 386 | D | E | 0.49 | Neutral | [rs141391984](http://www.ncbi.nlm.nih.gov/projects/SNP/snp_ref.cgi?rs=rs141391984) |
| 10,50667105,T,C | CTG C[A/G]G GAA | 1413 | Q | R | -1.09 | Neutral | [rs2228529](http://www.ncbi.nlm.nih.gov/projects/SNP/snp_ref.cgi?rs=rs2228529) |
|  | CTG C[A/G]G GAA | 790 | Q | R | -1.15 | Neutral | [rs2228529](http://www.ncbi.nlm.nih.gov/projects/SNP/snp_ref.cgi?rs=rs2228529) |
|  | CTG C[A/G]G GAA | 783 | Q | R | -1.09 | Neutral | [rs2228529](http://www.ncbi.nlm.nih.gov/projects/SNP/snp_ref.cgi?rs=rs2228529) |
| 1,242035438,G,A | GAA [G/A]TG TTT | 458 | V | M | 0.48 | Neutral | [rs4149965](http://www.ncbi.nlm.nih.gov/projects/SNP/snp_ref.cgi?rs=rs4149965) |
|  | GAA [G/A]TG TTT | 458 | V | M | 0.48 | Neutral | [rs4149965](http://www.ncbi.nlm.nih.gov/projects/SNP/snp_ref.cgi?rs=rs4149965) |
|  | GAA [G/A]TG TTT | 458 | V | M | 0.48 | Neutral | [rs4149965](http://www.ncbi.nlm.nih.gov/projects/SNP/snp_ref.cgi?rs=rs4149965) |
| 20,10625804,T,G | GGA AC[A/C] ACC | 738 | T | T | 0 | Neutral | [rs1801140](http://www.ncbi.nlm.nih.gov/projects/SNP/snp_ref.cgi?rs=rs1801140) |
|  | GGA AC[A/C] ACC | 579 | T | T | 0 | Neutral | [rs1801140](http://www.ncbi.nlm.nih.gov/projects/SNP/snp_ref.cgi?rs=rs1801140) |
| 8,90967711,A,G | CAA GA[T/C] GCA | 399 | D | D | 0 | Neutral | [rs709816](http://www.ncbi.nlm.nih.gov/projects/SNP/snp_ref.cgi?rs=rs709816) |
|  | CAA GA[T/C] GCA | 317 | D | D | 0 | Neutral | [rs709816](http://www.ncbi.nlm.nih.gov/projects/SNP/snp_ref.cgi?rs=rs709816) |
|  | CAA GA[T/C] GCA | 399 | D | D | 0 | Neutral | [rs709816](http://www.ncbi.nlm.nih.gov/projects/SNP/snp_ref.cgi?rs=rs709816) |
| 4,178257364,G,C | AAA CA[G/C] AAA | 172 | Q | H | -2.36 | Neutral | [rs17064658](http://www.ncbi.nlm.nih.gov/projects/SNP/snp_ref.cgi?rs=rs17064658) |
| 3,121208176,T,C | TCT C[A/G]T GAA | 1201 | H | R | -0.47 | Neutral | [rs3218651](http://www.ncbi.nlm.nih.gov/projects/SNP/snp_ref.cgi?rs=rs3218651) |
|  | TCT C[A/G]T GAA | 1337 | H | R | -0.47 | Neutral | [rs3218651](http://www.ncbi.nlm.nih.gov/projects/SNP/snp_ref.cgi?rs=rs3218651) |
|  | TCT C[A/G]T GAA | 824 | H | R | -0.44 | Neutral | [rs3218651](http://www.ncbi.nlm.nih.gov/projects/SNP/snp_ref.cgi?rs=rs3218651) |
| 9,110084328,C,T | GGG G[C/T]T CCT | 249 | A | V | -0.99 | Neutral | [rs1805329](http://www.ncbi.nlm.nih.gov/projects/SNP/snp_ref.cgi?rs=rs1805329) |
|  | GGG G[C/T]T CCT | 177 | A | V | -0.96 | Neutral | [rs1805329](http://www.ncbi.nlm.nih.gov/projects/SNP/snp_ref.cgi?rs=rs1805329) |
| 4,39303925,A,G | GAT TC[T/C] CAC | 836 | S | S | 0 | Neutral | [rs2066782](http://www.ncbi.nlm.nih.gov/projects/SNP/snp_ref.cgi?rs=rs2066782) |
|  | GAT TC[T/C] CAC | 837 | S | S | 0 | Neutral | [rs2066782](http://www.ncbi.nlm.nih.gov/projects/SNP/snp_ref.cgi?rs=rs2066782) |
| 3,186509517,G,A | ATT GC[C/T] GGG | 266 | A | A | 0 | Neutral | [rs187868](http://www.ncbi.nlm.nih.gov/projects/SNP/snp_ref.cgi?rs=rs187868) |
|  | ATT GC[C/T] GGG | 266 | A | A | 0 | Neutral | [rs187868](http://www.ncbi.nlm.nih.gov/projects/SNP/snp_ref.cgi?rs=rs187868) |
|  | ATT GC[C/T] GGG | 266 | A | A | 0 | Neutral | [rs187868](http://www.ncbi.nlm.nih.gov/projects/SNP/snp_ref.cgi?rs=rs187868) |
|  | ATT GC[C/T] GGG | 41 | A | A | 0 | Neutral | [rs187868](http://www.ncbi.nlm.nih.gov/projects/SNP/snp_ref.cgi?rs=rs187868) |
| 12,118465820,T,C | CAC [T/C]TG TTT | 201 | L | L | 0 | Neutral | [rs5745873](http://www.ncbi.nlm.nih.gov/projects/SNP/snp_ref.cgi?rs=rs5745873) |
|  | CAC [T/C]TG TTT | 265 | L | L | 0 | Neutral | [rs5745873](http://www.ncbi.nlm.nih.gov/projects/SNP/snp_ref.cgi?rs=rs5745873) |
|  | CAC [T/C]TG TTT | 286 | L | L | 0 | Neutral | [rs5745873](http://www.ncbi.nlm.nih.gov/projects/SNP/snp_ref.cgi?rs=rs5745873) |
| 17,1782952,A,G | GAC [A/G]CA TCC | 351 | T | A | -0.41 | Neutral | [rs5030755](http://www.ncbi.nlm.nih.gov/projects/SNP/snp_ref.cgi?rs=rs5030755) |
| 3,14187449,G,T | GAG [C/A]AG CTG | 939 | Q | K | 1.67 | Neutral | [rs2228001](http://www.ncbi.nlm.nih.gov/projects/SNP/snp_ref.cgi?rs=rs2228001) |
|  | GAG [C/A]AG CTG | 902 | Q | K | 1.67 | Neutral | [rs2228001](http://www.ncbi.nlm.nih.gov/projects/SNP/snp_ref.cgi?rs=rs2228001) |
| 3,14199887,G,A | CCA G[C/T]G GCA | 499 | A | V | -0.96 | Neutral | [rs2228000](http://www.ncbi.nlm.nih.gov/projects/SNP/snp_ref.cgi?rs=rs2228000) |
|  | CCA G[C/T]G GCA | 462 | A | V | -0.96 | Neutral | [rs2228000](http://www.ncbi.nlm.nih.gov/projects/SNP/snp_ref.cgi?rs=rs2228000) |
| 22,42052998,G,A | GGC AA[G/A] ATG | 461 | K | K | 0 | Neutral | [rs147467039](http://www.ncbi.nlm.nih.gov/projects/SNP/snp_ref.cgi?rs=rs147467039) |
|  | GGC AA[G/A] ATG | 461 | K | K | 0 | Neutral | [rs147467039](http://www.ncbi.nlm.nih.gov/projects/SNP/snp_ref.cgi?rs=rs147467039) |
|  | GGC AA[G/A] ATG | 411 | K | K | 0 | Neutral | [rs147467039](http://www.ncbi.nlm.nih.gov/projects/SNP/snp_ref.cgi?rs=rs147467039) |
|  | GGC AA[G/A] ATG | 461 | K | K | 0 | Neutral | [rs147467039](http://www.ncbi.nlm.nih.gov/projects/SNP/snp_ref.cgi?rs=rs147467039) |
|  | GGC AA[G/A] ATG | 461 | K | K | 0 | Neutral | [rs147467039](http://www.ncbi.nlm.nih.gov/projects/SNP/snp_ref.cgi?rs=rs147467039) |
|  | GGC AA[G/A] ATG | 420 | K | K | 0 | Neutral | [rs147467039](http://www.ncbi.nlm.nih.gov/projects/SNP/snp_ref.cgi?rs=rs147467039) |
|  | GGC AA[G/A] ATG | 328 | K | K | 0 | Neutral | [rs147467039](http://www.ncbi.nlm.nih.gov/projects/SNP/snp_ref.cgi?rs=rs147467039) |
| 10,50740876,G,C | TAC CT[C/G] TCC | 45 | L | L | 0 | Neutral | [rs2228524](http://www.ncbi.nlm.nih.gov/projects/SNP/snp_ref.cgi?rs=rs2228524) |
|  | TAC CT[C/G] TCC | 45 | L | L | 0 | Neutral | [rs2228524](http://www.ncbi.nlm.nih.gov/projects/SNP/snp_ref.cgi?rs=rs2228524) |
|  | TAC CT[C/G] TCC | 45 | L | L | 0 | Neutral | [rs2228524](http://www.ncbi.nlm.nih.gov/projects/SNP/snp_ref.cgi?rs=rs2228524) |
|  | TAC CT[C/G] TCC | 45 | L | L | 0 | Neutral | [rs2228524](http://www.ncbi.nlm.nih.gov/projects/SNP/snp_ref.cgi?rs=rs2228524) |
| 2,48018081,A,G | TCA CC[A/G] GGA | 92 | P | P | 0 | Neutral | [rs1800932](http://www.ncbi.nlm.nih.gov/projects/SNP/snp_ref.cgi?rs=rs1800932) |
|  | TCA CC[A/G] GGA | 92 | P | P | 0 | Neutral | [rs1800932](http://www.ncbi.nlm.nih.gov/projects/SNP/snp_ref.cgi?rs=rs1800932) |
|  | TCA CC[A/G] GGA | 92 | P | P | 0 | Neutral | [rs1800932](http://www.ncbi.nlm.nih.gov/projects/SNP/snp_ref.cgi?rs=rs1800932) |
|  | TCA CC[A/G] GGA | 90 | P | P | 0 | Neutral | [rs1800932](http://www.ncbi.nlm.nih.gov/projects/SNP/snp_ref.cgi?rs=rs1800932) |
| 4,178274694,T,G | AGT GT[T/G] TGT | 424 | V | V | 0 | Neutral | [rs10007075](http://www.ncbi.nlm.nih.gov/projects/SNP/snp_ref.cgi?rs=rs10007075) |
| 4,178281754,G,A | GTG [G/A]GG AAG | 520 | G | R | 2.34 | Neutral | [rs1876268](http://www.ncbi.nlm.nih.gov/projects/SNP/snp_ref.cgi?rs=rs1876268) |
| 4,39302029,T,C | GGC CC[A/G] TTT | 847 | P | P | 0 | Neutral | [rs2066786](http://www.ncbi.nlm.nih.gov/projects/SNP/snp_ref.cgi?rs=rs2066786) |
|  | GGC CC[A/G] TTT | 848 | P | P | 0 | Neutral | [rs2066786](http://www.ncbi.nlm.nih.gov/projects/SNP/snp_ref.cgi?rs=rs2066786) |
| 6,41903782,A,C | GCC [T/G]CT CAG | 209 | S | A | -0.32 | Neutral | [rs1051130](http://www.ncbi.nlm.nih.gov/projects/SNP/snp_ref.cgi?rs=rs1051130) |
|  | GCC [T/G]CT CAG | 178 | S | A | -0.35 | Neutral | [rs1051130](http://www.ncbi.nlm.nih.gov/projects/SNP/snp_ref.cgi?rs=rs1051130) |
|  | GCC [T/G]CT CAG | 259 | S | A | -0.45 | Neutral | [rs1051130](http://www.ncbi.nlm.nih.gov/projects/SNP/snp_ref.cgi?rs=rs1051130) |
|  | GCC [T/G]CT CAG | 187 | S | A | -0.11 | Neutral | [rs1051130](http://www.ncbi.nlm.nih.gov/projects/SNP/snp_ref.cgi?rs=rs1051130) |
|  | GCC [T/G]CT CAG | 63 | S | A | -0.23 | Neutral | [rs1051130](http://www.ncbi.nlm.nih.gov/projects/SNP/snp_ref.cgi?rs=rs1051130) |
|  | AGC C[T/G]C TCA | 132 | L | R | -1.79 | Neutral | [rs1051130](http://www.ncbi.nlm.nih.gov/projects/SNP/snp_ref.cgi?rs=rs1051130) |
|  | GCC [T/G]CT CAG | 178 | S | A | -0.35 | Neutral | [rs1051130](http://www.ncbi.nlm.nih.gov/projects/SNP/snp_ref.cgi?rs=rs1051130) |
| 1,242030151,A,G | AGT C[A/G]T AGT | 354 | H | R | -0.92 | Neutral | [rs735943](http://www.ncbi.nlm.nih.gov/projects/SNP/snp_ref.cgi?rs=rs735943) |
|  | AGT C[A/G]T AGT | 354 | H | R | -0.92 | Neutral | [rs735943](http://www.ncbi.nlm.nih.gov/projects/SNP/snp_ref.cgi?rs=rs735943) |
|  | AGT C[A/G]T AGT | 354 | H | R | -0.92 | Neutral | [rs735943](http://www.ncbi.nlm.nih.gov/projects/SNP/snp_ref.cgi?rs=rs735943) |
| 2,48018081,A,G | TCA CC[A/G] GGA | 92 | P | P | 0 | Neutral | [rs1800932](http://www.ncbi.nlm.nih.gov/projects/SNP/snp_ref.cgi?rs=rs1800932) |
|  | TCA CC[A/G] GGA | 92 | P | P | 0 | Neutral | [rs1800932](http://www.ncbi.nlm.nih.gov/projects/SNP/snp_ref.cgi?rs=rs1800932) |
|  | TCA CC[A/G] GGA | 92 | P | P | 0 | Neutral | [rs1800932](http://www.ncbi.nlm.nih.gov/projects/SNP/snp_ref.cgi?rs=rs1800932) |
|  | TCA CC[A/G] GGA | 90 | P | P | 0 | Neutral | [rs1800932](http://www.ncbi.nlm.nih.gov/projects/SNP/snp_ref.cgi?rs=rs1800932) |
| 8,90967711,A,G | CAA GA[T/C] GCA | 399 | D | D | 0 | Neutral | [rs709816](http://www.ncbi.nlm.nih.gov/projects/SNP/snp_ref.cgi?rs=rs709816) |
|  | CAA GA[T/C] GCA | 317 | D | D | 0 | Neutral | [rs709816](http://www.ncbi.nlm.nih.gov/projects/SNP/snp_ref.cgi?rs=rs709816) |
|  | CAA GA[T/C] GCA | 399 | D | D | 0 | Neutral | [rs709816](http://www.ncbi.nlm.nih.gov/projects/SNP/snp_ref.cgi?rs=rs709816) |
| 3,121208833,G,C | CAG A[C/G]A TGT | 982 | T | R | -0.34 | Neutral | [rs3218649](http://www.ncbi.nlm.nih.gov/projects/SNP/snp_ref.cgi?rs=rs3218649) |
|  | CAG A[C/G]A TGT | 1118 | T | R | -0.34 | Neutral | [rs3218649](http://www.ncbi.nlm.nih.gov/projects/SNP/snp_ref.cgi?rs=rs3218649) |
|  | CAG A[C/G]A TGT | 605 | T | R | -0.28 | Neutral | [rs3218649](http://www.ncbi.nlm.nih.gov/projects/SNP/snp_ref.cgi?rs=rs3218649) |
| 9,133761001,A,G | GGT CC[A/G] GCG | 1108 | P | P | 0 | Neutral | [rs1056171](http://www.ncbi.nlm.nih.gov/projects/SNP/snp_ref.cgi?rs=rs1056171) |
|  | GGT CC[A/G] GCG | 1127 | P | P | 0 | Neutral | [rs1056171](http://www.ncbi.nlm.nih.gov/projects/SNP/snp_ref.cgi?rs=rs1056171) |
|  | GGT CC[A/G] GCG | 923 | P | P | 0 | Neutral | [rs1056171](http://www.ncbi.nlm.nih.gov/projects/SNP/snp_ref.cgi?rs=rs1056171) |
| 3,142277536,A,G | GAT GA[T/C] GGC | 605 | D | D | 0 | Neutral | [rs2227929](http://www.ncbi.nlm.nih.gov/projects/SNP/snp_ref.cgi?rs=rs2227929) |
|  | GAT GA[T/C] GGC | 541 | D | D | 0 | Neutral | [rs2227929](http://www.ncbi.nlm.nih.gov/projects/SNP/snp_ref.cgi?rs=rs2227929) |
|  | GAT GA[T/C] GGC | 222 | D | D | 0 | Neutral | [rs2227929](http://www.ncbi.nlm.nih.gov/projects/SNP/snp_ref.cgi?rs=rs2227929) |
| 3,142277575,A,T | TGT GG[T/A] ATG | 592 | G | G | 0 | Neutral | [rs2227930](http://www.ncbi.nlm.nih.gov/projects/SNP/snp_ref.cgi?rs=rs2227930) |
|  | TGT GG[T/A] ATG | 528 | G | G | 0 | Neutral | [rs2227930](http://www.ncbi.nlm.nih.gov/projects/SNP/snp_ref.cgi?rs=rs2227930) |
|  | TGT GG[T/A] ATG | 209 | G | G | 0 | Neutral | [rs2227930](http://www.ncbi.nlm.nih.gov/projects/SNP/snp_ref.cgi?rs=rs2227930) |
| 3,142281612,A,G | TTA A[T/C]G GTT | 211 | M | T | -0.2 | Neutral | [rs2227928](http://www.ncbi.nlm.nih.gov/projects/SNP/snp_ref.cgi?rs=rs2227928) |
|  | TTA A[T/C]G GTT | 211 | M | T | 0.02 | Neutral | [rs2227928](http://www.ncbi.nlm.nih.gov/projects/SNP/snp_ref.cgi?rs=rs2227928) |
| 5,68531253,C,T | AAG AA[C/T] ACC | 33 | N | N | 0 | Neutral | [rs2972388](http://www.ncbi.nlm.nih.gov/projects/SNP/snp_ref.cgi?rs=rs2972388) |
|  | AAG AA[C/T] ACC | 33 | N | N | 0 | Neutral | [rs2972388](http://www.ncbi.nlm.nih.gov/projects/SNP/snp_ref.cgi?rs=rs2972388) |
|  | AAG AA[C/T] ACC | 33 | N | N | 0 | Neutral | [rs2972388](http://www.ncbi.nlm.nih.gov/projects/SNP/snp_ref.cgi?rs=rs2972388) |
|  | AAG AA[C/T] ACC | 33 | N | N | 0 | Neutral | [rs2972388](http://www.ncbi.nlm.nih.gov/projects/SNP/snp_ref.cgi?rs=rs2972388) |
|  | AAG AA[C/T] ACC | 33 | N | N | 0 | Neutral | [rs2972388](http://www.ncbi.nlm.nih.gov/projects/SNP/snp_ref.cgi?rs=rs2972388) |
|  | AAG AA[C/T] ACC | 33 | N | N | 0 | Neutral | [rs2972388](http://www.ncbi.nlm.nih.gov/projects/SNP/snp_ref.cgi?rs=rs2972388) |
|  | AAG AA[C/T] ACC | 33 | N | N | 0 | Neutral | [rs2972388](http://www.ncbi.nlm.nih.gov/projects/SNP/snp_ref.cgi?rs=rs2972388) |
|  | AAG AA[C/T] ACC | 33 | N | N | 0 | Neutral | [rs2972388](http://www.ncbi.nlm.nih.gov/projects/SNP/snp_ref.cgi?rs=rs2972388) |
| 1,23847464,C,A | GAC CA[G/T] CTC | 226 | Q | H | -1.61 | Neutral | [rs2075995](http://www.ncbi.nlm.nih.gov/projects/SNP/snp_ref.cgi?rs=rs2075995) |
| 6,30877760,A,C | CAC AC[A/C] CAG | 98 | T | T | 0 | Neutral | [rs114336365](http://www.ncbi.nlm.nih.gov/projects/SNP/snp_ref.cgi?rs=rs114336365) |
|  | CAC AC[A/C] CAG | 98 | T | T | 0 | Neutral | [rs114336365](http://www.ncbi.nlm.nih.gov/projects/SNP/snp_ref.cgi?rs=rs114336365) |
|  | CAC AC[A/C] CAG | 98 | T | T | 0 | Neutral | [rs114336365](http://www.ncbi.nlm.nih.gov/projects/SNP/snp_ref.cgi?rs=rs114336365) |
|  | CAC AC[A/C] CAG | 42 | T | T | 0 | Neutral | [rs114336365](http://www.ncbi.nlm.nih.gov/projects/SNP/snp_ref.cgi?rs=rs114336365) |
| 3,37053568,A,G | TCC [A/G]TC TTT | 219 | I | V | -0.46 | Neutral | [rs1799977](http://www.ncbi.nlm.nih.gov/projects/SNP/snp_ref.cgi?rs=rs1799977) |
|  | TCC [A/G]TC TTT | 83 | I | V | -0.36 | Neutral | [rs1799977](http://www.ncbi.nlm.nih.gov/projects/SNP/snp_ref.cgi?rs=rs1799977) |
|  | TCC [A/G]TC TTT | 13 | I | V | -0.3 | Neutral | [rs1799977](http://www.ncbi.nlm.nih.gov/projects/SNP/snp_ref.cgi?rs=rs1799977) |
|  | TCC [A/G]TC TTT | 121 | I | V | -0.43 | Neutral | [rs1799977](http://www.ncbi.nlm.nih.gov/projects/SNP/snp_ref.cgi?rs=rs1799977) |
|  | TCC [A/G]TC TTT | 185 | I | V | -0.4 | Neutral | [rs1799977](http://www.ncbi.nlm.nih.gov/projects/SNP/snp_ref.cgi?rs=rs1799977) |
|  | TCC [A/G]TC TTT | 211 | I | V | -0.39 | Neutral | [rs1799977](http://www.ncbi.nlm.nih.gov/projects/SNP/snp_ref.cgi?rs=rs1799977) |
|  | TCC [A/G]TC TTT | 185 | I | V | -0.46 | Neutral | [rs1799977](http://www.ncbi.nlm.nih.gov/projects/SNP/snp_ref.cgi?rs=rs1799977) |
| 4,178262784,A,G | CAA C[A/G]T GTT | 286 | H | R | -0.78 | Neutral | [rs34193982](http://www.ncbi.nlm.nih.gov/projects/SNP/snp_ref.cgi?rs=rs34193982) |
| 3,121154974,T,C | GAC C[A/G]A ACA | 2513 | Q | R | 0.77 | Neutral | [rs1381057](http://www.ncbi.nlm.nih.gov/projects/SNP/snp_ref.cgi?rs=rs1381057) |
|  | GAC C[A/G]A ACA | 2649 | Q | R | 0.77 | Neutral | [rs1381057](http://www.ncbi.nlm.nih.gov/projects/SNP/snp_ref.cgi?rs=rs1381057) |
|  | GAC C[A/G]A ACA | 2136 | Q | R | 0.84 | Neutral | [rs1381057](http://www.ncbi.nlm.nih.gov/projects/SNP/snp_ref.cgi?rs=rs1381057) |
| 13,32912560,G,A | GAC TT[G/A] CTA | 1356 | L | L | 0 | Neutral | [rs28897724](http://www.ncbi.nlm.nih.gov/projects/SNP/snp_ref.cgi?rs=rs28897724) |
|  | GAC TT[G/A] CTA | 1356 | L | L | 0 | Neutral | [rs28897724](http://www.ncbi.nlm.nih.gov/projects/SNP/snp_ref.cgi?rs=rs28897724) |
| 13,32911888,A,G | AGA AA[A/G] CCA | 1132 | K | K | 0 | Neutral | [rs1801406](http://www.ncbi.nlm.nih.gov/projects/SNP/snp_ref.cgi?rs=rs1801406) |
|  | AGA AA[A/G] CCA | 1132 | K | K | 0 | Neutral | [rs1801406](http://www.ncbi.nlm.nih.gov/projects/SNP/snp_ref.cgi?rs=rs1801406) |
| 10,98078239,A,G | ATA [A/G]GA GCA | 112 | R | G | 1.15 | Neutral | [rs6584066](http://www.ncbi.nlm.nih.gov/projects/SNP/snp_ref.cgi?rs=rs6584066) |
|  | ATA [A/G]GA GCA | 112 | R | G | 1.15 | Neutral | [rs6584066](http://www.ncbi.nlm.nih.gov/projects/SNP/snp_ref.cgi?rs=rs6584066) |
| 10,50678717,T,C | CAC [A/G]TG AGT | 1097 | M | V | 0.32 | Neutral | [rs2228526](http://www.ncbi.nlm.nih.gov/projects/SNP/snp_ref.cgi?rs=rs2228526) |
|  | CAC [A/G]TG AGT | 474 | M | V | 0.2 | Neutral | [rs2228526](http://www.ncbi.nlm.nih.gov/projects/SNP/snp_ref.cgi?rs=rs2228526) |
|  | CAC [A/G]TG AGT | 467 | M | V | 0.17 | Neutral | [rs2228526](http://www.ncbi.nlm.nih.gov/projects/SNP/snp_ref.cgi?rs=rs2228526) |
| 12,124144395,A,G | CCC CC[A/G] GTT | 205 | P | P | 0 | Neutral | [rs1051793](http://www.ncbi.nlm.nih.gov/projects/SNP/snp_ref.cgi?rs=rs1051793) |
|  | CCC CC[A/G] GTT | 132 | P | P | 0 | Neutral | [rs1051793](http://www.ncbi.nlm.nih.gov/projects/SNP/snp_ref.cgi?rs=rs1051793) |
|  | CCC [A/G]GT TCA | 198 | S | G | -0.87 | Neutral | [rs1051793](http://www.ncbi.nlm.nih.gov/projects/SNP/snp_ref.cgi?rs=rs1051793) |
|  | CCC CC[A/G] GTT | 196 | P | P | 0 | Neutral | [rs1051793](http://www.ncbi.nlm.nih.gov/projects/SNP/snp_ref.cgi?rs=rs1051793) |
|  | CCC CC[A/G] GTT | 203 | P | P | 0 | Neutral | [rs1051793](http://www.ncbi.nlm.nih.gov/projects/SNP/snp_ref.cgi?rs=rs1051793) |
|  | CCC CC[A/G] GTT | 246 | P | P | 0 | Neutral | [rs1051793](http://www.ncbi.nlm.nih.gov/projects/SNP/snp_ref.cgi?rs=rs1051793) |
| 14,75483812,T,C | CAG CA[A/G] TCC | 1421 | Q | Q | 0 | Neutral | [rs13712](http://www.ncbi.nlm.nih.gov/projects/SNP/snp_ref.cgi?rs=rs13712) |
|  | CAG CA[A/G] TCC | 1445 | Q | Q | 0 | Neutral | [rs13712](http://www.ncbi.nlm.nih.gov/projects/SNP/snp_ref.cgi?rs=rs13712) |
|  | CAG CA[A/G] TCC | 383 | Q | Q | 0 | Neutral | [rs13712](http://www.ncbi.nlm.nih.gov/projects/SNP/snp_ref.cgi?rs=rs13712) |
|  | CAG CA[A/G] TCC | 116 | Q | Q | 0 | Neutral | [rs13712](http://www.ncbi.nlm.nih.gov/projects/SNP/snp_ref.cgi?rs=rs13712) |
|  | CAG CA[A/G] TCC | 469 | Q | Q | 0 | Neutral | [rs13712](http://www.ncbi.nlm.nih.gov/projects/SNP/snp_ref.cgi?rs=rs13712) |
|  | CAG CA[A/G] TCC | 1267 | Q | Q | 0 | Neutral | [rs13712](http://www.ncbi.nlm.nih.gov/projects/SNP/snp_ref.cgi?rs=rs13712) |
|  | CAG CA[A/G] TCC | 1445 | Q | Q | 0 | Neutral | [rs13712](http://www.ncbi.nlm.nih.gov/projects/SNP/snp_ref.cgi?rs=rs13712) |
| 18,51820805,G,A | AGA [G/A]CA GGA | 731 | A | T | -0.4 | Neutral | [rs8305](http://www.ncbi.nlm.nih.gov/projects/SNP/snp_ref.cgi?rs=rs8305) |
|  | AGA [G/A]CA GGA | 652 | A | T | -0.42 | Neutral | [rs8305](http://www.ncbi.nlm.nih.gov/projects/SNP/snp_ref.cgi?rs=rs8305) |
| 10,98078127,C,T | CAC AT[C/T] GTA | 74 | I | I | 0 | Neutral | [rs7081385](http://www.ncbi.nlm.nih.gov/projects/SNP/snp_ref.cgi?rs=rs7081385) |
|  | CAC AT[C/T] GTA | 74 | I | I | 0 | Neutral | [rs7081385](http://www.ncbi.nlm.nih.gov/projects/SNP/snp_ref.cgi?rs=rs7081385) |
| 20,10620275,G,A | GCG TA[C/T] ACG | 1176 | Y | Y | 0 | Neutral | [rs1051421](http://www.ncbi.nlm.nih.gov/projects/SNP/snp_ref.cgi?rs=rs1051421) |
|  | GCG TA[C/T] ACG | 1017 | Y | Y | 0 | Neutral | [rs1051421](http://www.ncbi.nlm.nih.gov/projects/SNP/snp_ref.cgi?rs=rs1051421) |
| 3,142277575,A,T | TGT GG[T/A] ATG | 592 | G | G | 0 | Neutral | [rs2227930](http://www.ncbi.nlm.nih.gov/projects/SNP/snp_ref.cgi?rs=rs2227930) |
|  | TGT GG[T/A] ATG | 528 | G | G | 0 | Neutral | [rs2227930](http://www.ncbi.nlm.nih.gov/projects/SNP/snp_ref.cgi?rs=rs2227930) |
|  | TGT GG[T/A] ATG | 209 | G | G | 0 | Neutral | [rs2227930](http://www.ncbi.nlm.nih.gov/projects/SNP/snp_ref.cgi?rs=rs2227930) |
| 3,142281612,A,G | TTA A[T/C]G GTT | 211 | M | T | -0.2 | Neutral | [rs2227928](http://www.ncbi.nlm.nih.gov/projects/SNP/snp_ref.cgi?rs=rs2227928) |
|  | TTA A[T/C]G GTT | 211 | M | T | 0.02 | Neutral | [rs2227928](http://www.ncbi.nlm.nih.gov/projects/SNP/snp_ref.cgi?rs=rs2227928) |
| 15,91354521,G,A | CCC [G/A]TA TCT | 1321 | V | I | -0.43 | Neutral | [rs7167216](http://www.ncbi.nlm.nih.gov/projects/SNP/snp_ref.cgi?rs=rs7167216) |
|  | CCC [G/A]TA TCT | 508 | V | I | -0.45 | Neutral | [rs7167216](http://www.ncbi.nlm.nih.gov/projects/SNP/snp_ref.cgi?rs=rs7167216) |
|  | CCC [G/A]TA TCT | 951 | V | I | -0.43 | Neutral | [rs7167216](http://www.ncbi.nlm.nih.gov/projects/SNP/snp_ref.cgi?rs=rs7167216) |
|  | CCC [G/A]TA TCT | 1190 | V | I | -0.2 | Neutral | [rs7167216](http://www.ncbi.nlm.nih.gov/projects/SNP/snp_ref.cgi?rs=rs7167216) |
| 15,91337479,G,A | ATA AC[G/A] GAA | 1034 | T | T | 0 | Neutral | [rs2227933](http://www.ncbi.nlm.nih.gov/projects/SNP/snp_ref.cgi?rs=rs2227933) |
|  | ATA AC[G/A] GAA | 221 | T | T | 0 | Neutral | [rs2227933](http://www.ncbi.nlm.nih.gov/projects/SNP/snp_ref.cgi?rs=rs2227933) |
|  | ATA AC[G/A] GAA | 664 | T | T | 0 | Neutral | [rs2227933](http://www.ncbi.nlm.nih.gov/projects/SNP/snp_ref.cgi?rs=rs2227933) |
|  | ATA AC[G/A] GAA | 1034 | T | T | 0 | Neutral | [rs2227933](http://www.ncbi.nlm.nih.gov/projects/SNP/snp_ref.cgi?rs=rs2227933) |
| 17,41234470,A,G | GAC TC[T/C] TCT | 1436 | S | S | 0 | Neutral | [rs1060915](http://www.ncbi.nlm.nih.gov/projects/SNP/snp_ref.cgi?rs=rs1060915) |
|  | GAC TC[T/C] TCT | 1140 | S | S | 0 | Neutral | [rs1060915](http://www.ncbi.nlm.nih.gov/projects/SNP/snp_ref.cgi?rs=rs1060915) |
|  | GAC TC[T/C] TCT | 294 | S | S | 0 | Neutral | [rs1060915](http://www.ncbi.nlm.nih.gov/projects/SNP/snp_ref.cgi?rs=rs1060915) |
|  | GAC TC[T/C] TCT | 1436 | S | S | 0 | Neutral | [rs1060915](http://www.ncbi.nlm.nih.gov/projects/SNP/snp_ref.cgi?rs=rs1060915) |
|  | GAC TC[T/C] TCT | 253 | S | S | 0 | Neutral | [rs1060915](http://www.ncbi.nlm.nih.gov/projects/SNP/snp_ref.cgi?rs=rs1060915) |
|  | GAC TC[T/C] TCT | 1436 | S | S | 0 | Neutral | [rs1060915](http://www.ncbi.nlm.nih.gov/projects/SNP/snp_ref.cgi?rs=rs1060915) |
|  | GAC TC[T/C] TCT | 286 | S | S | 0 | Neutral | [rs1060915](http://www.ncbi.nlm.nih.gov/projects/SNP/snp_ref.cgi?rs=rs1060915) |
|  | GAC TC[T/C] TCT | 1436 | S | S | 0 | Neutral | [rs1060915](http://www.ncbi.nlm.nih.gov/projects/SNP/snp_ref.cgi?rs=rs1060915) |
|  | GAC TC[T/C] TCT | 333 | S | S | 0 | Neutral | [rs1060915](http://www.ncbi.nlm.nih.gov/projects/SNP/snp_ref.cgi?rs=rs1060915) |
|  | GAC TC[T/C] TCT | 201 | S | S | 0 | Neutral | [rs1060915](http://www.ncbi.nlm.nih.gov/projects/SNP/snp_ref.cgi?rs=rs1060915) |
|  | GAC TC[T/C] TCT | 208 | S | S | 0 | Neutral | [rs1060915](http://www.ncbi.nlm.nih.gov/projects/SNP/snp_ref.cgi?rs=rs1060915) |
|  | GAC TC[T/C] TCT | 1389 | S | S | 0 | Neutral | [rs1060915](http://www.ncbi.nlm.nih.gov/projects/SNP/snp_ref.cgi?rs=rs1060915) |
|  | GAC TC[T/C] TCT | 286 | S | S | 0 | Neutral | [rs1060915](http://www.ncbi.nlm.nih.gov/projects/SNP/snp_ref.cgi?rs=rs1060915) |
|  | GAC TC[T/C] TCT | 1436 | S | S | 0 | Neutral | [rs1060915](http://www.ncbi.nlm.nih.gov/projects/SNP/snp_ref.cgi?rs=rs1060915) |
|  | GAC TC[T/C] TCT | 207 | S | S | 0 | Neutral | [rs1060915](http://www.ncbi.nlm.nih.gov/projects/SNP/snp_ref.cgi?rs=rs1060915) |
|  | GAC TC[T/C] TCT | 332 | S | S | 0 | Neutral | [rs1060915](http://www.ncbi.nlm.nih.gov/projects/SNP/snp_ref.cgi?rs=rs1060915) |
|  | GAC TC[T/C] TCT | 333 | S | S | 0 | Neutral | [rs1060915](http://www.ncbi.nlm.nih.gov/projects/SNP/snp_ref.cgi?rs=rs1060915) |
| 17,41245466,G,A | GAC AG[C/T] GAT | 694 | S | S | 0 | Neutral | [rs1799949](http://www.ncbi.nlm.nih.gov/projects/SNP/snp_ref.cgi?rs=rs1799949) |
|  | GAC AG[C/T] GAT | 398 | S | S | 0 | Neutral | [rs1799949](http://www.ncbi.nlm.nih.gov/projects/SNP/snp_ref.cgi?rs=rs1799949) |
|  | GAC AG[C/T] GAT | 694 | S | S | 0 | Neutral | [rs1799949](http://www.ncbi.nlm.nih.gov/projects/SNP/snp_ref.cgi?rs=rs1799949) |
|  | GAC AG[C/T] GAT | 694 | S | S | 0 | Neutral | [rs1799949](http://www.ncbi.nlm.nih.gov/projects/SNP/snp_ref.cgi?rs=rs1799949) |
|  | GAC AG[C/T] GAT | 694 | S | S | 0 | Neutral | [rs1799949](http://www.ncbi.nlm.nih.gov/projects/SNP/snp_ref.cgi?rs=rs1799949) |
|  | GAC AG[C/T] GAT | 647 | S | S | 0 | Neutral | [rs1799949](http://www.ncbi.nlm.nih.gov/projects/SNP/snp_ref.cgi?rs=rs1799949) |
|  | GAC AG[C/T] GAT | 694 | S | S | 0 | Neutral | [rs1799949](http://www.ncbi.nlm.nih.gov/projects/SNP/snp_ref.cgi?rs=rs1799949) |
| 17,41223094,T,C | CAG [A/G]GT CCA | 1317 | S | G | -0.44 | Neutral | [rs1799966](http://www.ncbi.nlm.nih.gov/projects/SNP/snp_ref.cgi?rs=rs1799966) |
|  | CAG [A/G]GT CCA | 471 | S | G | -2.09 | Neutral | [rs1799966](http://www.ncbi.nlm.nih.gov/projects/SNP/snp_ref.cgi?rs=rs1799966) |
|  | CAG [A/G]GT CCA | 430 | S | G | -1.95 | Neutral | [rs1799966](http://www.ncbi.nlm.nih.gov/projects/SNP/snp_ref.cgi?rs=rs1799966) |
|  | CAG [A/G]GT CCA | 1613 | S | G | -0.51 | Neutral | [rs1799966](http://www.ncbi.nlm.nih.gov/projects/SNP/snp_ref.cgi?rs=rs1799966) |
|  | CAG [A/G]GT CCA | 462 | S | G | -1.99 | Neutral | [rs1799966](http://www.ncbi.nlm.nih.gov/projects/SNP/snp_ref.cgi?rs=rs1799966) |
|  | CAG [A/G]GT CCA | 1634 | S | G | -0.51 | Neutral | [rs1799966](http://www.ncbi.nlm.nih.gov/projects/SNP/snp_ref.cgi?rs=rs1799966) |
|  | CAG [A/G]GT CCA | 509 | S | G | -1.86 | Neutral | [rs1799966](http://www.ncbi.nlm.nih.gov/projects/SNP/snp_ref.cgi?rs=rs1799966) |
|  | CAG [A/G]GT CCA | 1566 | S | G | -0.5 | Neutral | [rs1799966](http://www.ncbi.nlm.nih.gov/projects/SNP/snp_ref.cgi?rs=rs1799966) |
|  | CAG [A/G]GT CCA | 463 | S | G | -1.83 | Neutral | [rs1799966](http://www.ncbi.nlm.nih.gov/projects/SNP/snp_ref.cgi?rs=rs1799966) |
|  | CAG [A/G]GT CCA | 1635 | S | G | -0.51 | Neutral | [rs1799966](http://www.ncbi.nlm.nih.gov/projects/SNP/snp_ref.cgi?rs=rs1799966) |
|  | CAG [A/G]GT CCA | 384 | S | G | -1.76 | Neutral | [rs1799966](http://www.ncbi.nlm.nih.gov/projects/SNP/snp_ref.cgi?rs=rs1799966) |
|  | CAG [A/G]GT CCA | 509 | S | G | -1.45 | Neutral | [rs1799966](http://www.ncbi.nlm.nih.gov/projects/SNP/snp_ref.cgi?rs=rs1799966) |
|  | CAG [A/G]GT CCA | 509 | S | G | -1.99 | Neutral | [rs1799966](http://www.ncbi.nlm.nih.gov/projects/SNP/snp_ref.cgi?rs=rs1799966) |
| 17,41245237,A,G | TCA [T/C]TG GTA | 771 | L | L | 0 | Neutral | [rs16940](http://www.ncbi.nlm.nih.gov/projects/SNP/snp_ref.cgi?rs=rs16940) |
|  | TCA [T/C]TG GTA | 475 | L | L | 0 | Neutral | [rs16940](http://www.ncbi.nlm.nih.gov/projects/SNP/snp_ref.cgi?rs=rs16940) |
|  | TCA [T/C]TG GTA | 771 | L | L | 0 | Neutral | [rs16940](http://www.ncbi.nlm.nih.gov/projects/SNP/snp_ref.cgi?rs=rs16940) |
|  | TCA [T/C]TG GTA | 771 | L | L | 0 | Neutral | [rs16940](http://www.ncbi.nlm.nih.gov/projects/SNP/snp_ref.cgi?rs=rs16940) |
|  | TCA [T/C]TG GTA | 771 | L | L | 0 | Neutral | [rs16940](http://www.ncbi.nlm.nih.gov/projects/SNP/snp_ref.cgi?rs=rs16940) |
|  | TCA [T/C]TG GTA | 724 | L | L | 0 | Neutral | [rs16940](http://www.ncbi.nlm.nih.gov/projects/SNP/snp_ref.cgi?rs=rs16940) |
|  | TCA [T/C]TG GTA | 771 | L | L | 0 | Neutral | [rs16940](http://www.ncbi.nlm.nih.gov/projects/SNP/snp_ref.cgi?rs=rs16940) |
| 17,41244000,T,C | CAG A[A/G]A GGA | 1183 | K | R | 0.49 | Neutral | [rs16942](http://www.ncbi.nlm.nih.gov/projects/SNP/snp_ref.cgi?rs=rs16942) |
|  | CAG A[A/G]A GGA | 887 | K | R | 0.29 | Neutral | [rs16942](http://www.ncbi.nlm.nih.gov/projects/SNP/snp_ref.cgi?rs=rs16942) |
|  | CAG A[A/G]A GGA | 1183 | K | R | 0.49 | Neutral | [rs16942](http://www.ncbi.nlm.nih.gov/projects/SNP/snp_ref.cgi?rs=rs16942) |
|  | CAG A[A/G]A GGA | 1183 | K | R | 0.4 | Neutral | [rs16942](http://www.ncbi.nlm.nih.gov/projects/SNP/snp_ref.cgi?rs=rs16942) |
|  | CAG A[A/G]A GGA | 1183 | K | R | 0.42 | Neutral | [rs16942](http://www.ncbi.nlm.nih.gov/projects/SNP/snp_ref.cgi?rs=rs16942) |
|  | CAG A[A/G]A GGA | 1136 | K | R | 0.38 | Neutral | [rs16942](http://www.ncbi.nlm.nih.gov/projects/SNP/snp_ref.cgi?rs=rs16942) |
|  | CAG A[A/G]A GGA | 1183 | K | R | 0.42 | Neutral | [rs16942](http://www.ncbi.nlm.nih.gov/projects/SNP/snp_ref.cgi?rs=rs16942) |
| 13,32906729,A,C | GCA [A/C]AT CAG | 372 | N | H | -0.6 | Neutral | [rs144848](http://www.ncbi.nlm.nih.gov/projects/SNP/snp_ref.cgi?rs=rs144848) |
|  | GCA [A/C]AT CAG | 370 | N | H | -1.55 | Neutral | [rs144848](http://www.ncbi.nlm.nih.gov/projects/SNP/snp_ref.cgi?rs=rs144848) |
|  | GCA [A/C]AT CAG | 372 | N | H | -0.6 | Neutral | [rs144848](http://www.ncbi.nlm.nih.gov/projects/SNP/snp_ref.cgi?rs=rs144848) |
| 4,122743681,T,C | CAG [A/G]AG AAG | 112 | K | E | -1.07 | Neutral | [rs34932724](http://www.ncbi.nlm.nih.gov/projects/SNP/snp_ref.cgi?rs=rs34932724) |
| 12,56360876,G,A | GGA GA[G/A] GTG | 28 | E | E | 0 | Neutral | [rs2069398](http://www.ncbi.nlm.nih.gov/projects/SNP/snp_ref.cgi?rs=rs2069398) |
|  | GGA GA[G/A] GTG | 28 | E | E | 0 | Neutral | [rs2069398](http://www.ncbi.nlm.nih.gov/projects/SNP/snp_ref.cgi?rs=rs2069398) |
|  | GGA GA[G/A] GTG | 28 | E | E | 0 | Neutral | [rs2069398](http://www.ncbi.nlm.nih.gov/projects/SNP/snp_ref.cgi?rs=rs2069398) |
|  | GGA GA[G/A] GTG | 28 | E | E | 0 | Neutral | [rs2069398](http://www.ncbi.nlm.nih.gov/projects/SNP/snp_ref.cgi?rs=rs2069398) |
|  | GGA GA[G/A] GTG | 28 | E | E | 0 | Neutral | [rs2069398](http://www.ncbi.nlm.nih.gov/projects/SNP/snp_ref.cgi?rs=rs2069398) |
|  | GGA GA[G/A] GTG | 28 | E | E | 0 | Neutral | [rs2069398](http://www.ncbi.nlm.nih.gov/projects/SNP/snp_ref.cgi?rs=rs2069398) |
| 11,47256165,T,C | CGA A[T/C]G GTG | 215 | M | T | -2.34 | Neutral | [rs4647750](http://www.ncbi.nlm.nih.gov/projects/SNP/snp_ref.cgi?rs=rs4647750) |
|  | CGA A[T/C]G GTG | 215 | M | T | -2.04 | Neutral | [rs4647750](http://www.ncbi.nlm.nih.gov/projects/SNP/snp_ref.cgi?rs=rs4647750) |
|  | CGA A[T/C]G GTG | 151 | M | T | -2.28 | Neutral | [rs4647750](http://www.ncbi.nlm.nih.gov/projects/SNP/snp_ref.cgi?rs=rs4647750) |
| 6,20488470,G,A | CCC [G/A]CT TCC | 376 | A | T | -0.44 | Neutral | [rs147333935](http://www.ncbi.nlm.nih.gov/projects/SNP/snp_ref.cgi?rs=rs147333935) |
|  | CCC [G/A]CT TCC | 245 | A | T | -0.54 | Neutral | [rs147333935](http://www.ncbi.nlm.nih.gov/projects/SNP/snp_ref.cgi?rs=rs147333935) |
| 10,50681033,G,A | GTG GG[C/T] GGC | 917 | G | G | 0 | Neutral | [rs2229760](http://www.ncbi.nlm.nih.gov/projects/SNP/snp_ref.cgi?rs=rs2229760) |
|  | GTG GG[C/T] GGC | 294 | G | G | 0 | Neutral | [rs2229760](http://www.ncbi.nlm.nih.gov/projects/SNP/snp_ref.cgi?rs=rs2229760) |
|  | GTG GG[C/T] GGC | 287 | G | G | 0 | Neutral | [rs2229760](http://www.ncbi.nlm.nih.gov/projects/SNP/snp_ref.cgi?rs=rs2229760) |
| 1,242030151,A,G | AGT C[A/G]T AGT | 354 | H | R | -0.92 | Neutral | [rs735943](http://www.ncbi.nlm.nih.gov/projects/SNP/snp_ref.cgi?rs=rs735943) |
|  | AGT C[A/G]T AGT | 354 | H | R | -0.92 | Neutral | [rs735943](http://www.ncbi.nlm.nih.gov/projects/SNP/snp_ref.cgi?rs=rs735943) |
|  | AGT C[A/G]T AGT | 354 | H | R | -0.92 | Neutral | [rs735943](http://www.ncbi.nlm.nih.gov/projects/SNP/snp_ref.cgi?rs=rs735943) |
| 12,124144395,A,G | CCC CC[A/G] GTT | 205 | P | P | 0 | Neutral | [rs1051793](http://www.ncbi.nlm.nih.gov/projects/SNP/snp_ref.cgi?rs=rs1051793) |
|  | CCC CC[A/G] GTT | 132 | P | P | 0 | Neutral | [rs1051793](http://www.ncbi.nlm.nih.gov/projects/SNP/snp_ref.cgi?rs=rs1051793) |
|  | CCC [A/G]GT TCA | 198 | S | G | -0.87 | Neutral | [rs1051793](http://www.ncbi.nlm.nih.gov/projects/SNP/snp_ref.cgi?rs=rs1051793) |
|  | CCC CC[A/G] GTT | 196 | P | P | 0 | Neutral | [rs1051793](http://www.ncbi.nlm.nih.gov/projects/SNP/snp_ref.cgi?rs=rs1051793) |
|  | CCC CC[A/G] GTT | 203 | P | P | 0 | Neutral | [rs1051793](http://www.ncbi.nlm.nih.gov/projects/SNP/snp_ref.cgi?rs=rs1051793) |
|  | CCC CC[A/G] GTT | 246 | P | P | 0 | Neutral | [rs1051793](http://www.ncbi.nlm.nih.gov/projects/SNP/snp_ref.cgi?rs=rs1051793) |
| 6,30881103,C,A | CCC CC[C/A] ACC | 385 | P | P | 0 | Neutral | [rs1132408](http://www.ncbi.nlm.nih.gov/projects/SNP/snp_ref.cgi?rs=rs1132408) |
|  | CCC CC[C/A] ACC | 385 | P | P | 0 | Neutral | [rs1132408](http://www.ncbi.nlm.nih.gov/projects/SNP/snp_ref.cgi?rs=rs1132408) |
| 6,30877760,A,C | CAC AC[A/C] CAG | 98 | T | T | 0 | Neutral | [rs114336365](http://www.ncbi.nlm.nih.gov/projects/SNP/snp_ref.cgi?rs=rs114336365) |
|  | CAC AC[A/C] CAG | 98 | T | T | 0 | Neutral | [rs114336365](http://www.ncbi.nlm.nih.gov/projects/SNP/snp_ref.cgi?rs=rs114336365) |
|  | CAC AC[A/C] CAG | 98 | T | T | 0 | Neutral | [rs114336365](http://www.ncbi.nlm.nih.gov/projects/SNP/snp_ref.cgi?rs=rs114336365) |
|  | CAC AC[A/C] CAG | 42 | T | T | 0 | Neutral | [rs114336365](http://www.ncbi.nlm.nih.gov/projects/SNP/snp_ref.cgi?rs=rs114336365) |
| 20,10633237,G,A | CAG TA[C/T] GGC | 255 | Y | Y | 0 | Neutral | [rs1131695](http://www.ncbi.nlm.nih.gov/projects/SNP/snp_ref.cgi?rs=rs1131695) |
|  | CAG TA[C/T] GGC | 96 | Y | Y | 0 | Neutral | [rs1131695](http://www.ncbi.nlm.nih.gov/projects/SNP/snp_ref.cgi?rs=rs1131695) |
| 14,75483812,T,C | CAG CA[A/G] TCC | 1421 | Q | Q | 0 | Neutral | [rs13712](http://www.ncbi.nlm.nih.gov/projects/SNP/snp_ref.cgi?rs=rs13712) |
|  | CAG CA[A/G] TCC | 1445 | Q | Q | 0 | Neutral | [rs13712](http://www.ncbi.nlm.nih.gov/projects/SNP/snp_ref.cgi?rs=rs13712) |
|  | CAG CA[A/G] TCC | 383 | Q | Q | 0 | Neutral | [rs13712](http://www.ncbi.nlm.nih.gov/projects/SNP/snp_ref.cgi?rs=rs13712) |
|  | CAG CA[A/G] TCC | 116 | Q | Q | 0 | Neutral | [rs13712](http://www.ncbi.nlm.nih.gov/projects/SNP/snp_ref.cgi?rs=rs13712) |
|  | CAG CA[A/G] TCC | 469 | Q | Q | 0 | Neutral | [rs13712](http://www.ncbi.nlm.nih.gov/projects/SNP/snp_ref.cgi?rs=rs13712) |
|  | CAG CA[A/G] TCC | 1267 | Q | Q | 0 | Neutral | [rs13712](http://www.ncbi.nlm.nih.gov/projects/SNP/snp_ref.cgi?rs=rs13712) |
|  | CAG CA[A/G] TCC | 1445 | Q | Q | 0 | Neutral | [rs13712](http://www.ncbi.nlm.nih.gov/projects/SNP/snp_ref.cgi?rs=rs13712) |
| 2,47637439,C,T | CTC CT[C/T] ATC | 191 | L | L | 0 | Neutral | [rs1800151](http://www.ncbi.nlm.nih.gov/projects/SNP/snp_ref.cgi?rs=rs1800151) |
|  | CTC CT[C/T] ATC | 191 | L | L | 0 | Neutral | [rs1800151](http://www.ncbi.nlm.nih.gov/projects/SNP/snp_ref.cgi?rs=rs1800151) |
|  | CTC CT[C/T] ATC | 191 | L | L | 0 | Neutral | [rs1800151](http://www.ncbi.nlm.nih.gov/projects/SNP/snp_ref.cgi?rs=rs1800151) |
|  | CTC CT[C/T] ATC | 191 | L | L | 0 | Neutral | [rs1800151](http://www.ncbi.nlm.nih.gov/projects/SNP/snp_ref.cgi?rs=rs1800151) |
|  | CTC CT[C/T] ATC | 191 | L | L | 0 | Neutral | [rs1800151](http://www.ncbi.nlm.nih.gov/projects/SNP/snp_ref.cgi?rs=rs1800151) |
|  | CTC CT[C/T] ATC | 191 | L | L | 0 | Neutral | [rs1800151](http://www.ncbi.nlm.nih.gov/projects/SNP/snp_ref.cgi?rs=rs1800151) |
|  | CTC CT[C/T] ATC | 125 | L | L | 0 | Neutral | [rs1800151](http://www.ncbi.nlm.nih.gov/projects/SNP/snp_ref.cgi?rs=rs1800151) |
|  | CTC CT[C/T] ATC | 191 | L | L | 0 | Neutral | [rs1800151](http://www.ncbi.nlm.nih.gov/projects/SNP/snp_ref.cgi?rs=rs1800151) |
|  | CTC CT[C/T] ATC | 125 | L | L | 0 | Neutral | [rs1800151](http://www.ncbi.nlm.nih.gov/projects/SNP/snp_ref.cgi?rs=rs1800151) |
| 8,90955583,A,C | TAT CC[T/G] GGA | 694 | P | P | 0 | Neutral | [rs7823648](http://www.ncbi.nlm.nih.gov/projects/SNP/snp_ref.cgi?rs=rs7823648) |
|  | TAT CC[T/G] GGA | 612 | P | P | 0 | Neutral | [rs7823648](http://www.ncbi.nlm.nih.gov/projects/SNP/snp_ref.cgi?rs=rs7823648) |
| 8,90995019,C,T | ATT CT[G/A] ATT | 34 | L | L | 0 | Neutral | [rs1063045](http://www.ncbi.nlm.nih.gov/projects/SNP/snp_ref.cgi?rs=rs1063045) |
|  | ATT CT[G/A] ATT | 34 | L | L | 0 | Neutral | [rs1063045](http://www.ncbi.nlm.nih.gov/projects/SNP/snp_ref.cgi?rs=rs1063045) |
|  | ATT CT[G/A] ATT | 34 | L | L | 0 | Neutral | [rs1063045](http://www.ncbi.nlm.nih.gov/projects/SNP/snp_ref.cgi?rs=rs1063045) |
|  | ATT CT[G/A] ATT | 34 | L | L | 0 | Neutral | [rs1063045](http://www.ncbi.nlm.nih.gov/projects/SNP/snp_ref.cgi?rs=rs1063045) |
|  | ATT CT[G/A] ATT | 34 | L | L | 0 | Neutral | [rs1063045](http://www.ncbi.nlm.nih.gov/projects/SNP/snp_ref.cgi?rs=rs1063045) |
| 14,35873770, G,A | CTG GA[C/T] GAC | 27 | D | D | 0 | Neutral | [rs1957106](http://www.ncbi.nlm.nih.gov/projects/SNP/snp_ref.cgi?rs=rs1957106) |
|  | CTG GA[C/T] GAC | 27 | D | D | 0 | Neutral | [rs1957106](http://www.ncbi.nlm.nih.gov/projects/SNP/snp_ref.cgi?rs=rs1957106) |
|  | CTG GA[C/T] GAC | 27 | D | D | 0 | Neutral | [rs1957106](http://www.ncbi.nlm.nih.gov/projects/SNP/snp_ref.cgi?rs=rs1957106) |
|  | CTG GA[C/T] GAC | 27 | D | D | 0 | Neutral | [rs1957106](http://www.ncbi.nlm.nih.gov/projects/SNP/snp_ref.cgi?rs=rs1957106) |
| 2,220023045,C,T | TGG [G/A]CG TGG | 14 | A | T | -1.52 | Neutral | [rs34689457](http://www.ncbi.nlm.nih.gov/projects/SNP/snp_ref.cgi?rs=rs34689457) |
|  | TGG [G/A]CG TGG | 14 | A | T | -1.52 | Neutral | [rs34689457](http://www.ncbi.nlm.nih.gov/projects/SNP/snp_ref.cgi?rs=rs34689457) |
|  | TGG [G/A]CG TGG | 14 | A | T | -1.39 | Neutral | [rs34689457](http://www.ncbi.nlm.nih.gov/projects/SNP/snp_ref.cgi?rs=rs34689457) |
|  | TGG [G/A]CG TGG | 14 | A | T | -1.52 | Neutral | [rs34689457](http://www.ncbi.nlm.nih.gov/projects/SNP/snp_ref.cgi?rs=rs34689457) |
| 1,226570840,T,C | AAA AA[A/G] CAG | 352 | K | K | 0 | Neutral | [rs1805415](http://www.ncbi.nlm.nih.gov/projects/SNP/snp_ref.cgi?rs=rs1805415) |
| 2,190719179,T,C | GAT A[T/C]G CAT | 218 | M | T | -0.51 | Neutral | [rs1145231](http://www.ncbi.nlm.nih.gov/projects/SNP/snp_ref.cgi?rs=rs1145231) |
|  | GAT A[T/C]G CAT | 355 | M | T | -0.66 | Neutral | [rs1145231](http://www.ncbi.nlm.nih.gov/projects/SNP/snp_ref.cgi?rs=rs1145231) |
|  | GAT A[T/C]G CAT | 179 | M | T | -0.02 | Neutral | [rs1145231](http://www.ncbi.nlm.nih.gov/projects/SNP/snp_ref.cgi?rs=rs1145231) |
|  | GAT A[T/C]G CAT | 333 | M | T | -0.5 | Neutral | [rs1145231](http://www.ncbi.nlm.nih.gov/projects/SNP/snp_ref.cgi?rs=rs1145231) |
|  | GAT A[T/C]G CAT | 218 | M | T | -0.6 | Neutral | [rs1145231](http://www.ncbi.nlm.nih.gov/projects/SNP/snp_ref.cgi?rs=rs1145231) |
|  | GAT A[T/C]G CAT | 394 | M | T | -0.45 | Neutral | [rs1145231](http://www.ncbi.nlm.nih.gov/projects/SNP/snp_ref.cgi?rs=rs1145231) |
|  | GAT A[T/C]G CAT | 218 | M | T | -0.6 | Neutral | [rs1145231](http://www.ncbi.nlm.nih.gov/projects/SNP/snp_ref.cgi?rs=rs1145231) |
|  | GAT A[T/C]G CAT | 355 | M | T | -0.58 | Neutral | [rs1145231](http://www.ncbi.nlm.nih.gov/projects/SNP/snp_ref.cgi?rs=rs1145231) |
|  | GAT A[T/C]G CAT | 394 | M | T | -0.61 | Neutral | [rs1145231](http://www.ncbi.nlm.nih.gov/projects/SNP/snp_ref.cgi?rs=rs1145231) |
| 2,190719641,A,G | AAC A[A/G]A AAA | 372 | K | R | -0.54 | Neutral | [rs61736576](http://www.ncbi.nlm.nih.gov/projects/SNP/snp_ref.cgi?rs=rs61736576) |
|  | AAC A[A/G]A AAA | 509 | K | R | -0.57 | Neutral | [rs61736576](http://www.ncbi.nlm.nih.gov/projects/SNP/snp_ref.cgi?rs=rs61736576) |
|  | AAC A[A/G]A AAA | 333 | K | R | -0.59 | Neutral | [rs61736576](http://www.ncbi.nlm.nih.gov/projects/SNP/snp_ref.cgi?rs=rs61736576) |
|  | AAC A[A/G]A AAA | 487 | K | R | -0.49 | Neutral | [rs61736576](http://www.ncbi.nlm.nih.gov/projects/SNP/snp_ref.cgi?rs=rs61736576) |
|  | AAC A[A/G]A AAA | 372 | K | R | -0.42 | Neutral | [rs61736576](http://www.ncbi.nlm.nih.gov/projects/SNP/snp_ref.cgi?rs=rs61736576) |
|  | AAC A[A/G]A AAA | 548 | K | R | -0.51 | Neutral | [rs61736576](http://www.ncbi.nlm.nih.gov/projects/SNP/snp_ref.cgi?rs=rs61736576) |
|  | AAC A[A/G]A AAA | 372 | K | R | -0.42 | Neutral | [rs61736576](http://www.ncbi.nlm.nih.gov/projects/SNP/snp_ref.cgi?rs=rs61736576) |
|  | AAC A[A/G]A AAA | 509 | K | R | -0.71 | Neutral | [rs61736576](http://www.ncbi.nlm.nih.gov/projects/SNP/snp_ref.cgi?rs=rs61736576) |
|  | AAC A[A/G]A AAA | 548 | K | R | -0.67 | Neutral | [rs61736576](http://www.ncbi.nlm.nih.gov/projects/SNP/snp_ref.cgi?rs=rs61736576) |
| 2,190732559,T,C | TTA [T/C]AT AAA | 754 | Y | H | -0.06 | Neutral | [rs1145234](http://www.ncbi.nlm.nih.gov/projects/SNP/snp_ref.cgi?rs=rs1145234) |
|  | TTA [T/C]AT AAA | 416 | Y | H | -0.2 | Neutral | [rs1145234](http://www.ncbi.nlm.nih.gov/projects/SNP/snp_ref.cgi?rs=rs1145234) |
|  | TTA [T/C]AT AAA | 181 | Y | H | -0.07 | Neutral | [rs1145234](http://www.ncbi.nlm.nih.gov/projects/SNP/snp_ref.cgi?rs=rs1145234) |
|  | TTA [T/C]AT AAA | 617 | Y | H | -0.05 | Neutral | [rs1145234](http://www.ncbi.nlm.nih.gov/projects/SNP/snp_ref.cgi?rs=rs1145234) |
|  | TTA [T/C]AT AAA | 631 | Y | H | 0.84 | Neutral | [rs1145234](http://www.ncbi.nlm.nih.gov/projects/SNP/snp_ref.cgi?rs=rs1145234) |
|  | TTA [T/C]AT AAA | 617 | Y | H | -0.05 | Neutral | [rs1145234](http://www.ncbi.nlm.nih.gov/projects/SNP/snp_ref.cgi?rs=rs1145234) |
|  | TTA [T/C]AT AAA | 793 | Y | H | -0.19 | Neutral | [rs1145234](http://www.ncbi.nlm.nih.gov/projects/SNP/snp_ref.cgi?rs=rs1145234) |
| 5,131977963,T,C | GAA TA[T/C] GTG | 1282 | Y | Y | 0 | Neutral | [rs1804670](http://www.ncbi.nlm.nih.gov/projects/SNP/snp_ref.cgi?rs=rs1804670) |
|  | GAA TA[T/C] GTG | 1143 | Y | Y | 0 | Neutral | [rs1804670](http://www.ncbi.nlm.nih.gov/projects/SNP/snp_ref.cgi?rs=rs1804670) |
| 13,48955516,A,G | ACA AG[A/G] GAA | 544 | R | R | 0 | Neutral | [rs143948310](http://www.ncbi.nlm.nih.gov/projects/SNP/snp_ref.cgi?rs=rs143948310) |
|  | ACA AG[A/G] GAA | 523 | R | R | 0 | Neutral | [rs143948310](http://www.ncbi.nlm.nih.gov/projects/SNP/snp_ref.cgi?rs=rs143948310) |
| 18,20577669,G,A | AAG AA[G/A] CAA | 705 | K | K | 0 | Neutral | [rs17852769](http://www.ncbi.nlm.nih.gov/projects/SNP/snp_ref.cgi?rs=rs17852769) |
|  | AAG AA[G/A] CAA | 705 | K | K | 0 | Neutral | [rs17852769](http://www.ncbi.nlm.nih.gov/projects/SNP/snp_ref.cgi?rs=rs17852769) |
|  | AAG AA[G/A] CAA | 705 | K | K | 0 | Neutral | [rs17852769](http://www.ncbi.nlm.nih.gov/projects/SNP/snp_ref.cgi?rs=rs17852769) |
|  | AAG AA[G/A] CAA | 705 | K | K | 0 | Neutral | [rs17852769](http://www.ncbi.nlm.nih.gov/projects/SNP/snp_ref.cgi?rs=rs17852769) |
|  | AAG AA[G/A] CAA | 705 | K | K | 0 | Neutral | [rs17852769](http://www.ncbi.nlm.nih.gov/projects/SNP/snp_ref.cgi?rs=rs17852769) |
| 4,39306504,C,G | ACC CG[G/C] AGT | 680 | R | R | 0 | Neutral | [rs2066792](http://www.ncbi.nlm.nih.gov/projects/SNP/snp_ref.cgi?rs=rs2066792) |
|  | ACC CG[G/C] AGT | 681 | R | R | 0 | Neutral | [rs2066792](http://www.ncbi.nlm.nih.gov/projects/SNP/snp_ref.cgi?rs=rs2066792) |
|  | ACC CG[G/C] AGT | 143 | R | R | 0 | Neutral | [rs2066792](http://www.ncbi.nlm.nih.gov/projects/SNP/snp_ref.cgi?rs=rs2066792) |
| 13,34405447,G,A | TAT CT[G/A] AGG | 255 | L | L | 0 | Neutral | [rs9598144](http://www.ncbi.nlm.nih.gov/projects/SNP/snp_ref.cgi?rs=rs9598144) |
|  | TAT CT[G/A] AGG | 255 | L | L | 0 | Neutral | [rs9598144](http://www.ncbi.nlm.nih.gov/projects/SNP/snp_ref.cgi?rs=rs9598144) |
| 3,186509573,C,T | AGC [G/A]CT ACT | 248 | A | T | -1.91 | Neutral | [rs56354557](http://www.ncbi.nlm.nih.gov/projects/SNP/snp_ref.cgi?rs=rs56354557) |
|  | AGC [G/A]CT ACT | 248 | A | T | -1.91 | Neutral | [rs56354557](http://www.ncbi.nlm.nih.gov/projects/SNP/snp_ref.cgi?rs=rs56354557) |
|  | AGC [G/A]CT ACT | 248 | A | T | -1.78 | Neutral | [rs56354557](http://www.ncbi.nlm.nih.gov/projects/SNP/snp_ref.cgi?rs=rs56354557) |
|  | AGC [G/A]CT ACT | 23 | A | T | -2.35 | Neutral | [rs56354557](http://www.ncbi.nlm.nih.gov/projects/SNP/snp_ref.cgi?rs=rs56354557) |
| 12,118465820,T,C | CAC [T/C]TG TTT | 201 | L | L | 0 | Neutral | [rs5745873](http://www.ncbi.nlm.nih.gov/projects/SNP/snp_ref.cgi?rs=rs5745873) |
|  | CAC [T/C]TG TTT | 265 | L | L | 0 | Neutral | [rs5745873](http://www.ncbi.nlm.nih.gov/projects/SNP/snp_ref.cgi?rs=rs5745873) |
|  | CAC [T/C]TG TTT | 286 | L | L | 0 | Neutral | [rs5745873](http://www.ncbi.nlm.nih.gov/projects/SNP/snp_ref.cgi?rs=rs5745873) |
| 21,42852497,C,T | CAG [G/A]TG TAC | 160 | V | M | -1.89 | Neutral | [rs12329760](http://www.ncbi.nlm.nih.gov/projects/SNP/snp_ref.cgi?rs=rs12329760) |
|  | CAG [G/A]TG TAC | 197 | V | M | -1.89 | Neutral | [rs12329760](http://www.ncbi.nlm.nih.gov/projects/SNP/snp_ref.cgi?rs=rs12329760) |
|  | CAG [G/A]TG TAC | 160 | V | M | -1.89 | Neutral | [rs12329760](http://www.ncbi.nlm.nih.gov/projects/SNP/snp_ref.cgi?rs=rs12329760) |
|  | CAG [G/A]TG TAC | 160 | V | M | -1.89 | Neutral | [rs12329760](http://www.ncbi.nlm.nih.gov/projects/SNP/snp_ref.cgi?rs=rs12329760) |
|  | CAG [G/A]TG TAC | 120 | V | M | -1.78 | Neutral | [rs12329760](http://www.ncbi.nlm.nih.gov/projects/SNP/snp_ref.cgi?rs=rs12329760) |
| 17,7579472,G,C | CCC C[C/G]C GTG | 72 | P | R | -0.23 | Neutral | [rs1042522](http://www.ncbi.nlm.nih.gov/projects/SNP/snp_ref.cgi?rs=rs1042522) |
|  | CCC C[C/G]C GTG | 72 | P | R | -0.19 | Neutral | [rs1042522](http://www.ncbi.nlm.nih.gov/projects/SNP/snp_ref.cgi?rs=rs1042522) |
|  | CCC C[C/G]C GTG | 72 | P | R | -0.23 | Neutral | [rs1042522](http://www.ncbi.nlm.nih.gov/projects/SNP/snp_ref.cgi?rs=rs1042522) |
|  | CCC C[C/G]C GTG | 72 | P | R | -0.19 | Neutral | [rs1042522](http://www.ncbi.nlm.nih.gov/projects/SNP/snp_ref.cgi?rs=rs1042522) |
|  | CCC C[C/G]C GTG | 72 | P | R | -0.23 | Neutral | [rs1042522](http://www.ncbi.nlm.nih.gov/projects/SNP/snp_ref.cgi?rs=rs1042522) |
|  | CCC C[C/G]C GTG | 72 | P | R | -0.19 | Neutral | [rs1042522](http://www.ncbi.nlm.nih.gov/projects/SNP/snp_ref.cgi?rs=rs1042522) |
|  | CCC C[C/G]C GTG | 72 | P | R | -0.29 | Neutral | [rs1042522](http://www.ncbi.nlm.nih.gov/projects/SNP/snp_ref.cgi?rs=rs1042522) |
|  | CCC C[C/G]C GTG | 72 | P | R | -0.73 | Neutral | [rs1042522](http://www.ncbi.nlm.nih.gov/projects/SNP/snp_ref.cgi?rs=rs1042522) |
|  | CCC C[C/G]C GTG | 72 | P | R | -0.24 | Neutral | [rs1042522](http://www.ncbi.nlm.nih.gov/projects/SNP/snp_ref.cgi?rs=rs1042522) |
| 3,14187449,G,T | GAG [C/A]AG CTG | 939 | Q | K | 1.67 | Neutral | [rs2228001](http://www.ncbi.nlm.nih.gov/projects/SNP/snp_ref.cgi?rs=rs2228001) |
|  | GAG [C/A]AG CTG | 902 | Q | K | 1.67 | Neutral | [rs2228001](http://www.ncbi.nlm.nih.gov/projects/SNP/snp_ref.cgi?rs=rs2228001) |
| 19,44047550,T,C | CCG CA[A/G] GCC | 632 | Q | Q | 0 | Neutral | [rs3547](http://www.ncbi.nlm.nih.gov/projects/SNP/snp_ref.cgi?rs=rs3547) |
|  | CCG CA[A/G] GCC | 646 | Q | Q | 0 | Neutral | [rs3547](http://www.ncbi.nlm.nih.gov/projects/SNP/snp_ref.cgi?rs=rs3547) |
|  | CCG CA[A/G] GCC | 601 | Q | Q | 0 | Neutral | [rs3547](http://www.ncbi.nlm.nih.gov/projects/SNP/snp_ref.cgi?rs=rs3547) |
| 19,44047825,T,G | GAC A[A/C]T ATG | 576 | N | T | -0.17 | Neutral | [rs2307177](http://www.ncbi.nlm.nih.gov/projects/SNP/snp_ref.cgi?rs=rs2307177) |
|  | GAC A[A/C]T ATG | 590 | N | T | -0.17 | Neutral | [rs2307177](http://www.ncbi.nlm.nih.gov/projects/SNP/snp_ref.cgi?rs=rs2307177) |
|  | GAC A[A/C]T ATG | 545 | N | T | -0.2 | Neutral | [rs2307177](http://www.ncbi.nlm.nih.gov/projects/SNP/snp_ref.cgi?rs=rs2307177) |
| 17,41244936,G,A | GCT C[C/T]G TTT | 871 | P | L | 5.71 | Neutral | [rs799917](http://www.ncbi.nlm.nih.gov/projects/SNP/snp_ref.cgi?rs=rs799917) |
|  | GCT C[C/T]G TTT | 575 | P | L | 5.67 | Neutral | [rs799917](http://www.ncbi.nlm.nih.gov/projects/SNP/snp_ref.cgi?rs=rs799917) |
|  | GCT C[C/T]G TTT | 871 | P | L | 5.71 | Neutral | [rs799917](http://www.ncbi.nlm.nih.gov/projects/SNP/snp_ref.cgi?rs=rs799917) |
|  | GCT C[C/T]G TTT | 871 | P | L | 5.74 | Neutral | [rs799917](http://www.ncbi.nlm.nih.gov/projects/SNP/snp_ref.cgi?rs=rs799917) |
|  | GCT C[C/T]G TTT | 871 | P | L | 5.84 | Neutral | [rs799917](http://www.ncbi.nlm.nih.gov/projects/SNP/snp_ref.cgi?rs=rs799917) |
|  | GCT C[C/T]G TTT | 824 | P | L | 5.7 | Neutral | [rs799917](http://www.ncbi.nlm.nih.gov/projects/SNP/snp_ref.cgi?rs=rs799917) |
|  | GCT C[C/T]G TTT | 871 | P | L | 5.84 | Neutral | [rs799917](http://www.ncbi.nlm.nih.gov/projects/SNP/snp_ref.cgi?rs=rs799917) |
| 11,125525195,A,G | AAG [A/G]TT TGG | 427 | I | V | 0.4 | Neutral | [rs506504](http://www.ncbi.nlm.nih.gov/projects/SNP/snp_ref.cgi?rs=rs506504) |
|  | AAG [A/G]TT TGG | 471 | I | V | 0.38 | Neutral | [rs506504](http://www.ncbi.nlm.nih.gov/projects/SNP/snp_ref.cgi?rs=rs506504) |
|  | AAG [A/G]TT TGG | 487 | I | V | 0.42 | Neutral | [rs506504](http://www.ncbi.nlm.nih.gov/projects/SNP/snp_ref.cgi?rs=rs506504) |
|  | AAG [A/G]TT TGG | 471 | I | V | 0.38 | Neutral | [rs506504](http://www.ncbi.nlm.nih.gov/projects/SNP/snp_ref.cgi?rs=rs506504) |
|  | AAG [A/G]TT TGG | 471 | I | V | 0.38 | Neutral | [rs506504](http://www.ncbi.nlm.nih.gov/projects/SNP/snp_ref.cgi?rs=rs506504) |
|  | AAG [A/G]TT TGG | 471 | I | V | 0.38 | Neutral | [rs506504](http://www.ncbi.nlm.nih.gov/projects/SNP/snp_ref.cgi?rs=rs506504) |
|  | AAG [A/G]TT TGG | 437 | I | V | 0.35 | Neutral | [rs506504](http://www.ncbi.nlm.nih.gov/projects/SNP/snp_ref.cgi?rs=rs506504) |
| 1,242042301,G,A | TTT [G/A]AG AGC | 589 | E | K | -0.18 | Neutral | [rs1047840](http://www.ncbi.nlm.nih.gov/projects/SNP/snp_ref.cgi?rs=rs1047840) |
|  | TTT [G/A]AG AGC | 589 | E | K | -0.18 | Neutral | [rs1047840](http://www.ncbi.nlm.nih.gov/projects/SNP/snp_ref.cgi?rs=rs1047840) |
|  | TTT [G/A]AG AGC | 589 | E | K | -0.12 | Neutral | [rs1047840](http://www.ncbi.nlm.nih.gov/projects/SNP/snp_ref.cgi?rs=rs1047840) |
| 1,242042545,A,G | GAA G[A/G]G GCA | 670 | E | G | -1.55 | Neutral | [rs1776148](http://www.ncbi.nlm.nih.gov/projects/SNP/snp_ref.cgi?rs=rs1776148) |
|  | GAA G[A/G]G GCA | 670 | E | G | -1.55 | Neutral | [rs1776148](http://www.ncbi.nlm.nih.gov/projects/SNP/snp_ref.cgi?rs=rs1776148) |
|  | GAA G[A/G]G GCA | 69 | E | G | -0.79 | Neutral | [rs1776148](http://www.ncbi.nlm.nih.gov/projects/SNP/snp_ref.cgi?rs=rs1776148) |
|  | GAA G[A/G]G GCA | 670 | E | G | -1.51 | Neutral | [rs1776148](http://www.ncbi.nlm.nih.gov/projects/SNP/snp_ref.cgi?rs=rs1776148) |
| 3,121208833,G,C | CAG A[C/G]A TGT | 982 | T | R | -0.34 | Neutral | [rs3218649](http://www.ncbi.nlm.nih.gov/projects/SNP/snp_ref.cgi?rs=rs3218649) |
|  | CAG A[C/G]A TGT | 1118 | T | R | -0.34 | Neutral | [rs3218649](http://www.ncbi.nlm.nih.gov/projects/SNP/snp_ref.cgi?rs=rs3218649) |
|  | CAG A[C/G]A TGT | 605 | T | R | -0.28 | Neutral | [rs3218649](http://www.ncbi.nlm.nih.gov/projects/SNP/snp_ref.cgi?rs=rs3218649) |
| 9,133760029,C,G | CCC CC[C/G] AGG | 784 | P | P | 0 | Neutral | [rs2229070](http://www.ncbi.nlm.nih.gov/projects/SNP/snp_ref.cgi?rs=rs2229070) |
|  | CCC CC[C/G] AGG | 803 | P | P | 0 | Neutral | [rs2229070](http://www.ncbi.nlm.nih.gov/projects/SNP/snp_ref.cgi?rs=rs2229070) |
|  | CCC CC[C/G] AGG | 599 | P | P | 0 | Neutral | [rs2229070](http://www.ncbi.nlm.nih.gov/projects/SNP/snp_ref.cgi?rs=rs2229070) |
| 3,58512285,T,C | TAC TC[A/G] AAG | 418 | S | S | 0 | Neutral | [rs13097249](http://www.ncbi.nlm.nih.gov/projects/SNP/snp_ref.cgi?rs=rs13097249) |
|  | TAC TC[A/G] AAG | 404 | S | S | 0 | Neutral | [rs13097249](http://www.ncbi.nlm.nih.gov/projects/SNP/snp_ref.cgi?rs=rs13097249) |
| 1,23847464,C,A | GAC CA[G/T] CTC | 226 | Q | H | -1.61 | Neutral | [rs2075995](http://www.ncbi.nlm.nih.gov/projects/SNP/snp_ref.cgi?rs=rs2075995) |
| 2,73519629,C,A | CTG CC[G/T] GGT | 139 | P | P | 0 | Neutral |  |
|  | CTG CC[G/T] GGT | 242 | P | P | 0 | Neutral |  |
| 1,242021877,C,A | AGA [C/A]AG CTT | 205 | Q | K | -1.43 | Neutral |  |
|  | AGA [C/A]AG CTT | 165 | Q | K | -1.33 | Neutral |  |
|  | AGA [C/A]AG CTT | 205 | Q | K | -1.43 | Neutral |  |
|  | AGA [C/A]AG CTT | 162 | Q | K | -1.33 | Neutral |  |
|  | AGA [C/A]AG CTT | 165 | Q | K | -1.33 | Neutral |  |
|  | AGA [C/A]AG CTT | 205 | Q | K | -1.43 | Neutral |  |
| 1,242035382,C,T | TTT A[C/T]G AAG | 439 | T | M | -2.07 | Neutral | [rs4149963](http://www.ncbi.nlm.nih.gov/projects/SNP/snp_ref.cgi?rs=rs4149963) |
|  | TTT A[C/T]G AAG | 439 | T | M | -2.07 | Neutral | [rs4149963](http://www.ncbi.nlm.nih.gov/projects/SNP/snp_ref.cgi?rs=rs4149963) |
|  | TTT A[C/T]G AAG | 439 | T | M | -2.07 | Neutral | [rs4149963](http://www.ncbi.nlm.nih.gov/projects/SNP/snp_ref.cgi?rs=rs4149963) |
| 1,242042301,G,A | TTT [G/A]AG AGC | 589 | E | K | -0.18 | Neutral | [rs1047840](http://www.ncbi.nlm.nih.gov/projects/SNP/snp_ref.cgi?rs=rs1047840) |
|  | TTT [G/A]AG AGC | 589 | E | K | -0.18 | Neutral | [rs1047840](http://www.ncbi.nlm.nih.gov/projects/SNP/snp_ref.cgi?rs=rs1047840) |
|  | TTT [G/A]AG AGC | 589 | E | K | -0.12 | Neutral | [rs1047840](http://www.ncbi.nlm.nih.gov/projects/SNP/snp_ref.cgi?rs=rs1047840) |
| 1,242042545,A,G | GAA G[A/G]G GCA | 670 | E | G | -1.55 | Neutral | [rs1776148](http://www.ncbi.nlm.nih.gov/projects/SNP/snp_ref.cgi?rs=rs1776148) |
|  | GAA G[A/G]G GCA | 670 | E | G | -1.55 | Neutral | [rs1776148](http://www.ncbi.nlm.nih.gov/projects/SNP/snp_ref.cgi?rs=rs1776148) |
|  | GAA G[A/G]G GCA | 69 | E | G | -0.79 | Neutral | [rs1776148](http://www.ncbi.nlm.nih.gov/projects/SNP/snp_ref.cgi?rs=rs1776148) |
|  | GAA G[A/G]G GCA | 670 | E | G | -1.51 | Neutral | [rs1776148](http://www.ncbi.nlm.nih.gov/projects/SNP/snp_ref.cgi?rs=rs1776148) |
| 6,30880145,G,A | TCT GA[G/A] ATG | 333 | E | E | 0 | Neutral |  |
|  | TCT GA[G/A] ATG | 333 | E | E | 0 | Neutral |  |
| 3,129152089,G,A | TCA GG[C/T] AAA | 471 | G | G | 0 | Neutral | [rs140696](http://www.ncbi.nlm.nih.gov/projects/SNP/snp_ref.cgi?rs=rs140696) |
|  | TCA GG[C/T] AAA | 153 | G | G | 0 | Neutral | [rs140696](http://www.ncbi.nlm.nih.gov/projects/SNP/snp_ref.cgi?rs=rs140696) |
|  | TCA GG[C/T] AAA | 465 | G | G | 0 | Neutral | [rs140696](http://www.ncbi.nlm.nih.gov/projects/SNP/snp_ref.cgi?rs=rs140696) |
|  | TCA GG[C/T] AAA | 471 | G | G | 0 | Neutral | [rs140696](http://www.ncbi.nlm.nih.gov/projects/SNP/snp_ref.cgi?rs=rs140696) |
|  | TCA GG[C/T] AAA | 471 | G | G | 0 | Neutral | [rs140696](http://www.ncbi.nlm.nih.gov/projects/SNP/snp_ref.cgi?rs=rs140696) |
| 2,47709945,C,A | TTC [C/A]TG TCC | 888 | L | M | -1.37 | Neutral |  |
|  | TTC [C/A]TG TCC | 674 | L | M | -1.57 | Neutral |  |
|  | TTC [C/A]TG TCC | 822 | L | M | -1.54 | Neutral |  |
| 2,47693825,G,A | AAA CT[G/A] GAT | 513 | L | L | 0 | Neutral |  |
|  | AAA CT[G/A] GAT | 513 | L | L | 0 | Neutral |  |
|  | AAA CT[G/A] GAT | 513 | L | L | 0 | Neutral |  |
|  | AAA CT[G/A] GAT | 513 | L | L | 0 | Neutral |  |
|  | AAA CT[G/A] GAT | 299 | L | L | 0 | Neutral |  |
|  | AAA CT[G/A] GAT | 447 | L | L | 0 | Neutral |  |
| 8,90958422,T,C | AAT CC[A/G] TCT | 672 | P | P | 0 | Neutral | [rs1061302](http://www.ncbi.nlm.nih.gov/projects/SNP/snp_ref.cgi?rs=rs1061302) |
|  | AAT CC[A/G] TCT | 590 | P | P | 0 | Neutral | [rs1061302](http://www.ncbi.nlm.nih.gov/projects/SNP/snp_ref.cgi?rs=rs1061302) |
| 8,90990479,C,G | GTT [G/C]AG TCC | 185 | E | Q | 0.53 | Neutral | [rs1805794](http://www.ncbi.nlm.nih.gov/projects/SNP/snp_ref.cgi?rs=rs1805794) |
|  | GTT [G/C]AG TCC | 103 | E | Q | 0.56 | Neutral | [rs1805794](http://www.ncbi.nlm.nih.gov/projects/SNP/snp_ref.cgi?rs=rs1805794) |
|  | GTT [G/C]AG TCC | 103 | E | Q | 0.69 | Neutral | [rs1805794](http://www.ncbi.nlm.nih.gov/projects/SNP/snp_ref.cgi?rs=rs1805794) |
|  | GTT [G/C]AG TCC | 185 | E | Q | 0.63 | Neutral | [rs1805794](http://www.ncbi.nlm.nih.gov/projects/SNP/snp_ref.cgi?rs=rs1805794) |
| 8,90995019,C,T | ATT CT[G/A] ATT | 34 | L | L | 0 | Neutral | [rs1063045](http://www.ncbi.nlm.nih.gov/projects/SNP/snp_ref.cgi?rs=rs1063045) |
|  | ATT CT[G/A] ATT | 34 | L | L | 0 | Neutral | [rs1063045](http://www.ncbi.nlm.nih.gov/projects/SNP/snp_ref.cgi?rs=rs1063045) |
|  | ATT CT[G/A] ATT | 34 | L | L | 0 | Neutral | [rs1063045](http://www.ncbi.nlm.nih.gov/projects/SNP/snp_ref.cgi?rs=rs1063045) |
|  | ATT CT[G/A] ATT | 34 | L | L | 0 | Neutral | [rs1063045](http://www.ncbi.nlm.nih.gov/projects/SNP/snp_ref.cgi?rs=rs1063045) |
|  | ATT CT[G/A] ATT | 34 | L | L | 0 | Neutral | [rs1063045](http://www.ncbi.nlm.nih.gov/projects/SNP/snp_ref.cgi?rs=rs1063045) |
|  | TTT C[G/A]G AAT | 56 | R | Q | 0.29 | Neutral |  |
| 4,178257364,G,C | AAA CA[G/C] AAA | 172 | Q | H | -2.36 | Neutral | [rs17064658](http://www.ncbi.nlm.nih.gov/projects/SNP/snp_ref.cgi?rs=rs17064658) |
| 4,178274694,T,G | AGT GT[T/G] TGT | 424 | V | V | 0 | Neutral | [rs10007075](http://www.ncbi.nlm.nih.gov/projects/SNP/snp_ref.cgi?rs=rs10007075) |
| 4,178281754,G,A | GTG [G/A]GG AAG | 520 | G | R | 2.34 | Neutral | [rs1876268](http://www.ncbi.nlm.nih.gov/projects/SNP/snp_ref.cgi?rs=rs1876268) |
| 3,121186422,G,A | GCT G[C/T]A GAC | 2304 | A | V | -1.55 | Neutral | [rs532411](http://www.ncbi.nlm.nih.gov/projects/SNP/snp_ref.cgi?rs=rs532411) |
|  | GCT G[C/T]A GAC | 2440 | A | V | -1.55 | Neutral | [rs532411](http://www.ncbi.nlm.nih.gov/projects/SNP/snp_ref.cgi?rs=rs532411) |
|  | GCT G[C/T]A GAC | 1927 | A | V | -1.61 | Neutral | [rs532411](http://www.ncbi.nlm.nih.gov/projects/SNP/snp_ref.cgi?rs=rs532411) |
| 4,39353075,C,T | GGA [G/A]TA ATA | 9 | V | I | -0.3 | Neutral |  |
|  | GGA [G/A]TA ATA | 9 | V | I | -0.3 | Neutral |  |
|  | GGA [G/A]TA ATA | 9 | V | I | -0.91 | Neutral |  |
|  | GGA [G/A]TA ATA | 9 | V | I | -0.85 | Neutral |  |
| 4,39306504,C,G | ACC CG[G/C] AGT | 680 | R | R | 0 | Neutral | [rs2066792](http://www.ncbi.nlm.nih.gov/projects/SNP/snp_ref.cgi?rs=rs2066792) |
|  | ACC CG[G/C] AGT | 681 | R | R | 0 | Neutral | [rs2066792](http://www.ncbi.nlm.nih.gov/projects/SNP/snp_ref.cgi?rs=rs2066792) |
|  | ACC CG[G/C] AGT | 143 | R | R | 0 | Neutral | [rs2066792](http://www.ncbi.nlm.nih.gov/projects/SNP/snp_ref.cgi?rs=rs2066792) |
| 7,73651743,T,C | CTG GT[A/G] AAG | 263 | V | V | 0 | Neutral | [rs3135688](http://www.ncbi.nlm.nih.gov/projects/SNP/snp_ref.cgi?rs=rs3135688) |
|  | CTG GT[A/G] AAG | 229 | V | V | 0 | Neutral | [rs3135688](http://www.ncbi.nlm.nih.gov/projects/SNP/snp_ref.cgi?rs=rs3135688) |
|  | CTG GT[A/G] AAG | 56 | V | V | 0 | Neutral | [rs3135688](http://www.ncbi.nlm.nih.gov/projects/SNP/snp_ref.cgi?rs=rs3135688) |
|  | CTG GT[A/G] AAG | 119 | V | V | 0 | Neutral | [rs3135688](http://www.ncbi.nlm.nih.gov/projects/SNP/snp_ref.cgi?rs=rs3135688) |
| 3,186507789,G,A | GCA A[C/T]T GTG | 354 | T | I | -1.65 | Neutral |  |
|  | GCA A[C/T]T GTG | 354 | T | I | -1.65 | Neutral |  |
|  | GCA A[C/T]T GTG | 327 | T | I | -1.52 | Neutral |  |
| 3,186515343,C,A | GCA [G/T]CA GCT | 91 | A | S | -2.02 | Neutral | [rs113938310](http://www.ncbi.nlm.nih.gov/projects/SNP/snp_ref.cgi?rs=rs113938310) |
|  | GCA [G/T]CA GCT | 91 | A | S | -2.02 | Neutral | [rs113938310](http://www.ncbi.nlm.nih.gov/projects/SNP/snp_ref.cgi?rs=rs113938310) |
|  | GCA [G/T]CA GCT | 91 | A | S | -2.17 | Neutral | [rs113938310](http://www.ncbi.nlm.nih.gov/projects/SNP/snp_ref.cgi?rs=rs113938310) |
|  | GCA [G/T]CA GCT | 91 | A | S | -2.14 | Neutral | [rs113938310](http://www.ncbi.nlm.nih.gov/projects/SNP/snp_ref.cgi?rs=rs113938310) |
|  | GCA [G/T]CA GCT | 91 | A | S | -2.03 | Neutral | [rs113938310](http://www.ncbi.nlm.nih.gov/projects/SNP/snp_ref.cgi?rs=rs113938310) |
|  | GCA [G/T]CA GCT | 91 | A | S | -2.19 | Neutral | [rs113938310](http://www.ncbi.nlm.nih.gov/projects/SNP/snp_ref.cgi?rs=rs113938310) |
|  | GCA [G/T]CA GCT | 91 | A | S | -2.13 | Neutral | [rs113938310](http://www.ncbi.nlm.nih.gov/projects/SNP/snp_ref.cgi?rs=rs113938310) |
|  | GCA [G/T]CA GCT | 91 | A | S | -2.1 | Neutral | [rs113938310](http://www.ncbi.nlm.nih.gov/projects/SNP/snp_ref.cgi?rs=rs113938310) |
| X,96140010,C,A | AAG G[C/A]C ATC | 234 | A | D | -0.58 | Neutral |  |
| 2,48023115,T,C | GCA GA[T/C] GAA | 180 | D | D | 0 | Neutral | [rs1800935](http://www.ncbi.nlm.nih.gov/projects/SNP/snp_ref.cgi?rs=rs1800935) |
|  | GCA GA[T/C] GAA | 81 | D | D | 0 | Neutral | [rs1800935](http://www.ncbi.nlm.nih.gov/projects/SNP/snp_ref.cgi?rs=rs1800935) |
|  | GCA GA[T/C] GAA | 81 | D | D | 0 | Neutral | [rs1800935](http://www.ncbi.nlm.nih.gov/projects/SNP/snp_ref.cgi?rs=rs1800935) |
|  | GCA GA[T/C] GAA | 81 | D | D | 0 | Neutral | [rs1800935](http://www.ncbi.nlm.nih.gov/projects/SNP/snp_ref.cgi?rs=rs1800935) |
|  | GCA GA[T/C] GAA | 180 | D | D | 0 | Neutral | [rs1800935](http://www.ncbi.nlm.nih.gov/projects/SNP/snp_ref.cgi?rs=rs1800935) |
|  | GCA GA[T/C] GAA | 178 | D | D | 0 | Neutral | [rs1800935](http://www.ncbi.nlm.nih.gov/projects/SNP/snp_ref.cgi?rs=rs1800935) |
| 4,178274565,A,G | AAC AG[A/G] AAA | 381 | R | R | 0 | Neutral | [rs113130668](http://www.ncbi.nlm.nih.gov/projects/SNP/snp_ref.cgi?rs=rs113130668) |
| 4,178274750,C,T | CAA C[C/T]A TCC | 443 | P | L | -0.36 | Neutral | [rs13112358](http://www.ncbi.nlm.nih.gov/projects/SNP/snp_ref.cgi?rs=rs13112358) |
| 4,178274835,A,C | GCC CA[A/C] TAC | 471 | Q | H | 1.51 | Neutral | [rs13112390](http://www.ncbi.nlm.nih.gov/projects/SNP/snp_ref.cgi?rs=rs13112390) |
| 7,6026988,G,A | AGA [C/T]CT CAG | 470 | P | S | 0.2 | Neutral | [rs1805321](http://www.ncbi.nlm.nih.gov/projects/SNP/snp_ref.cgi?rs=rs1805321) |
|  | AGA [C/T]CT CAG | 423 | P | S | 0.36 | Neutral | [rs1805321](http://www.ncbi.nlm.nih.gov/projects/SNP/snp_ref.cgi?rs=rs1805321) |
|  | AGA [C/T]CT CAG | 470 | P | S | 0.27 | Neutral | [rs1805321](http://www.ncbi.nlm.nih.gov/projects/SNP/snp_ref.cgi?rs=rs1805321) |
|  | AGA [C/T]CT CAG | 364 | P | S | 0.11 | Neutral | [rs1805321](http://www.ncbi.nlm.nih.gov/projects/SNP/snp_ref.cgi?rs=rs1805321) |
| 7,6036980,G,C | TGT TC[C/G] GAT | 260 | S | S | 0 | Neutral | [rs1805319](http://www.ncbi.nlm.nih.gov/projects/SNP/snp_ref.cgi?rs=rs1805319) |
|  | TGT TC[C/G] GAT | 260 | S | S | 0 | Neutral | [rs1805319](http://www.ncbi.nlm.nih.gov/projects/SNP/snp_ref.cgi?rs=rs1805319) |
|  | TGT TC[C/G] GAT | 213 | S | S | 0 | Neutral | [rs1805319](http://www.ncbi.nlm.nih.gov/projects/SNP/snp_ref.cgi?rs=rs1805319) |
|  | TGT TC[C/G] GAT | 260 | S | S | 0 | Neutral | [rs1805319](http://www.ncbi.nlm.nih.gov/projects/SNP/snp_ref.cgi?rs=rs1805319) |
|  | TGT TC[C/G] GAT | 154 | S | S | 0 | Neutral | [rs1805319](http://www.ncbi.nlm.nih.gov/projects/SNP/snp_ref.cgi?rs=rs1805319) |
| 3,121208833,G,C | CAG A[C/G]A TGT | 982 | T | R | -0.34 | Neutral | [rs3218649](http://www.ncbi.nlm.nih.gov/projects/SNP/snp_ref.cgi?rs=rs3218649) |
|  | CAG A[C/G]A TGT | 1118 | T | R | -0.34 | Neutral | [rs3218649](http://www.ncbi.nlm.nih.gov/projects/SNP/snp_ref.cgi?rs=rs3218649) |
|  | CAG A[C/G]A TGT | 605 | T | R | -0.28 | Neutral | [rs3218649](http://www.ncbi.nlm.nih.gov/projects/SNP/snp_ref.cgi?rs=rs3218649) |
| 1,3638674,C,T | GGC AC[C/T] GCC | 173 | T | T | 0 | Neutral | [rs1801174](http://www.ncbi.nlm.nih.gov/projects/SNP/snp_ref.cgi?rs=rs1801174) |
|  | GGC AC[C/T] GCC | 173 | T | T | 0 | Neutral | [rs1801174](http://www.ncbi.nlm.nih.gov/projects/SNP/snp_ref.cgi?rs=rs1801174) |
|  | GGC AC[C/T] GCC | 173 | T | T | 0 | Neutral | [rs1801174](http://www.ncbi.nlm.nih.gov/projects/SNP/snp_ref.cgi?rs=rs1801174) |
|  | GGC AC[C/T] GCC | 124 | T | T | 0 | Neutral | [rs1801174](http://www.ncbi.nlm.nih.gov/projects/SNP/snp_ref.cgi?rs=rs1801174) |
|  | GGC AC[C/T] GCC | 124 | T | T | 0 | Neutral | [rs1801174](http://www.ncbi.nlm.nih.gov/projects/SNP/snp_ref.cgi?rs=rs1801174) |
|  | GGC AC[C/T] GCC | 124 | T | T | 0 | Neutral | [rs1801174](http://www.ncbi.nlm.nih.gov/projects/SNP/snp_ref.cgi?rs=rs1801174) |
|  | GGC AC[C/T] GCC | 102 | T | T | 0 | Neutral | [rs1801174](http://www.ncbi.nlm.nih.gov/projects/SNP/snp_ref.cgi?rs=rs1801174) |
|  | GGC AC[C/T] GCC | 173 | T | T | 0 | Neutral | [rs1801174](http://www.ncbi.nlm.nih.gov/projects/SNP/snp_ref.cgi?rs=rs1801174) |
| 11,108202739,C,A | GTG C[C/A]T AAA | 2588 | P | H | -1.93 | Neutral |  |
|  | GTG C[C/A]T AAA | 2588 | P | H | -1.93 | Neutral |  |
| 3,142226881,C,A | ACT CT[G/T] GCA | 1641 | L | L | 0 | Neutral |  |
|  | ACT CT[G/T] GCA | 1577 | L | L | 0 | Neutral |  |
| 8,86115473,C,A | ATG GA[C/A] GAT | 163 | D | E | -0.21 | Neutral |  |
|  | ATG GA[C/A] GAT | 163 | D | E | -0.34 | Neutral |  |
|  | ATG GA[C/A] GAT | 163 | D | E | -0.34 | Neutral |  |
|  | ATG GA[C/A] GAT | 2 | D | E | 0.16 | Neutral |  |
| 11,94180485,G,T | GAT AG[C/A] ATC | 561 | S | R | -1.16 | Neutral |  |
|  | GAT AG[C/A] ATC | 561 | S | R | -1.33 | Neutral |  |
|  | GAT AG[C/A] ATC | 561 | S | R | -1.13 | Neutral |  |
|  | GAT AG[C/A] ATC | 564 | S | R | -1.16 | Neutral |  |
| 4,178274781,C,T | ACA AT[C/T] AGT | 453 | I | I | 0 | Neutral |  |
| 7,6026908,G,A | GGG CA[C/T] GGC | 496 | H | H | 0 | Neutral | [rs1805320](http://www.ncbi.nlm.nih.gov/projects/SNP/snp_ref.cgi?rs=rs1805320) |
|  | GGG CA[C/T] GGC | 449 | H | H | 0 | Neutral | [rs1805320](http://www.ncbi.nlm.nih.gov/projects/SNP/snp_ref.cgi?rs=rs1805320) |
|  | GGG CA[C/T] GGC | 496 | H | H | 0 | Neutral | [rs1805320](http://www.ncbi.nlm.nih.gov/projects/SNP/snp_ref.cgi?rs=rs1805320) |
|  | GGG CA[C/T] GGC | 390 | H | H | 0 | Neutral | [rs1805320](http://www.ncbi.nlm.nih.gov/projects/SNP/snp_ref.cgi?rs=rs1805320) |
| 6,43565446,A,T | CGA AA[A/T] CAA | 168 | K | N | -0.64 | Neutral |  |
|  | CGA AA[A/T] CAA | 168 | K | N | -0.64 | Neutral |  |
|  | CGA AA[A/T] CAA | 106 | K | N | -0.51 | Neutral |  |
| 6,43581898,G,A | GTG TC[G/A] AAG | 582 | S | S | 0 | Neutral |  |
|  | GTG TC[G/A] AAG | 520 | S | S | 0 | Neutral |  |
| 2,216981384,G,T | CAG GT[G/T] TTT | 46 | V | V | 0 | Neutral |  |
|  | CAG GT[G/T] TTT | 46 | V | V | 0 | Neutral |  |
|  | CAG GT[G/T] TTT | 33 | V | V | 0 | Neutral |  |
| 2,217054974,G,A | ATC [G/A]AA CAG | 621 | E | K | -0.41 | Neutral |  |
|  | ATC [G/A]AA CAG | 621 | E | K | -0.41 | Neutral |  |
| 20,10653469,C,T | GCC GG[G/A] GGG | 89 | G | G | 0 | Neutral | [rs1051415](http://www.ncbi.nlm.nih.gov/projects/SNP/snp_ref.cgi?rs=rs1051415) |
| 4,39302029,T,C | GGC CC[A/G] TTT | 847 | P | P | 0 | Neutral | [rs2066786](http://www.ncbi.nlm.nih.gov/projects/SNP/snp_ref.cgi?rs=rs2066786) |
|  | GGC CC[A/G] TTT | 848 | P | P | 0 | Neutral | [rs2066786](http://www.ncbi.nlm.nih.gov/projects/SNP/snp_ref.cgi?rs=rs2066786) |
| 5,79950718,G,C | GCC [G/C]CA GCG | 58 | A | P | 1.64 | Neutral | [rs148550291](http://www.ncbi.nlm.nih.gov/projects/SNP/snp_ref.cgi?rs=rs148550291) |
| 5,79950708,T,C | GCG GC[T/C] GCA | 54 | A | A | 0 | Neutral | [rs2405875](http://www.ncbi.nlm.nih.gov/projects/SNP/snp_ref.cgi?rs=rs2405875) |
|  | GCG GC[T/C] GCA | 54 | A | A | 0 | Neutral | [rs2405875](http://www.ncbi.nlm.nih.gov/projects/SNP/snp_ref.cgi?rs=rs2405875) |
| 5,79950715,G,C | GCG [G/C]CC GCA | 57 | A | P | 0.48 | Neutral | [rs144776112](http://www.ncbi.nlm.nih.gov/projects/SNP/snp_ref.cgi?rs=rs144776112) |
| 4,39302029,T,C | GGC CC[A/G] TTT | 847 | P | P | 0 | Neutral | [rs2066786](http://www.ncbi.nlm.nih.gov/projects/SNP/snp_ref.cgi?rs=rs2066786) |
|  | GGC CC[A/G] TTT | 848 | P | P | 0 | Neutral | [rs2066786](http://www.ncbi.nlm.nih.gov/projects/SNP/snp_ref.cgi?rs=rs2066786) |
| 0 50678717 T C |  |  |  |  |  |  |  |
| 5,74880753,T,G |  |  |  |  |  |  |  |
| 8,42213086,G,A |  |  |  |  |  |  |  |
| 5,74872759,G,A |  |  |  |  |  |  |  |
| 5,74877275,T,C |  |  |  |  |  |  |  |
| 9,45855890,C,A |  |  |  |  |  |  |  |
| 7,41231399,G,T |  |  |  |  |  |  |  |
| 12,75513828,G,A |  |  |  |  |  |  |  |
| 13,35872049,G,A |  |  |  |  |  |  |  |
|  |  |  |  |  |  |  |  |
